# Simulation of the VUV Absorption Spectra of Oxygenates and Hydrocarbons: A Joint Theoretical–Experimental Study

**DOI:** 10.1021/acs.jpca.2c07743

**Published:** 2023-04-25

**Authors:** Addison K. Bralick, Erica C. Mitchell, Anna C. Doner, Annabelle R. Webb, Matthew G. Christianson, Justin M. Turney, Brandon Rotavera, Henry F. Schaefer

**Affiliations:** † Department of Chemistry, University of Georgia, 302 East Campus Road, Athens, Georgia 30602, United States; ‡ Center for Computational Quantum Chemistry, University of Georgia, 1004 Cedar Street, Athens, Georgia 30602, United States; § College of Engineering, University of Georgia, 597 D.W. Brooks Drive, Athens, Georgia 30602, United States

## Abstract

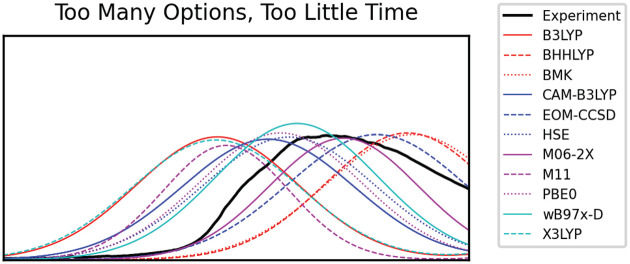

Vacuum UV absorption spectroscopy is regularly used to provide unambiguous identification of a target species, insight into the electronic structure of molecules, and quantitative species concentrations. As molecules of interest have become more complex, theoretical spectra have been used in tandem with laboratory spectroscopic analysis or as a replacement when experimental data is unavailable. However, it is difficult to determine which theoretical methodologies can best simulate experiment. This study examined the performance of EOM-CCSD and 10 TD-DFT functionals (B3LYP, BH&HLYP, BMK, CAM-B3LYP, HSE, M06-2X, M11, PBE0, ωB97X-D, and X3LYP) to produce reliable vacuum UV absorption spectra for 19 small oxygenates and hydrocarbons using vertical excitation energies. The simulated spectra were analyzed against experiment using both a qualitative analysis and quantitative metrics, including cosine similarity, relative integral change, mean signed error, and mean absolute error. Based on our ranking system, it was determined that M06-2X was consistently the top performing TD-DFT method with BMK, CAM-B3LYP, and ωB97X-D also producing reliable spectra for these small combustion species.

## Introduction

Vacuum ultraviolet (VUV) absorption spectroscopy is a powerful analytical tool that can probe high-energy valence electronic transitions (e.g., σ → σ* and *n* → σ*) and Rydberg transitions, which are common in hydrocarbons and oxygenates.^1−3^ By accessing these transitions, a molecule’s VUV absorption spectrum allows the definitive detection of the target species, a more complete description of the species’ electronic structure, and quantitative, isomer-resolved speciation measurements.^1−4^ Despite its applicability, experimental VUV spectroscopy does have limitations. Primarily, it requires the target molecule to be feasible to synthesize and isolate at sufficient yield and purity. Additionally, if the target molecule has multiple functional groups it becomes difficult to resolve the peaks in the spectra due to overlapping transitions. As molecules of interest become larger and more complex, the ability to fully analyze them using only experimental spectroscopy becomes less practical. To combat this, experimental chemists can use theoretical results to aid in their analysis.^5−9^ Theoretical computations provide clear excitation energies and allow for more straightforward orbital and electronic structure analysis. However, their utility is limited by the accuracy of the method. The need for highly accurate and cost-efficient methods for computing excited-state energies, transition dipole moments, and oscillator strengths is one that many theoretical chemists have taken an interest in,^10−16^ as these properties are crucial in analyzing and understanding target molecules and their possible uses.

Two common methods for computing excited-state properties are time-dependent density functional theory (TD-DFT) and equation-of-motion coupled cluster (EOM-CC). The accuracy of both methods has been previously studied and most benchmarks conclude that highly correlated ab initio methods perform better than TD-DFT methods.^10−12,14,15,17−30^ However, the higher accuracy methods are also significantly more computationally expensive. One of the most well-known benchmarks for excited-state calculations was done by Thiel and co-workers using CASPT2, CC2, linear-response CCSD, and CC3.^10^ That study established a set of 28 medium-sized organic molecules, aptly named Thiel’s set, and the theoretical best estimates that many later benchmarks reference. Despite its thoroughness, this study was limited by the computational cost of the chosen methods. Namely, the basis set chosen did not include diffuse functions, which the study emphasized was “insufficient for those excited states that are spatially extended and have (partial) Rydberg character”.^10^ Indeed, two years later, Thiel and co-workers updated their TBE using the aug-cc-pVTZ basis set and found that the inclusion of diffuse functions caused a shift in the computed vertical excitation energies and first-order properties.^17^ Because of the impact diffuse functions had on the energies and first-order properties, the current study utilizes the heavily augmented d-aug-cc-pVTZ basis set.

In order to make the computations tractable for large molecules, studies often utilize TD-DFT. Due to the number of functionals available, there have been numerous past studies benchmarking TD-DFT methods.^11,14,15,18,21,23−31^ Notably, Jacquemin and co-workers have published several TD-DFT benchmarks,^11−13,18,21,22,24,25,28,29,32−38^ including a general review of TD-DFT benchmarks of transition energies.^22^ Although these past studies provide some useful insights, there is still much debate as to which functionals are ideal. For example, hybrid functionals containing 20–40% Hartree–Fock exact exchange energy tend to perform better, but conclusions regarding which type of functional (traditional, meta, or range-separated) are more ambiguous.^11,13,24,25,29^ Functional dependence of first-order properties, such as oscillator strengths, also needs to be considered.^13,26,39,40^

In many of the previously mentioned benchmarks, theoretical excitation energies are compared to either the maximum absorbance wavelength obtained from experimental data or a theoretical best estimate. The issue with the former arises with the fact that vertical excitation energies are not directly comparable to any experimental observables, such as the maximum absorbance wavelength.^24,37,41−47^ Past studies have shown that vertical excitation energies tend to be blue-shifted compared to the experimental band maximum.^41,46,48−50^ Thus, directly comparing the two can lead to systematic errors in the analysis. One way to combat this shift is to go beyond a simple vertical excitation energy and compute either an adiabatic excitation energy or a 0–0 excitation energy. However, both the adiabatic excitation energy and the 0–0 excitation energy require optimization of the excited-state structure, and the 0–0 excitation energy requires a vibrational analysis of the excited-state structure. While achievable, especially at the DFT level of theory, these extra computations make obtaining theoretical excitation energies more complex and computationally demanding. Another way to ensure that the analysis is not subject to systematic bias is to compare the spectral band shapes, rather than individual energies. By approximating the spectral shape using a Gaussian broadening function and calculated oscillator strengths (eq 1), we are able to compare the theoretically produced spectra to experimental with minimal additional computational cost. A qualitative analysis of the spectra can be used to determine accuracy while bypassing the systematic error of vertical excitation energies.^42,46^

In the present research, the accuracy of 10 TD-DFT functionals and the EOM-CCSD method at simulating the experimental spectra from excitation energies and first-order properties within the vertical approximation are determined. The methods are benchmarked for 19 small organic molecules that are important in many areas of chemistry, such as atmospheric, interstellar, catalytic, and combustion chemistry. The predicted spectra are examined against gas-phase vacuum ultraviolet (VUV) absorption spectra to allow for a direct comparison between theory and experiment. By comparing to high-resolution experimental spectra, we strive to ensure that the theoretical spectra can be used in tandem with experimental analysis. We also predict which TD-DFT functionals could produce reliable spectra for molecules that cannot be experimentally characterized. With this study, we aim to provide insight into method accuracy for computing theoretical spectra and a foundation for predicting spectra of larger, more complex molecules that are unobtainable experimentally.

## Methods

### Computational

**Figure 1:**
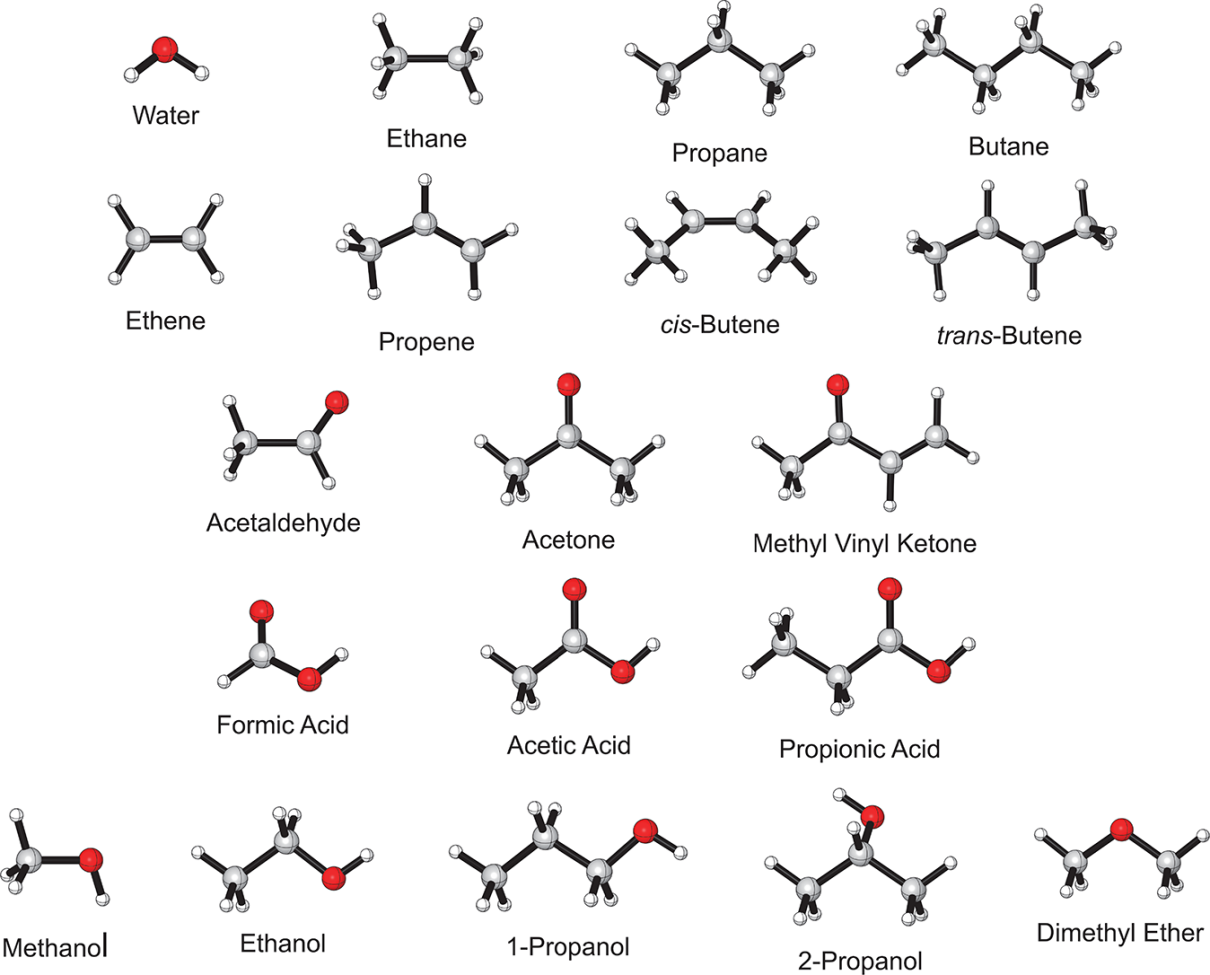
Set of 19 small molecules for which spectra are produced and analyzed.

This study compares the theoretical and experimental spectra for a set of 19 molecules depicted in Figure 1. These molecules were investigated using TD-DFT^51−53^ and EOM-CCSD.^54−57^ Based on past benchmarks, 10 TD-DFT functionals were chosen: B3LYP,^58,59^ BH&HLYP,^60^ BMK,^61^ CAM-B3LYP,^62^ HSE,^63,64^ M06-2X,^65,66^ M11,^67^ PBE0,^68,69^ ωB97X-D,^70,71^ and X3LYP.^72^ Ground-state molecular structures were computed for each of the 19 molecules using the 10 DFT functionals listed and Dunning’s d-aug-cc-pVTZ basis set^73−75^ taken from the Basis Set Exchange.^76−78^ For the geometry optimizations, the RMS force was converged to 10^–6^, and the energy was converged to 10^–10^. Harmonic vibrational frequencies were then computed using the same level of theory and basis set to confirm the geometries are genuine minima. The doubly augmented basis set and Lebedev-Treutler (99, 590) grid were both necessary to properly characterize Rydberg excitations.^79−81^ All DFT geometries and frequencies were computed using Psi4 1.4.^82,83^ For the EOM-CCSD method, ground-state geometries were computed using the CCSD(T)^84−87^ level of theory with Dunning’s d-aug-cc-pVTZ basis set. For the geometry optimizations, the RMS force was converged to 10^–7^ and the energy, coupled cluster amplitudes, and lambda equations converged to 10^–10^. Again, harmonic vibrational frequencies were computed using the same level of theory and basis set to confirm the ground state structures. CC geometries and frequencies were performed using Molpro 2010.1.^88−92^

Excitation energies, transition dipole moments, and oscillator strengths were determined using the same 10 TD-DFT functionals and the EOM-CCSD level of theory with the d-aug-cc-pVTZ basis set. The TD-DFT utilized the random-phase approximation and the SG-3 grid. Convergence criteria for both methods was set to the QChem defaults. TD-DFT excited-state computations were done using QChem 5.0,^93^ and EOM-CCSD excited-state computations were done using QChem 4.4.^94^ From the excited-state properties, absorption cross-section spectra were determined through convolution of the oscillator strengths by use of a Gaussian function,
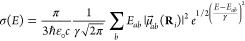
1The shape of the spectra depends heavily on the bandwidth parameter, γ, of the Gaussian. The bandwidth parameters were chosen so that the theoretical data aligns with the onset energy of the experimental data. For each molecule, we took the experimental onset energy to be the slope of the first peak except for 2-propanol, where the slope of the second peak was chosen. Alignment of the theoretical and experimental onset energies allows for quantitative data analysis from the theoretical spectrum. In cases where the onset energies do not align, the bandwidth parameter was selected so that the predicted spectra best mimics the experimental spectra. The spectra were produced by a modified version of the spectrum.py add-on to Psi4.

In order to compare spectra, four analytical techniques were used. First, the cosine-similarity expression was utilized,
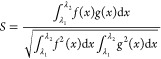
2where the range to examine the similarity was taken to best match the onset energy curve. With eq 2, similarity values, *S*, fall between 0 and 1 with 1 being a 100% match. The relative integral change was also used,
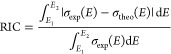
3where again, the range is selected to match the experimental onset energy. Values for relative integral change correspond to the difference in area under the two curves, with lower values indicating a closer match. Finally, the mean signed error (MSE) and mean average error (MAE) for the selected energy range is calculated. Along with the quantitative metrics, a qualitative analysis of the theoretically produced spectra was done. For this qualitative analysis, overall spectral shapes, including peak location, height, and width, were considered and accuracy determined by how closely the theoretical spectra mimic the experimental spectra.

### Experimental Section

The experimental method used to measure absorption cross sections is described in detail by Doner and co-workers.^1^ As a concise overview, differential absorption spectroscopy measurements were conducted on species in the gas phase using a temperature- and pressure-controlled flow cell with a path length of 10 cm. Prior to each absorption measurement, a reference scan (to measure background absorbance) and a dark scan (with the light source, a lamp, blocked) were performed sequentially using He flow for 1 min to enable background-subtraction and proper determination of the incident intensity, *I*_0_. Time-averaged absorption signals were then measured and repeated to enable statistical calculations for uncertainty quantification.

Absorption cross sections were quantified as a function of photon energy, σ(*E*) (cm^2^), using the Beer–Lambert relation (eq 4); *I*_0_ is the incident intensity of the white light from the deuterium lamp, *I* is the transmitted intensity after passing through the flow cell, *L* is the path length (cm), and *N* (molecules cm^–3^) is the gas-phase number density of absorbing species determines at the temperature of the flow cell (50 °C) using partial pressure calculations. Units for absorption cross section are reported in megabarns (Mb); 1 Mb = 10^–18^ cm^2^.
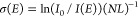
4Using the definition of absorbance (eq 5), the equation for the absorption cross-section is written in an alternative form (eq 6):
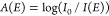
5
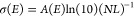
6The absorption spectra were then measured from 5.17 to 9.92 eV at a frequency of 91 Hz with resolution of ≤4 meV and were averaged at a frequency of 4.5 Hz for a total of 20 measurements to produce a single spectrum (each cross-section represents 20 separate data points). Given the range of photon energies and the energy resolution, a total of 2300 distinct cross sections measured per species. Measurements for each spectrum were repeated 3–5 times and a statistical average of absorption signal (eq 7) was produced using *n* values of a signal at a given photon energy, *x*(*E*). That signal showed no signs of spectral interference from contamination (e.g., oxygen or water), where 3 < *n* < 5.
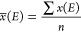
7Given the number of measurements used to produce the absorption cross sections, signal deviation was accounted for to quantify repeatability error using calculations of the standard deviation, *s*(*E*), standard error, ϵ(*E*), and the ratio of standard error-to-average, , for a given spectrum:
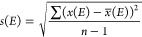
8
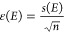
9
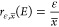
10Two main sources of experimental uncertainty were quantified: (1) gas-phase concentration within the absorption region and (2) experimental repeatability. Details and methods for calculating both types of uncertainty are outlined in Doner et al.^1^ All uncertainties were determined assuming errors as random variables with uniform distribution, which enables application of the root-sum-square (RSS) method to calculate an overall uncertainty for cross sections as a function of photon energy.

For spectral regions above a threshold of 2% of maximum absorbance, a standard-error-to-average ratio of <5% is typical.^1^ The standard-error-to-average ratio, , is used as a measure of repeatability, and determined for all species. Uncertainties in gas-phase concentration in the absorption cell were convolved with standard-error-to-average ratios for all species in order to report overall photon energy-specific uncertainties. The Supporting Information lists overall uncertainties for all 2300 cross sections measured for each of the 19 species, which are highest in regions where absorption is <2%.

## Results and Discussion

As mentioned previously, vertical excitation energies do not provide a direct comparison with any experimental observables. Using vertical excitation energies to produce the theoretical absorption spectra often results in the spectrum being either red- or blue-shifted, with the direction and extent of the shifting a result of the method or functional used. Past studies^11,14,15,18,21,23−30^ have determined that functionals with low amounts of HF-EX tend to underestimate the transition energy while functionals with higher amounts tend to overestimate. Similarly, the EOM-CCSD method nearly always overestimates the excitation energy.^10,13,14,26,43^ The vertical approximation also does not account for any vibrational contributions. There are vibrational methods that could be included to improve the accuracy of the spectra but these generally involve the addition of more expensive calculations, i.e., gradients and Hessians. However, using properties from the vertical approximation along with eq 1, the simulated spectra were determined to have relatively high accuracy compared to the high-resolution experimental data. It is then anticipated that the vertical approximation may provide a solid base for larger, more complex molecules in which more expensive computations are not feasible.

In order to measure each theoretical method’s accuracy, the simulated spectra were compared to experiment by both a qualitative analysis and four quantitative metrics including cosine similarity (*S*), relative integral change (RIC), mean signed error (MSE), and mean absolute error (MAE). When analyzing the spectra, two factors are considered: a) the overall shape of the spectrum and b) the alignment of the peaks. Specifically, alignment of the experimental and theoretical onset energies. If we are able to closely align these energies, it ensures that the theoretical spectra can be used for quantitative analysis as well as qualitative. To condense the results and summarize the main observations, a point system was used to determine accuracy across the 19 molecules. For each of the five metrics, the top three performing methods were awarded points; three points for the top performance, two for the second best, and one for the third. The points given are summed across the molecules for each method, and the total points are then used to determine which methods performed well. Due to similarities in spectra across methods, the quantitative analysis often concluded that spectra were equally accurate and so multiple spectra were awarded points. Data for the quantitative metrics and spectra for each of the molecules can be found in the Supporting Information.

### Hydrogen–Oxygen Molecules

**Figure 2:**
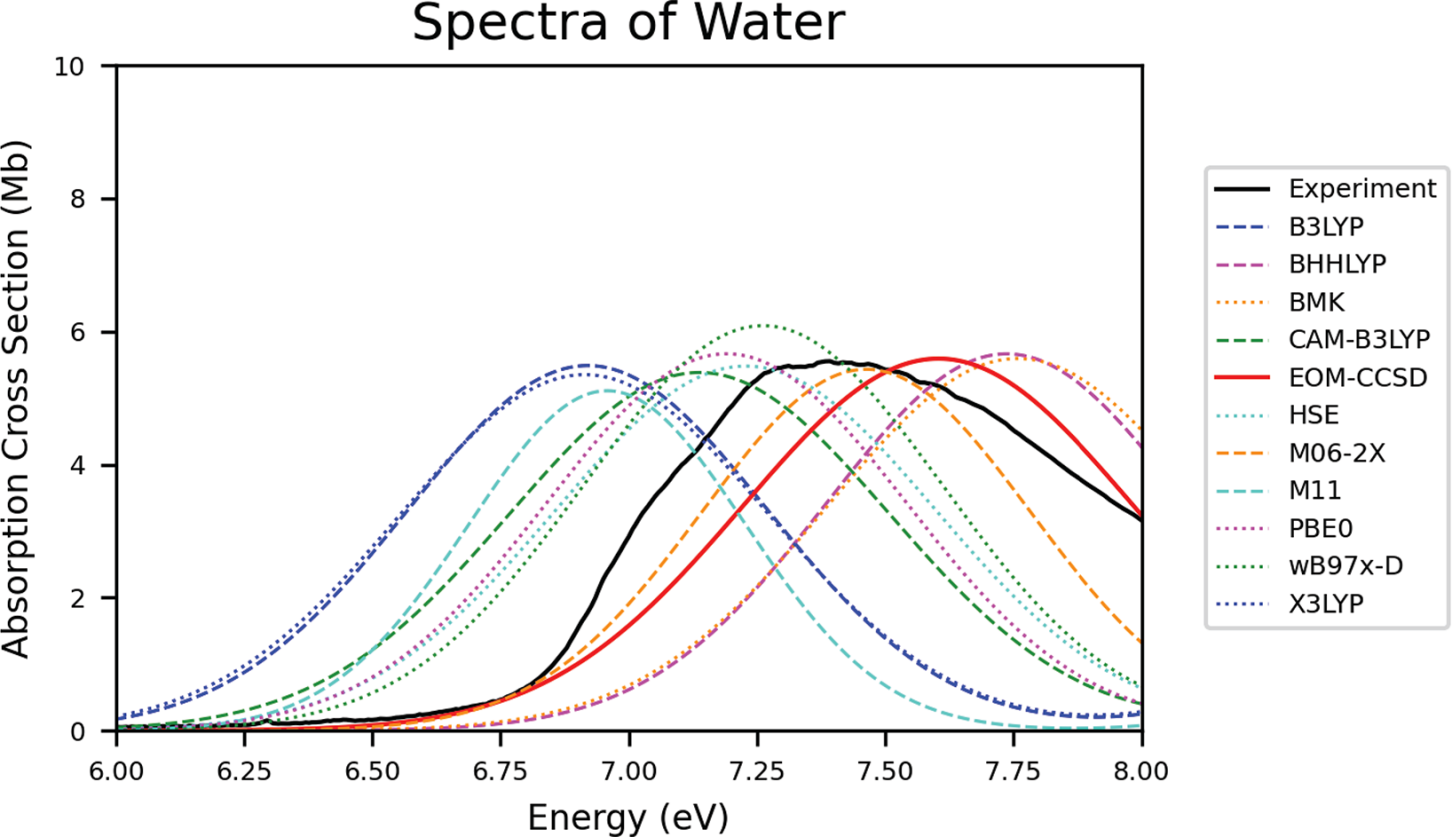
Spectra of water depicting the data collected from experiment, EOM-CCSD, and all TD-DFT functionals.

**Figure 3:**
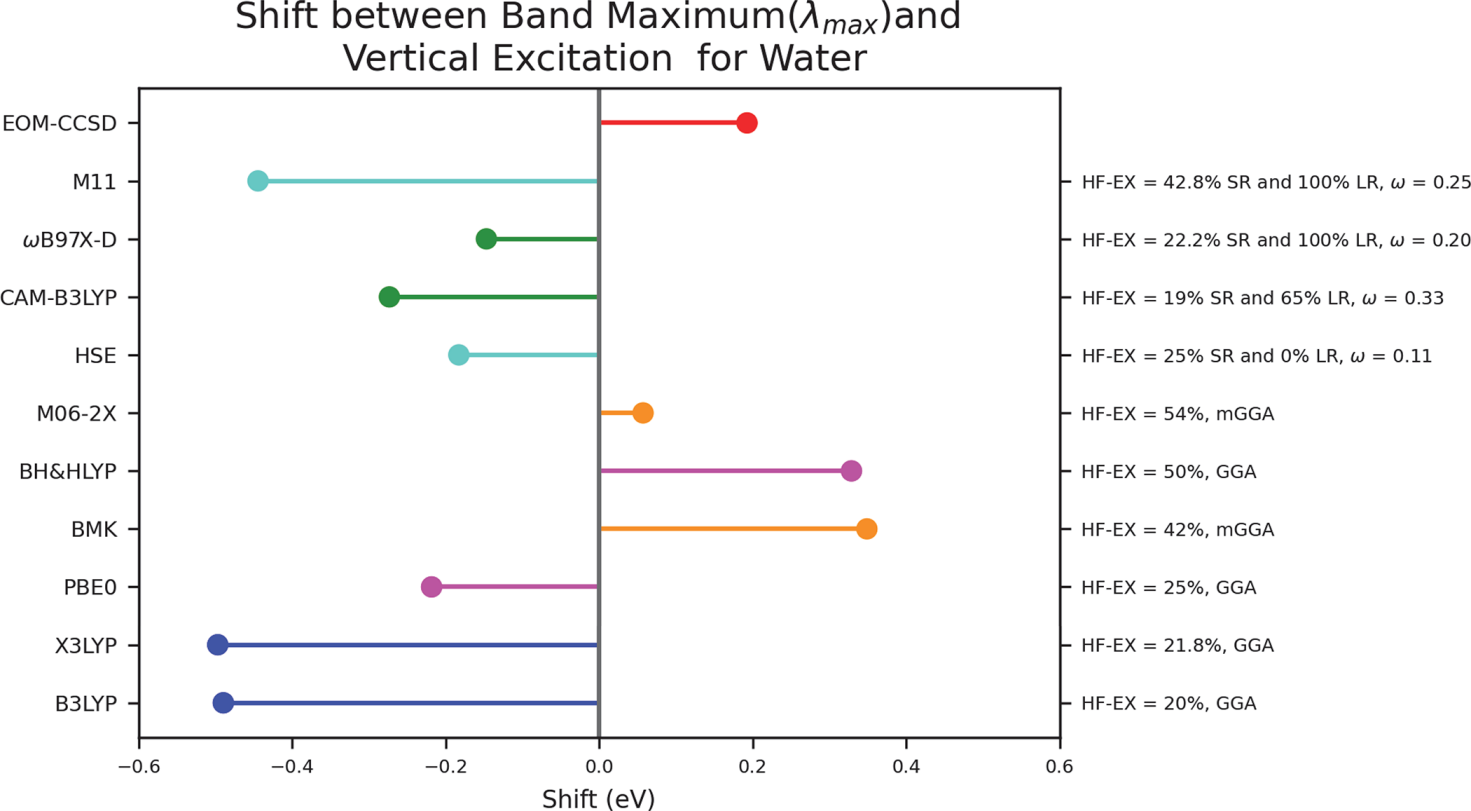
Difference between the experimental band maximum (λ_max_) and the computed vertical excitation energy of the various methods for water.

Figure 2 shows the experimental and theoretical spectra for water. The experimental absorption spectra displays a single broad peak, with the maximum absorbance at approximately 7.40 eV.^95^ All of the methods tested produced spectra with similar shapes; the only difference being slight variations in the peak location. Table 1 reports the S, RIC, MSE, and MAE values as well as the total points awarded. One can easily see that the M06-2X functional was the top performing method. It produced the best spectrum with the maximum absorbance band lining up almost exactly with the experimental one, and the quantitative metrics attest to its accuracy. The EOM-CCSD, HSE, and ωB97X-D spectra are also fairly accurate, being only slightly shifted. Many of the TD-DFT functionals show the spectrum to be slightly red-shifted, with B3LYP, M11, and X3LYP the most significantly red-shifted. The red-shifting observed here is expected for B3LYP and X3LYP as they are global hybrid GGAs with low HF-EX, 20% and 21.8% respectively. Interestingly, the significant red-shifting shown by M11 is unexpected. M11 being a range separated mGGA with 42.8% short-range HF-EX and 100% long-range HF-EX, one would anticipate its spectrum to be fairly reliable, if slightly blue-shifted. CAM-B3LYP and PBE0 also showed some red-shifting, though not as extreme as the previously mentioned methods. A few of the functionals, namely, BH&HLYP and BMK, are slightly blue-shifted. Again, this shift is in agreement with predictions based on the amount of HF-EX in each functional, 50% and 42%, respectively. The effects of HF-EX on shifting are depicted in Figure 3.

**Table 1:** Average Cosine Similarity (*S*), Relative Integral Change (RIC), Mean Signed Error (MSE), Mean Absolute Error (MAE), and Total Points for Water, with All Quantitative Metrics in Mb

	*S*	RIC	MSE	MAE	Points
B3LYP	0.825	0.874	1.887	2.283	0
BH&HLYP	0.978	0.751	–1.973	1.973	0
BMK	0.983	0.695	–1.898	1.898	1
CAM-B3LYP	0.932	0.675	1.671	1.676	0
HSE	0.958	0.547	1.609	1.609	1
M06-2X	0.997	0.248	–0.601	0.601	15
M11	0.829	0.799	1.752	2.332	0
PBE0	0.953	0.634	1.795	1.795	0
ωB97X-D	0.964	0.513	1.472	1.472	4
X3LYP	0.851	0.736	2.058	2.435	0
EOM-CCSD	0.996	0.377	–1.023	1.023	10

### Alkanes

**Figure 4:**
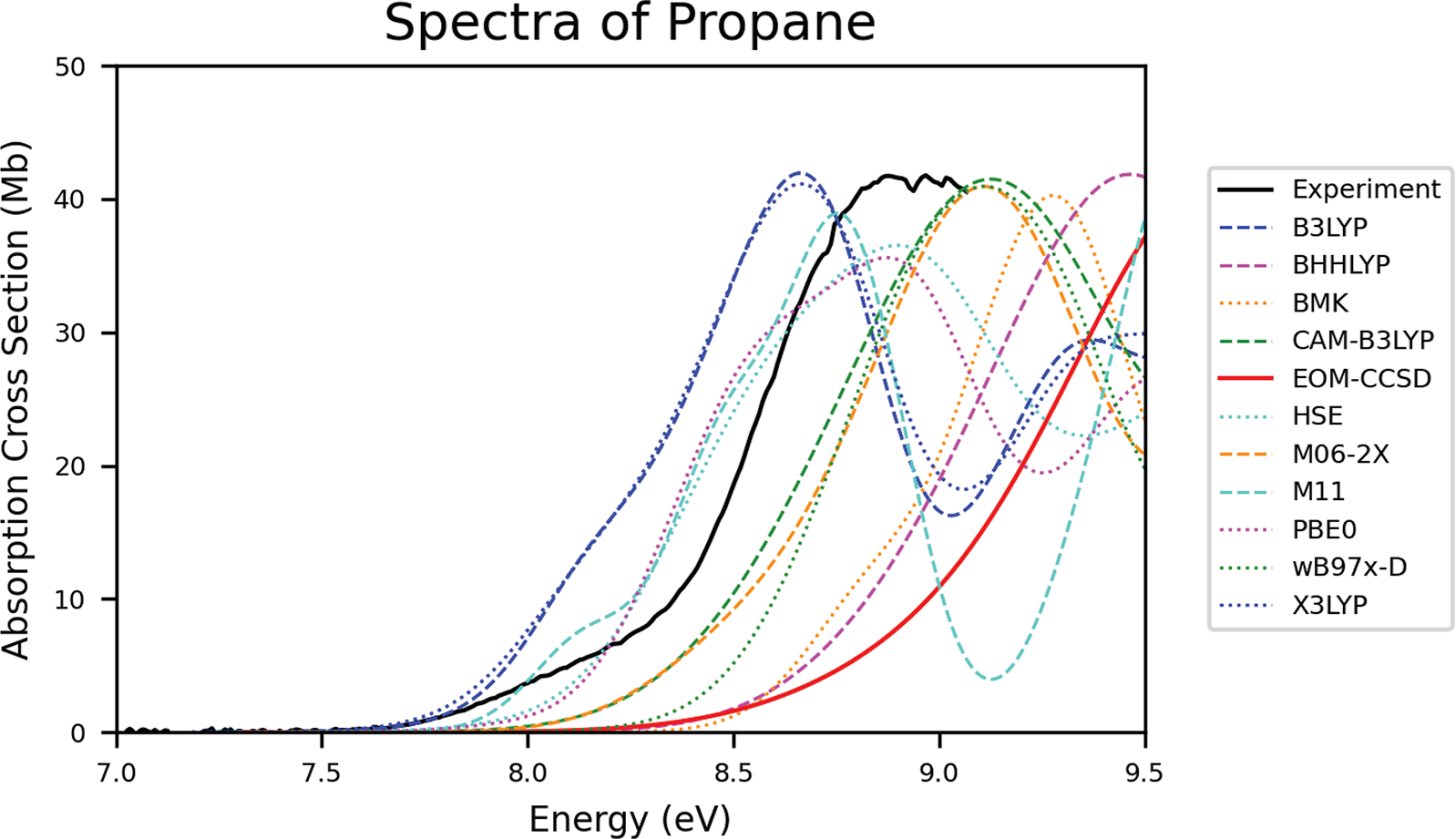
Spectra of propane depicting the data collected from experiment, EOM-CCSD, and all TD-DFT functionals.

Table 2 reports the average *S*, RIC, MSE, and MAE values for ethane, propane, and *n*-butane. It also reports the total points awarded for each method. Similar to the water molecule, there is one method that performs the best in all of the considered metrics, CAM-B3LYP. Also in agreement with the trends seen in water, there is a systematic shifting in the functionals that corresponds to the amount of HF-EX. The red-shifting observed in the B3LYP and X3LYP spectra is expected due to the low amounts of HF-EX in the functionals. Similarly, the blue-shifting of BH&HLYP, BMK, and EOM-CCSD was predicted. CAM-B3LYP is the top performing method for the alkanes. HSE, M06-2X, and ωB97X-D also perform well. CAM-B3LYP, HSE, and ωB97X-D are all range-separated hybrid functionals. In these functionals, it seems the percentage of long-range HF-EX can explain the slight shifting seen. CAM-B3LYP, which has 65% long-range HF-EX, produced maximum absorption bands that most closely match the band maxima. HSE, which has 0% long-range HF-EX, often aligns well, but still tends to shift slightly to lower energy. ωB97X-D, which has 100% long-range HF-EX, also aligns well but tends to shift to slightly higher energies. Based on the amount of HF-EX (54%), one would expect M06-2X to produce reliable spectra, and this prediction is confirmed by the accuracy of the alkane spectra. Figure 4 shows the experimental and theoretical spectra for propane. Here again, many of the methods tested are able to produce the shape of the spectrum but with slight shifting.

**Table 2:** Average Cosine Similarity (*S*), Relative Integral Change (RIC), Mean Signed Error (MSE), Mean Absolute Error (MAE), and Total Points for Ethane, Propane, and *n*-Butane, with All Quantitative Metrics in Mb

	*S*	RIC	MSE	MAE	Points
B3LYP	0.971	1.702	11.347	11.598	3
BH&HLYP	0.924	0.871	–8.873	8.873	0
BMK	0.876	0.858	–8.595	8.595	0
CAM-B3LYP	0.988	0.296	–1.958	2.603	27
HSE	0.984	0.552	2.776	3.834	21
M06-2X	0.987	0.350	–2.231	3.235	15
M11	0.956	0.781	4.846	6.406	7
PBE0	0.978	0.823	4.175	5.526	5
ωB97X-D	0.968	0.402	–3.796	3.803	17
X3LYP	0.967	1.810	12.084	12.233	2
EOM-CCSD	0.932	0.903	–9.143	9.143	0

### Alkenes

In contrast to the clear front-runner in the water and alkane analysis, Table 3 shows that there is more disparity in top-performing method for alkenes. M06-2X, CAM-B3LYP, and ωB97X-D are all the most accurate in one or more of the quantitative metrics. There are also more discrepancies in the shifting observed than in the previous sections. For example, BH&HLYP and BMK, which show non-negligible blue-shifting for many of the spectra, align well for ethene and are only slightly shifted for propene, *cis*-butene, and *trans*-butene. There are still similarities to the water and alkane analyses. In particular, EOM-CCSD is significantly blue-shifted in all of the spectra, and B3LYP, M11, and X3LYP are red-shifted.

**Table 3:** Average Cosine Similarity (*S*), Relative Integral Change (RIC), Mean Signed Error (MSE), Mean Absolute Error (MAE), and Total Points for Ethene, Propene, *cis*-Butene, and *trans*-Butene, with All Quantitative Metrics in Mb

	*S*	RIC	MSE	MAE	Points
B3LYP	0.981	0.466	7.729	8.725	1
BH&HLYP	0.990	0.188	–1.113	3.912	6
BMK	0.988	0.217	–3.731	4.912	8
CAM-B3LYP	0.991	0.123	1.312	2.956	25
HSE	0.986	0.259	3.282	4.954	15
M06-2X	0.990	0.116	–0.473	2.822	28
M11	0.990	0.242	3.106	4.640	9
PBE0	0.987	0.268	3.247	4.972	22
ωB97X-D	0.990	0.133	–0.455	3.138	21
X3LYP	0.982	0.469	7.925	8.509	2
EOM-CCSD	0.980	0.669	–14.299	14.309	0

**Figure 5:**
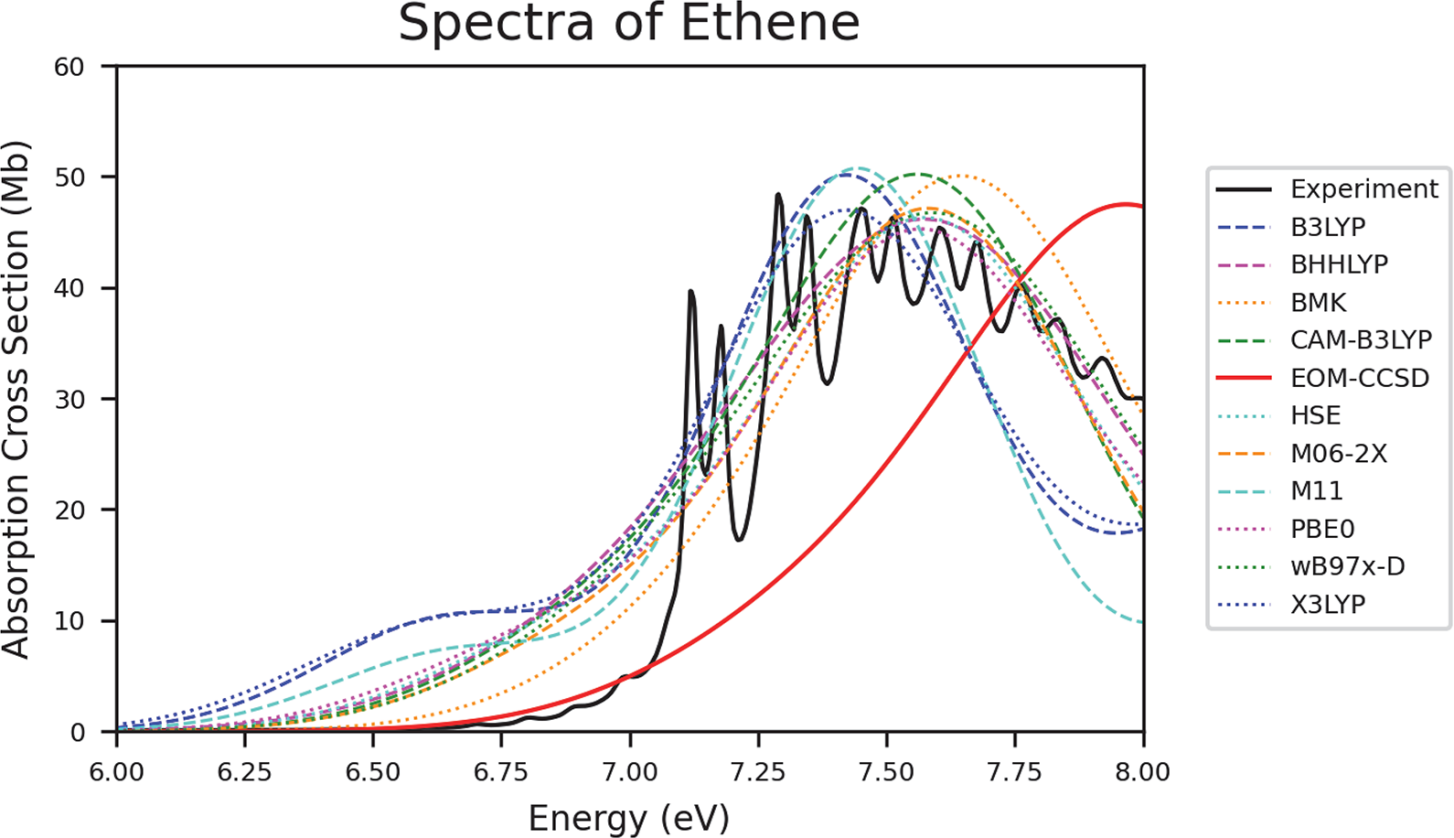
Spectra of ethene depicting the data collected from experiment, EOM-CCSD, and all TD-DFT functionals.

We mentioned previously that while inclusion of vibrational contributions would undoubtedly improve the accuracy of the predicted spectra, we purposefully exclude them here to analyze the accuracy of the simplest approximation to recreating one-photon absorption spectra. The spectra of ethene, Figure 5, are good examples of this. Though the theoretical spectra do not show the vibrational resolution, the overall shape of the spectra are reasonably accurate.

### Aldehydes

For acetaldehyde, again we see one predominant method, M06-2X. It is worth noting here that even the top performing method for acetaldehyde is not as accurate as the spectra seen in the previous sections. This is especially clear when looking at the relatively low *S* value, Table 4. However, when looking at the spectrum (Figure S9) one can see that this low *S* value is more a result of the Gaussian fitting than the computed excitation energies. The peaks for the M06-2X spectrum align reasonably well, but the slopes of the onset energies vary. The acetaldehyde spectra also show more drastic shifting than many of the other molecules. This can be seen in the significant red-shifting of the B3LYP, HSE, M11, PBE0, and X3LYP. Specifically HSE and PBE0 methods, which perform well in other scenarios. The spectra for CAM-B3LYP and ωB97X-D, other methods that typically perform well, are also slightly shifted. Since acetaldehyde was the only aldehyde considered in this benchmarking set, it is difficult to tell if these trends would be consistent across other aldehydes.

**Table 4:** Average Cosine Similarity (*S*), Relative Integral Change (RIC), Mean Signed Error (MSE), Mean Absolute Error (MAE), and Total Points for Acetaldehyde, with All Quantitative Metrics in Mb

	*S*	RIC	MSE	MAE	Points
B3LYP	0.548	1.233	1.826	6.353	0
BH&HLYP	0.527	0.858	–2.429	4.468	4
BMK	0.390	1.005	–2.402	5.339	2
CAM-B3LYP	0.464	1.188	–0.856	5.812	3
HSE	0.664	2.088	9.915	10.974	3
M06-2X	0.663	0.708	–2.039	3.523	11
M11	0.542	2.333	8.801	12.187	0
PBE0	0.657	2.176	10.557	11.697	1
ωB97X-D	0.527	1.072	–0.695	5.421	4
X3LYP	0.507	1.533	4.075	8.305	0
EOM-CCSD	0.527	0.919	–1.194	4.597	3

Similar to the trend observed in the alkanes, BH&HLYP, BMK, and EOM-CCSD are blue-shifted. The M06-2X spectrum produced the most accurate spectrum. The EOM-CCSD, CAM-B3LYP, and ωB97X-D spectra are comparable to the experimental spectrum, but are less reliable due to the shifting noted earlier.

### Ketones

**Figure 6:**
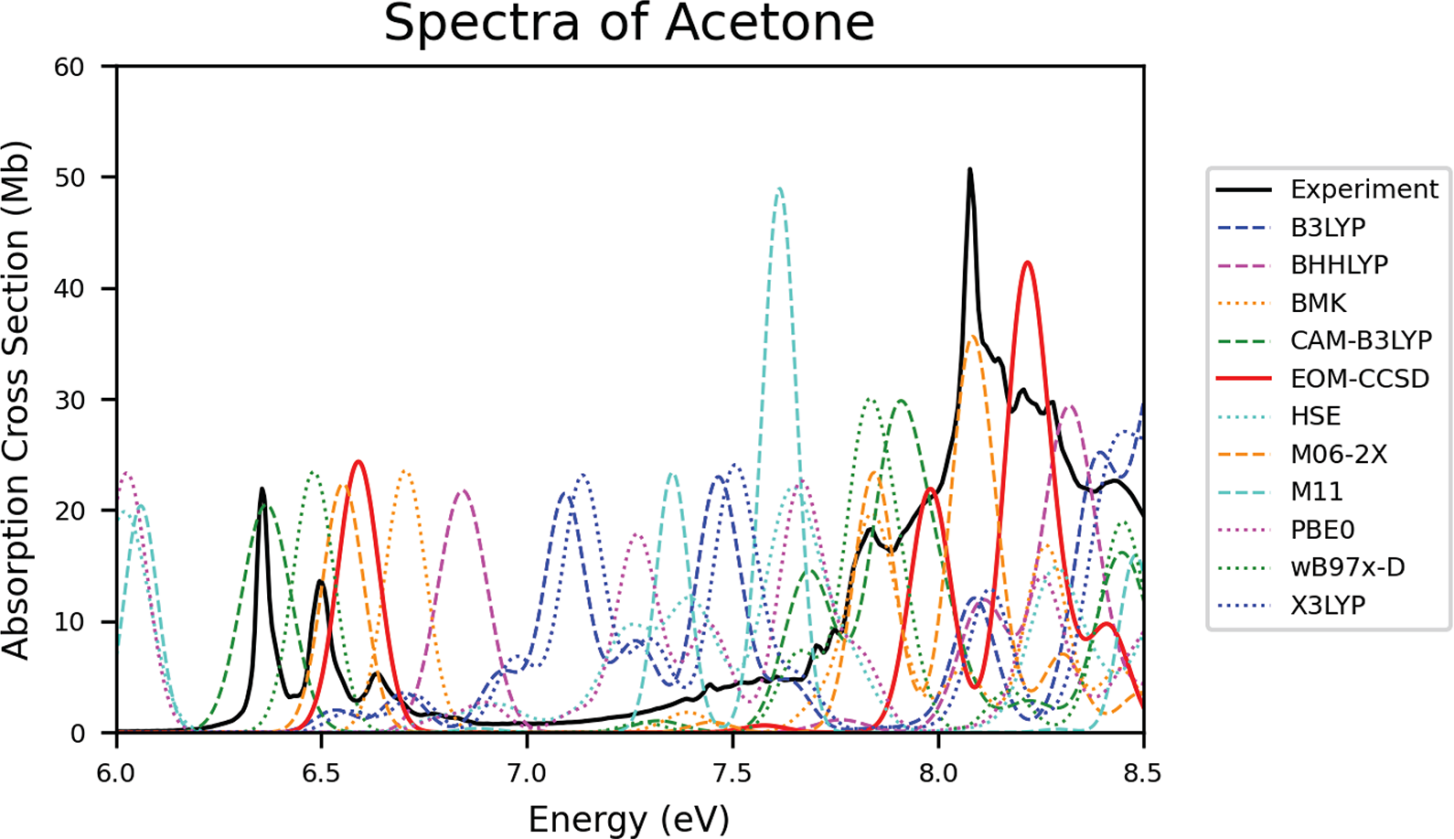
Spectra of acetone depicting the data collected from experiment, EOM-CCSD, and all TD-DFT functionals.

Table 5 reports the quantitative metrics and total points for acetone and methyl vinyl ketone. Similar to the results for acetaldehyde, the quantitative values for acetone and methyl vinyl ketone are worse than for water, alkanes, and alkenes. For acetone, the error seems to stem from a similar reasoning as acetaldehyde, the Gaussian fit is not able to align with the steep onset energies of the experimental data and there is significant shifting observed. Figure 6 shows the extensive shifting by most, if not all, of the methods tested. The shifting observed in the acetone spectra goes against expectations and cannot be explained by the amount of HF-EX as it is in previously mentioned molecules. For example, the B3LYP and X3LYP spectra are blue-shifted for acetone. The spectra for methyl vinyl ketone (Figure S11) also show shifting, though not as drastic as acetone.

**Table 5:** Average Cosine Similarity (*S*), Relative Integral Change (RIC), Mean Signed Error (MSE), Mean Absolute Error (MAE), and Total Points for Acetone and Methyl Vinyl Ketone, with All Quantitative Metrics in Mb

	*S*	RIC	MSE	MAE	Points
B3LYP	0.640	0.794	3.434	10.451	6
BH&HLYP	0.537	0.623	–0.053	5.353	14
BMK	0.593	0.934	3.443	7.973	7
CAM-B3LYP	0.825	0.792	5.485	8.137	4
HSE	0.508	0.830	3.687	9.743	1
M06-2X	0.734	0.740	4.197	7.173	9
M11	0.514	0.680	1.277	6.760	5
PBE0	0.502	831	3.832	9.673	1
ωB97X-D	0.788	0.788	5.294	7.924	3
X3LYP	0.606	0.794	3.444	10.143	4
EOM-CCSD	0.689	0.863	–3.820	7.266	10

In previous molecular classes, we often saw consistencies in method accuracy. For the ketones, however, methods performed differently for acetone and methyl vinyl ketone. The most accurate methods for acetone were M06-2X, CAM-B3LYP, and ωB97X-D, which match previous sections, despite the unusual shifting seen. For methyl vinyl ketone, the top performing methods were BH&HLYP and EOM-CCSD, which have, in previous sections, been the most consistently shifted.

### Carboxylic Acids

**Figure 7:**
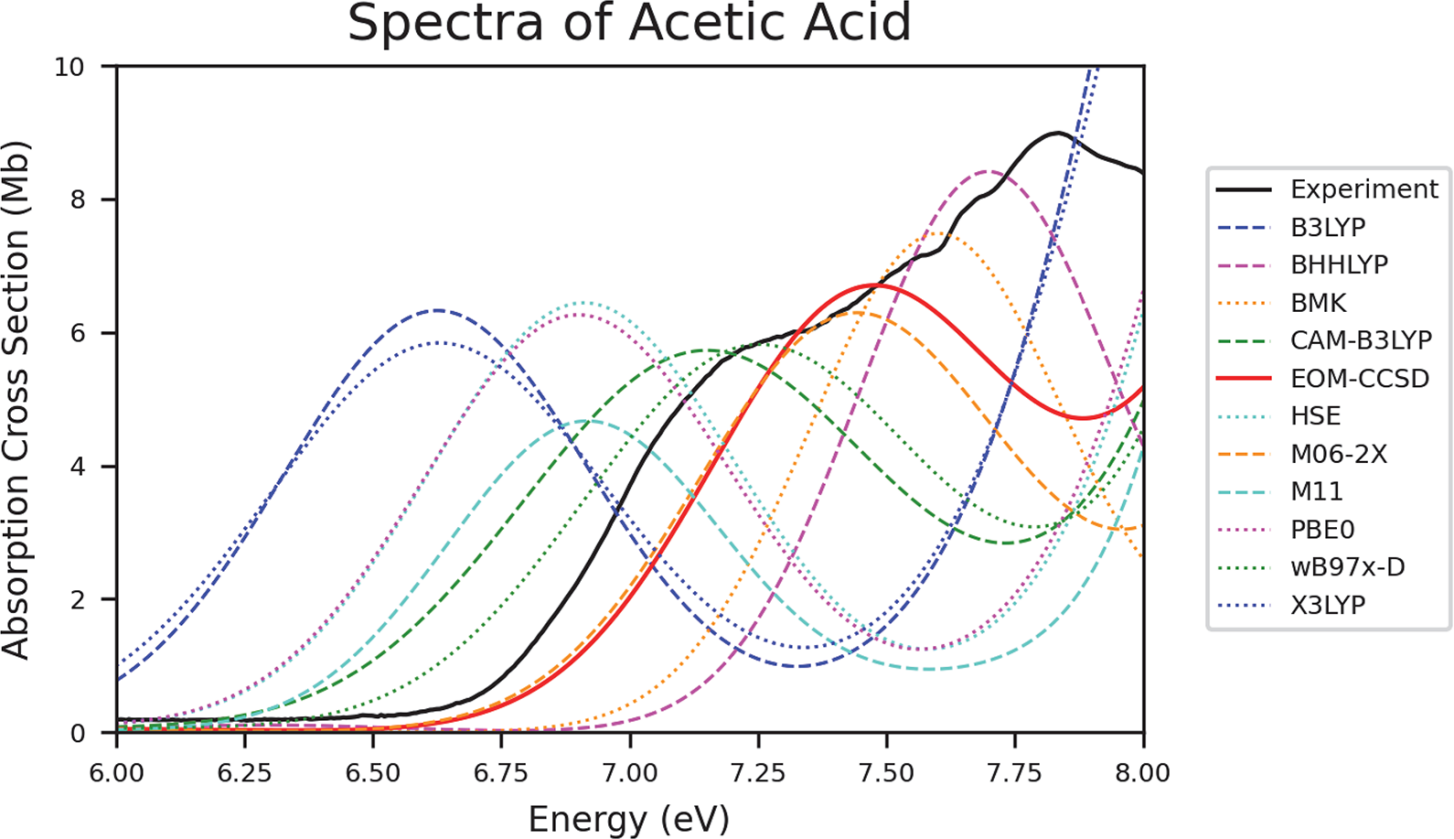
Spectra of acetic acid depicting the data collected from experiment, EOM-CCSD, and all TD-DFT functionals.

The results for carboxylic acids, Table 6, are more in line with those of water, alkanes, and alkenes. M06-2X is again the top performing method in all of the metrics considered. ωB97X-D also performs well for all of the carboxylic acids. Figure 7 shows the experimental and theoretical spectra for acetic acid. Again, many of the methods are able to mimic the shape of the experimental spectrum, but are shifted. Interestingly, the spectra for propionic acid (Figure S14) are split, with B3LYP, M11, PBE0, and X3LYP mimicking the shape more accurately while CAM-B3LYP, M06-2X, and ωB97X-D are not able to resolve the shape but align with the onset energy very closely. For the sake of the current analysis, the alignment of the onset energy is considered to be more accurate, and so those methods are found to be the top performers.

**Table 6:** Average Cosine Similarity (*S*), Relative Integral Change (RIC), Mean Signed Error (MSE), Mean Absolute Error (MAE), and Total Points for Formic Acid, Acetic Acid, and Propionic Acid, with All Quantitative Metrics in Mb

	*S*	RIC	MSE	MAE	Points
B3LYP	0.842	1.388	2.263	3.227	3
BH&HLYP	0.948	0.505	–1.449	1.487	10
BMK	0.960	0.394	–1.137	1.208	11
CAM-B3LYP	0.976	0.561	1.261	1.271	7
HSE	0.925	1.105	2.144	2.345	0
M06-2X	0.993	0.267	–0.069	0.691	25
M11	0.925	0.517	0.879	1.383	3
PBE0	0.924	1.032	2.232	2.486	3
ωB97X-D	0.989	0.371	0.767	0.828	22
X3LYP	0.858	1.329	2.189	3.108	0
EOM-CCSD	0.985	0.485	–0.474	1.124	9

The carboxylic acids follow trends similar to the other molecule types analyzed. B3LYP, HSE, M11, PBE0, and X3LYP are consistently red-shifted while BH&HLYP, BMK, EOM-CCSD are consistently blue-shifted. The top performing methods are M06-2X and ωB97X-D. BH&HLYP and BMK also performed well for carboxylic acids. The carboxylic acids group seems to have the most functionals that are consistently shifted.

### Alcohols

**Figure 8:**
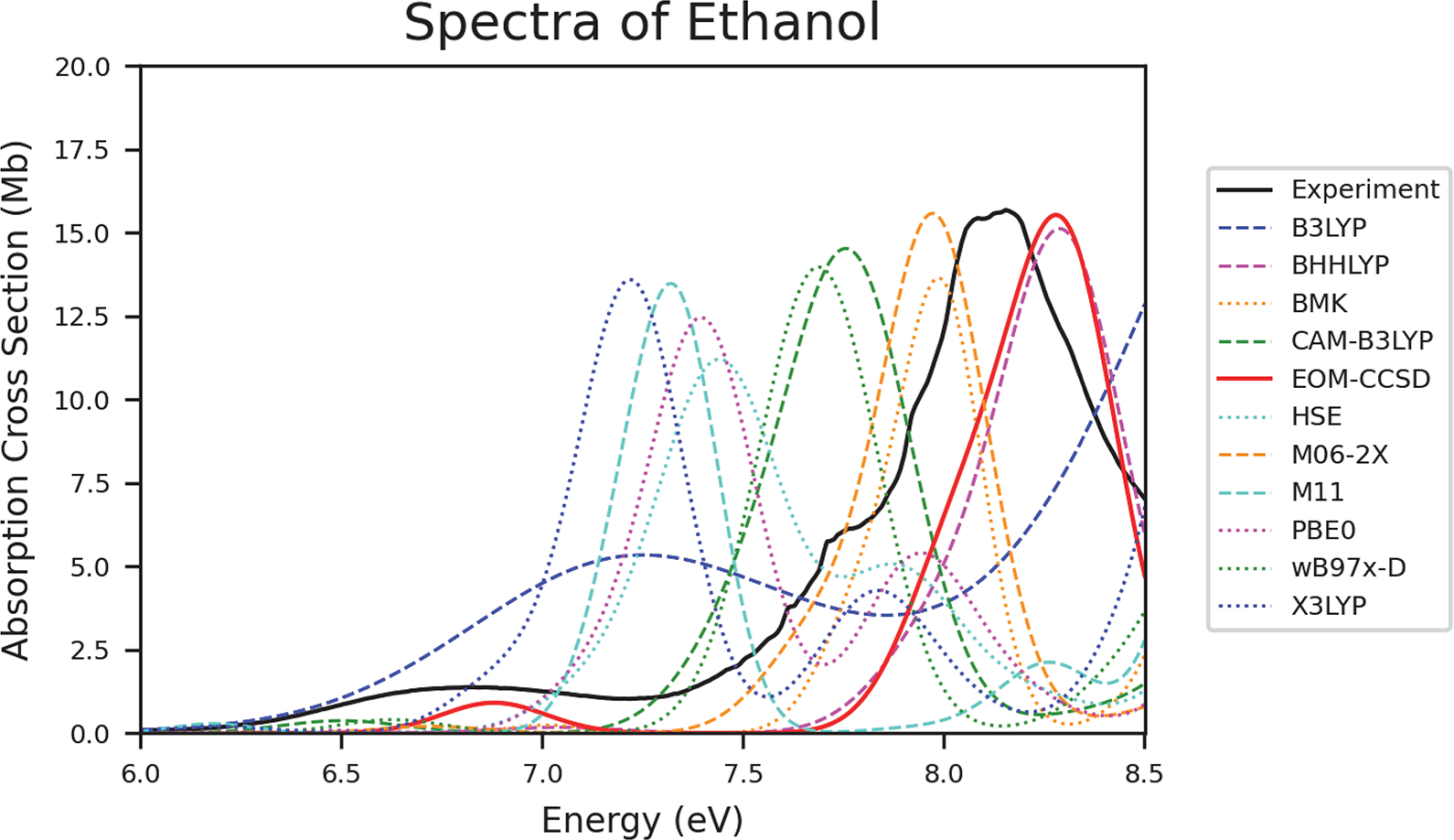
Spectra of ethanol depicting the data collected from experiment, EOM-CCSD, and all TD-DFT functionals.

Table 7 shows that BMK and M06-2X are the two top performing methods for alcohols. While M06-2X has performed well for most of the molecules in this study, BMK has often been fairly shifted, and so its high accuracy is surprising. Similar to some of the previous molecule groups, B3LYP HSE, M11, PBE0, and X3LYP are significantly and consistently red-shifted for all of the alcohols analyzed. This shifting can be seen in Figure 8. In contrast, BMK and EOM-CCSD, which are often significantly blue-shifted, are two of the top performing methods for alcohols, and M06-2X is the only top performing method in agreement with other molecule groups. CAM-B3LYP and ωB97X-D, which have been performing well in all of the previous molecule groups, are outperformed for alcohols.

**Table 7:** Average Cosine Similarity (*S*), Relative Integral Change (RIC), Mean Signed Error (MSE), Mean Absolute Error (MAE), and Total Points for Methanol, Ethanol, 1-Propanol, and 2-Propanol, with All Quantitative Metrics in Mb

	*S*	RIC	MSE	MAE	Points
B3LYP	0.721	1.181	1.641	3.740	3
BH&HLYP	0.910	0.724	–2.926	2.926	7
BMK	0.962	0.405	–0.109	1.441	44
CAM-B3LYP	0.807	1.346	3.845	4.718	0
HSE	0.767	1.215	3.093	4.206	3
M06-2X	0.973	0.424	0.744	1.449	44
M11	0.480	1.450	0.792	5.175	0
PBE0	0.751	1.201	2.488	4.081	0
ωB97X-D	0.806	1.140	2.846	4.208	1
X3LYP	0.732	1.241	1.391	3.791	1
EOM-CCSD	0.925	0.691	–2.619	2.622	18

### Ethers

Table 8 reports the values for quantitative metrics and total points for dimethyl ether. Most of the methods are able to produce the correct spectral shape, but there is shifting of the band maximum. As expected, B3LYP, HSE, M11, PBE0, and X3LYP are red-shifted, while BH&HLYP, BMK, and EOM-CCSD are blue-shifted. For the ether, CAM-B3LYP and ωB97X-D are also red-shifted. M06-2X produced the most accurate spectrum, aligning with experiment quite well. Interestingly, the significant red-shifting observed in the B3LYP spectrum results in it performing deceptively well. The theoretical spectrum is red-shifted by approximately 0.75 eV, which results in the higher energy peak aligning with the onset energy of the experimental spectrum. Similar shifting is observed in the X3LYP spectrum. Thus, in order to not include the false positives of B3LYP and X3LYP in the final results, the points earned by B3LYP and X3LYP for dimethyl ether are removed from the overall total points used for ranking.

**Table 8:** Average Cosine Similarity (*S*), Relative Integral Change (RIC), Mean Signed Error (MSE), Mean Absolute Error (MAE), and Total Points for Dimethyl Ether, with All Quantitative Metrics in Mb

	*S*	RIC	MSE	MAE	Points
B3LYP^a^	0.992	0.127	0.223	0.570	11
BHI&HLYP	0.752	0.831	1.076	3.649	0
BMK	0.917	0.451	0.790	2.004	0
CAM-B3LYP	0.752	0.650	–0.843	2.848	0
HSE	0.824	0.523	–0.402	2.329	0
M06-2X	0.980	0.194	–0.113	0.837	12
M11	0.957	0.295	0.278	1.289	0
PBE0	0.863	0.456	–0.255	2.013	1
ωB97X-D	0.830	0.548	–0.710	2.392	1
X3LYP^a^	0.977	0.253	0.571	1.107	3
EOM-CCSD	0.909	0.385	–0.661	1.682	2

a Wrong peak aligned.

### Complete Set

The overall accuracy of each method is reported in Table 9 which shows the average *S*, RIC, MSE, and MAE for the 19 molecules, as well as the total points earned. Though there were some individual cases in which the methods seemed to perform poorly, when considering the entire set one can see that method evaluated here does produce theoretical spectra with reasonable accuracy. Based on the data presented in Table 9, it is clear that M06-2X out-performs the other methods. It is one of the top performing methods in each of the four metrics and its total points are nearly double that of the second best performing method. M06-2X is the only method to have an average *S* value greater than 0.9, an average RIC value less than 0.5, and an average MAE less than 4.0. The second and third best performing methods are BMK and ωB97X-D, with 79 and 71 points, respectively. BMK has the second lowest RIC value. ωB97X-D has the lowest MSE value. CAM-B3LYP, which is almost tied with ωB97X-D, produced the third best *S*, MSE, and MAE values. The third best performing method is ωB97X-D, with 70 total points. ωB97X-D performed the best in MSE value and the third best in RIC and MAE values. EOM-CCSD produced the second highest *S* value, but was ranked sixth according to overall points. BH&HLYP also performed well in the overall quantitative metrics, second lowest MAE and third lowest RIC values, but was ranked seventh.

**Table 9:** Average Cosine Similarity (*S*), Relative Integral Change (RIC), Mean Signed Error (MSE), Mean Absolute Error (MAE), and Total Points for All 19 Molecules, with All Quantitative Metrics in Mb

	*S*	RIC	MSE	MAE	Points
B3LYP	0.825	1.106	5.307	7.434	19
BH&HLYP	0.837	0.643	–3.106	4.274	48
BMK	0.860	0.607	–2.116	4.850	79
CAM-B3LYP	0.863	0.685	1.610	4.409	74
HSE	0.842	0.851	3.490	5.288	58
M06-2X	0.923	0.431	1.081	3.693	163
M11	0.779	0.896	3.501	6.017	28
PBE0	0.833	0.883	3.348	5.690	39
ωB97X-D	0.862	0.664	1.024	4.590	74
X3LYP	0.822	1.148	5.735	7.827	12
EOM-CCSD	0.890	0.708	–5.682	6.389	60

The data shown in Table 9 can also give insight into systematic errors in the methods. Because vertical excitations are being used, it is expected that the theoretical spectra are shifted. Overestimating the vertical excitation energy would lead to a blue-shifted spectrum and a negative MSE value. This can be seen clearly in the results for BH&HLYP, BMK, and EOM-CCSD. Many of the other methods, on average, underestimate the transition energy. This is especially true for B3LYP and X3LYP, who have the largest positive MSE. Again, the analysis of individual molecules and molecule type groups also showed this trend. B3LYP, HSE, M11, PBE0, and X3LYP were consistently red-shifted. The relatively low MSE and MAE values of M06-2X, CAM-B3LYP, and ωB97X-D confirm that they most often aligned with the experimental spectra.

### QUEST Database Comparisons

In addition to a thorough comparison of the simulated spectra to the high-resolution experimental spectrum, the vertical excitation energies are compared to those within the QUEST database. The QUEST database was created by Loos, Jacquemin, and others to provide highly accurate and reliable vertical transition energies, which may be used to help compare and develop various excited-state methods. Within this database, a set of theoretical best estimates (TBEs) using the aug-cc-pVTZ basis set are defined. TBEs allow a more straightforward comparison of the excitation energy for theoretical methods since they do not suffer from the various environmental factors influencing experimental spectra. In this section, we present the mean signed error (MSE) and mean average error (MAE) of the selected methods with respect to the TBE/aug-cc-pVTZ data of Loos, Jacquemin, and co-workers. Further comparisons are contained in the SI.

The QUEST database only contains water, ethylene, acetaldehyde, and acetone out of the set of molecules within this study. In Tables 10 and 11, the differences between the QUEST database TBE/aug-cc-pVTZ and our computed transition energy for water and acetone are presented. It is plain that EOM-CCSD matches the most consistently for all transitions of the methods used in this work with a MSE and MAE of 0.08 for water and a MSE of −0.05 and MAE of 0.06 for acetone. The best performing TD-DFT methods differ between the molecules. BMK occurs as one of the top three functionals in all four molecules with MAEs of 0.29, 0.30, 0.01, and 0.16 for water, ethylene, acetaldehyde, and acetone, respectively. For water, ethylene, and acetone, the B3LYP and X3LYP have the highest errors but for acetaldehyde they provide a lowest singlet transition energy matching better than 0.01 eV.

**Table 10:** Difference for the First Three Lowest Singlet Transitions of Water between the TBE/aug-cc-pVTZ Taken from the QUEST Database and the Methods Utilized in This Work, Which Used the d-aug-cc-pVTZ Basis Set^a^

method	^1^*B*_1_	^1^*A*_2_	^1^*A*_1_	MSE	MAE
B3LYP	0.72	1.13	1.23	1.03	1.03
BH&HLYP	–0.10	0.13	0.20	0.08	0.14
BMK	–0.12	0.30	0.44	0.21	0.29
CAM-B3LYP	0.50	0.74	0.86	0.70	0.70
HSE	0.41	0.79	0.91	0.70	0.70
M06-2X	0.17	0.50	0.49	0.39	0.39
M11	0.67	1.28	1.13	1.03	1.03
PBE0	0.45	0.87	1.01	0.78	0.78
ωB97X-D	0.37	0.79	0.96	0.71	0.71
X3LYP	0.72	1.10	1.20	1.01	1.01
EOM-CCSD	0.04	0.07	0.12	0.08	0.08

a All values are in eV.

**Table 11:** Difference for the First Five Lowest Singlet Transitions of Acetone between the TBE/aug-cc-pVTZ Taken from the QUEST Database and the Methods Utilized in This Work, Which Used the d-aug-cc-pVTZ Basis Set^a^

method	^1^*A*_2_	^1^*B*_2_	^1^*A*_2_	^1^*A*_1_	^1^*B*_2_	MSE	MAE
B3LYP	0.04	0.70	0.84	0.98	0.92	0.70	0.70
BH&HLYP	–0.31	–0.38	–0.25	–0.20	–0.15	–0.26	0.26
BMK	0.02	–0.24	0.13	0.17	0.22	0.06	0.16
CAM-B3LYP	–0.05	0.09	0.20	0.22	0.30	0.15	0.17
HSE	–0.01	0.44	0.59	0.72	0.69	0.49	0.49
M06-2X	0.19	–0.09	0.12	0.07	0.17	0.09	0.13
M11	0.28	0.40	0.81	0.59	0.75	0.57	0.57
PBE0	–0.01	0.43	0.62	0.73	0.72	0.50	0.50
ωB97X-D	–0.06	–0.02	0.27	0.22	0.35	0.15	0.18
X3LYP	0.03	0.70	0.81	0.94	0.89	0.67	0.67
EOM-CCSD	–0.03	–0.13	–0.03	–0.09	0.04	–0.05	0.06

a All values are in eV.

With the time-dependent density functionals, the ones that best reproduce the TBE/aug-cc-pVTZ have some deviations from the ones that were determined to be choice for simulating the experimental spectra. In Table 10, the transition energies for water are best replicated by the density functional BH&HLYP with BMK and M06-2X following, while in Table 11, acetone is best represented by M06-2X, BMK, and then CAM-B3LYP. Ethylene also slightly differs from water and acetone, with the choice functionals being BMK, ωB97X-D, and BH&HLYP. As noted earlier, acetaldehyde deviates strongly from all prior results with B3LYP, X3LYP, and then BMK duplicating the TBE most closely. Water, ethylene, and acetone more closely reflect the results obtained comparing to experimental data, with the exception of the good performance of BH&HLYP for computing vertical transition energies. In comparing the final results of the ranking in Table 9 to the QUEST comparisons, the ability to accurately compute the excitation energy does not directly correlate to producing a spectrum that matches experiment.

## Conclusion

Given the sheer number of available functionals, methods, and basis sets to choose from, it is difficult to know which method to use. This study analyzed 11 methods, 10 TD-DFT functionals and EOM-CCSD to determine their ability to produce accurate VUV spectra for a set of 19 small molecules relevant to combustion and atmospheric chemistry. Four quantitative metrics are used along with a qualitative analysis of the simulated spectra to determine which methods perform well. When looking at the results for individual molecules, it can be difficult to determine methods that are outperforming or underperforming, due to the similarities in the spectra. However, certain trends were uncovered, specifically regarding the shifts different methods experienced. As expected, the EOM-CCSD spectra were consistently blue-shifted. The BH&HLYP and BMK spectra were also often blue-shifted. In contrast, B3LYP, HSE, M11, PBE0, and X3LYP were almost always red-shifted. When looking at the average metrics across the 19 molecules, it is evident that M06-2X outperforms the other methods. It produced accurate spectra, performed well in the metric comparisons, and did so consistently across the set of molecules. BMK, CAM-B3LYP, and ωB97X-D were also able to produce reliable spectra. This study is one of few^39,41,42,45,46^ that compare spectral shapes and band maxima between theoretical and experimental spectra. Though this study utilized vertical excitation energies and approximated band shape using a Gaussian function with no vibrational considerations, the theoretical spectra produced were able to replicate the overall shape of the experimental spectra quite accurately. With this work, we serve to provide a better understanding on how excited-state methods perform for various types of systems and offer insight into which methods may be used as a more black-box approach to simulating spectra for larger molecules with these functional groups.

## Supporting Information Available

Cartesian coordinates of optimized CCSD(T) and M06-2X geometries, CCSD(T) and M06-2X harmonic frequencies, CCSD(T) and M06-2X transition energies, quantitative metrics and bandwidth parameter (γ) for each molecule and method, spectra for each molecule and method, and further comparisons with all methods available for water, ethene, acetaldehyde, and acetone in the QUEST Database. (PDF)
Experimental VUV excitation energies, absorption cross sections, and uncertainties for each molecule. (XLSX)

## References

[ref1] Doner A. C.; Christianson M. G.; Davis J. C.; Koritzke A. L.; Larsson A.; Frandsen K.; Rotavera B. Vacuum-ultraviolet absorption cross-sections of functionalized cyclic hydrocarbons: Six-membered rings. J. Quant. Spectrosc. Radiat. Transfer 2019, 236, 106603 10.1016/j.jqsrt.2019.106603.

[ref2] Christianson M. G.; Doner A. C.; Koritzke A. L.; Frandsen K.; Rotavera B. Vacuum-ultraviolet absorption cross-sections of functionalized cyclic hydrocarbons: Five-membered rings. J. Quant. Spectrosc. Radiat. Transfer 2021, 258, 107274 10.1016/j.jqsrt.2020.107274.

[ref3] Doner A. C.; Webb A. R.; Dewey N. S.; Hartness S. W.; Christianson M. G.; Koritzke A. L.; Larsson A.; Frandsen K. M.; Rotavera B. Vacuum-ultraviolet absorption cross-sections of functionalized four-carbon species. J. Quant. Spectrosc. Radiat. Transfer 2022, 292, 108346 10.1016/j.jqsrt.2022.108346.

[ref4] Leach S.; Schwell M.; Un S.; Jochims H.-W.; Baumgärtel H. VUV absorption spectrum of acetic acid between 6 and 20 eV. Chem. Phys. 2006, 321, 159–170. 10.1016/j.chemphys.2005.08.044.

[ref5] Feng D.; Wang S.; Zhuang Q.; Guo P.; Wu P.; Han Z. Joint theoretical and experimental study of the UV absorption spectra of polybenzoxazoles. J. Mol. Struct. 2004, 707, 169–177. 10.1016/j.molstruc.2004.07.018.

[ref6] Grante I.; Actins A.; Orola L. Protonation effects on the UV/Vis absorption spectra of imatinib: A theoretical and experimental study. Spectrochim. Acta A Mol. Biomol. Spectrosc. 2014, 129, 326–332. 10.1016/j.saa.2014.03.059. 24747856

[ref7] Quarti C.; Grancini G.; Mosconi E.; Bruno P.; Ball J. M.; Lee M. M.; Snaith H. J.; Petrozza A.; De Angelis F. The Raman spectrum of the CH_3_NH_3_PbI_3_ hybrid perovskite: interplay of theory and experiment. J. Phys. Chem. Lett. 2014, 5, 279–284. 10.1021/jz402589q. 26270700

[ref8] Jones G. S.; Mavrikakis M.; Barteau M. A.; Vohs J. M. First synthesis, experimental and theoretical vibrational spectra of an oxametallacycle on a metal surface. J. Am. Chem. Soc. 1998, 120, 3196–3204. 10.1021/ja973609h.

[ref9] Hess B. A. Jr; Schaad L. J.; Carsky P.; Zahradnik R. Ab initio calculations of vibrational spectra and their use in the identification of unusual molecules. Chem. Rev. 1986, 86, 709–730. 10.1021/cr00074a004.

[ref10] Schreiber M.; Silva-Junior M. R.; Sauer S. P. A.; Thiel W. Benchmarks for electronically excited states: CASPT2, CC2, CCSD, and CC3. J. Chem. Phys. 2008, 128, 134110 10.1063/1.2889385. 18397056

[ref11] Jacquemin D.; Wathelet V.; Perpète E. A.; Adamo C. Extensive TD-DFT benchmark: singlet-excited states of organic molecules. J. Chem. Theory Comput. 2009, 5, 2420–2435. 10.1021/ct900298e. 26616623

[ref12] Chrayteh A.; Blondel A.; Loos P.-F.; Jacquemin D. Mountaineering Strategy to Excited States: Highly Accurate Oscillator Strengths and Dipole Moments of Small Molecules. J. Chem. Theory Comput. 2021, 17, 416–438. 10.1021/acs.jctc.0c01111. 33256412

[ref13] Sarkar R.; Boggio-Pasqua M.; Loos P.-F.; Jacquemin D. Benchmarking TD-DFT and wave function methods for oscillator strengths and excited-state dipole moments. J. Chem. Theory Comput. 2021, 17, 1117–1132. 10.1021/acs.jctc.0c01228. 33492950

[ref14] Loos P.-F.; Scemama A.; Blondel A.; Garniron Y.; Caffarel M.; Jacquemin D. A mountaineering strategy to excited states: Highly accurate reference energies and benchmarks. J. Chem. Theory Comput. 2018, 14, 4360–4379. 10.1021/acs.jctc.8b00406. 29966098

[ref15] Leang S. S.; Zahariev F.; Gordon M. S. Benchmarking the performance of time-dependent density functional methods. J. Chem. Phys. 2012, 136, 104101 10.1063/1.3689445. 22423822

[ref16] Dreuw A.; Head-Gordon M. Single-reference ab initio methods for the calculation of excited states of large molecules. Chem. Rev. 2005, 105, 4009–4037. 10.1021/cr0505627. 16277369

[ref17] Silva-Junior M. R.; Sauer S. P. A.; Schreiber M.; Thiel W. Basis set effects on coupled cluster benchmarks of electronically excited states: CC3, CCSDR(3) and CC2. Mol. Phys. 2010, 108, 453–465. 10.1080/00268970903549047.

[ref18] Jacquemin D.; Duchemin I.; Blase X. 0–0 energies using hybrid schemes: Benchmarks of TD-DFT, CIS(D), ADC(2), CC2, and BSE/GW formalisms for 80 real-life compounds. J. Chem. Theory Comput. 2015, 11, 5340–5359. 10.1021/acs.jctc.5b00619. 26574326 PMC4642227

[ref19] Watson T. J. Jr; Lotrich V. F.; Szalay P. G.; Perera A.; Bartlett R. J. Benchmarking for perturbative triple-excitations in EE-EOM-CC methods. J. Phys. Chem. A 2013, 117, 2569–2579. 10.1021/jp308634q. 23406329

[ref20] Helmich-Paris B. Benchmarks for Electronically Excited States with CASSCF Methods. J. Chem. Theory Comput. 2019, 15, 4170–4179. 10.1021/acs.jctc.9b00325. 31136706 PMC6620717

[ref21] Silva-Junior M. R.; Schreiber M.; Sauer S. P. A.; Thiel W. Benchmarks for electronically excited states: Time-dependent density functional theory and density functional theory based multireference configuration interaction. J. Chem. Phys. 2008, 129, 104103 10.1063/1.2973541. 19044904

[ref22] Laurent A. D.; Jacquemin D. TD-DFT benchmarks: a review. Int. J. Quantum Chem. 2013, 113, 2019–2039. 10.1002/qua.24438.

[ref23] Fahim Z. M. E.; Bouzzine S. M.; Youssef A. A.; Bouachrine M.; Hamidi M. Ground state geometries, UV/vis absorption spectra and charge transfer properties of triphenylamine-thiophenes based dyes for DSSCs: A TD-DFT benchmark study. Comput. Theor. Chem. 2018, 1125, 39–48. 10.1016/j.comptc.2018.01.002.

[ref24] Jacquemin D.; Perpète E. A.; Scuseria G. E.; Ciofini I.; Adamo C. TD-DFT performance for the visible absorption spectra of organic dyes: conventional versus long-range hybrids. J. Chem. Theory Comput. 2008, 4, 123–135. 10.1021/ct700187z. 26619986

[ref25] Jacquemin D.; Perpète E. A.; Ciofini I.; Adamo C. Assessment of functionals for TD-DFT calculations of singlet-triplet transitions. J. Chem. Theory Comput. 2010, 6, 1532–1537. 10.1021/ct100005d. 26615688

[ref26] Caricato M.; Trucks G. W.; Frisch M. J.; Wiberg K. B. Electronic transition energies: A study of the performance of a large range of single reference density functional and wave function methods on valence and Rydberg states compared to experiment. J. Chem. Theory Comput. 2010, 6, 370–383. 10.1021/ct9005129. 26617296

[ref27] Isegawa M.; Peverati R.; Truhlar D. G. Performance of recent and high-performance approximate density functionals for time-dependent density functional theory calculations of valence and Rydberg electronic transition energies. J. Chem. Phys. 2012, 137, 244104 10.1063/1.4769078. 23277925

[ref28] Jacquemin D.; Mennucci B.; Adamo C. Excited-state calculations with TD-DFT: from benchmarks to simulations in complex environments. Phys. Chem. Chem. Phys. 2011, 13, 16987–16998. 10.1039/c1cp22144b. 21881657

[ref29] Jacquemin D.; Perpète E. A.; Ciofini I.; Adamo C. Assessment of the ωB97 family for excited-state calculations. Theor. Chem. Acc. 2011, 128, 127–136. 10.1007/s00214-010-0783-x.

[ref30] Guido C. A.; Knecht S.; Kongsted J.; Mennucci B. Benchmarking time-dependent density functional theory for excited state geometries of organic molecules in gas-phase and in solution. J. Chem. Theory Comput. 2013, 9, 2209–2220. 10.1021/ct400021c. 26583715

[ref31] Matsuzawa N. N.; Ishitani A.; Dixon D. A.; Uda T. Time-dependent density functional theory calculations of photoabsorption spectra in the vacuum ultraviolet region. J. Phys. Chem. A 2001, 105, 4953–4962. 10.1021/jp003937v.

[ref32] Jacquemin D.; Perpète E. A.; Vydrov O. A.; Scuseria G. E.; Adamo C. Assessment of long-range corrected functionals performance for n → π* transitions in organic dyes. J. Chem. Phys. 2007, 127, 094102 10.1063/1.2770700. 17824727

[ref33] Peach M. J. G.; Benfield P.; Helgaker T.; Tozer D. J. Excitation energies in density functional theory: An evaluation and a diagnostic test. J. Chem. Phys. 2008, 128, 044118 10.1063/1.2831900. 18247941

[ref34] Tao J.; Tretiak S.; Zhu J.-X. Performance of a nonempirical meta–generalized gradient approximation density functional for excitation energies. J. Chem. Phys. 2008, 128, 084110 10.1063/1.2837831. 18315036

[ref35] Rohrdanz M. A.; Martins K. M.; Herbert J. M. A long-range-corrected density functional that performs well for both ground-state properties and time-dependent density functional theory excitation energies, including charge-transfer excited states. J. Chem. Phys. 2009, 130, 054112 10.1063/1.3073302. 19206963

[ref36] Jacquemin D.; Perpète E. A.; Ciofini I.; Adamo C.; Valero R.; Zhao Y.; Truhlar D. G. On the performances of the M06 family of density functionals for electronic excitation energies. J. Chem. Theory Comput. 2010, 6, 2071–2085. 10.1021/ct100119e. 26615935

[ref37] Dierksen M.; Grimme S. The vibronic structure of electronic absorption spectra of large molecules: a time-dependent density functional study on the influence of “Exact” Hartree- Fock exchange. J. Phys. Chem. A 2004, 108, 10225–10237. 10.1021/jp047289h.

[ref38] Furche F.; Ahlrichs R. Adiabatic time-dependent density functional methods for excited state properties. J. Chem. Phys. 2002, 117, 7433–7447. 10.1063/1.1508368.

[ref39] Caricato M.; Trucks G. W.; Frisch M. J.; Wiberg K. B. Oscillator strength: How does TDDFT compare to EOM-CCSD?. J. Chem. Theory Comput. 2011, 7, 456–466. 10.1021/ct100662n. 26596165

[ref40] Adamo C.; Jacquemin D. The calculations of excited-state properties with Time-Dependent Density Functional Theory. Chem. Soc. Rev. 2013, 42, 845–856. 10.1039/C2CS35394F. 23117144

[ref41] Bai S.; Mansour R.; Stojanović L.; Toldo J. M.; Barbatti M. On the origin of the shift between vertical excitation and band maximum in molecular photoabsorption. J. Mol. Model. 2020, 26, 1–9. 10.1007/s00894-020-04355-y. PMC7174274 32318882

[ref42] Santoro F.; Jacquemin D. Going beyond the vertical approximation with time-dependent density functional theory. Wiley Interdiscip. Rev. Comput. Mol. Sci. 2016, 6, 460–486. 10.1002/wcms.1260.

[ref43] Fang C.; Oruganti B.; Durbeej B. How method-dependent are calculated differences between vertical, adiabatic, and 0–0 excitation energies?. J. Phys. Chem. A 2014, 118, 4157–4171. 10.1021/jp501974p. 24848558

[ref44] Send R.; Kühn M.; Furche F. Assessing excited state methods by adiabatic excitation energies. J. Chem. Theory Comput. 2011, 7, 2376–2386. 10.1021/ct200272b. 26606613

[ref45] Charaf-Eddin A.; Planchat A.; Mennucci B.; Adamo C.; Jacquemin D. Choosing a functional for computing absorption and fluorescence band shapes with TD-DFT. J. Chem. Theory Comput. 2013, 9, 2749–2760. 10.1021/ct4000795. 26583866

[ref46] Avila Ferrer F. J.; Cerezo J.; Stendardo E.; Improta R.; Santoro F. Insights for an accurate comparison of computational data to experimental absorption and emission spectra: beyond the vertical transition approximation. J. Chem. Theory Comput. 2013, 9, 2072–2082. 10.1021/ct301107m. 26583553

[ref47] Karunakaran V.; Senyushkina T.; Saroja G.; Liebscher J.; Ernsting N. P. 2-amino-7-nitro-fluorenes in neat and mixed solvents optical band shapes and solvatochromism. J. Phys. Chem. A 2007, 111, 10944–10952. 10.1021/jp073844e. 17929778

[ref48] Barbatti M.; Aquino A. J. A.; Lischka H. The UV absorption of nucleobases: semi-classical ab initio spectra simulations. Phys. Chem. Chem. Phys. 2010, 12, 4959–4967. 10.1039/b924956g. 20445902

[ref49] Bomble Y. J.; Sattelmeyer K. W.; Stanton J. F.; Gauss J. On the vertical excitation energy of cyclopentadiene. J. Chem. Phys. 2004, 121, 5236–5240. 10.1063/1.1780159. 15352816

[ref50] Zobel J. P.; Nogueira J. J.; González L. The IPEA dilemma in CASPT2. Chem. Sci. 2017, 8, 1482–1499. 10.1039/C6SC03759C. 28572908 PMC5452265

[ref51] Runge E.; Gross E. K. Density-functional theory for time-dependent systems. Phys. Rev. Lett. 1984, 52, 997–1000. 10.1103/PhysRevLett.52.997.

[ref52] Casida M. E. Recent Advances In Density Functional Methods (Part I); World Scientific, 1995; pp 155–192.

[ref53] Stratmann R. E.; Scuseria G. E.; Frisch M. J. An efficient implementation of time-dependent density-functional theory for the calculation of excitation energies of large molecules. J. Chem. Phys. 1998, 109, 8218–8224. 10.1063/1.477483.

[ref54] Sekino H.; Bartlett R. J. A linear response, coupled-cluster theory for excitation energy. Int. J. Quantum Chem. 1984, 26, 255–265. 10.1002/qua.560260826.

[ref55] Stanton J. F.; Bartlett R. J. The equation of motion coupled-cluster method. A systematic biorthogonal approach to molecular excitation energies, transition probabilities, and excited state properties. J. Chem. Phys. 1993, 98, 7029–7039. 10.1063/1.464746.

[ref56] Comeau D. C.; Bartlett R. J. The equation-of-motion coupled-cluster method. Applications to open-and closed-shell reference states. Chem. Phys. Lett. 1993, 207, 414–423. 10.1016/0009-2614(93)89023-B.

[ref57] Krylov A. I. Equation-of-motion coupled-cluster methods for open-shell and electronically excited species: The hitchhiker’s guide to Fock space. Annu. Rev. Phys. Chem. 2008, 59, 433–462. 10.1146/annurev.physchem.59.032607.093602. 18173379

[ref58] Becke A. D. Becke’s three parameter hybrid method using the LYP correlation functional. J. Chem. Phys. 1993, 98, 5648–5652. 10.1063/1.464913.

[ref59] Stephens P. J.; Devlin F. J.; Chabalowski C. F.; Frisch M. J. Ab initio calculation of vibrational absorption and circular dichroism spectra using density functional force fields. J. Phys. Chem. 1994, 98, 11623–11627. 10.1021/j100096a001.

[ref60] Becke A. D. A new mixing of Hartree–Fock and local density-functional theories. J. Chem. Phys. 1993, 98, 1372–1377. 10.1063/1.464304.

[ref61] Boese A. D.; Martin J. M. L. Development of density functionals for thermochemical kinetics. J. Chem. Phys. 2004, 121, 3405–3416. 10.1063/1.1774975. 15303903

[ref62] Yanai T.; Tew D. P.; Handy N. C. A new hybrid exchange–correlation functional using the Coulomb-attenuating method (CAM-B3LYP). Chem. Phys. Lett. 2004, 393, 51–57. 10.1016/j.cplett.2004.06.011.

[ref63] Krukau A. V.; Vydrov O. A.; Izmaylov A. F.; Scuseria G. E. Influence of the exchange screening parameter on the performance of screened hybrid functionals. J. Chem. Phys. 2006, 125, 224106 10.1063/1.2404663. 17176133

[ref64] Heyd J.; Scuseria G. E.; Ernzerhof M. Hybrid functionals based on a screened Coulomb potential. J. Chem. Phys. 2003, 118, 8207–8215. 10.1063/1.1564060.

[ref65] Zhao Y.; Truhlar D. G. A new local density functional for main-group thermochemistry, transition metal bonding, thermochemical kinetics, and noncovalent interactions. J. Chem. Phys. 2006, 125, 194101 10.1063/1.2370993. 17129083

[ref66] Zhao Y.; Truhlar D. G. The M06 suite of density functionals for main group thermochemistry, thermochemical kinetics, noncovalent interactions, excited states, and transition elements: two new functionals and systematic testing of four M06-class functionals and 12 other functionals. Theor. Chem. Acc. 2008, 120, 215–241. 10.1007/s00214-007-0310-x.

[ref67] Peverati R.; Truhlar D. G. Improving the accuracy of hybrid meta-GGA density functionals by range separation. J. Phys. Chem. Lett. 2011, 2, 2810–2817. 10.1021/jz201170d.

[ref68] Adamo C.; Barone V. Toward reliable density functional methods without adjustable parameters: The PBE0 model. J. Chem. Phys. 1999, 110, 6158–6170. 10.1063/1.478522.

[ref69] Ernzerhof M.; Scuseria G. E. Assessment of the Perdew–Burke–Ernzerhof exchange-correlation functional. J. Chem. Phys. 1999, 110, 5029–5036. 10.1063/1.478401. 15268348

[ref70] Chai J.-D.; Head-Gordon M. Long-range corrected hybrid density functionals with damped atom–atom dispersion corrections. Phys. Chem. Chem. Phys. 2008, 10, 6615–6620. 10.1039/b810189b. 18989472

[ref71] Chai J.-D.; Head-Gordon M. Systematic optimization of long-range corrected hybrid density functionals. J. Chem. Phys. 2008, 128, 084106 10.1063/1.2834918. 18315032

[ref72] Xu X.; Goddard W. A. III The X3LYP extended density functional for accurate descriptions of nonbond interactions, spin states, and thermochemical properties. Proc. Natl. Acad. Sci. U.S.A. 2004, 101, 2673–2677. 10.1073/pnas.0308730100. 14981235 PMC374194

[ref73] Dunning T. H. Jr Gaussian basis sets for use in correlated molecular calculations. I. The atoms boron through neon and hydrogen. J. Chem. Phys. 1989, 90, 1007–1023. 10.1063/1.456153.

[ref74] Kendall R. A.; Dunning T. H. Jr; Harrison R. J. Electron affinities of the first-row atoms revisited. Systematic basis sets and wave functions. J. Chem. Phys. 1992, 96, 6796–6806. 10.1063/1.462569.

[ref75] Woon D. E.; Dunning T. H. Jr Gaussian basis sets for use in correlated molecular calculations. IV. Calculation of static electrical response properties. J. Chem. Phys. 1994, 100, 2975–2988. 10.1063/1.466439.

[ref76] Pritchard B. P.; Altarawy D.; Didier B.; Gibson T. D.; Windus T. L. New basis set exchange: An open, up-to-date resource for the molecular sciences community. J. Chem. Inf. Model. 2019, 59, 4814–4820. 10.1021/acs.jcim.9b00725. 31600445

[ref77] Feller D. The role of databases in support of computational chemistry calculations. J. Comput. Chem. 1996, 17, 1571–1586. 10.1002/(SICI)1096-987X(199610)17:13<1571::AID-JCC9>3.0.CO;2-P.

[ref78] Schuchardt K. L.; Didier B. T.; Elsethagen T.; Sun L.; Gurumoorthi V.; Chase J.; Li J.; Windus T. L. Basis set exchange: a community database for computational sciences. J. Chem. Inf. Model. 2007, 47, 1045–1052. 10.1021/ci600510j. 17428029

[ref79] Bootsma A. N.; Wheeler S. Popular integration grids can result in large errors in DFT-computed free energies. ChemRxiv 2019, (preprint) 10.26434/chemrxiv.8864204.v5.

[ref80] Mardirossian N.; Head-Gordon M. How accurate are the Minnesota density functionals for noncovalent interactions, isomerization energies, thermochemistry, and barrier heights involving molecules composed of main-group elements?. J. Chem. Theory Comput. 2016, 12, 4303–4325. 10.1021/acs.jctc.6b00637. 27537680

[ref81] Wheeler S. E.; Houk K. N. Integration grid errors for meta-GGA-predicted reaction energies: Origin of grid errors for the M06 suite of functionals. J. Chem. Theory Comput. 2010, 6, 395–404. 10.1021/ct900639j. 20305831 PMC2840268

[ref82] Smith D. G. A.; Burns L. A.; Simmonett A. C.; Parrish R. M.; Schieber M. C.; Galvelis R.; Kraus P.; Kruse H.; Di Remigio R.; Alenaizan A.; et al. PSI4 1.4: Open-source software for high-throughput quantum chemistry. J. Chem. Phys. 2020, 152, 184108 10.1063/5.0006002. 32414239 PMC7228781

[ref83] Turney J. M.; Simmonett A. C.; Parrish R. M.; Hohenstein E. G.; Evangelista F. A.; Fermann J. T.; Mintz B. J.; Burns L. A.; Wilke J. J.; Abrams M. L.; et al. Psi4: an open-source ab initio electronic structure program. Wiley Interdiscip. Rev. Comput. Mol. Sci. 2012, 2, 556–565. 10.1002/wcms.93.

[ref84] Raghavachari K.; Trucks G. W.; Pople J. A.; Head-Gordon M. A fifth-order perturbation comparison of electron correlation theories. Chem. Phys. Lett. 1989, 157, 479–483. 10.1016/S0009-2614(89)87395-6.

[ref85] Bartlett R. J.; Watts J.; Kucharski S.; Noga J. Non-iterative fifth-order triple and quadruple excitation energy corrections in correlated methods. Chem. Phys. Lett. 1990, 165, 513–522. 10.1016/0009-2614(90)87031-L.

[ref86] Stanton J. F. Why CCSD(T) works: a different perspective. Chem. Phys. Lett. 1997, 281, 130–134. 10.1016/S0009-2614(97)01144-5.

[ref87] Shavitt I.; Bartlett R. J. Many-Body Methods in Chemistry and Physics: MBPT and Coupled-Cluster Theory; Cambridge University Press, 2009.

[ref88] Werner H.-J.; Knowles P. J.; Knizia G.; Manby F. R.; Schütz M.; Celani P.; Györffy W.; Kats D.; Korona T.; Lindh R. MOLPRO, version 2010.1, a package of ab initio programs; 2010; http://www.molpro.net (accessed 2021-08-16).

[ref89] Werner H.-J.; Knowles P. J.; Knizia G.; Manby F. R.; Schütz M. Molpro: a general-purpose quantum chemistry program package. Wiley Interdiscip. Rev. Comput. Mol. Sci. 2012, 2, 242–253. 10.1002/wcms.82.

[ref90] Werner H. J.; Knowles P. J.; Manby F. R.; Black J. A.; Doll K.; Heßelmann A.; Kats D.; Köhn A.; Korona T.; Kreplin D. A.; et al. The Molpro quantum chemistry package. J. Chem. Phys. 2020, 152, 144107 10.1063/5.0005081. 32295355

[ref91] Hampel C.; Peterson K. A.; Werner H.-J. A comparison of the efficiency and accuracy of the quadratic configuration interaction (QCISD), coupled cluster (CCSD), and Brueckner coupled cluster (BCCD) methods. Chem. Phys. Lett. 1992, 190, 1–12. 10.1016/0009-2614(92)86093-W.

[ref92] Deegan M. J.; Knowles P. J. Perturbative corrections to account for triple excitations in closed and open shell coupled cluster theories. Chem. Phys. Lett. 1994, 227, 321–326. 10.1016/0009-2614(94)00815-9.

[ref93] Epifanovsky E.; Gilbert A. T. B.; Feng X.; Lee J.; Mao Y.; Mardirossian N.; Pokhilko P.; White A. F.; Coons M. P.; Dempwolff A. L.; et al. Software for the frontiers of quantum chemistry: An overview of developments in the Q-Chem 5 package. J. Chem. Phys. 2021, 155, 084801 10.1063/5.0055522. 34470363 PMC9984241

[ref94] Shao Y.; Gan Z.; Epifanovsky E.; Gilbert A. T.; Wormit M.; Kussmann J.; Lange A. W.; Behn A.; Deng J.; Feng X.; et al. Advances in molecular quantum chemistry contained in the Q-Chem 4 program package. Mol. Phys. 2015, 113, 184–215. 10.1080/00268976.2014.952696.

[ref95] Doner A. C.; Moran H. A.; Webb A. R.; Christianson M. G.; Koritzke A. L.; Dewey N. S.; Hartness S. W.; Rotavera B. Machine learning models for binary molecular classification using VUV absorption spectra. J. Quant. Spectrosc. Radiat. Transfer 2023, 297, 108438 10.1016/j.jqsrt.2022.108438.

