# Supplementary material for: Simulation of the VUV Absorption Spectra of Oxygenates and Hydrocarbons: A Joint Theoretical–Experimental Study
Source: J Phys Chem A. 2023 Apr 25;127(17):3743–56. doi: 10.1021/acs.jpca.2c07743 (PMC10165657; doi:10.1021/acs.jpca.2c07743)
Supplement: Supplementary file 1 — jp2c07743_si_001.pdf [file jp2c07743_si_001.pdf]

Simulation of the VUV Absorption Spectra of Oxygenates and Hydrocarbons: A Joint Theoretical-Experimental Study

Addison K. Bralick [†,‡], Erica C. Mitchell [†,‡], Anna C. Doner [†], Annabelle R. Webb [†], Matthew G. Christianson [†], Brandon Rotavera [†,¶], Justin M. Turney [‡], Henry F. Schaefer III [†,‡]

[†] Department of Chemistry, University of Georgia, 302 East Campus Road, Athens, Georgia 30602, United States

[‡] Center for Computational Quantum Chemistry, University of Georgia, 1004 Cedar Street, Athens, Georgia 30602, United States

[¶] College of Engineering, University of Georgia, 597 D.W. Brooks Drive, Athens, Georgia 30602, United States

Contains additional information for each molecule including: the CCSD(T) and M06-2X geometries, frequencies, and transition energies, the quantitative metrics, and the resulting spectra. Also includes QUEST database comparisons for molecules available: water, ethene, acetaldehyde, and acetone.

1 WATER **S-2**

2 ETHANE **S-9**

3 PROPANE **S-17**

4 N-BUTANE **S-24**

5 ETHENE **S-33**

6 PROPENE **S-40**

7 CIS-BUTENE **S-48**

8 TRANS-BUTENE **S-56**

9 ACETALDEHYDE **S-64**

10 ACETONE **S-71**

11 METHYL VINYL KETONE **S-79**

12 FORMIC ACID **S-87**

13 ACETIC ACID **S-94**

14 PROPIONIC ACID **S-101**

15 METHANOL **S-109**

16 ETHANOL **S-116**

17 1-PROPANOL **S-124**

18 2-PROPANOL **S-132**

19 DIMETHYL ETHER **S-140**

20 QUEST DATABASE COMPARISONS **S-148**

1 Water

Table S1. Optimized Geometry of Water in Å

	CCSD(T)/d-aug-cc-pVTZ			M06-2X/d-aug-cc-pVTZ		
O	0.0000000000	0.0000000000	-0.0661332160	0.0000000000	0.0000000000	-0.0651187871
H	0.0000000000	0.7586932565	0.5248783540	0.0000000000	-0.7625859704	0.5167412031
H	0.0000000000	-0.7586932565	0.5248783540	0.0000000000	0.7625859704	0.5167412031

Table S2. Frequencies of Water in cm^{-1}

CCSD(T)/d-aug-cc-pVTZ	M06-2X/d-aug-cc-pVTZ
1647.693	1634.657592
3806.959	3852.014853
3918.798	3956.682186

Table S3. Transition Energies of Water in eV

CCSD(T)/d-aug-cc-pVTZ	M06-2X/d-aug-cc-pVTZ
7.585	7.449
9.341	8.911
9.867	9.499
9.998	9.516
10.217	9.814
	10.068

Table S4. Quantitative metrics for the bandwidth (γ), cosine similarity (S), relative integral change (RIC), mean signed error (MSE), and mean average error (MAE) for the band shape of water compared to experiment.

Method	γ	S	RIC	MSE	MAE
B3LYP	0.35	0.825	0.874	1.887	2.283
BH&HLYP	0.35	0.978	0.715	-1.973	1.973
BMK	0.37	0.983	0.695	-1.898	1.898
CAM-B3LYP	0.37	0.932	0.675	1.671	1.676
CC	0.38	0.996	0.377	-1.023	1.023
HSE	0.37	0.958	0.547	1.609	1.609
M06-2X	0.32	0.997	0.248	-0.601	0.601
M11	0.27	0.829	0.799	1.752	2.332
PBE0	0.35	0.953	0.634	1.795	1.795
wB97x-D	0.38	0.964	0.513	1.472	1.472
X3LYP	0.36	0.851	0.736	2.058	2.435

Figure S1. Spectra of Water

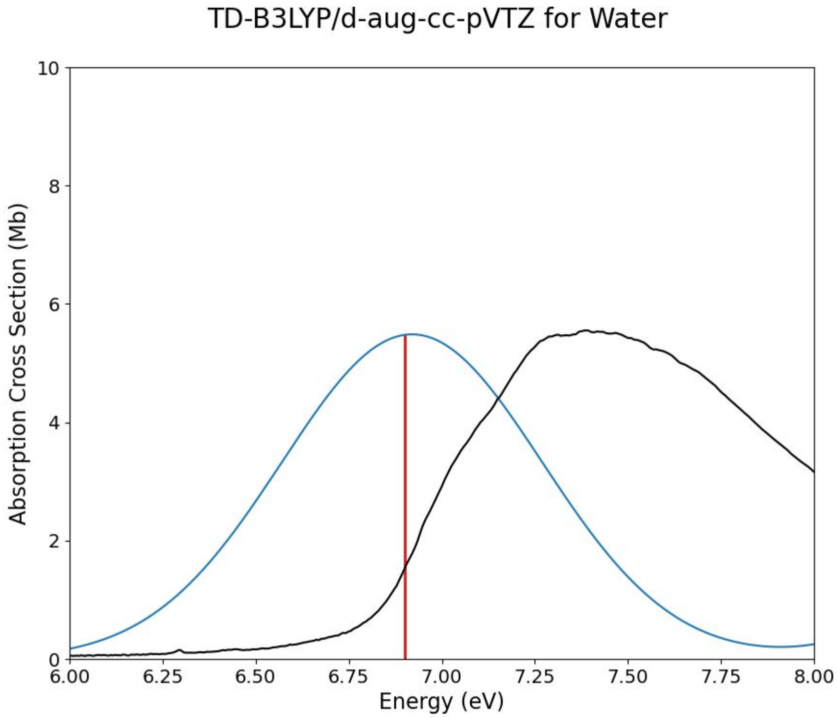

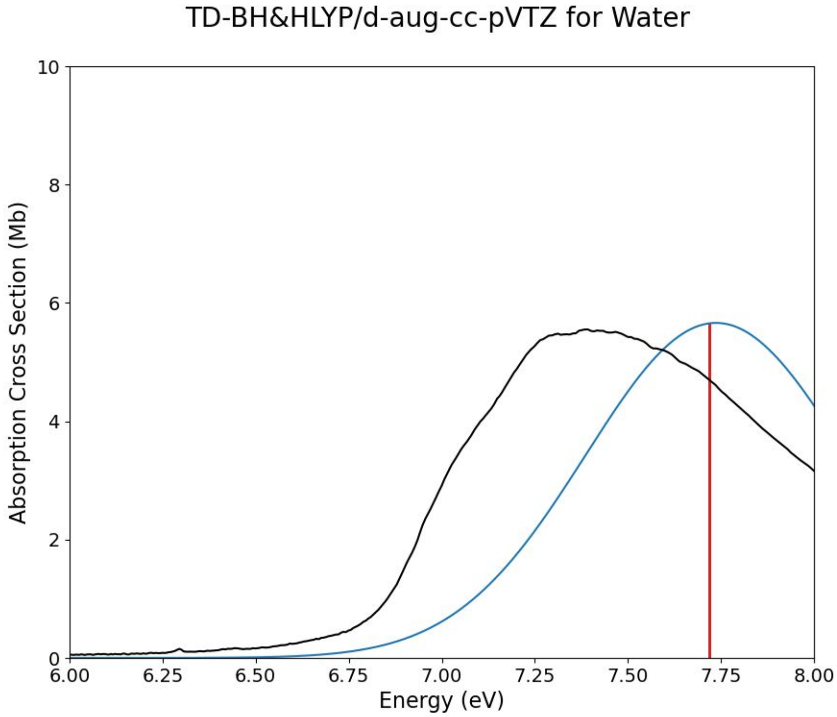

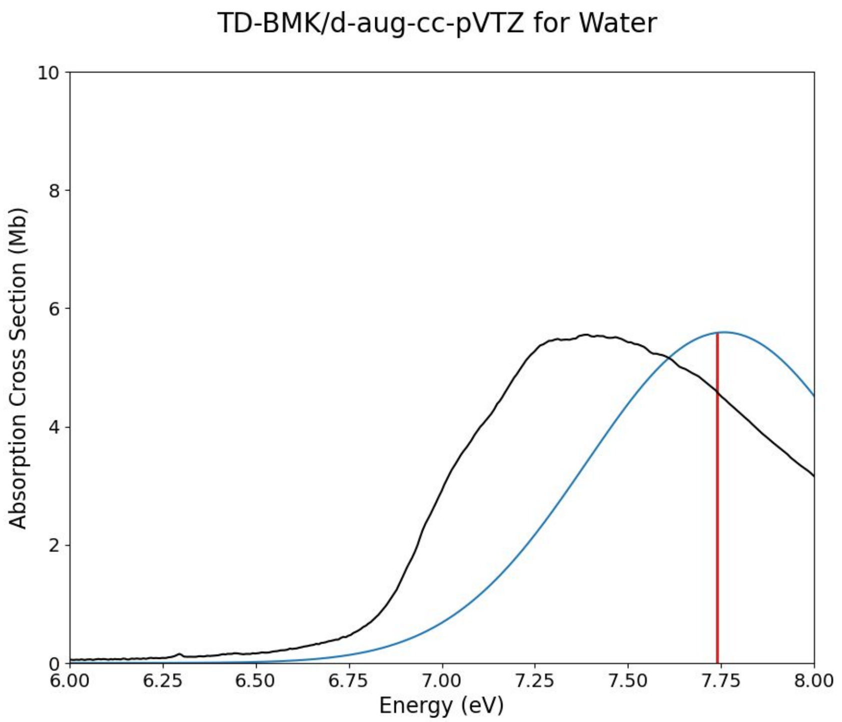
TD-BMK/d-aug-cc-pVTZ for Water
Absorption Cross Section (Mb)
Energy (eV)
0
2
4
6
8
10
6.00
6.25
6.50
6.75
7.00
7.25
7.50
7.75
8.00

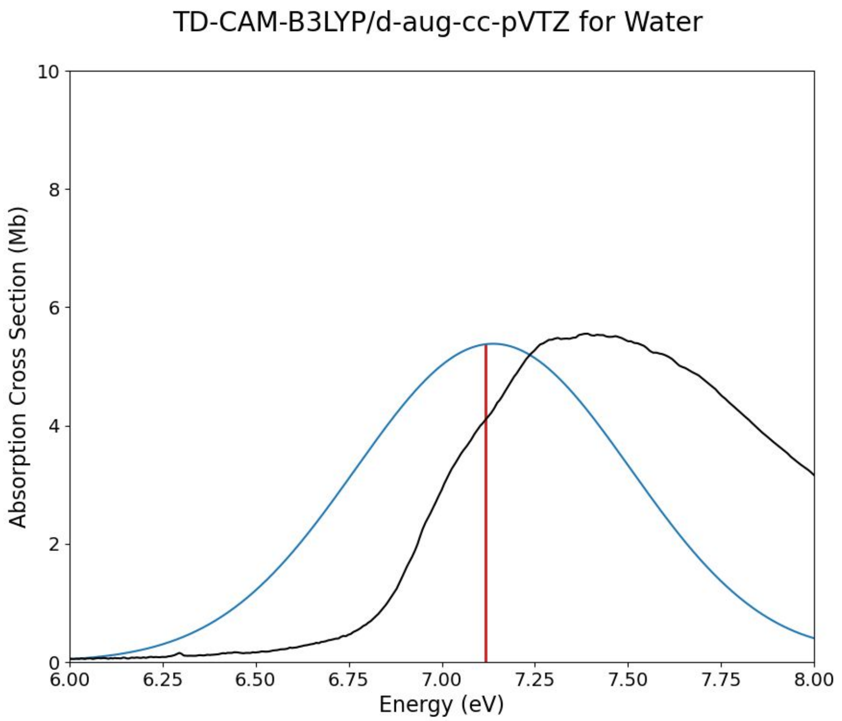
TD-CAM-B3LYP/d-aug-cc-pVTZ for Water
Absorption Cross Section (Mb)
Energy (eV)
0
2
4
6
8
10
6.00
6.25
6.50
6.75
7.00
7.25
7.50
7.75
8.00

EOM-CCSD/d-aug-cc-pVTZ for Water

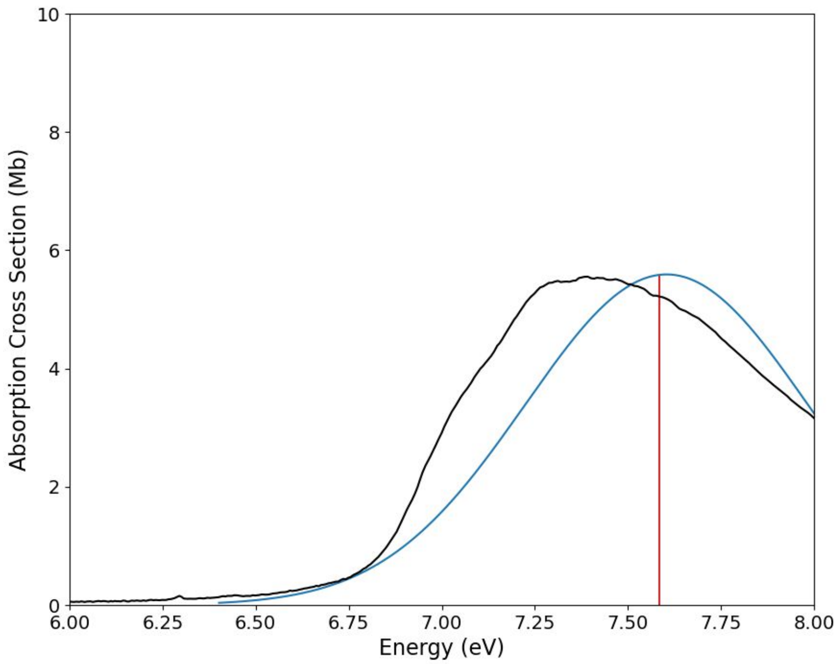

TD-HSE/d-aug-cc-pVTZ for Water

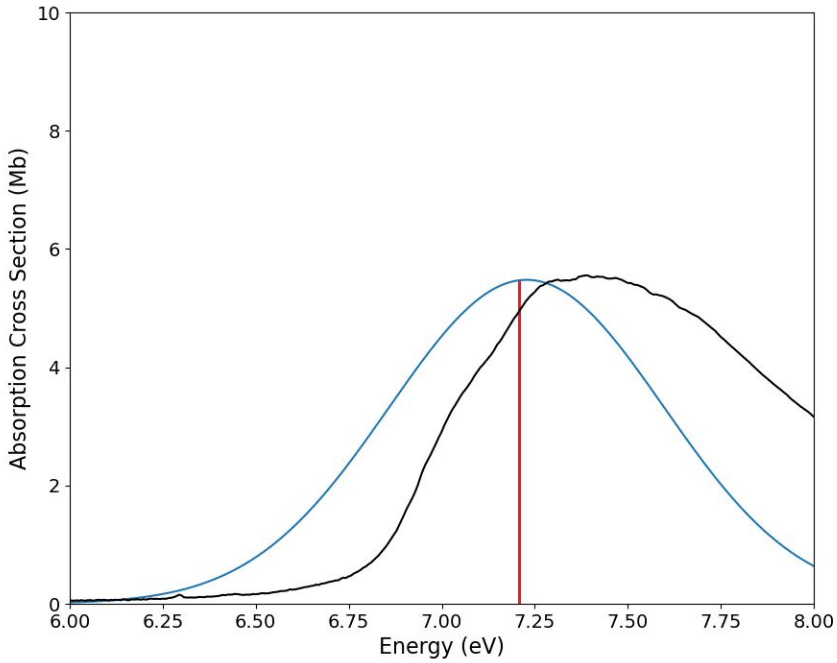

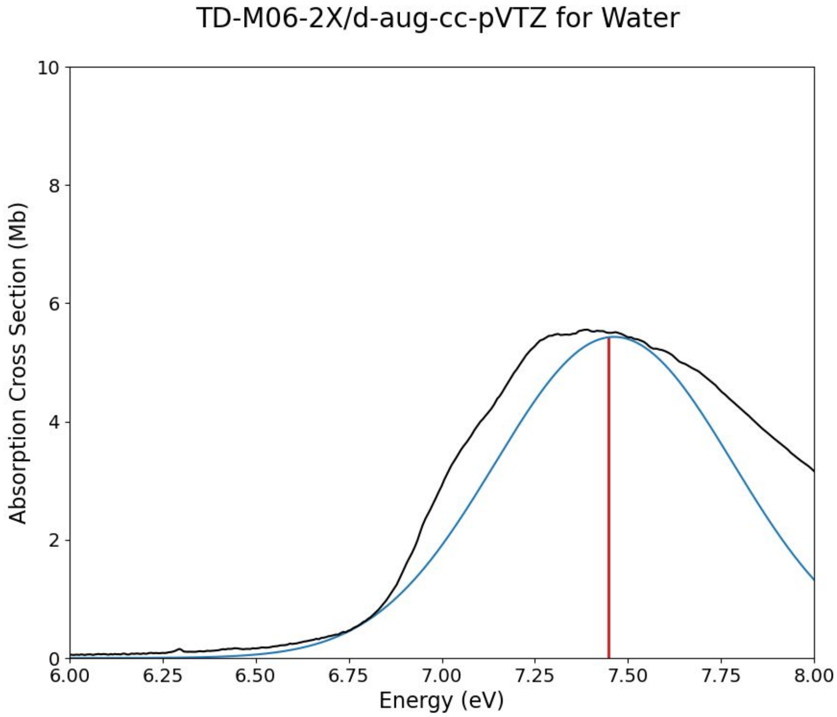

TD-M06-2X/d-aug-cc-pVTZ for Water
Absorption Cross Section (Mb)
Energy (eV)
0
2
4
6
8
10
6.00
6.25
6.50
6.75
7.00
7.25
7.50
7.75
8.00

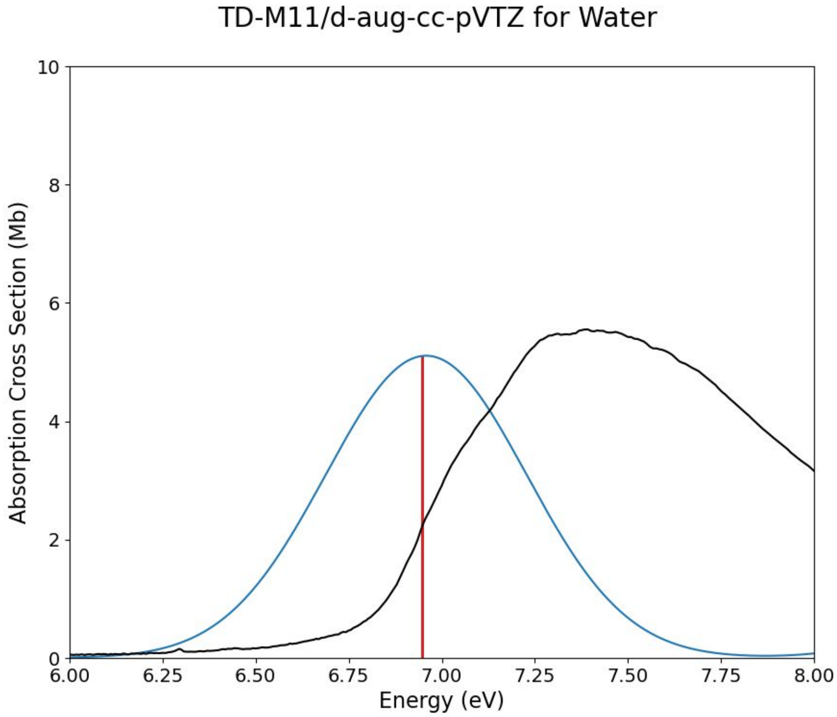

TD-M11/d-aug-cc-pVTZ for Water
Absorption Cross Section (Mb)
Energy (eV)
0
2
4
6
8
10
6.00
6.25
6.50
6.75
7.00
7.25
7.50
7.75
8.00

TD-PBE0/d-aug-cc-pVTZ for Water

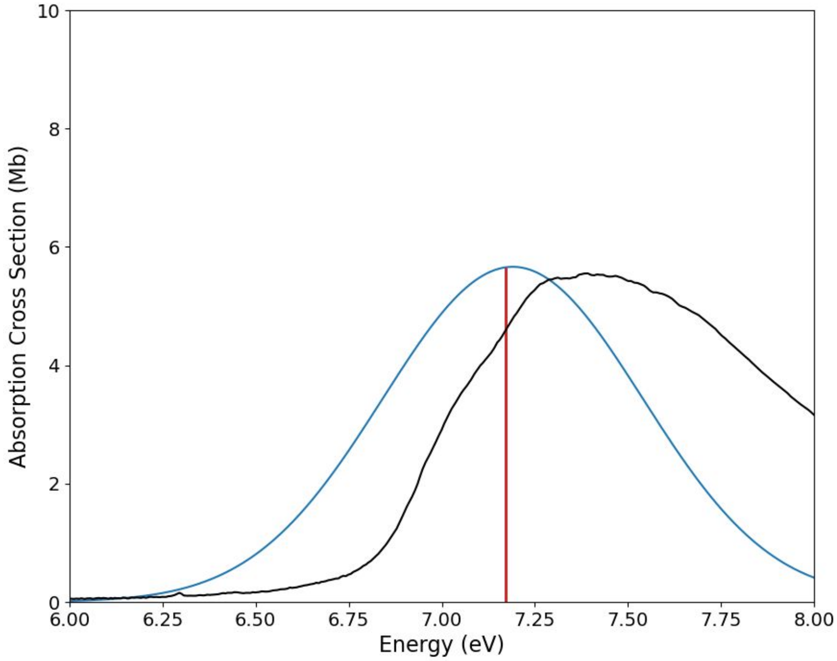

TD-wB97x-D/d-aug-cc-pVTZ for Water

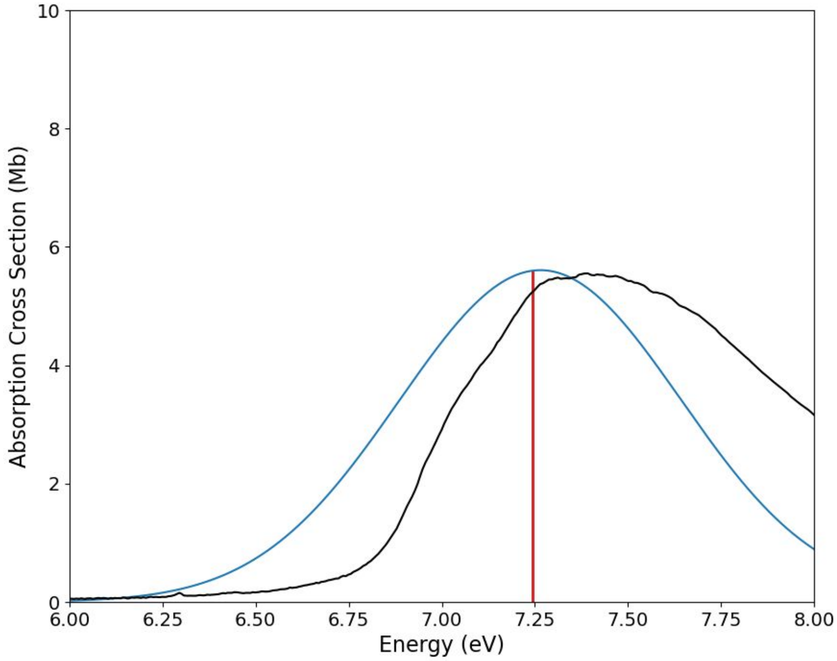

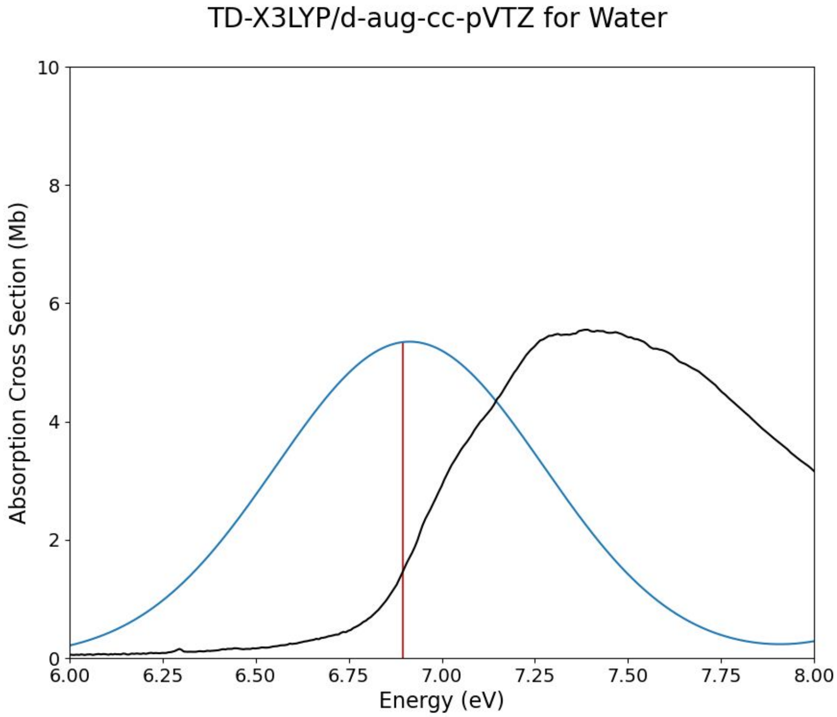
TD-X3LYP/d-aug-cc-pVTZ for Water
Absorption Cross Section (Mb)
Energy (eV)
0
2
4
6
8
10
6.00
6.25
6.50
6.75
7.00
7.25
7.50
7.75
8.00

2 Ethane

Table S5. Optimized Geometry of Ethane in Å

	CCSD(T)/d-aug-cc-pVTZ			M06-2X/d-aug-cc-pVTZ		
C	-0.0000095148	0.0000000000	-0.7650148235	-0.0000035738	-0.7618712393	0.0000000000
C	0.0000095148	0.0000000000	0.7650148235	0.0000035738	0.7618712393	0.0000000000
H	1.0189887049	0.0000000000	-1.1595945092	-1.0154876710	-1.1565167114	0.0000000000
H	-0.5094653038	-0.8825118541	-1.1595754991	0.5077291447	-1.1565021043	-0.8794600207
H	-0.5094653038	0.8825118541	-1.1595754991	0.5077291447	-1.1565021043	0.8794600207
H	-1.0189887049	0.0000000000	1.1595945092	1.0154876710	1.1565167114	0.0000000000
H	0.5094653038	-0.8825118541	1.1595754991	-0.5077291447	1.1565021043	-0.8794600207
H	0.5094653038	0.8825118541	1.1595754991	-0.5077291447	1.1565021043	0.8794600207

Table S6. Frequencies of Ethane in cm^{-1}

CCSD(T)/d-aug-cc-pVTZ	M06-2X/d-aug-cc-pVTZ
309.010	305.676
822.341	819.733
822.379	820.277
1010.724	1020.074
1225.897	1213.935
1225.918	1220.273
1411.918	1404.870
1427.776	1422.781
1512.942	1506.291
1513.012	1507.913
1514.578	1509.957
1514.655	1510.009
3031.320	3068.420
3032.540	3069.985
3090.308	3122.878
3090.357	3125.203
3113.469	3145.995
3113.487	3146.004

Table S7. Transition Energies of Ethane in eV

CCSD(T)/d-aug-cc-pVTZ	M06-2X/d-aug-cc-pVTZ
9.280	8.629
9.281	8.630
9.824	9.309
10.020	9.310
10.020	9.351
	9.433
	9.461
	9.461
	9.527
	10.032

Table S8. Quantitative Metrics for the Bandwidth (γ), cosine similarity (S), relative integral change (RIC), mean signed error (MSE), and mean average error (MAE) for the band shape of ethane compared to experiment.

Method	γ	S	RIC	MSE	MAE
B3LYP	0.25	0.929	0.511	7.780	8.535
BH&HLYP	0.25	0.856	0.920	-15.086	15.086
BMK	0.2	0.827	0.888	-14.554	14.554
CAM-B3LYP	0.3	0.994	0.119	-1.772	2.002
CC	0.3	0.836	0.986	-16.361	16.361
HSE	0.35	0.977	0.211	1.420	3.475
M06-2X	0.45	0.988	0.215	-2.739	3.504
M11	0.25	0.880	0.535	4.321	9.002
PBE0	0.33	0.968	0.256	1.792	4.255
wB97x-D	0.35	0.996	0.220	-3.572	3.572
X3LYP	0.25	0.928	0.542	8.947	9.395

Figure S2. Spectra of Ethane

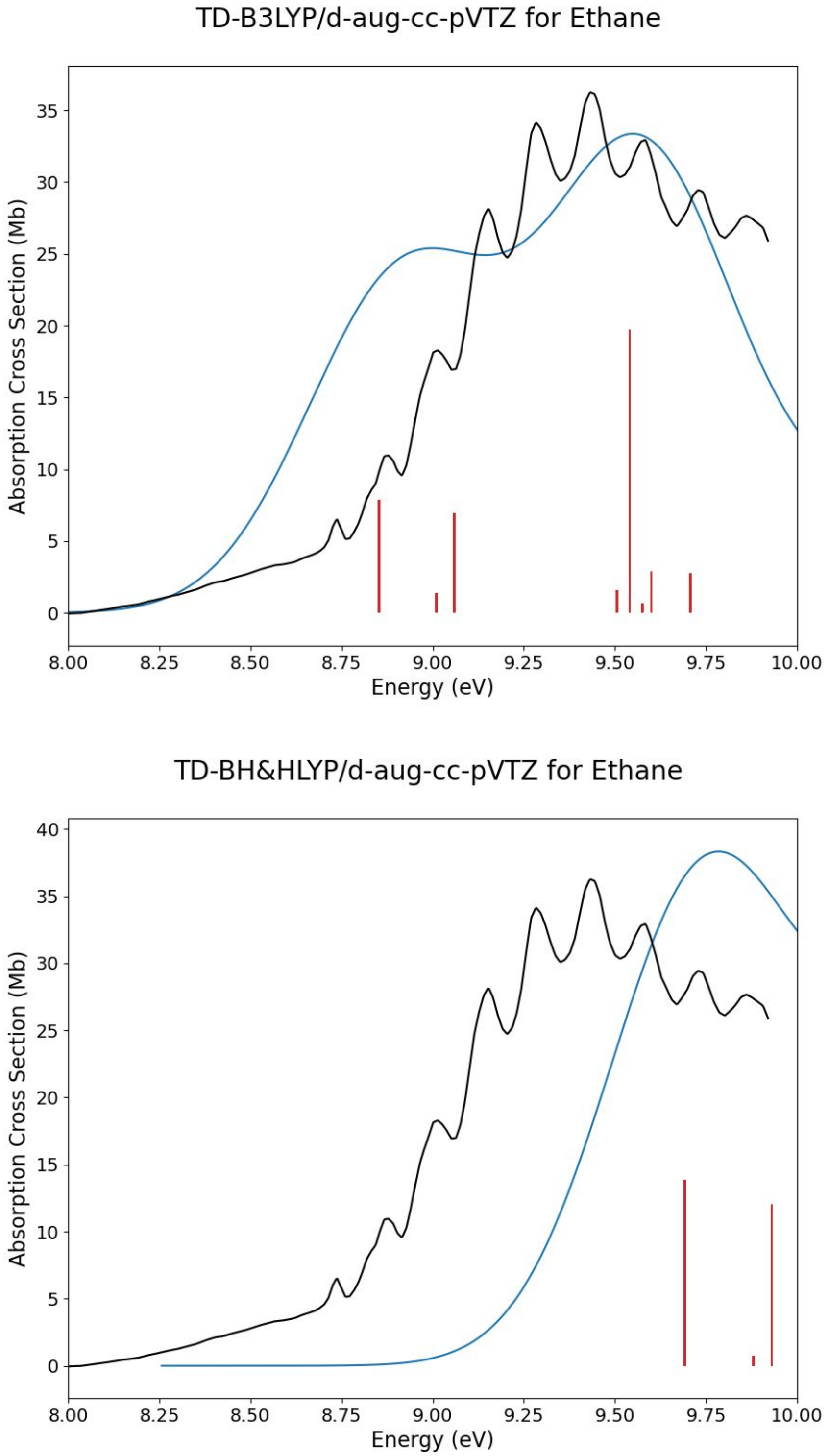

TD-BMK/d-aug-cc-pVTZ for Ethane

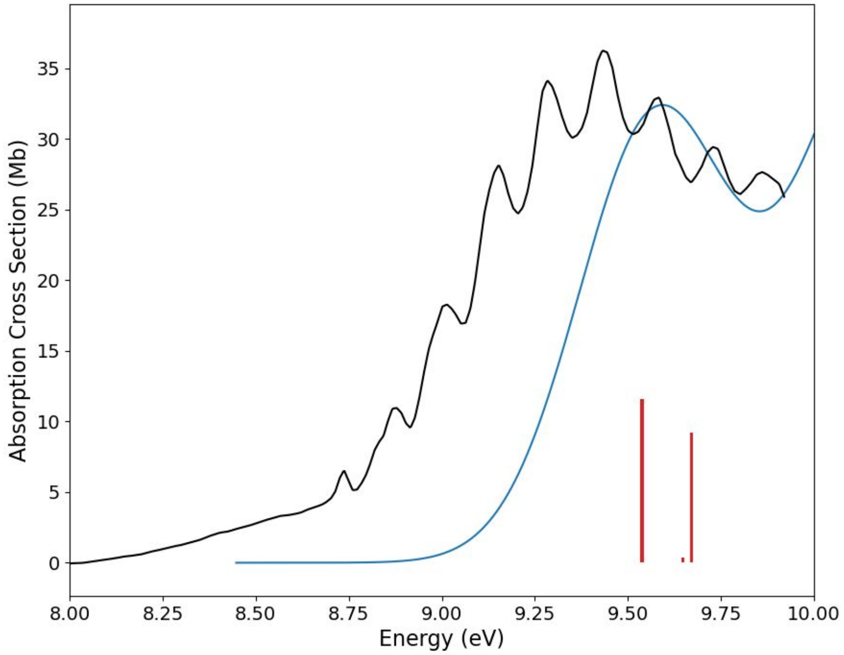

TD-CAM-B3LYP/d-aug-cc-pVTZ for Ethane

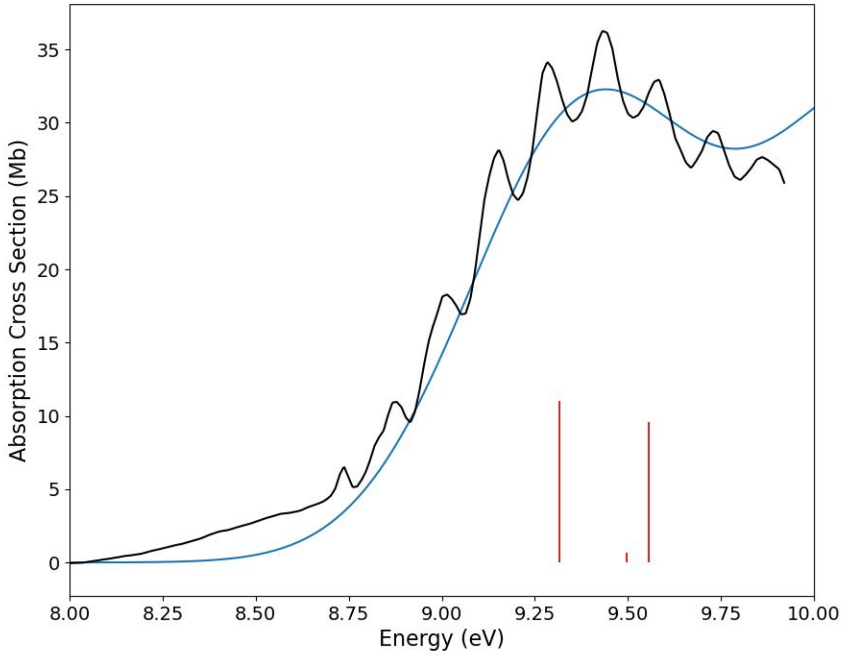

EOM-CCSD/d-aug-cc-pVTZ for Ethane

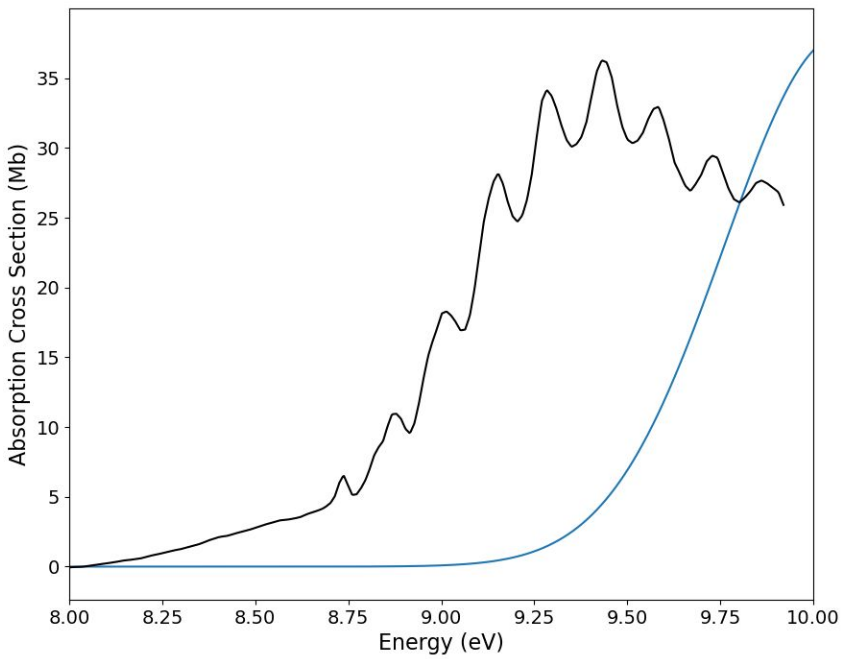

TD-HSE/d-aug-cc-pVTZ for Ethane

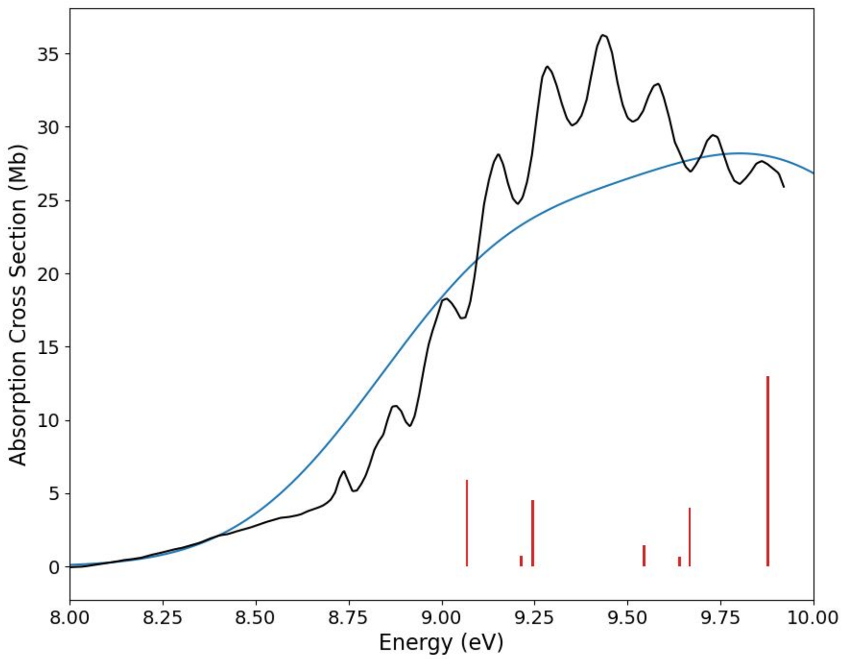

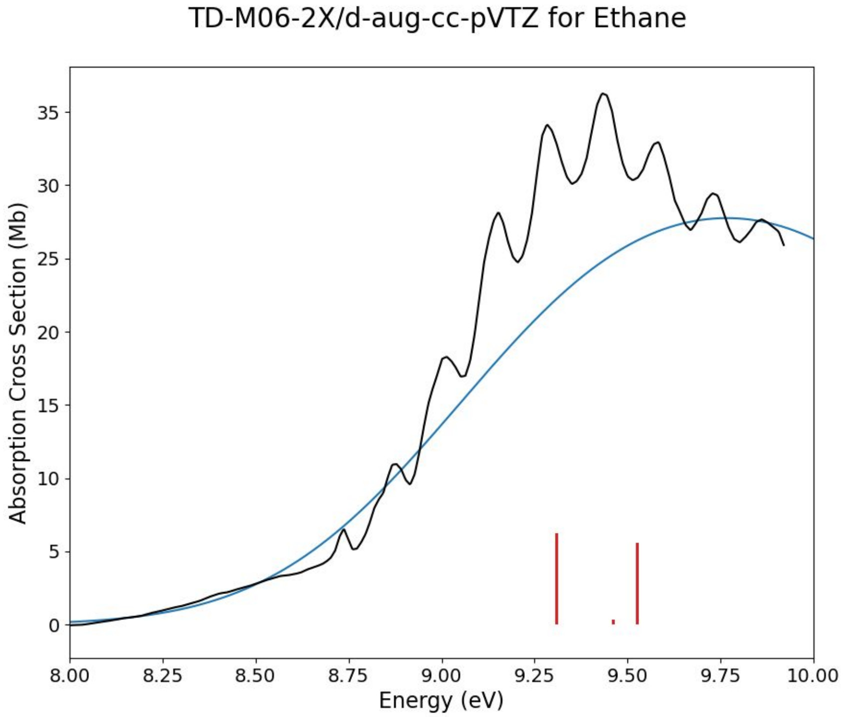
TD-M06-2X/d-aug-cc-pVTZ for Ethane
Absorption Cross Section (Mb)
Energy (eV)
0
5
10
15
20
25
30
35
8.00
8.25
8.50
8.75
9.00
9.25
9.50
9.75
10.00

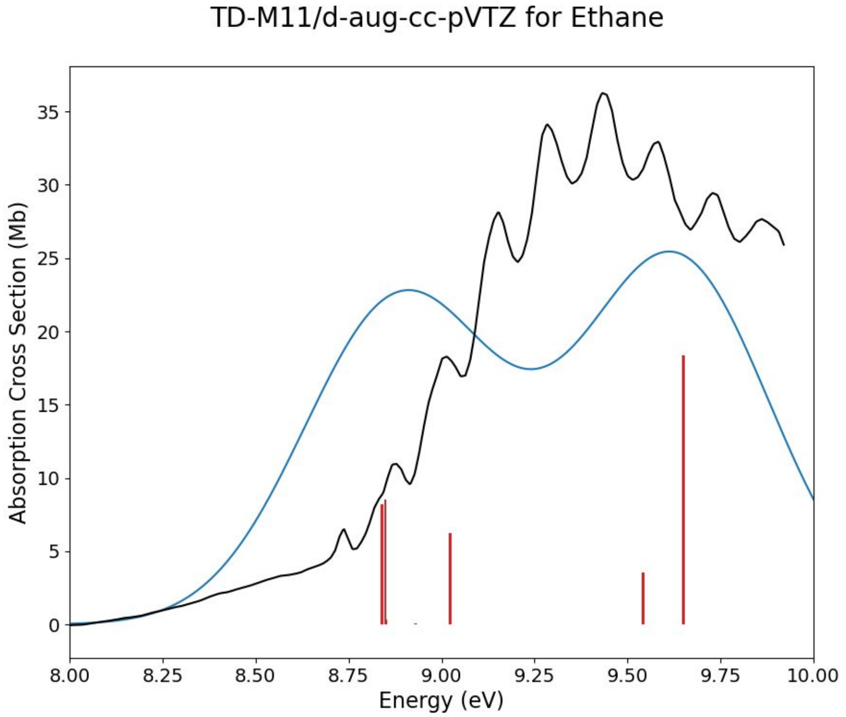
TD-M11/d-aug-cc-pVTZ for Ethane
Absorption Cross Section (Mb)
Energy (eV)
0
5
10
15
20
25
30
35
8.00
8.25
8.50
8.75
9.00
9.25
9.50
9.75
10.00

TD-PBE0/d-aug-cc-pVTZ for Ethane

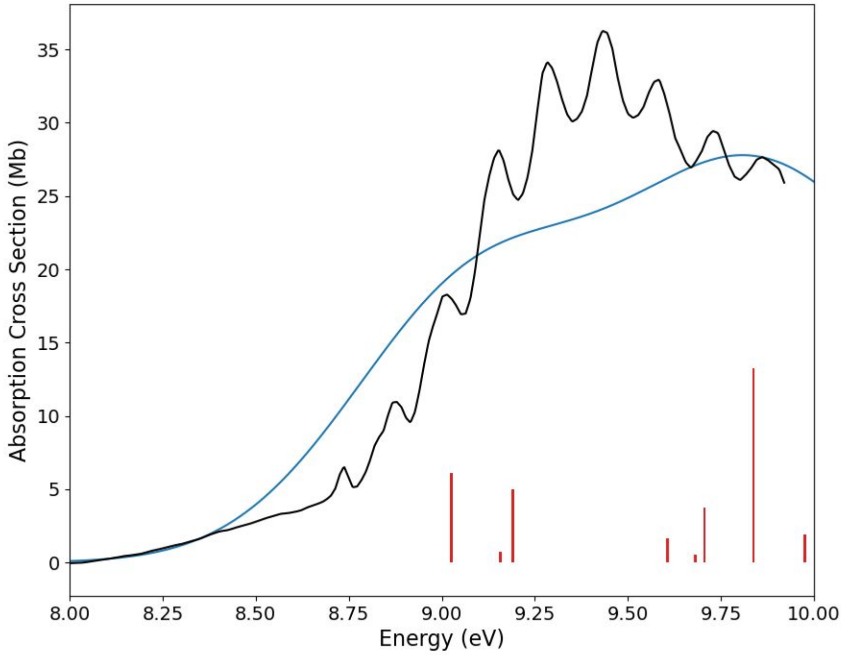

TD-wB97x-D/d-aug-cc-pVTZ for Ethane

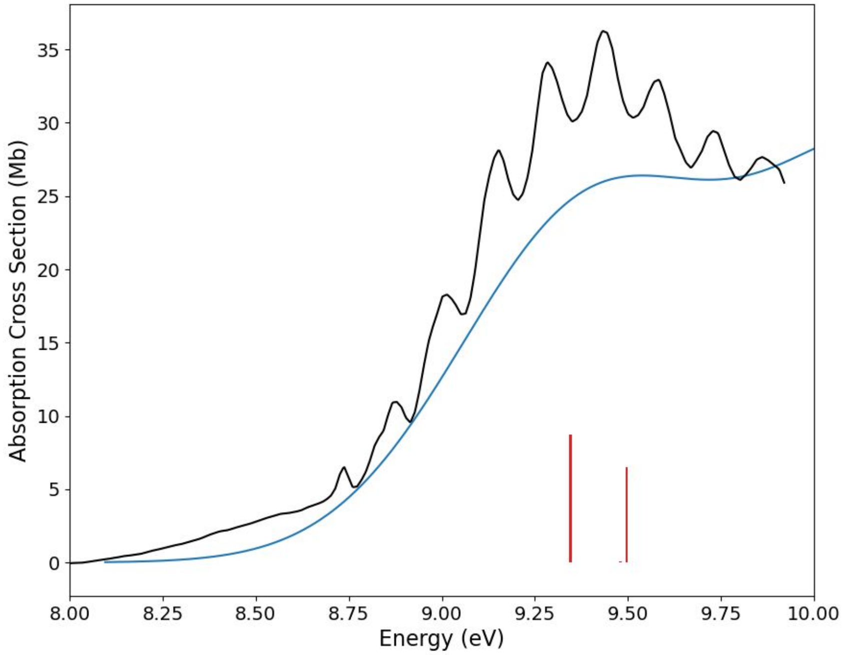

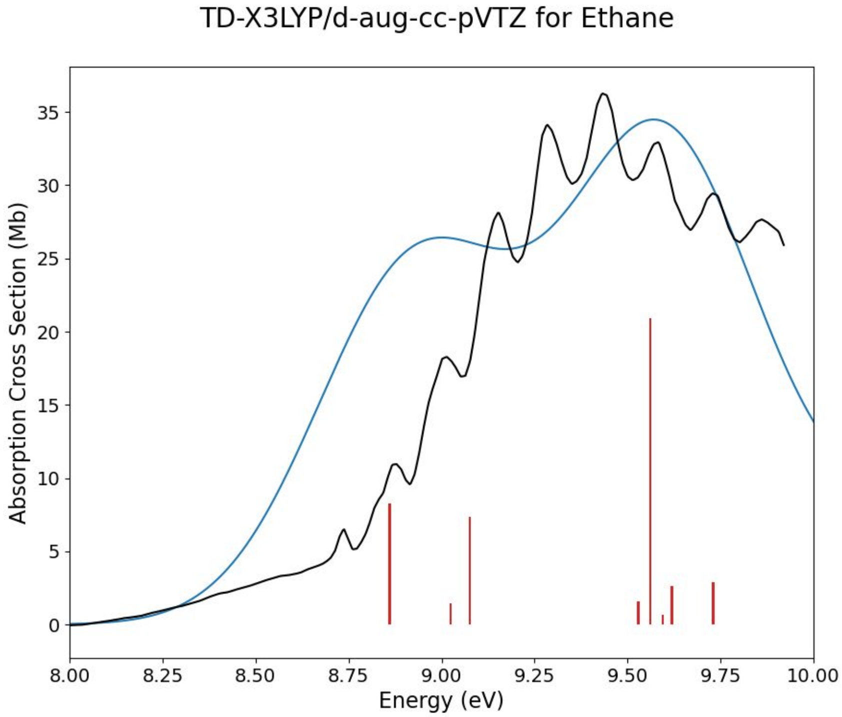
TD-X3LYP/d-aug-cc-pVTZ for Ethane
Absorption Cross Section (Mb)
35
30
25
20
15
10
5
0
8.00
8.25
8.50
8.75
9.00
9.25
9.50
9.75
10.00
Energy (eV)

3 Propane

Table S9. Optimized Geometry of Propane in Å

	CCSD(T)/d-aug-cc-pVTZ			M06-2X/d-aug-cc-pVTZ		
C	0.0000000000	0.0000000000	0.5956859006	0.0000000000	0.0000000000	0.5815938186
C	0.0000000000	1.2680986881	-0.2595457415	-0.0000000000	1.2632191361	-0.2699000437
C	0.0000000000	-1.2680986881	-0.2595457415	0.0000000000	-1.2632191361	-0.2699000437
H	0.8774922850	0.0000000000	1.2507506598	0.8738349639	0.0000000000	1.2358001871
H	-0.8774922850	0.0000000000	1.2507506598	-0.8738349639	-0.0000000000	1.2358001871
H	0.0000000000	2.1701237465	0.3575279784	-0.0000000000	2.1638330439	0.3431487310
H	0.0000000000	-2.1701237465	0.3575279784	0.0000000000	-2.1638330439	0.3431487310
H	0.8833716613	1.3003457174	-0.9044816735	0.8801355204	1.2949000887	-0.9138821585
H	-0.8833716613	1.3003457174	-0.9044816735	-0.8801355204	1.2949000887	-0.9138821585
H	-0.8833716613	-1.3003457174	-0.9044816735	-0.8801355204	-1.2949000887	-0.9138821585
H	0.8833716613	-1.3003457174	-0.9044816735	0.8801355204	-1.2949000887	-0.9138821585

Table S10. Frequencies of Propane in cm^{-1}

CCSD(T)/d-aug-cc-pVTZ	M06-2X/d-aug-cc-pVTZ
220.066	220.260
274.116	280.986
364.405	372.164
748.008	752.301
884.141	892.097
907.980	912.130
930.088	932.298
1071.205	1078.928
1182.742	1182.045
1218.047	1217.051
1320.017	1318.929
1369.597	1367.015
1407.860	1406.165
1422.465	1420.910
1497.927	1494.154
1498.565	1496.240
1504.098	1500.473
1515.802	1512.186
1519.635	1517.458
3019.916	3057.030
3021.497	3057.879
3026.468	3063.293
3054.961	3088.310
3087.535	3119.473
3098.149	3130.249
3098.457	3132.624

3101.084	3134.513

Table S11. Transition Energies of Propane in eV

CCSD(T)/d-aug-cc-pVTZ	M06-2X/d-aug-cc-pVTZ
8.903	8.272
9.034	8.538
9.134	8.591
9.610	8.910
9.649	8.939
9.678	8.965
9.702	9.169
9.739	9.170
9.757	9.180
9.776	9.185
9.787	9.192
9.844	9.223
	9.530
	9.697
	9.724
	9.765
	9.797
	9.801
	9.817
	9.853

Table S12. Quantitative Metrics for the Bandwidth (γ), cosine similarity (S), relative integral change (RIC), mean signed error (MSE), and mean average error (MAE) for the band shape of propane compared to experiment.

Method	γ	S	RIC	MSE	MAE
B3LYP	0.16	0.995	1.242	10.600	10.600
BH&HLYP	0.27	0.933	0.944	-7.956	7.956
BMK	0.16	0.818	0.978	-8.029	8.029
CAM-B3LYP	0.26	0.975	0.558	-4.852	4.852
CC	0.35	0.969	0.936	-7.366	7.366
HSE	0.2	0.985	0.378	2.108	3.163
M06-2X	0.22	0.978	0.575	-5.026	5.026
M11	0.13	0.998	0.468	3.815	3.815
PBE0	0.17	0.976	0.498	2.632	4.221
wB97x-D	0.2	0.915	0.856	-7.247	7.247
X3LYP	0.18	0.995	1.271	10.455	10.455

Figure S3. Spectra of Propane

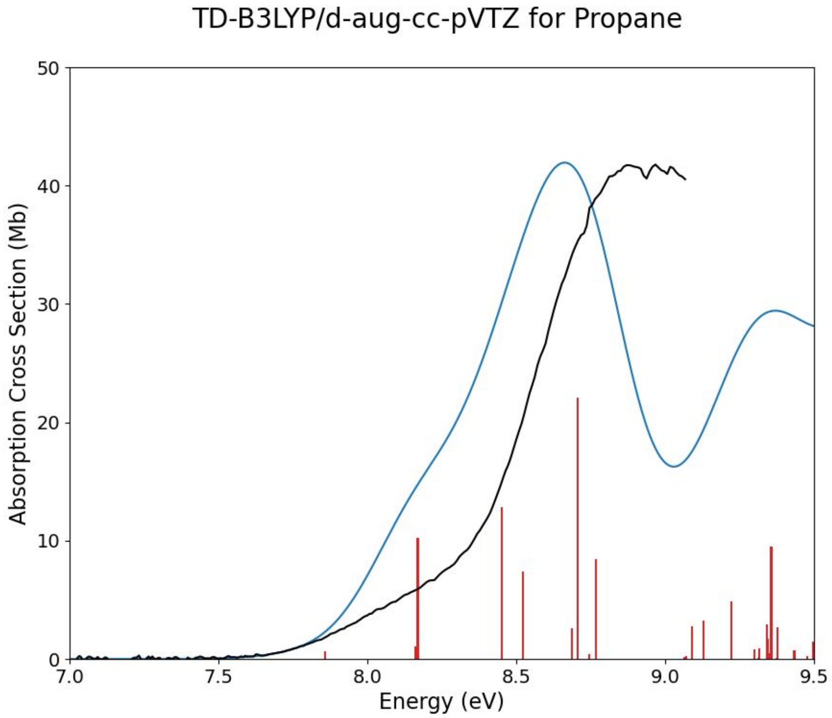

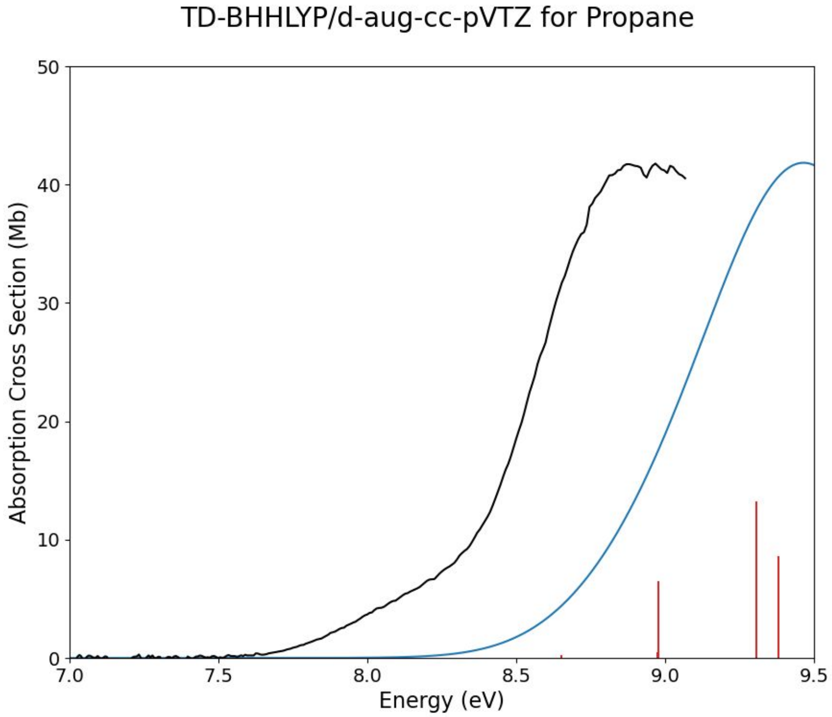

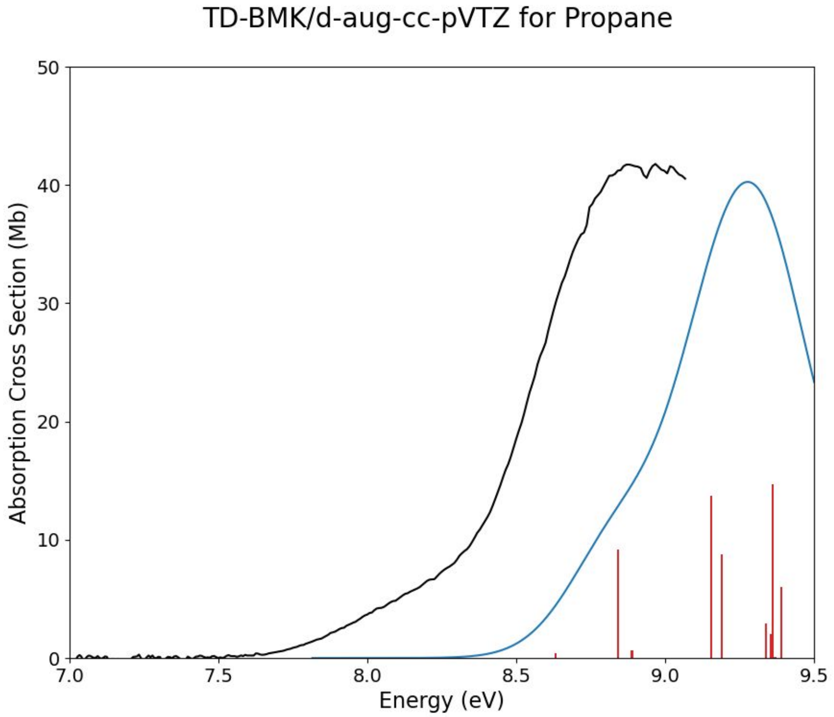
TD-BMK/d-aug-cc-pVTZ for Propane
Absorption Cross Section (Mb)
Energy (eV)
0
10
20
30
40
50
7.0
7.5
8.0
8.5
9.0
9.5

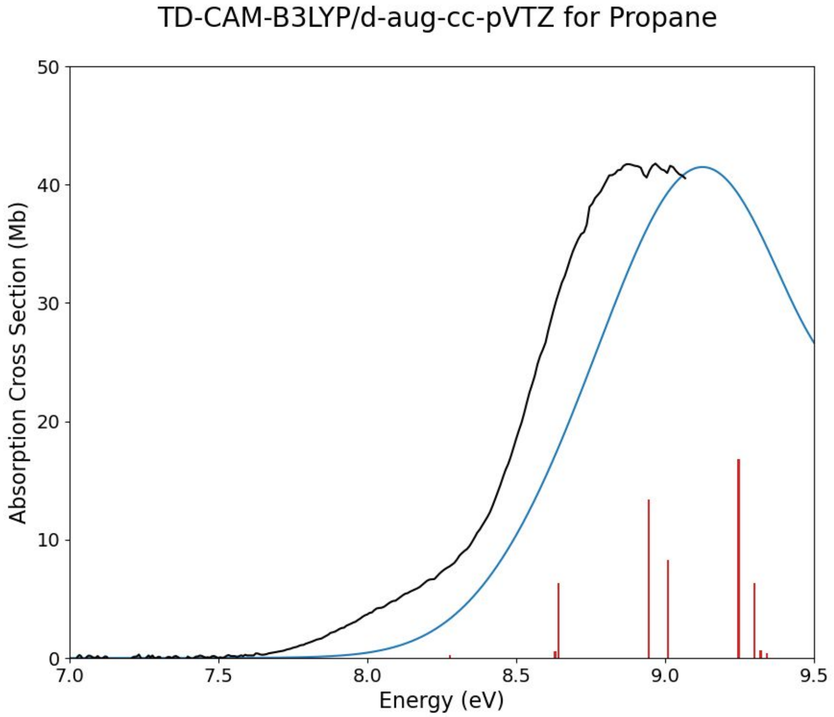
TD-CAM-B3LYP/d-aug-cc-pVTZ for Propane
Absorption Cross Section (Mb)
Energy (eV)
0
10
20
30
40
50
7.0
7.5
8.0
8.5
9.0
9.5

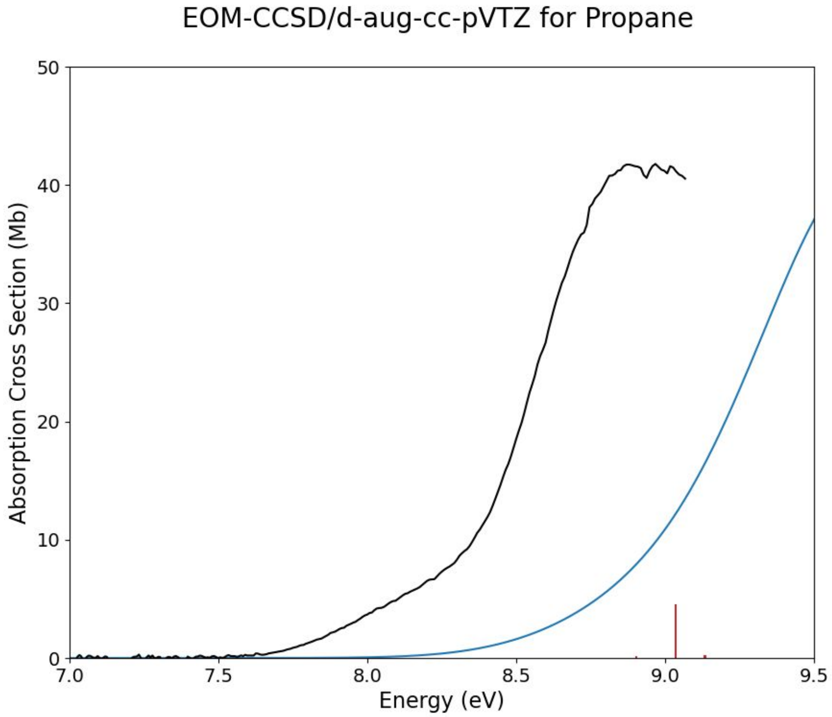
EOM-CCSD/d-aug-cc-pVTZ for Propane
Absorption Cross Section (Mb)
Energy (eV)
0
10
20
30
40
50
7.0
7.5
8.0
8.5
9.0
9.5

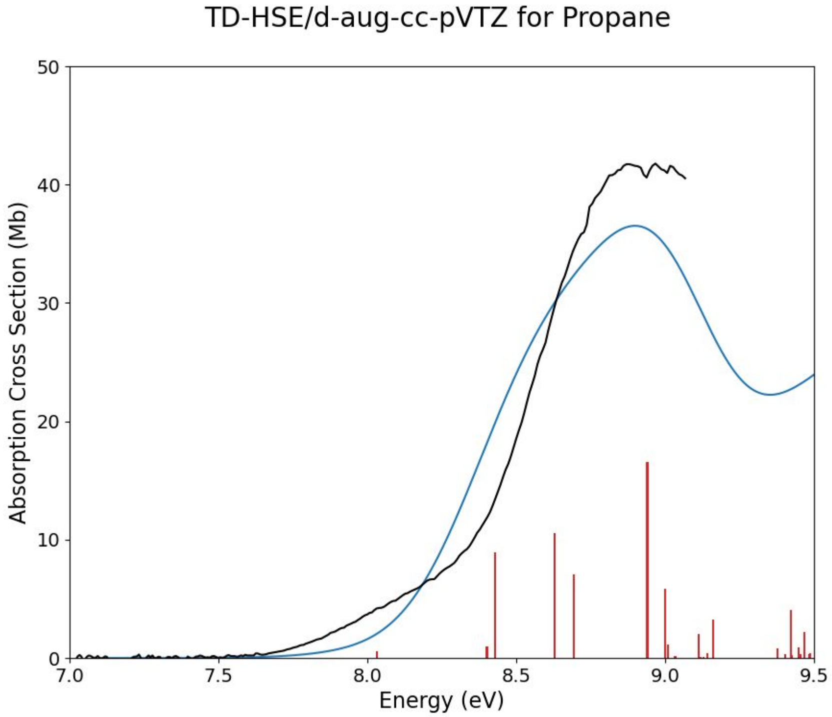
TD-HSE/d-aug-cc-pVTZ for Propane
Absorption Cross Section (Mb)
Energy (eV)
0
10
20
30
40
50
7.0
7.5
8.0
8.5
9.0
9.5

TD-M06-2X/d-aug-cc-pVTZ for Propane

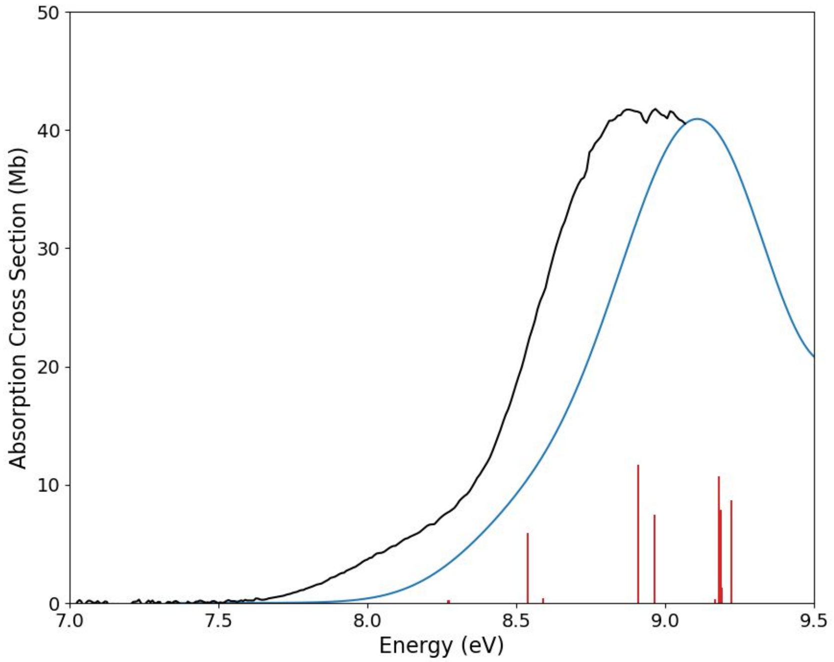

TD-M11/d-aug-cc-pVTZ for Propane

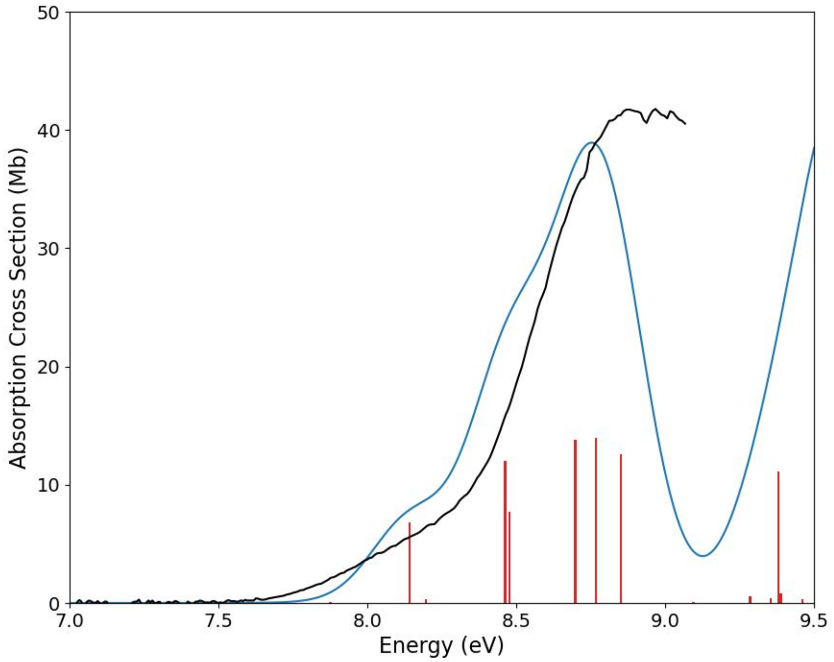

TD-PBE0/d-aug-cc-pVTZ for Propane

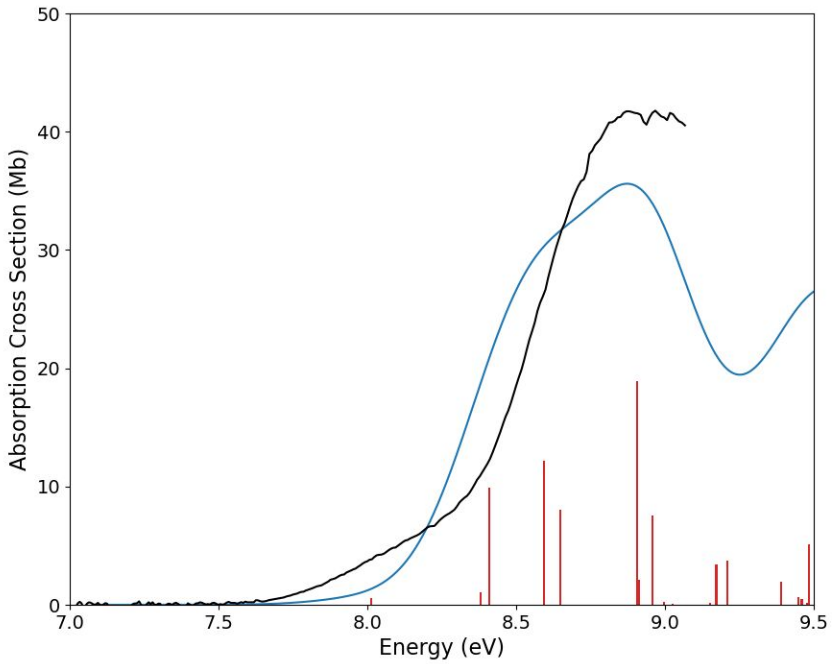

TD-wB97x-D/d-aug-cc-pVTZ for Propane

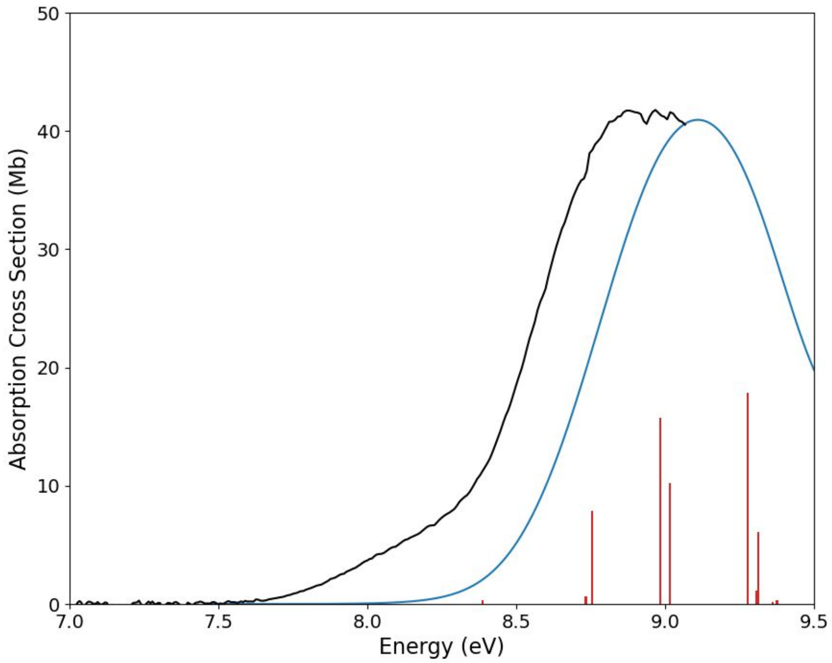

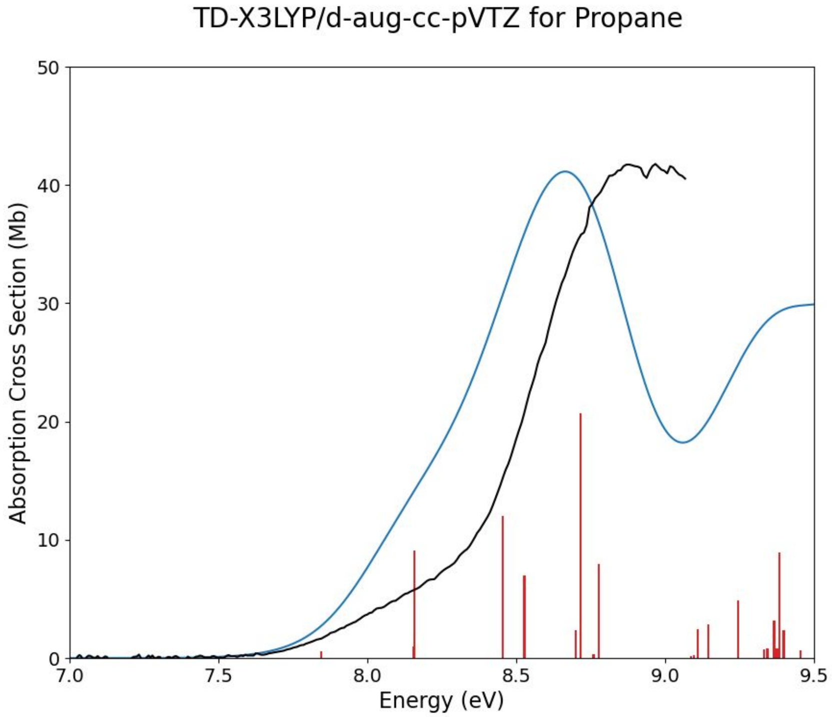

4 n-Butane

Table S13. Optimized Geometry of n-Butane in Å

	CCSD(T)/d-aug-cc-pVTZ			M06-2X/d-aug-cc-pVTZ		
C	0.0000000000	-0.5155399791	0.5647527512	-0.4204358510	0.6353402391	0.0000000000
C	0.0000000000	0.5155600423	0.5647097869	0.4204358510	-0.6353402391	0.0000000000
C	0.0000000000	0.1315024436	1.9503604313	0.4272090235	1.9006097484	0.0000000000
C	0.0000000000	-0.1315211617	1.9504005152	-0.4272090235	-1.9006097484	0.0000000000
H	0.8782966731	-1.1635220504	-0.4588143772	-1.0763940438	0.6293020069	0.8745043792
H	-0.8782966731	-1.1635220504	-0.4588143772	-1.0763940438	0.6293020069	-0.8745043792
H	0.8782966731	1.1635421136	0.4587714129	1.0763940438	-0.6293020069	0.8745043792
H	-0.8782966731	1.1635421136	0.4587714129	1.0763940438	-0.6293020069	-0.8745043792
H	0.0000000000	-0.6191488968	-2.7447415557	-0.1891138716	2.7989130668	0.0000000000
H	0.0000000000	0.6191301786	2.7447816396	1.0706874169	1.9356250323	0.8802490609
H	-0.8835429752	0.7630039465	-2.0837729892	1.0706874169	1.9356250323	-0.8802490609
H	0.8835429752	0.7630039465	2.0837729892	0.1891138716	-2.7989130668	0.0000000000
H	-0.8835429752	-0.7630226646	2.0838130731	-1.0706874169	-1.9356250323	0.8802490609
H	0.8835429752	-0.7630226646	2.0838130731	-1.0706874169	-1.9356250323	-0.8802490609

Table S14. Frequencies of n-Butane in cm^{-1}

CCSD(T)/d-aug-cc-pVTZ	M06-2X/d-aug-cc-pVTZ
116.683	115.683
223.232	221.592
255.857	256.881
259.948	261.084
422.769	430.098
732.631	730.724
807.790	808.010
848.138	854.427
960.047	959.729
977.646	980.013
1030.126	1039.310
1080.278	1086.566
1175.691	1176.865
1213.230	1209.342
1292.304	1288.825
1322.129	1319.684
1334.661	1331.596
1398.937	1398.010
1412.697	1410.185
1414.846	1412.558
1491.134	1489.180
1497.184	1493.899
1506.370	1502.469
1507.928	1503.701
1511.319	1508.083
1516.620	1514.290
3008.921	3043.384
3016.298	3052.232
3020.922	3059.326
3021.755	3059.430
3035.800	3068.311
3057.731	3090.209
3091.281	3123.585
3094.819	3126.796
3098.997	3133.437
3099.726	3133.982

Table S15. Transition Energies of n-Butane in eV

CCSD(T)/d-aug-cc-pVTZ	M06-2X/d-aug-cc-pVTZ
8.647	8.178
8.804	8.192
9.057	8.577
9.126	8.639
9.221	8.646
9.241	8.697
9.277	8.735
9.445	8.763
9.466	8.779
9.492	8.997
9.569	9.039
9.601	9.084
9.782	9.182
9.904	9.251
9.947	9.267
9.987	9.348
10.000	9.417
	9.454
	9.463
	9.494
	9.514
	9.565
	9.601
	9.638
	9.660
	9.699
	9.721
	9.741
	9.752
	9.778
	9.785
	9.836
	9.845
	9.866
	9.873
	9.878
	9.889
	9.897
	9.920
	9.930

Table S16. Quantitative Metrics for the Bandwidth (γ), cosine similarity (S), relative integral change (RIC), mean signed error (MSE), and mean average error (MAE) for the band shape of n-butane compared to experiment.

Method	γ	S	RIC	MSE	MAE
B3LYP	0.3	0.988	3.354	15.659	15.659
BH&HLYP	0.45	0.985	0.750	-3.578	3.578
BMK	0.3	0.983	0.708	-3.202	3.202
CAM-B3LYP	0.45	0.994	0.212	0.749	0.956
CC	0.5	0.990	0.787	-3.701	3.701
HSE	0.3	0.989	1.067	4.799	4.864
M06-2X	0.45	0.994	0.260	1.072	1.176
M11	0.3	0.991	1.339	6.401	6.401
PBE0	0.4	0.989	1.713	8.102	8.102
wB97x-D	0.45	0.994	0.130	-0.569	0.590
X3LYP	0.4	0.976	3.616	16.850	16.850

Figure S4. Spectra of n-Butane

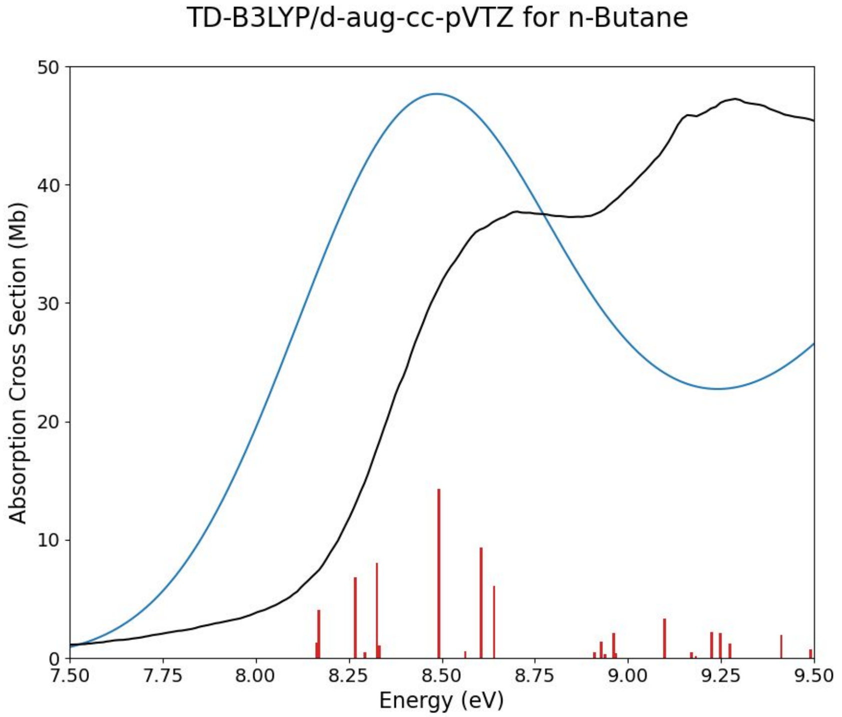

TD-BH&HLYP/d-aug-cc-pVTZ for n-Butane

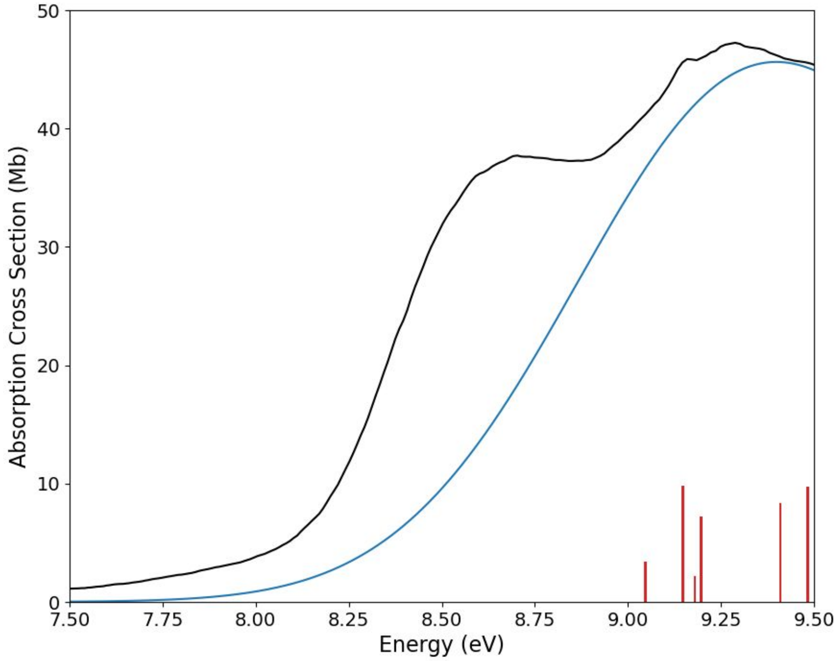

TD-BMK/d-aug-cc-pVTZ for n-Butane

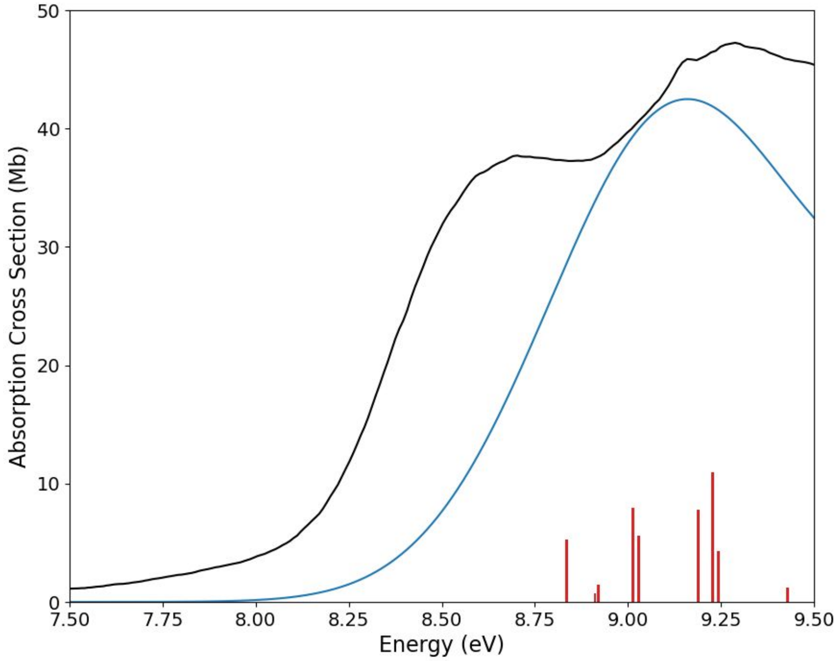

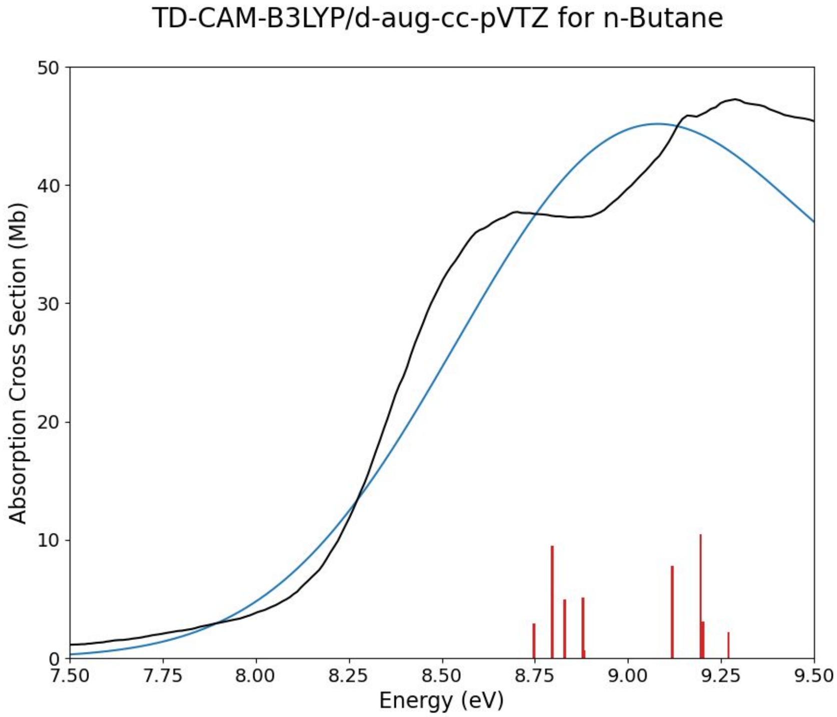
TD-CAM-B3LYP/d-aug-cc-pVTZ for n-Butane
Absorption Cross Section (Mb)
Energy (eV)
0
10
20
30
40
50
7.50
7.75
8.00
8.25
8.50
8.75
9.00
9.25
9.50

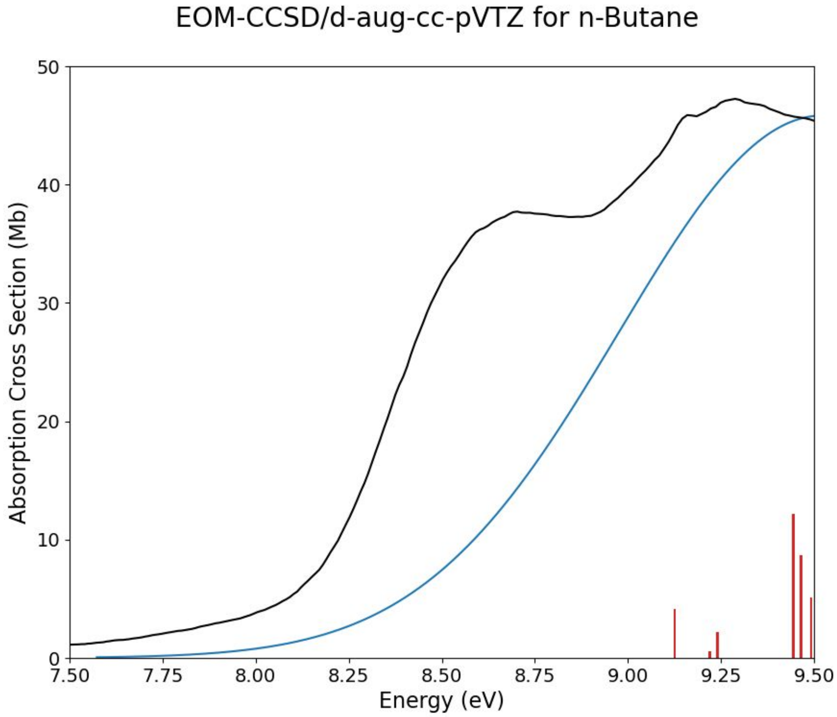
EOM-CCSD/d-aug-cc-pVTZ for n-Butane
Absorption Cross Section (Mb)
Energy (eV)
0
10
20
30
40
50
7.50
7.75
8.00
8.25
8.50
8.75
9.00
9.25
9.50

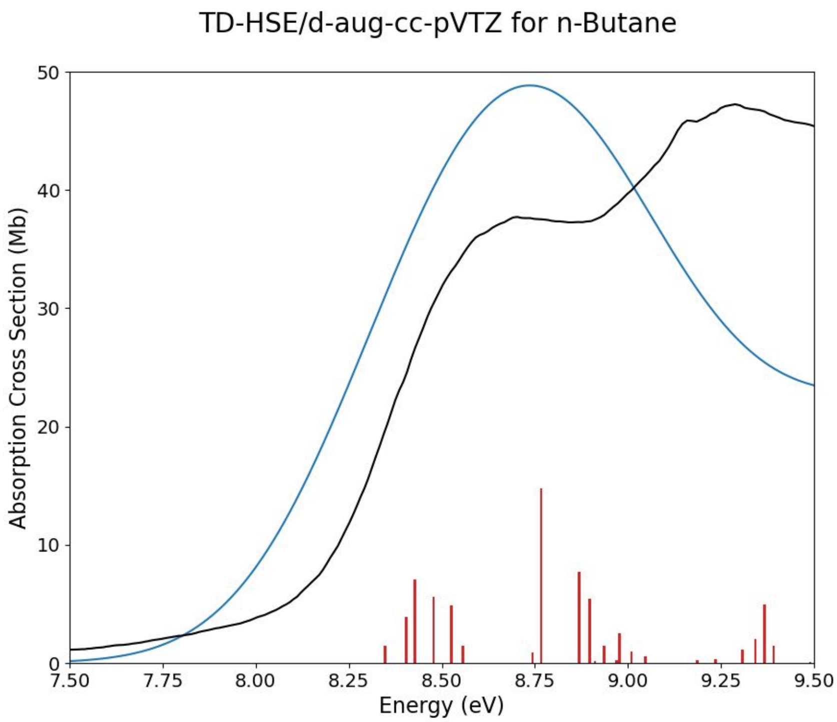
TD-HSE/d-aug-cc-pVTZ for n-Butane
Absorption Cross Section (Mb)
Energy (eV)
0
10
20
30
40
50
7.50
7.75
8.00
8.25
8.50
8.75
9.00
9.25
9.50

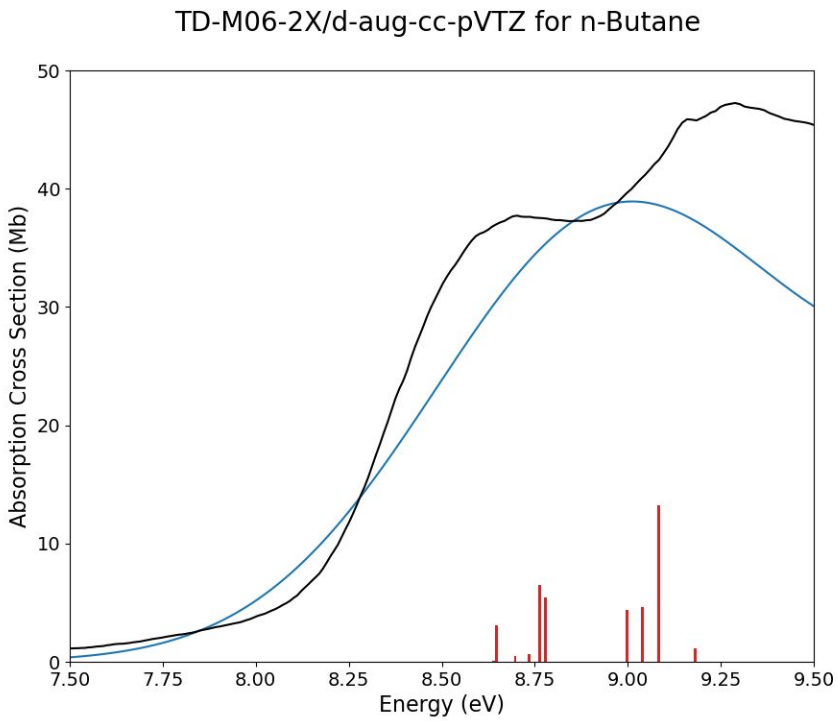
TD-M06-2X/d-aug-cc-pVTZ for n-Butane
Absorption Cross Section (Mb)
Energy (eV)
0
10
20
30
40
50
7.50
7.75
8.00
8.25
8.50
8.75
9.00
9.25
9.50

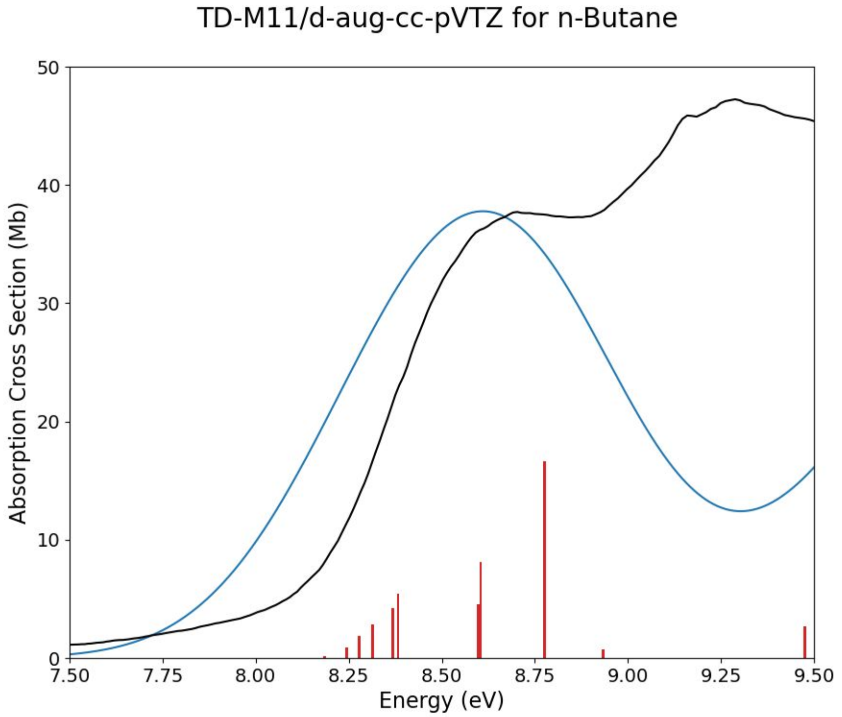

TD-M11/d-aug-cc-pVTZ for n-Butane
Absorption Cross Section (Mb)
Energy (eV)
0
10
20
30
40
50
7.50
7.75
8.00
8.25
8.50
8.75
9.00
9.25
9.50

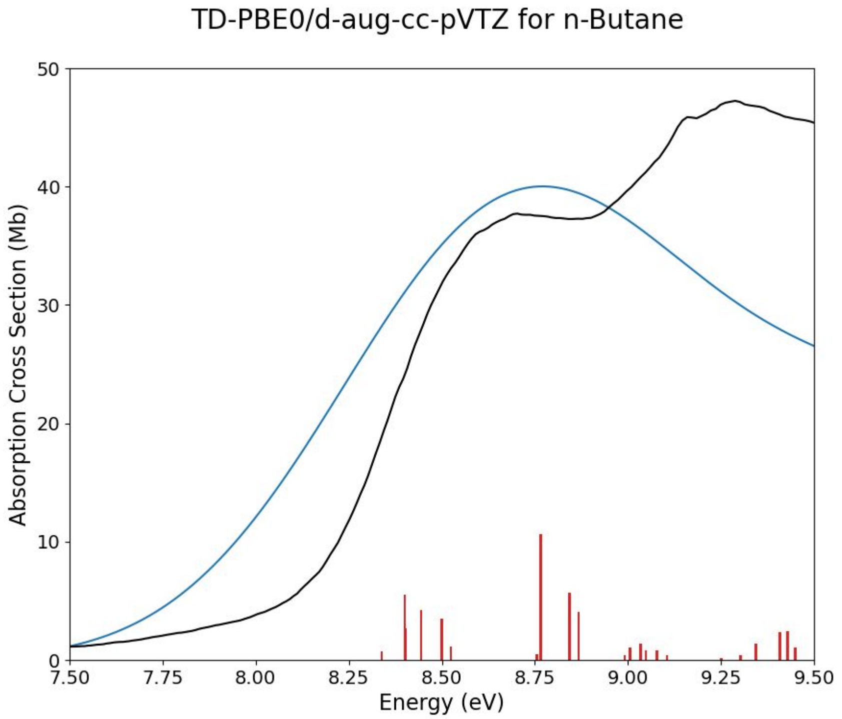

TD-PBE0/d-aug-cc-pVTZ for n-Butane
Absorption Cross Section (Mb)
Energy (eV)
0
10
20
30
40
50
7.50
7.75
8.00
8.25
8.50
8.75
9.00
9.25
9.50

TD-wB97x-D/d-aug-cc-pVTZ for n-Butane

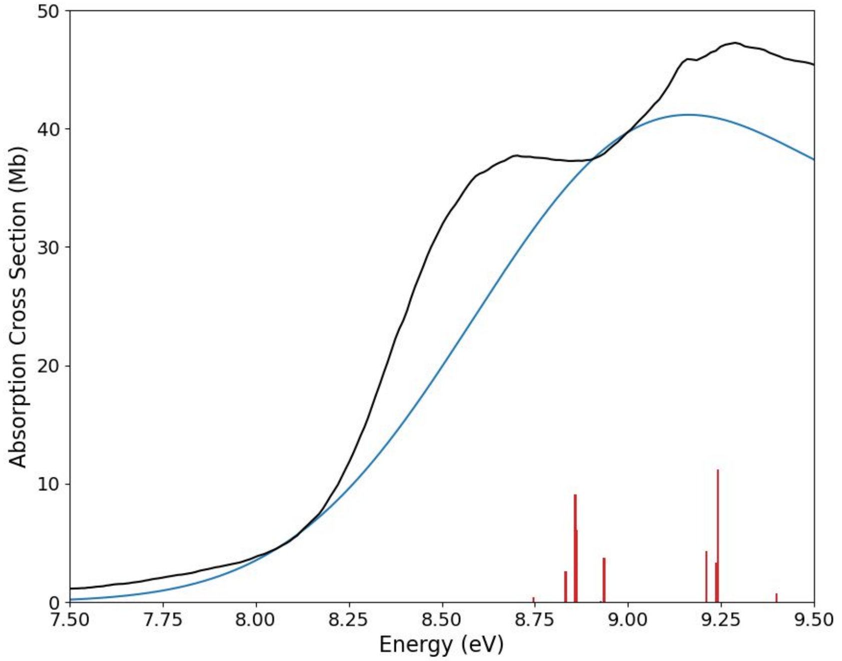

TD-X3LYP/d-aug-cc-pVTZ for n-Butane

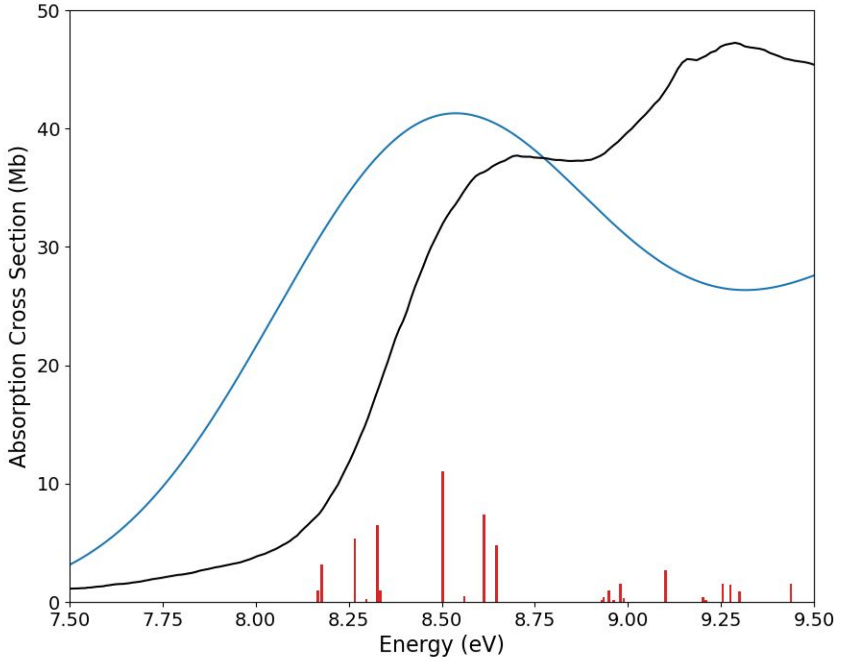

5 Ethene

Table S17. Optimized Geometry of Ethene in Å

	CCSD(T)/d-aug-cc-pVTZ			M06-2X/d-aug-cc-pVTZ		
C	0.0000000000	0.0000000000	-0.6690365244	0.0000000000	0.0000000000	0.6609834724
C	0.0000000000	0.0000000000	0.6690365244	0.0000000000	0.0000000000	-0.6609834724
H	0.0000000000	0.9247264002	-1.2344552438	0.0000000000	0.9218197941	1.2277098885
H	0.0000000000	-0.9247264002	-1.2344552438	0.0000000000	-0.9218197941	1.2277098885
H	0.0000000000	-0.9247264002	1.2344552438	0.0000000000	-0.9218197941	-1.2277098885
H	0.0000000000	0.9247264002	1.2344552438	0.0000000000	0.9218197941	-1.2277098885

Table S18. Frequencies of Ethene in cm^{-1}

CCSD(T)/d-aug-cc-pVTZ	M06-2X/d-aug-cc-pVTZ
817.869	826.214
920.046	988.494
959.094	1001.895
990.595	1058.450
1236.363	1241.405
1363.922	1387.059
1471.260	1474.103
1664.404	1716.378
3131.133	3160.032
3150.353	3175.639
3211.616	3235.126
3239.658	3261.172

Table S19. Transition Energies of Ethene in eV

CCSD(T)/d-aug-cc-pVTZ	M06-2X/d-aug-cc-pVTZ
7.401	6.987
7.983	7.497
8.047	7.585
8.105	7.592
8.450	7.972
8.536	8.001
8.890	8.336
	8.494
	8.604
	8.683
	8.701
	8.726
	8.743
	8.828
	9.038

Table S20. Quantitative Metrics for the Bandwidth (γ), cosine similarity (S), relative integral change (RIC), mean signed error (MSE), and mean average error (MAE) for the band shape of ethene compared to experiment.

Method	γ	S	RIC	MSE	MAE
B3LYP	0.25	0.973	0.319	8.063	9.380
BH&HLYP	0.35	0.967	0.296	3.373	7.129
BMK	0.3	0.964	0.234	-1.940	6.609
CAM-B3LYP	0.3	0.968	0.292	4.502	7.625
CC	0.33	0.967	0.557	-15.822	15.861
HSE	0.3	0.966	0.250	0.908	6.841
M06-2X	0.3	0.966	0.250	0.695	6.732
M11	0.25	0.975	0.292	5.680	7.858
PBE0	0.3	0.966	0.247	0.836	6.762
wB97x-D	0.33	0.967	0.264	2.405	6.926
X3LYP	0.27	0.973	0.326	7.127	8.542

Figure S5. Spectra of Ethene

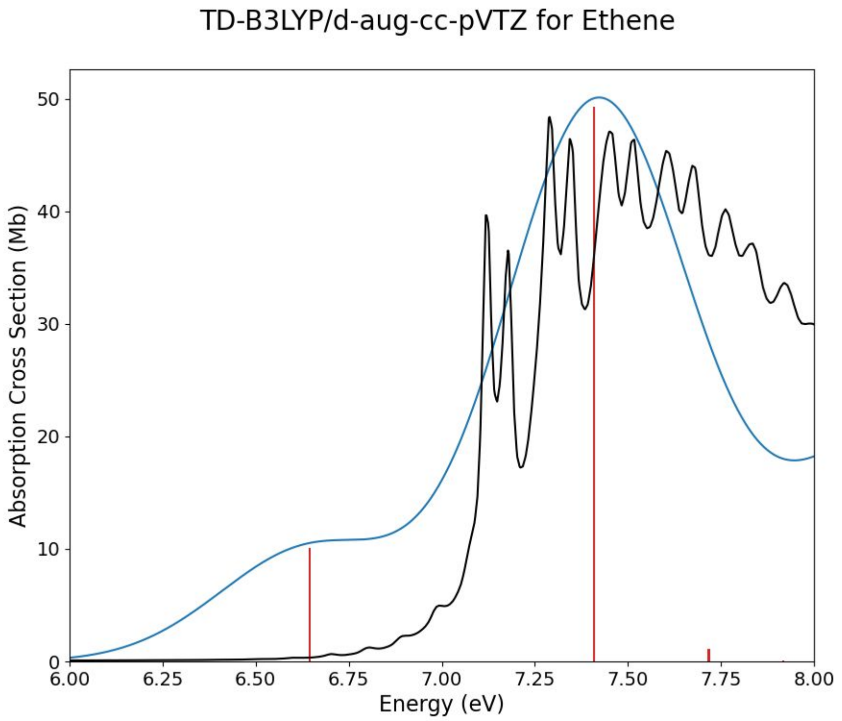

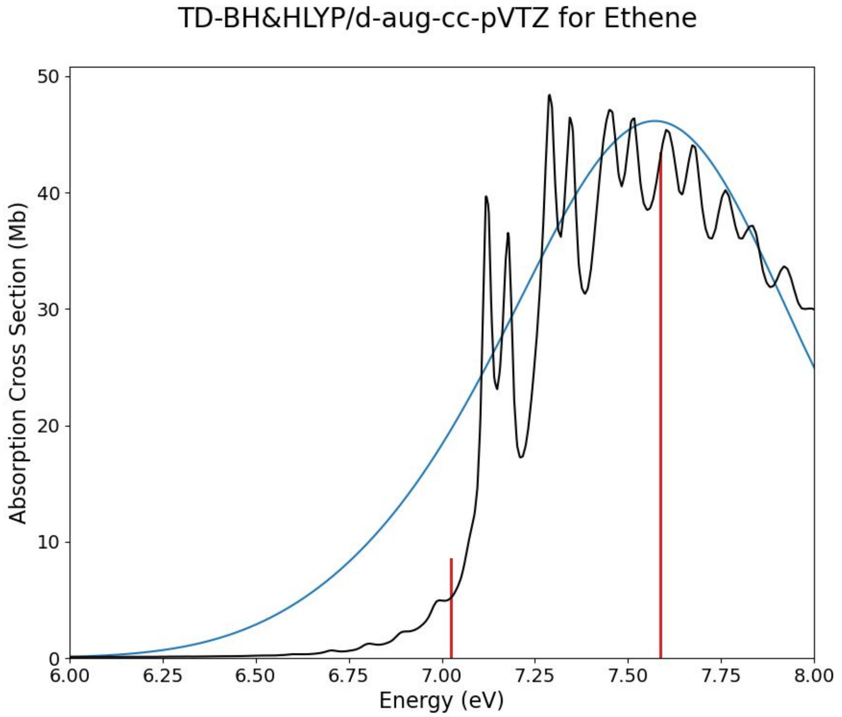
TD-BH&HLYP/d-aug-cc-pVTZ for Ethene
Absorption Cross Section (Mb)
0
10
20
30
40
50
6.00
6.25
6.50
6.75
7.00
7.25
7.50
7.75
8.00
Energy (eV)

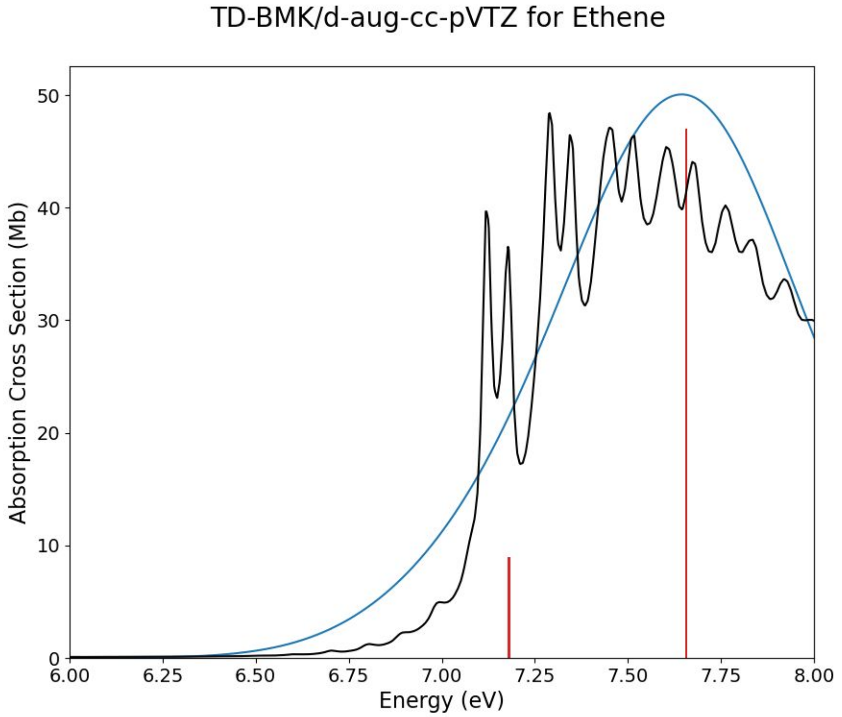
TD-BMK/d-aug-cc-pVTZ for Ethene
Absorption Cross Section (Mb)
0
10
20
30
40
50
6.00
6.25
6.50
6.75
7.00
7.25
7.50
7.75
8.00
Energy (eV)

TD-CAM-B3LYP/d-aug-cc-pVTZ for Ethene

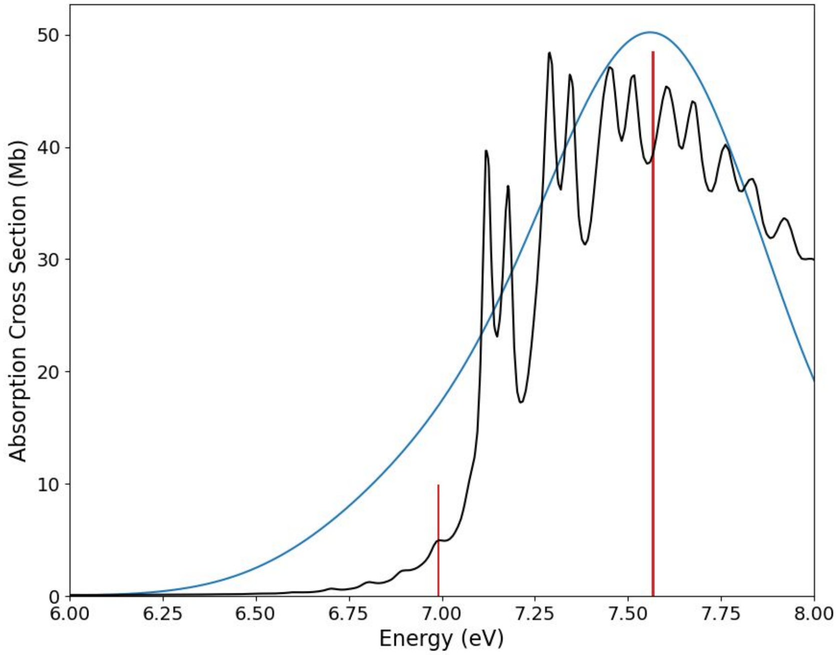

EOM-CCSD/d-aug-cc-pVTZ for Ethene

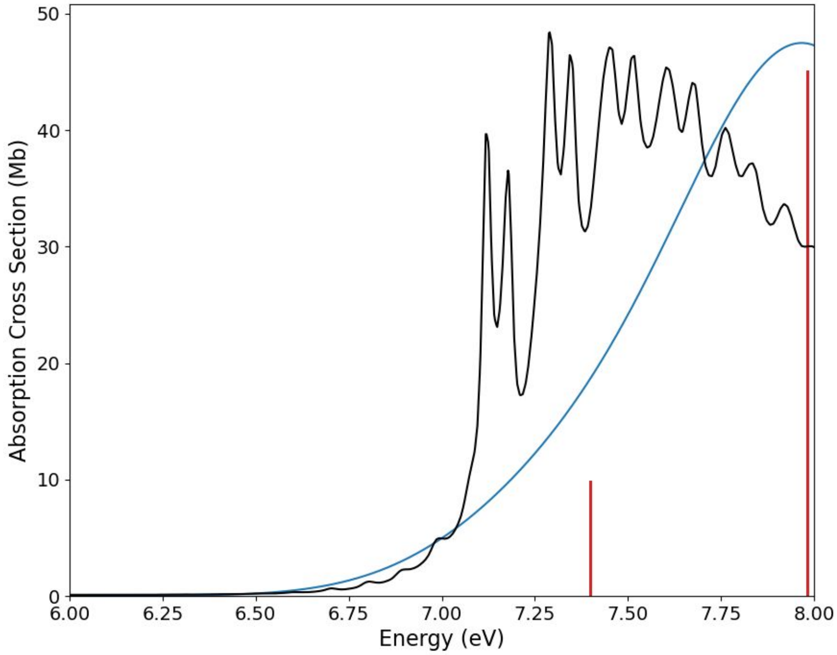

TD-HSE/d-aug-cc-pVTZ for Ethene

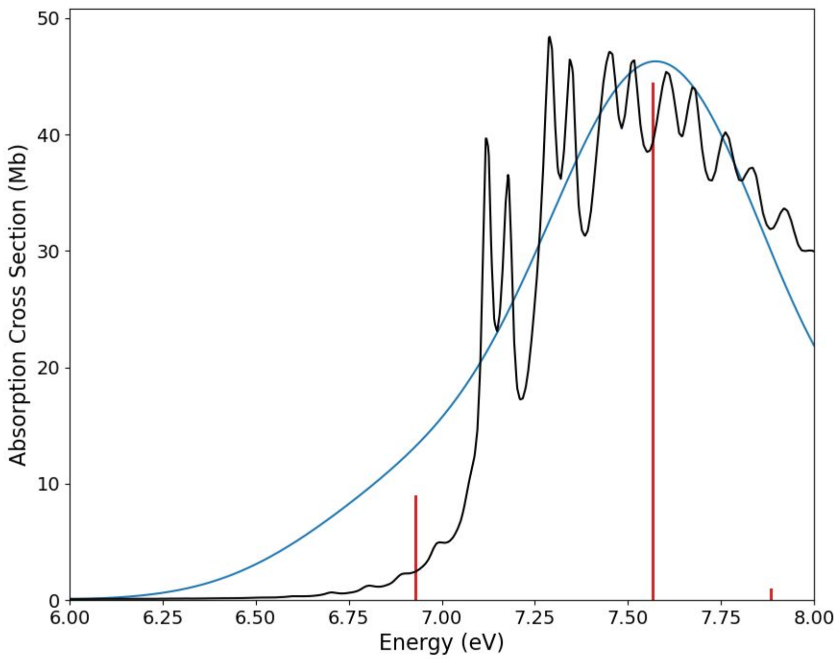

TD-M06-2X/d-aug-cc-pVTZ for Ethene

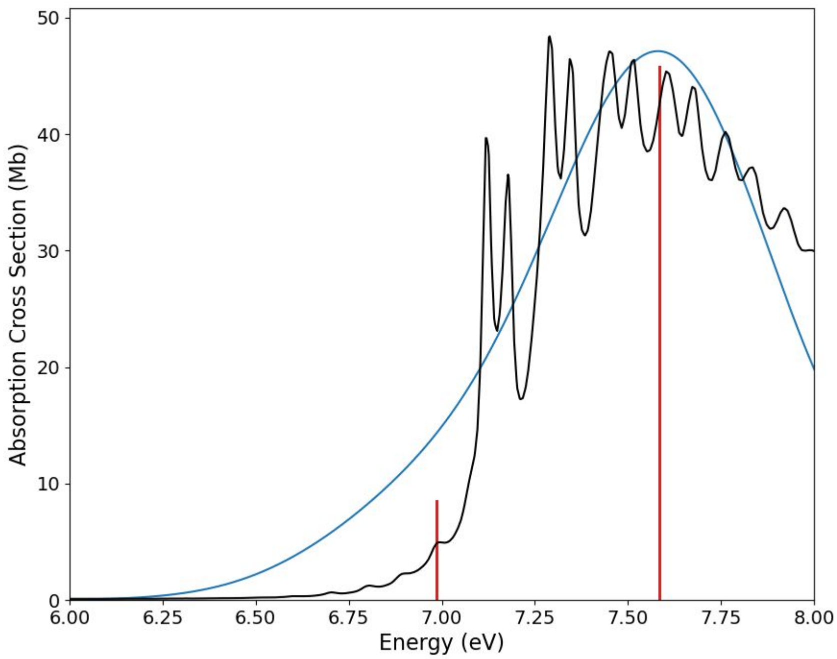

TD-M11/d-aug-cc-pVTZ for Ethene

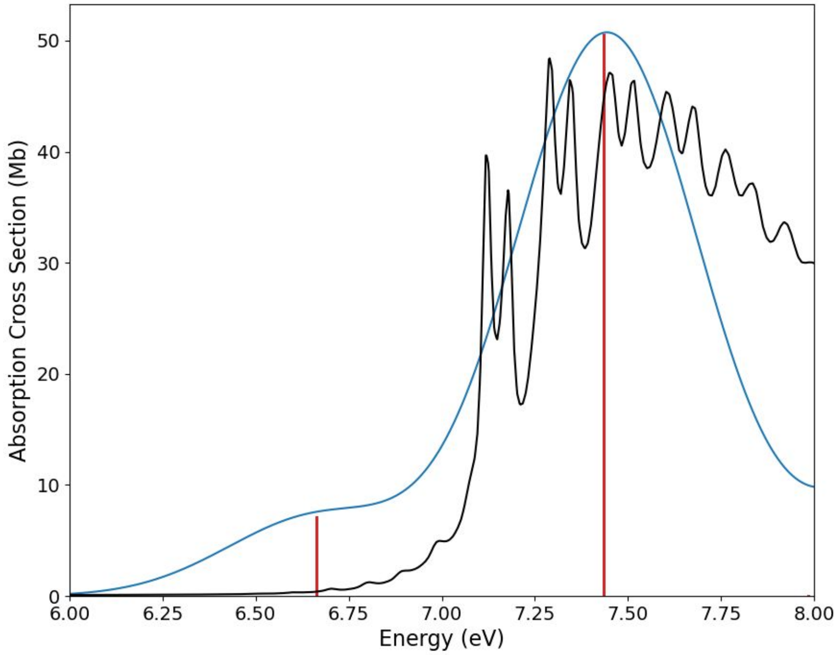

TD-PBE0/d-aug-cc-pVTZ for Ethene

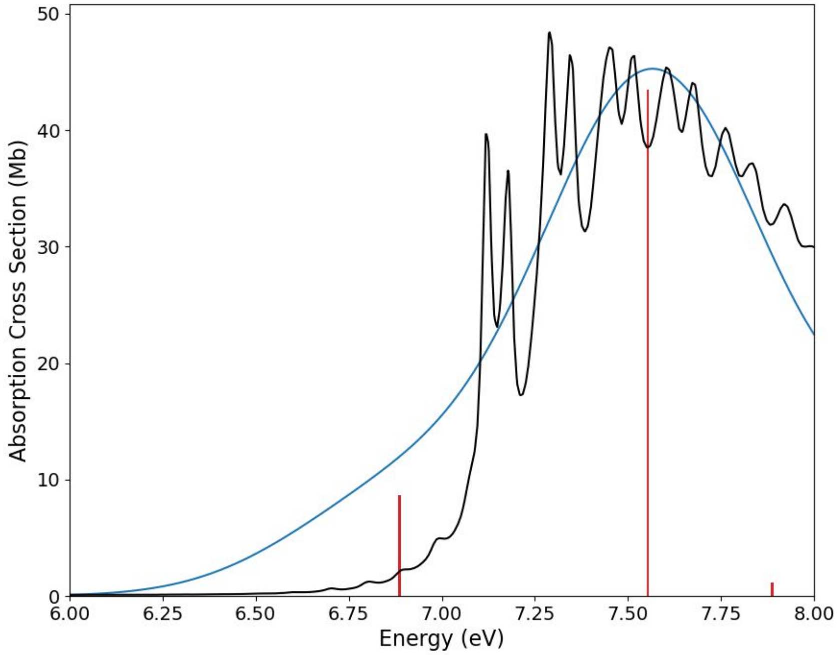

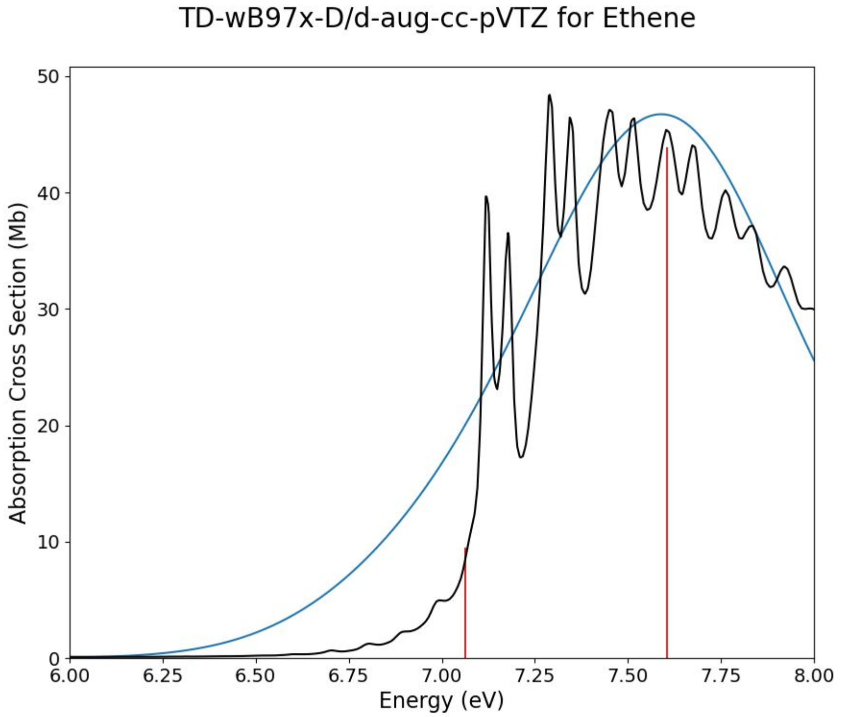
TD-wB97x-D/d-aug-cc-pVTZ for Ethene
Absorption Cross Section (Mb)
Energy (eV)
0
10
20
30
40
50
6.00
6.25
6.50
6.75
7.00
7.25
7.50
7.75
8.00

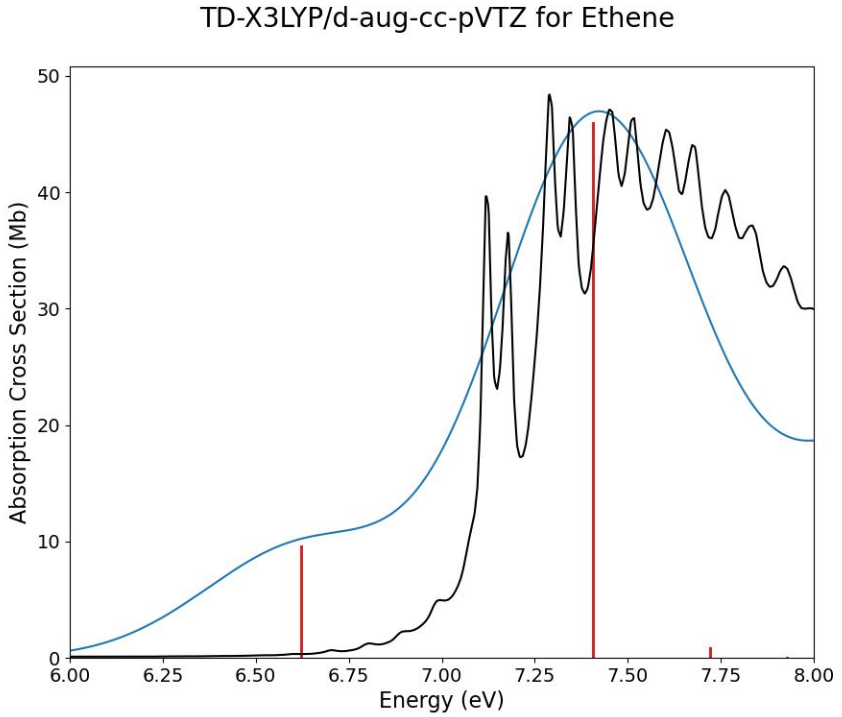
TD-X3LYP/d-aug-cc-pVTZ for Ethene
Absorption Cross Section (Mb)
Energy (eV)
0
10
20
30
40
50
6.00
6.25
6.50
6.75
7.00
7.25
7.50
7.75
8.00

6 Propene

Table S21. Optimized Geometry of Propene in Å

	CCSD(T)/d-aug-cc-pVTZ			M06-2X/d-aug-cc-pVTZ		
C	0.0000000000	0.2302283407	-1.2566225016	-1.1418870914	-0.5431850756	0.0000000000
C	0.0000000000	-0.4522472697	-0.1047013776	-0.0306662596	0.4577278350	0.0000000000
C	0.0000000000	0.1793062510	1.2584715127	1.2584328098	0.1561220477	0.0000000000
H	0.0000000000	-0.2793730643	-2.2129958041	1.5939235462	-0.8742996252	0.0000000000
H	0.0000000000	1.3158127337	-1.2690990173	2.0201686470	0.9235834815	0.0000000000
H	0.0000000000	-1.5393254973	-0.1387782612	-0.3242588334	1.5029244612	0.0000000000
H	0.0000000000	1.2690495107	1.1845419190	-0.7536076747	-1.5605107722	0.0000000000
H	0.8800456542	-0.1285915065	1.8309797249	-1.7793888442	-0.4165456490	0.8765484553
H	-0.8800456542	-0.1285915065	1.8309797249	-1.7793888442	-0.4165456490	-0.8765484553

Table S22. Frequencies of Propene in cm^{-1}

CCSD(T)/d-aug-cc-pVTZ	M06-2X/d-aug-cc-pVTZ
203.264	198.715
417.050	430.105
579.652	595.815
920.078	938.708
927.799	946.569
940.895	966.802
1010.597	1034.460
1069.536	1079.714
1190.948	1195.104
1317.723	1329.672
1409.696	1406.760
1451.282	1453.144
1490.517	1484.509
1503.550	1497.244
1689.455	1741.724
3023.598	3058.416
3084.684	3113.046
3107.253	3139.761
3129.536	3158.723
3143.333	3168.381
3222.730	3247.943

Table S23. Transition Energies of Propene in eV

CCSD(T)/d-aug-cc-pVTZ	M06-2X/d-aug-cc-pVTZ
6.912	6.507
7.471	6.981
7.544	7.120

7.635	7.129
7.947	7.493
8.315	7.769
8.400	7.863
8.429	7.908
8.531	8.000
8.609	8.070
8.645	8.107
8.675	8.158
8.798	8.194
8.861	8.223
8.933	8.296
	8.420
	8.456
	8.579
	8.664
	8.729
	8.869
	8.904
	8.913
	8.939
	8.959
	8.965
	8.983
	9.019

Table S24. Quantitative Metrics for the Bandwidth (γ), cosine similarity (S), relative integral change (RIC), mean signed error (MSE), and mean average error (MAE) for the band shape of propene compared to experiment.

Method	γ	S	RIC	MSE	MAE
B3LYP	0.23	0.989	0.128	1.556	4.223
BH&HLYP	0.33	0.996	0.108	-2.827	3.466
BMK	0.35	0.993	0.224	-7.059	7.060
CAM-B3LYP	0.35	0.999	0.057	-1.227	1.760
CC	0.32	0.961	0.624	-19.944	19.944
HSE	0.3	0.999	0.036	0.453	1.207
M06-2X	0.3	0.998	0.081	-2.452	2.599
M11	0.17	0.994	0.186	0.490	3.930
PBE0	0.27	0.999	0.035	0.144	1.122
wB97x-D	0.35	0.998	0.106	-3.316	3.394
X3LYP	0.17	0.986	0.127	3.340	4.260

Figure S6. Spectra of Propene

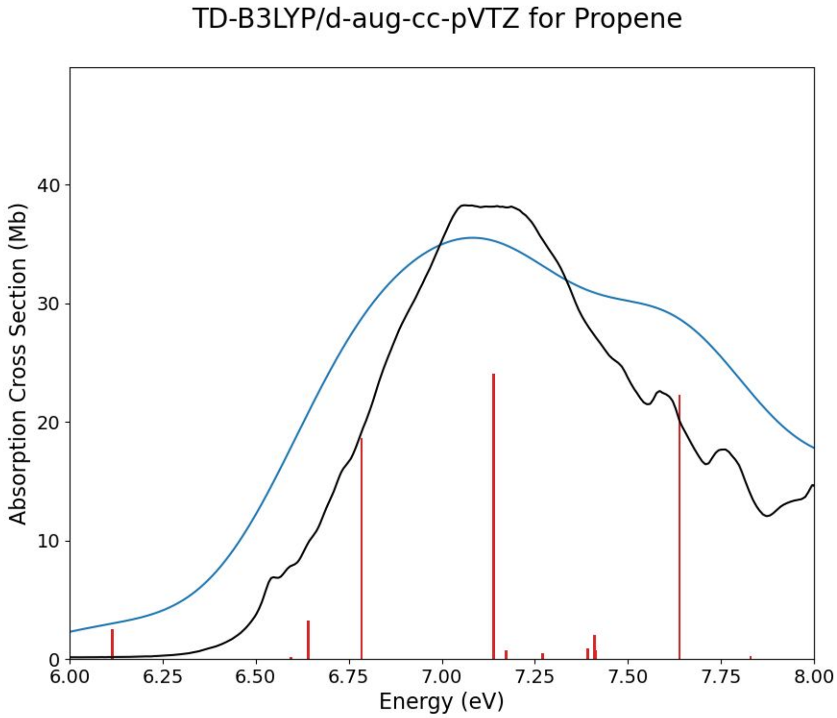

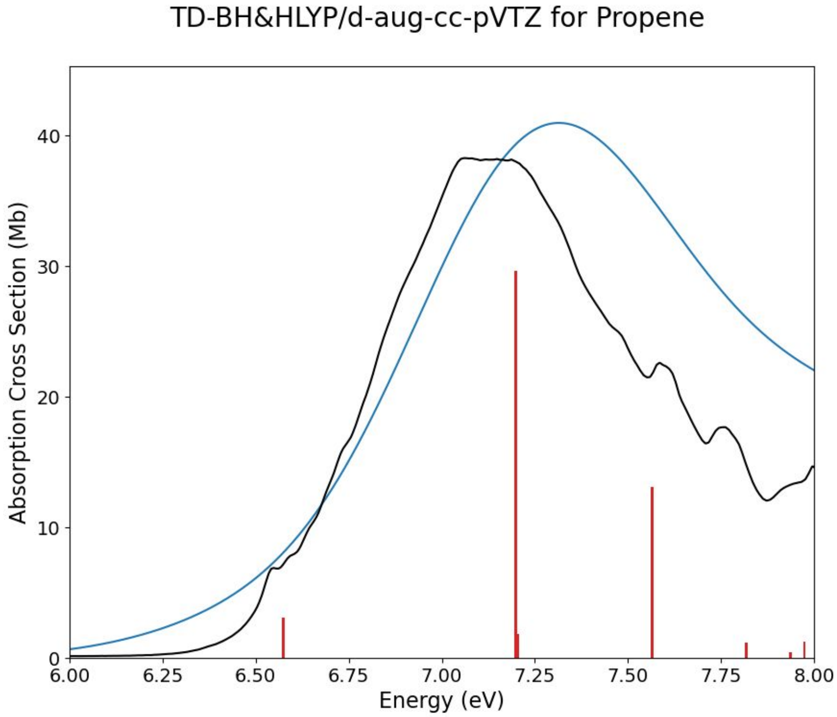

TD-BMK/d-aug-cc-pVTZ for Propene

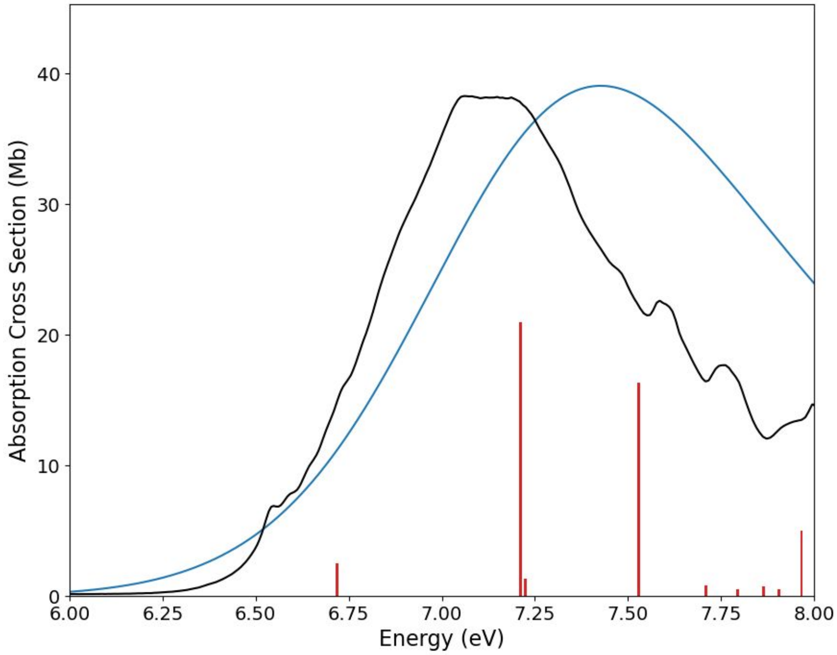

TD-CAM-B3LYP/d-aug-cc-pVTZ for Propene

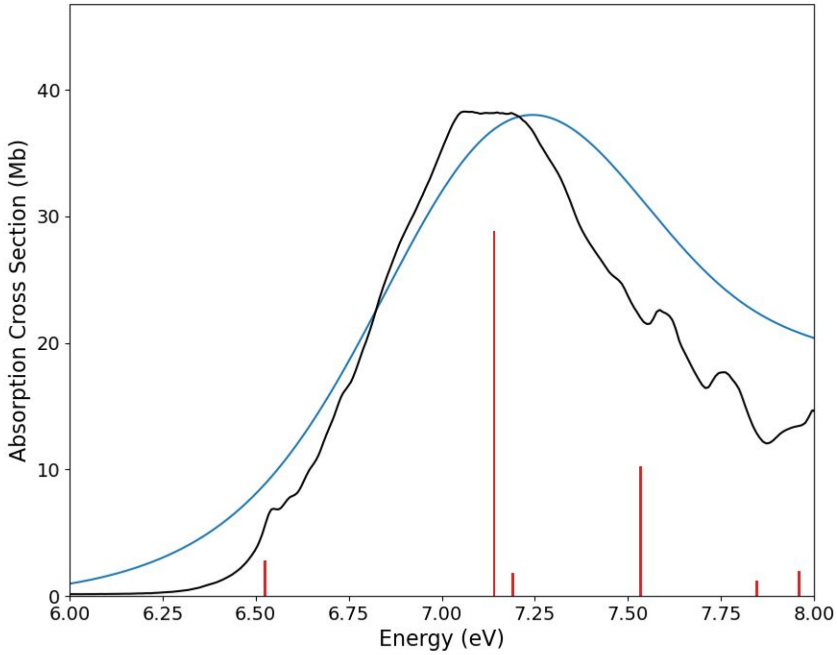

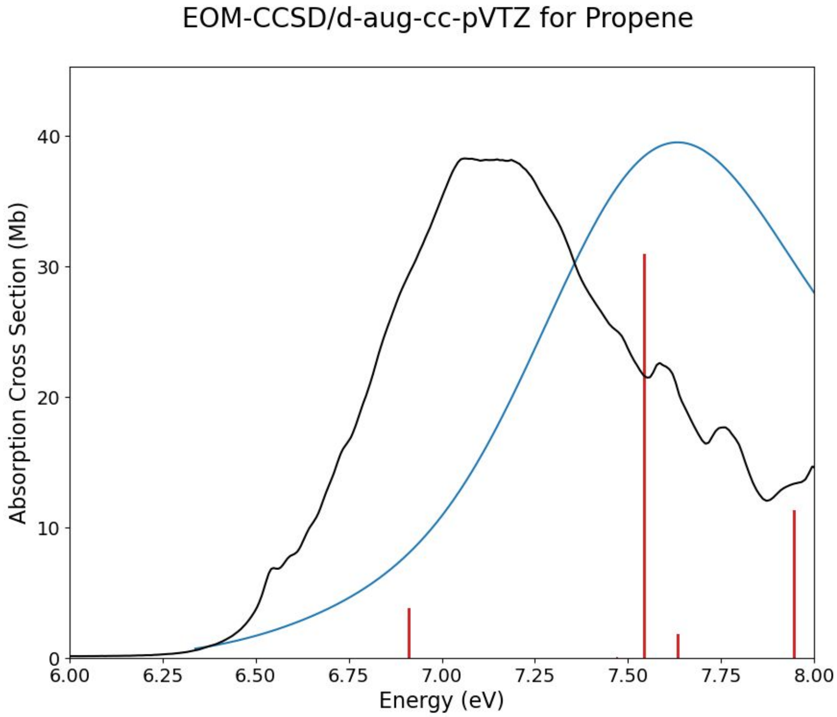
EOM-CCSD/d-aug-cc-pVTZ for Propene
Absorption Cross Section (Mb)
Energy (eV)
0
10
20
30
40
6.00
6.25
6.50
6.75
7.00
7.25
7.50
7.75
8.00

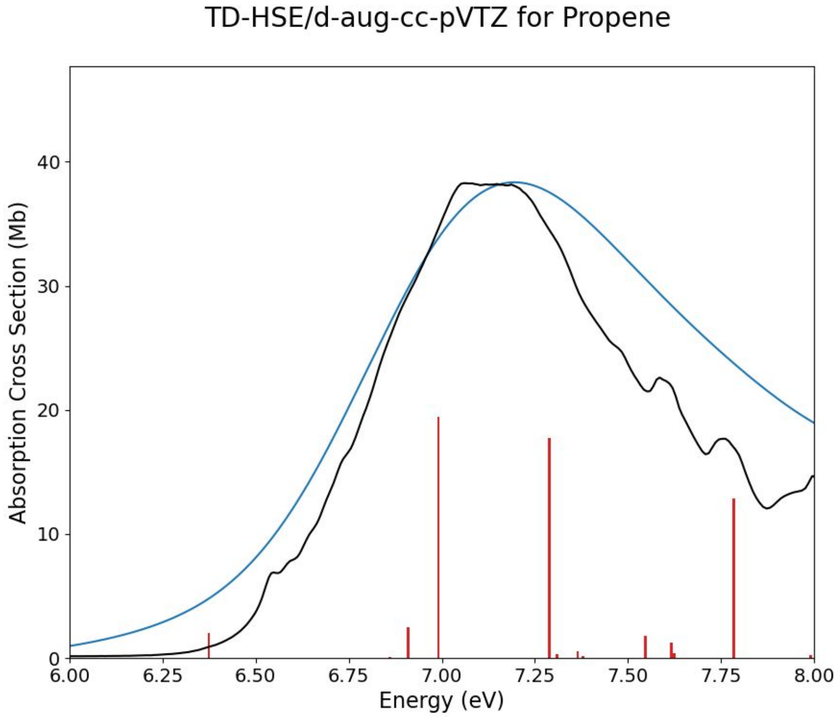
TD-HSE/d-aug-cc-pVTZ for Propene
Absorption Cross Section (Mb)
Energy (eV)
0
10
20
30
40
6.00
6.25
6.50
6.75
7.00
7.25
7.50
7.75
8.00

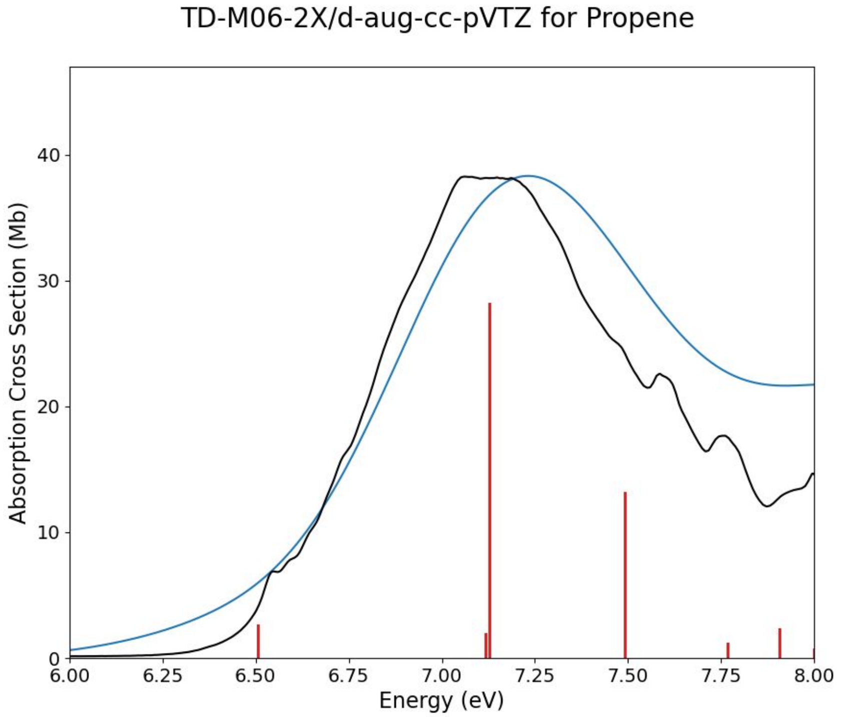
TD-M06-2X/d-aug-cc-pVTZ for Propene
Absorption Cross Section (Mb)
Energy (eV)
0
10
20
30
40
6.00
6.25
6.50
6.75
7.00
7.25
7.50
7.75
8.00

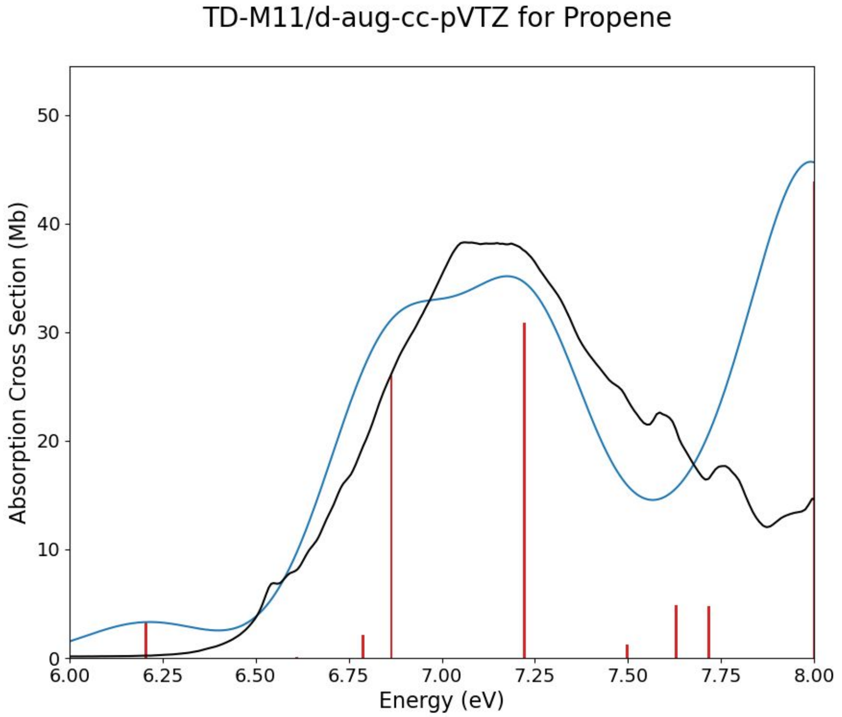
TD-M11/d-aug-cc-pVTZ for Propene
Absorption Cross Section (Mb)
Energy (eV)
0
10
20
30
40
50
6.00
6.25
6.50
6.75
7.00
7.25
7.50
7.75
8.00

TD-PBE0/d-aug-cc-pVTZ for Propene

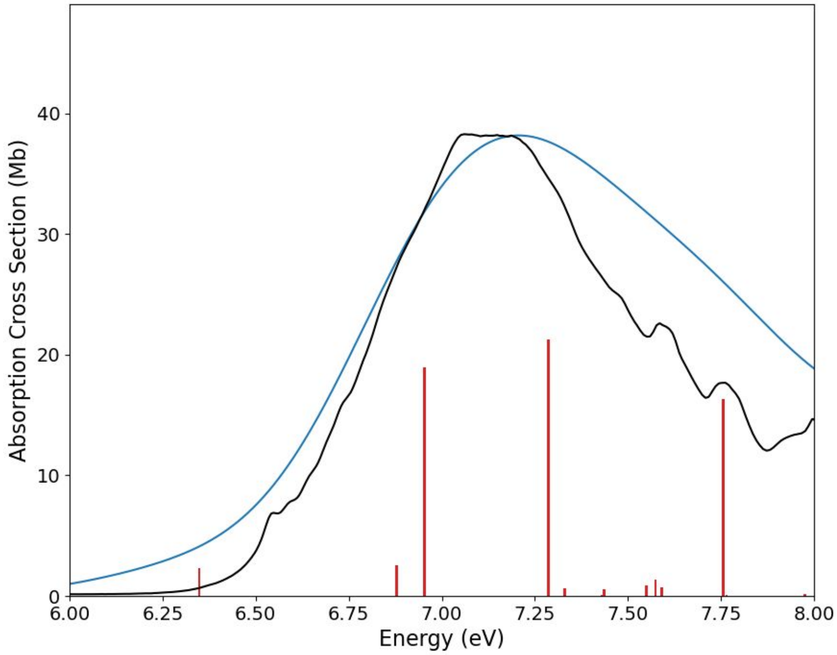

TD-wB97x-D/d-aug-cc-pVTZ for Propene

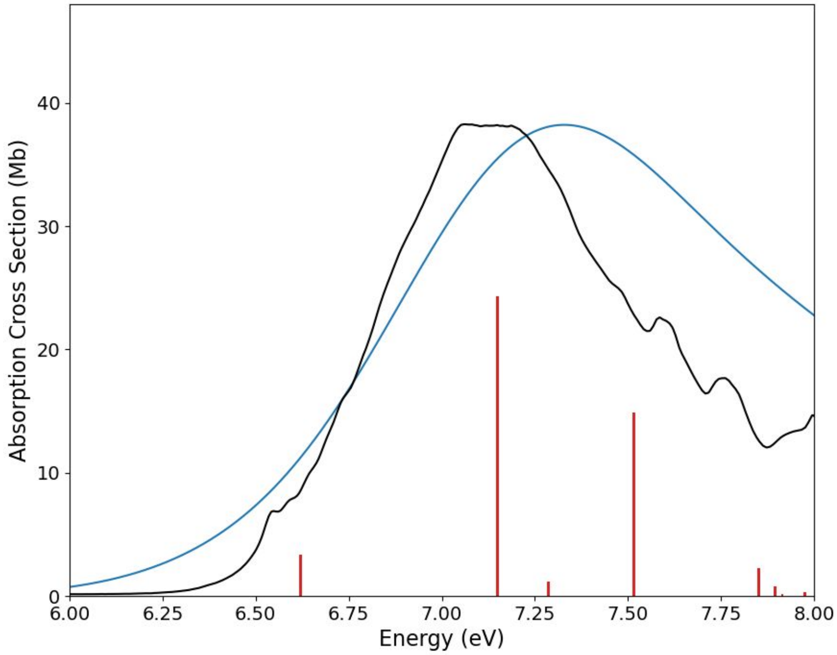

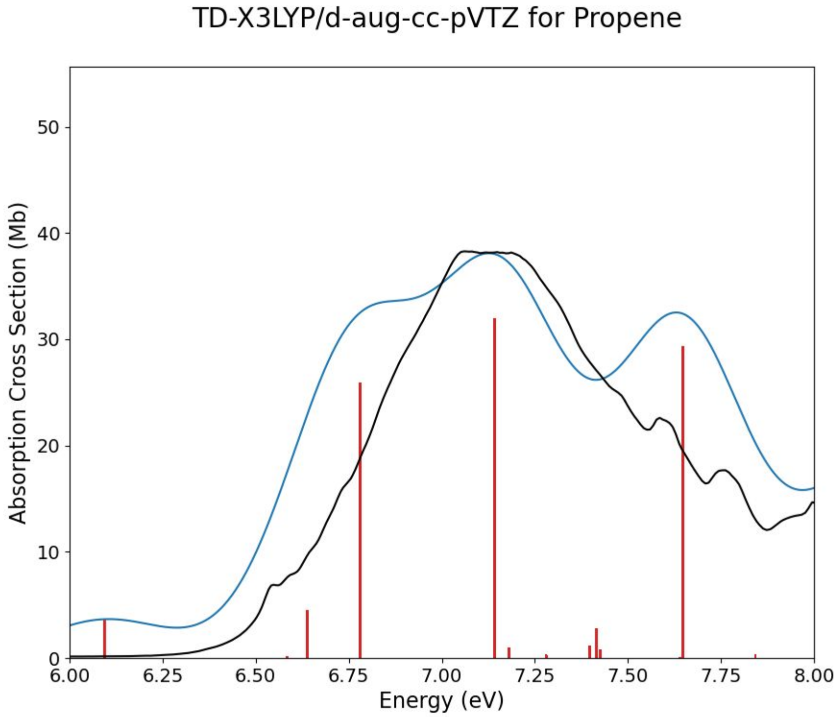
TD-X3LYP/d-aug-cc-pVTZ for Propene
Absorption Cross Section (Mb)
Energy (eV)
0
10
20
30
40
50
6.00
6.25
6.50
6.75
7.00
7.25
7.50
7.75
8.00

7 cis-Butene

Table S25. Optimized Geometry of cis-Butene in Å

	CCSD(T)/d-aug-cc-pVTZ			M06-2X/d-aug-cc-pVTZ		
C	0.0000000000	-0.6712951983	-0.6394368722	0.0000000000	0.6641404325	0.6338350173
C	0.0000000000	0.6712951983	-0.6394368722	0.0000000000	-0.6641404325	0.6338350173
C	0.0000000000	-1.5825695440	0.5571670432	0.0000000000	1.5774537236	-0.5525993343
C	0.0000000000	1.5825695440	0.5571670432	0.0000000000	-1.5774537236	-0.5525993343
H	0.0000000000	-1.1688478040	-1.6066061595	0.0000000000	1.1606139812	1.5987881401
H	0.0000000000	1.1688478040	-1.6066061595	0.0000000000	-1.1606139812	1.5987881401
H	0.0000000000	-1.0354688588	1.5002905681	0.0000000000	1.0404940682	-1.4975409279
H	0.0000000000	1.0354688588	1.5002905681	0.0000000000	-1.0404940682	-1.4975409279
H	-0.8796304048	-2.2335440848	0.5433372294	-0.8759114398	2.2283097680	-0.5342532865
H	0.8796304048	-2.2335440848	0.5433372294	0.8759114398	2.2283097680	-0.5342532865
H	0.8796304048	2.2335440848	0.5433372294	0.8759114398	-2.2283097680	-0.5342532865
H	-0.8796304048	2.2335440848	0.5433372294	-0.8759114398	-2.2283097680	-0.5342532865

Table S26. Frequencies of cis-Butene in cm^{-1}

CCSD(T)/d-aug-cc-pVTZ	M06-2X/d-aug-cc-pVTZ
113.970	129.420
125.607	132.680
293.749	303.881
380.756	400.265
556.287	567.357
683.160	703.328
870.553	883.475
982.276	988.235
985.357	1018.230
1023.145	1031.608
1056.276	1066.855
1056.725	1074.939
1157.496	1160.743
1280.522	1292.393
1388.900	1388.032
1422.933	1419.628
1437.851	1443.276
1489.816	1484.870
1495.009	1490.427
1497.018	1494.395
1505.065	1503.004
1713.768	1765.183
3024.491	3060.300
3027.382	3063.147
3079.111	3108.678

3079.590	3108.900
3112.144	3145.323
3128.205	3161.703
3128.559	3163.768
3151.556	3181.480

Table S27. Transition Energies of cis-Butene in eV

CCSD(T)/d-aug-cc-pVTZ	M06-2X/d-aug-cc-pVTZ
6.392	5.998
6.990	6.521
7.178	6.668
7.212	6.766
7.748	7.278
7.787	7.280
7.795	7.348
7.880	7.360
7.891	7.373
7.902	7.419
8.057	7.586
8.225	7.697
8.323	7.749
8.325	7.773
8.513	7.954
8.518	7.959
8.566	7.968
8.591	7.984
8.599	8.006
8.620	8.073
8.627	8.105
8.658	8.139
8.980	8.159
8.998	8.294
9.017	8.381
9.053	8.381
	8.446
	8.485
	8.494
	8.526
	8.543
	8.637
	8.699
	8.808
	8.829
	8.838
	8.883
	8.903

	8.914<br>8.962<br>9.061<br>9.077

Table S28. Quantitative Metrics for the Bandwidth (γ), cosine similarity (S), relative integral change (RIC), mean signed error (MSE), and mean average error (MAE) for the band shape of cis-butene compared to experiment.

Method	γ	S	RIC	MSE	MAE
B3LYP	0.4	0.985	0.685	12.638	12.638
BH&HLYP	0.4	0.997	0.152	-2.738	2.796
BMK	0.4	0.998	0.204	-3.591	3.606
CAM-B3LYP	0.4	0.998	0.109	2.009	2.017
CC	0.5	0.996	0.641	-11.586	11.586
HSE	0.5	0.978	0.439	8.194	8.194
M06-2X	0.4	0.998	0.063	0.488	1.155
M11	0.35	0.999	0.180	3.233	3.233
PBE0	0.4	0.993	0.410	7.556	7.556
wB97x-D	0.4	0.998	0.061	-0.062	1.114
X3LYP	0.35	0.994	0.688	12.599	12.599

Figure S7. Spectra of cis-Butene

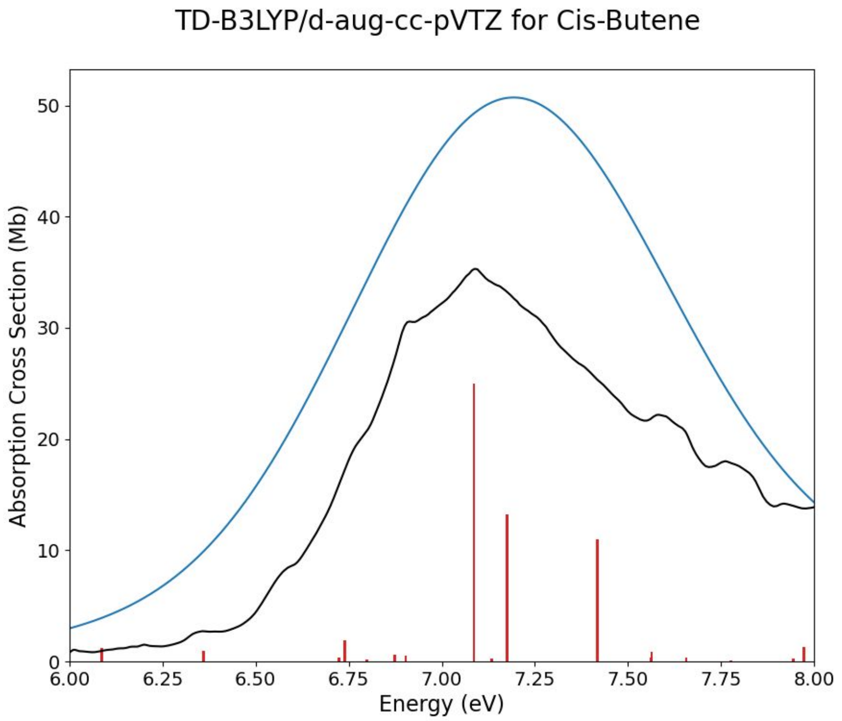

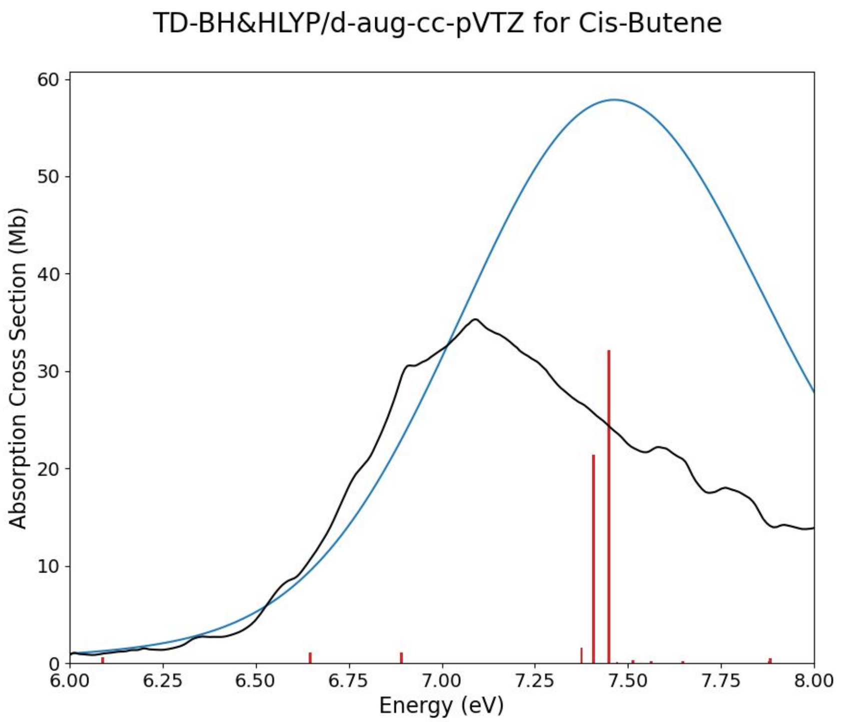
TD-BH&HLYP/d-aug-cc-pVTZ for Cis-Butene
Absorption Cross Section (Mb)
Energy (eV)
0
10
20
30
40
50
60
6.00
6.25
6.50
6.75
7.00
7.25
7.50
7.75
8.00

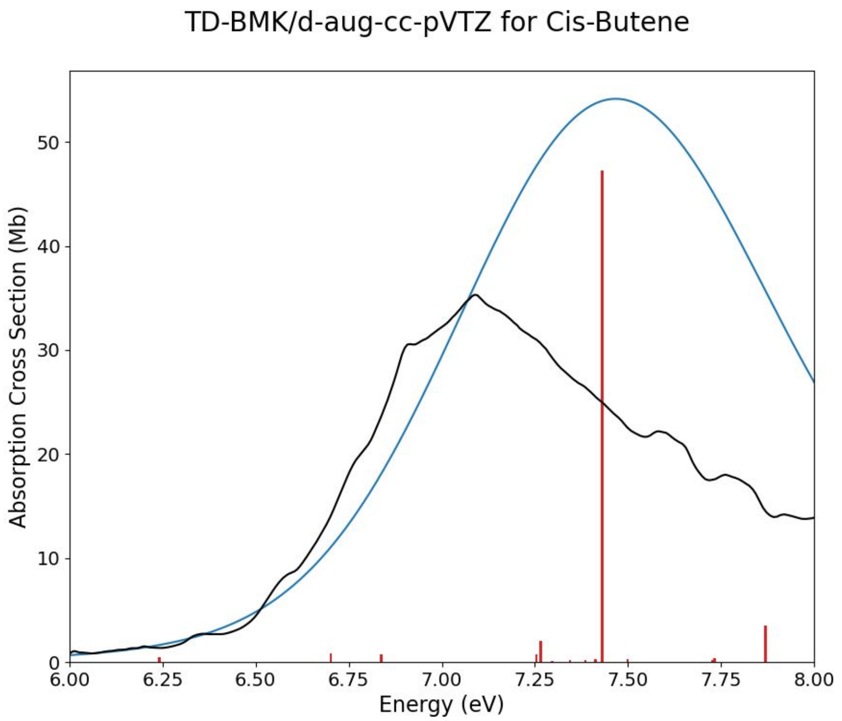
TD-BMK/d-aug-cc-pVTZ for Cis-Butene
Absorption Cross Section (Mb)
Energy (eV)
0
10
20
30
40
50
6.00
6.25
6.50
6.75
7.00
7.25
7.50
7.75
8.00

TD-CAM-B3LYP/d-aug-cc-pVTZ for Cis-Butene

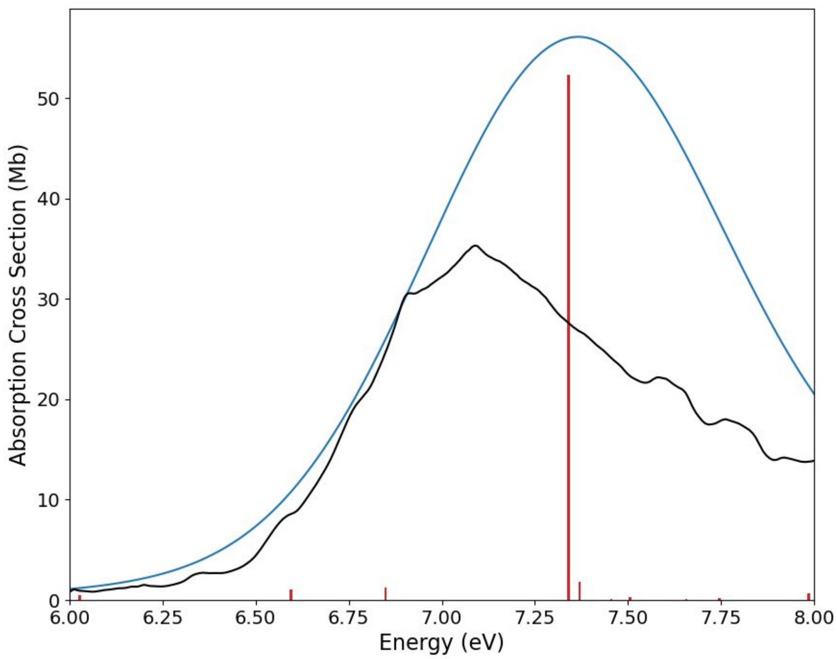

EOM-CCSD/d-aug-cc-pVTZ for Cis-Butene

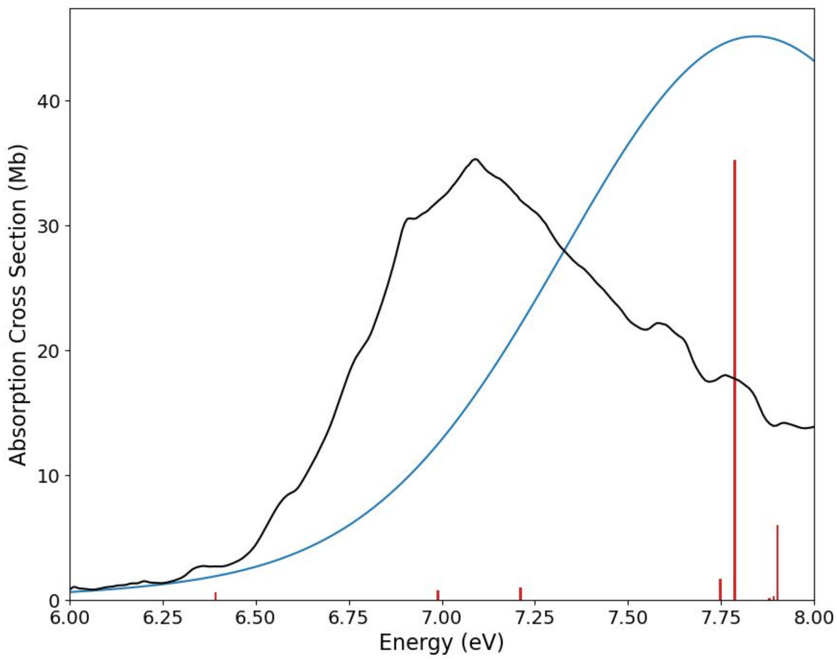

TD-HSE/d-aug-cc-pVTZ for Cis-Butene

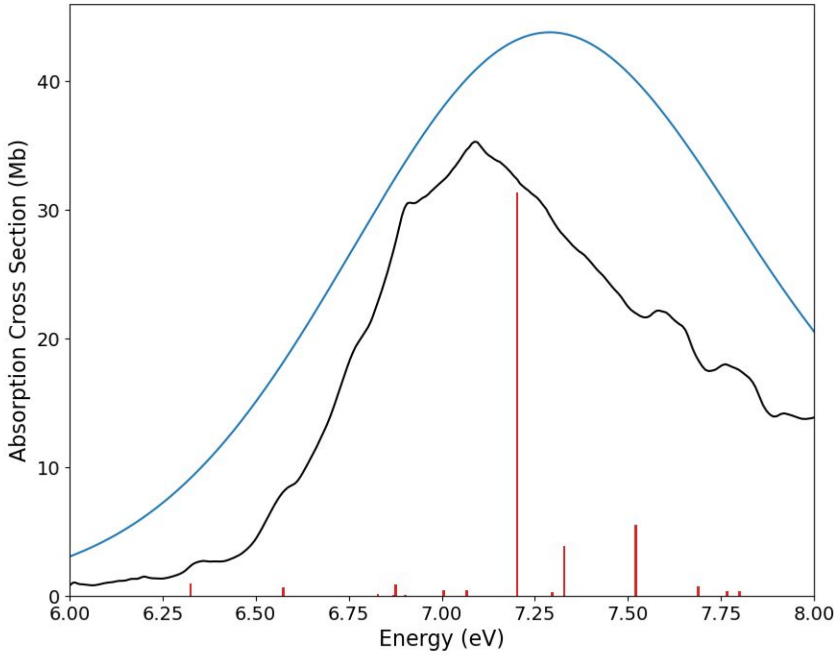

TD-M06-2X/d-aug-cc-pVTZ for Cis-Butene

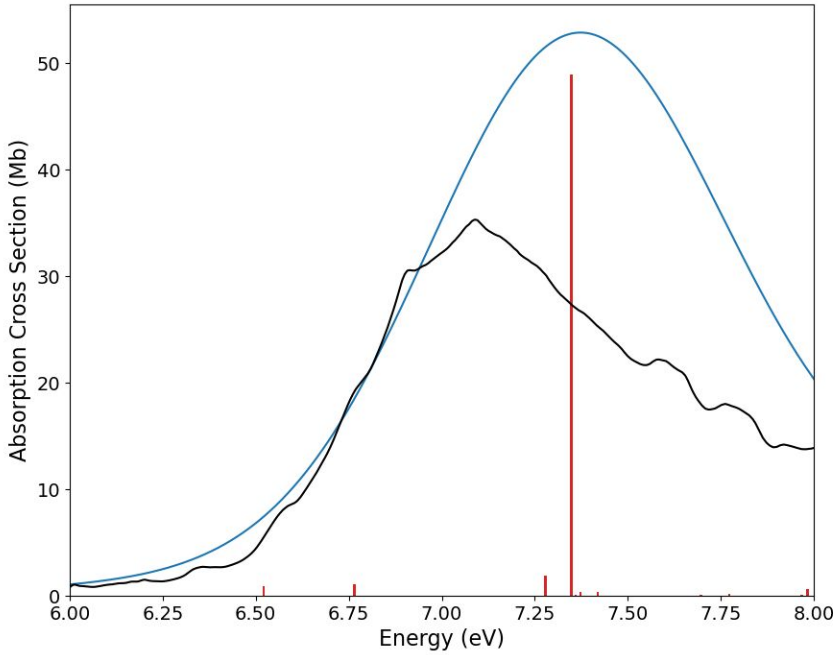

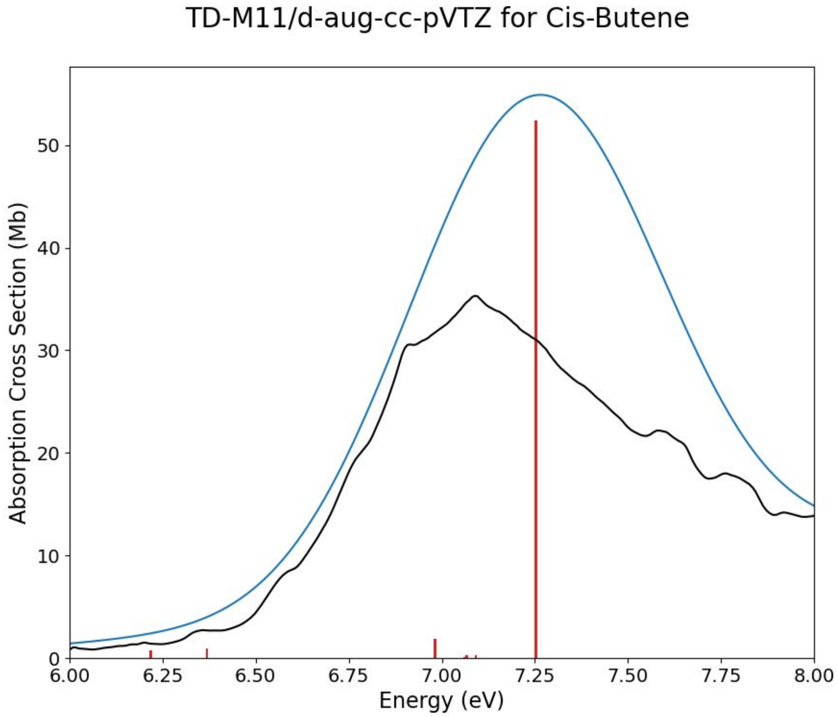
TD-M11/d-aug-cc-pVTZ for Cis-Butene
Absorption Cross Section (Mb)
Energy (eV)
0
10
20
30
40
50
6.00
6.25
6.50
6.75
7.00
7.25
7.50
7.75
8.00

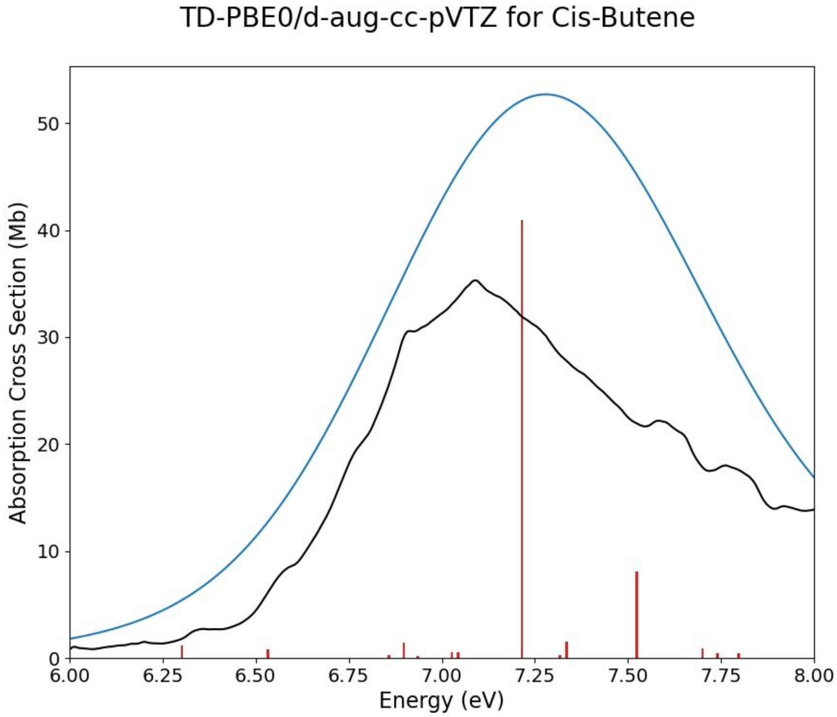
TD-PBE0/d-aug-cc-pVTZ for Cis-Butene
Absorption Cross Section (Mb)
Energy (eV)
0
10
20
30
40
50
6.00
6.25
6.50
6.75
7.00
7.25
7.50
7.75
8.00

TD-xB97x-D/d-aug-cc-pVTZ for Cis-Butene

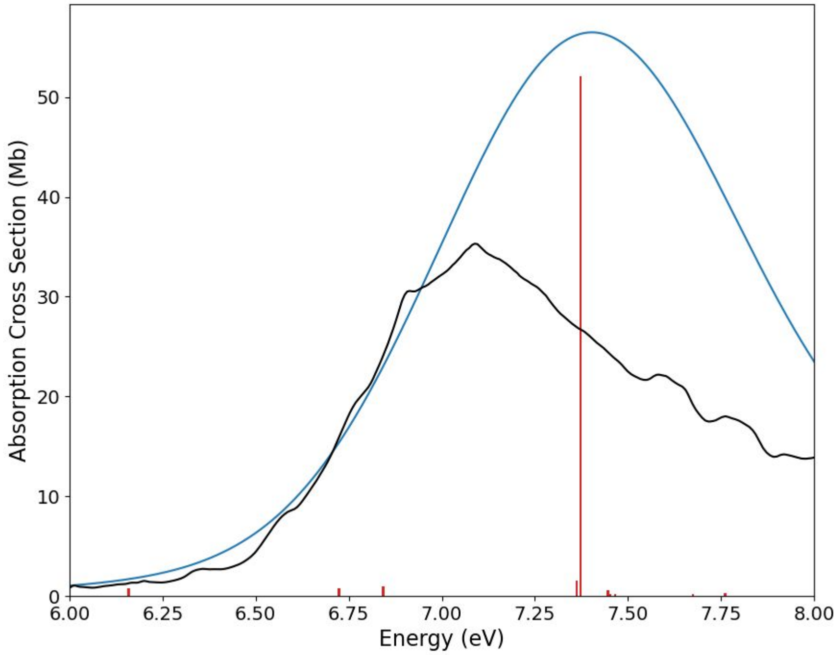

TD-X3LYP/d-aug-cc-pVTZ for Cis-Butene

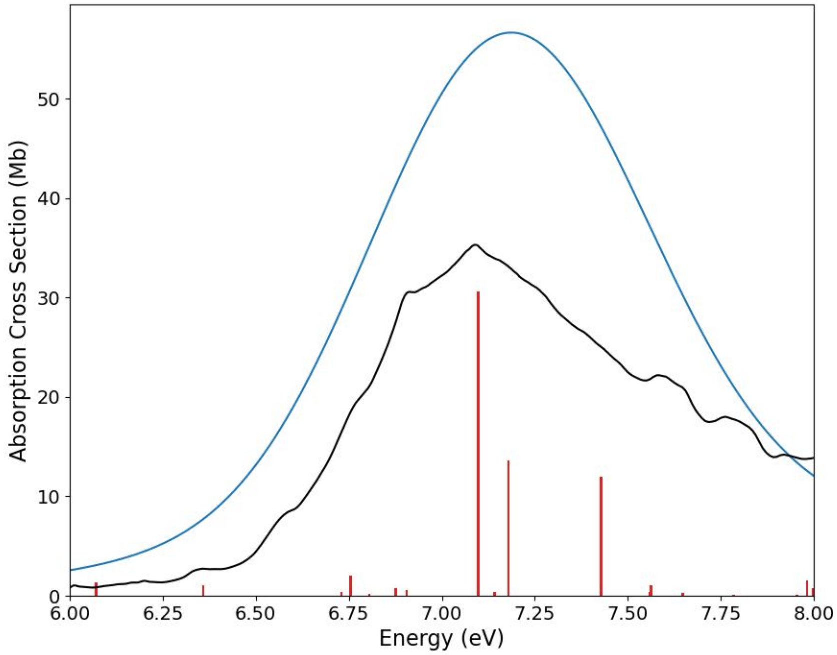

8 trans-Butene

Table S29. Optimized Geometry of trans-Butene in Å

	CCSD(T)/d-aug-cc-pVTZ			M06-2X/d-aug-cc-pVTZ		
C	0.5389039582	-0.3977223066	0.0000000000	0.3230591750	0.5786329175	0.0000000000
C	-0.5389039582	0.3977223066	0.0000000000	-0.3230591750	-0.5786329175	0.0000000000
C	1.9604280910	0.0908878813	0.0000000000	-0.3296392837	1.9250286249	0.0000000000
C	-1.9604280910	-0.0908878813	0.0000000000	0.3296392837	-1.9250286249	0.0000000000
H	0.3899238769	-1.4769155246	0.0000000000	1.4101780250	0.5655185618	0.0000000000
H	-0.3899238769	1.4769155246	0.0000000000	-1.4101780250	-0.5655185618	0.0000000000
H	1.9979725125	1.1827621296	0.0000000000	-1.4149700408	1.8340613373	0.0000000000
H	-1.9979725125	-1.1827621296	0.0000000000	1.4149700408	-1.8340613373	0.0000000000
H	2.5005697295	-0.2712192111	0.8801709350	-0.0346022313	2.5042974252	0.8766583572
H	2.5005697295	-0.2712192111	-0.8801709350	-0.0346022313	2.5042974252	-0.8766583572
H	-2.5005697295	0.2712192111	0.8801709350	0.0346022313	-2.5042974252	0.8766583572
H	-2.5005697295	0.2712192111	-0.8801709350	0.0346022313	-2.5042974252	-0.8766583572

Table S30. Frequencies of trans-Butene in cm^{-1}

CCSD(T)/d-aug-cc-pVTZ	M06-2X/d-aug-cc-pVTZ
174.749	169.585
227.567	225.676
242.087	247.405
275.359	285.806
495.393	507.407
740.161	769.198
874.069	882.830
984.254	987.452
984.946	1010.144
1062.243	1069.292
1070.584	1080.406
1083.553	1094.860
1169.512	1173.065
1322.340	1328.170
1323.518	1336.573
1416.573	1413.529
1417.430	1414.072
1489.582	1483.693
1490.086	1483.816
1499.624	1493.311
1506.882	1501.259
1722.866	1773.247
3019.952	3056.026
3020.573	3056.197
3079.530	3108.532

3079.932	3109.009
3101.136	3133.929
3102.405	3134.573
3120.944	3149.273
3126.555	3155.843

Table S31. Transition Energies of trans-Butene in eV

CCSD(T)/d-aug-cc-pVTZ	M06-2X/d-aug-cc-pVTZ
6.513	6.111
6.935	6.479
7.105	6.604
7.217	6.776
7.409	6.979
7.769	7.246
7.807	7.344
7.893	7.363
7.911	7.379
8.133	7.660
8.199	7.687
8.225	7.710
8.249	7.766
8.319	7.774
8.492	7.935
8.523	7.941
8.562	7.967
8.586	7.990
8.628	7.999
8.632	8.081
8.653	8.124
8.770	8.242
8.773	8.275
8.786	8.285
8.916	8.340
8.944	8.426
8.973	8.461
9.097	8.521
9.099	8.592
	8.625
	8.644
	8.665
	8.704
	8.771
	8.805
	8.813
	8.863
	8.889

	8.957<br>9.020<br>9.088

Table S32. Quantitative Metrics for the Bandwidth (γ), cosine similarity (S), relative integral change (RIC), mean signed error (MSE), and mean average error (MAE) for the band shape of trans-butene compared to experiment.

Method	γ	S	RIC	MSE	MAE
B3LYP	0.35	0.976	0.734	8.659	8.659
BH&HLYP	0.38	0.999	0.198	-2.258	2.258
BMK	0.43	0.997	0.207	-2.335	2.374
CAM-B3LYP	0.35	0.999	0.035	-0.036	0.420
CC	0.33	0.997	0.853	-9.844	9.844
HSE	0.3	0.999	0.311	3.573	3.573
M06-2X	0.35	0.999	0.070	-0.624	0.801
M11	0.2	0.995	0.311	3.019	3.539
PBE0	0.37	0.990	0.380	4.450	4.450
wB97x-D	0.38	0.998	0.100	-0.846	1.116
X3LYP	0.35	0.975	0.735	8.636	8.636

Figure S8. Spectra of trans-Butene

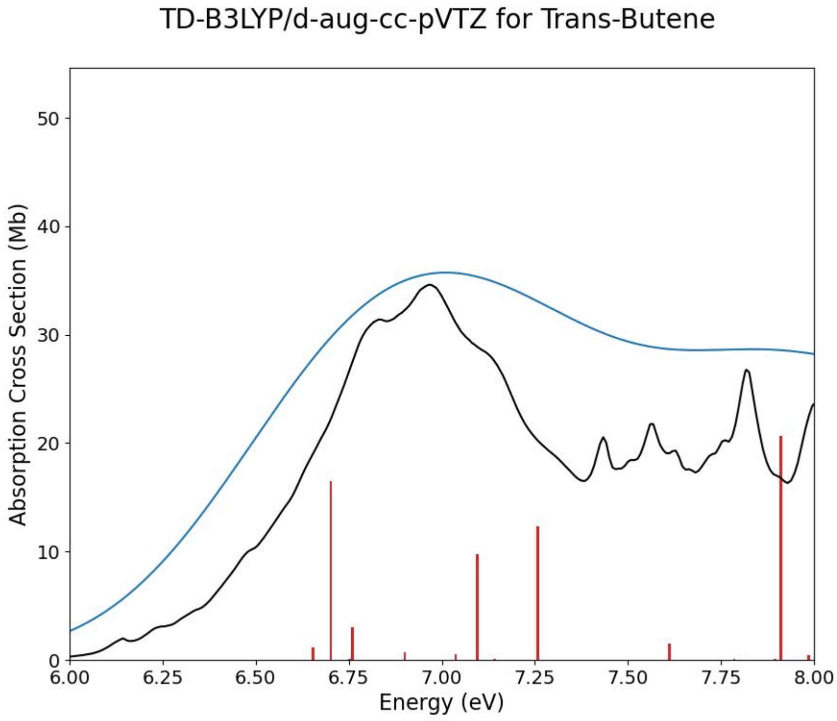

TD-BH&HLYP/d-aug-cc-pVTZ for Trans-Butene

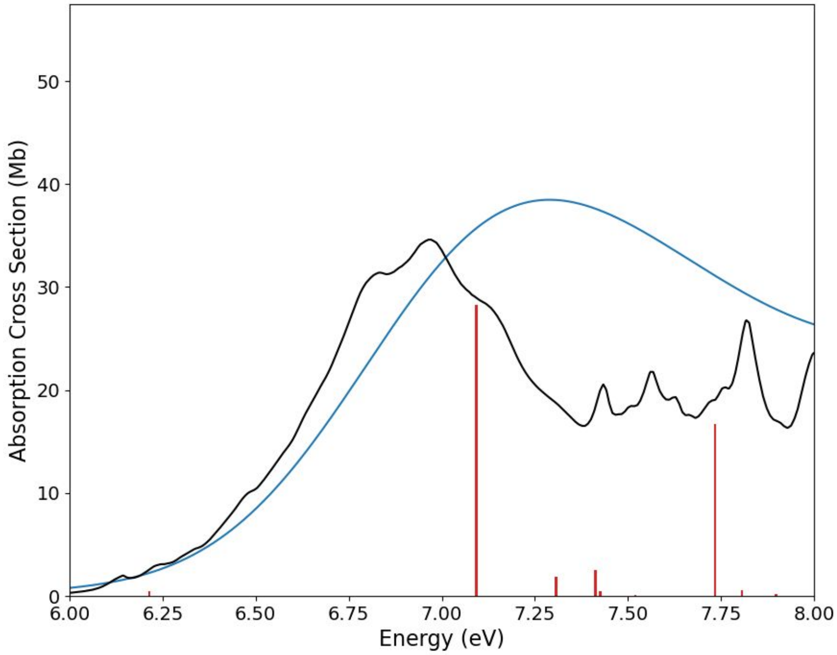

TD-BMK/d-aug-cc-pVTZ for Trans-Butene

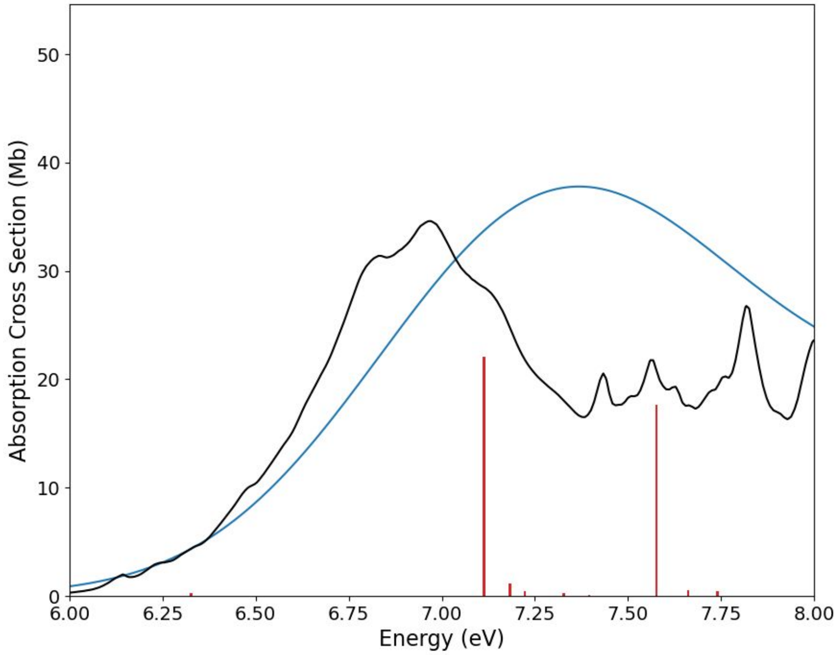

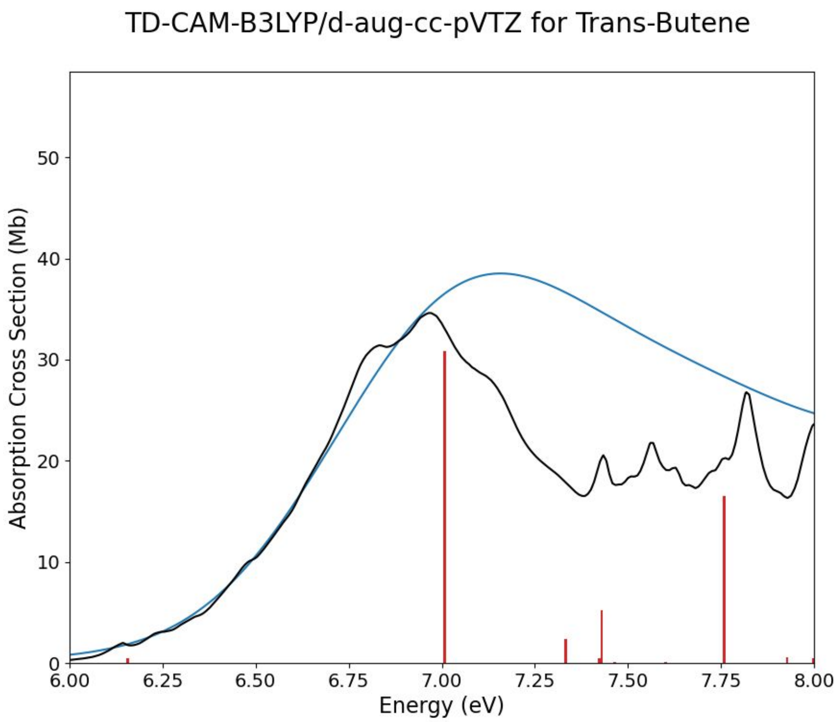
TD-CAM-B3LYP/d-aug-cc-pVTZ for Trans-Butene
Absorption Cross Section (Mb)
0
10
20
30
40
50
6.00
6.25
6.50
6.75
7.00
7.25
7.50
7.75
8.00
Energy (eV)

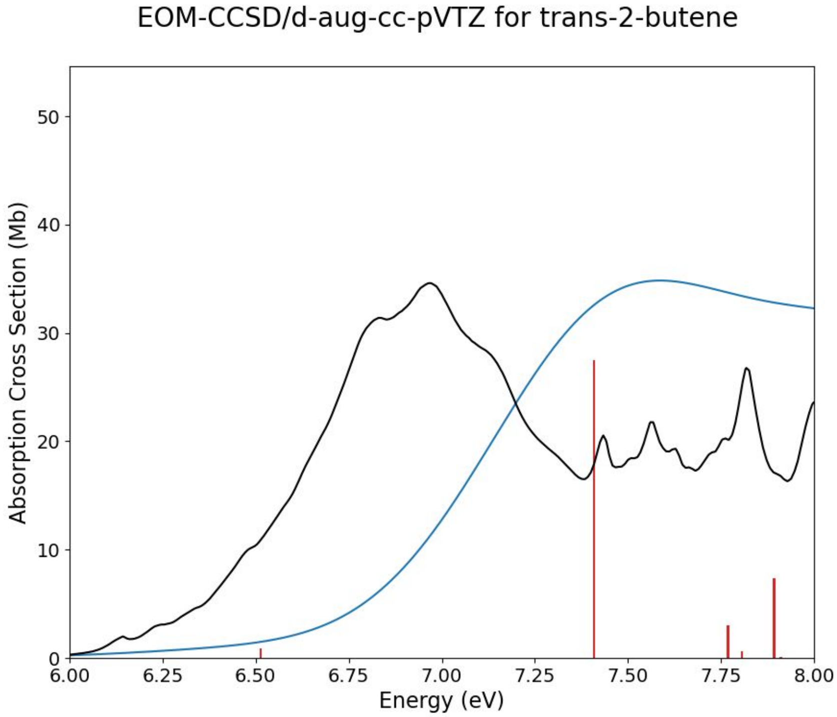
EOM-CCSD/d-aug-cc-pVTZ for trans-2-butene
Absorption Cross Section (Mb)
0
10
20
30
40
50
6.00
6.25
6.50
6.75
7.00
7.25
7.50
7.75
8.00
Energy (eV)

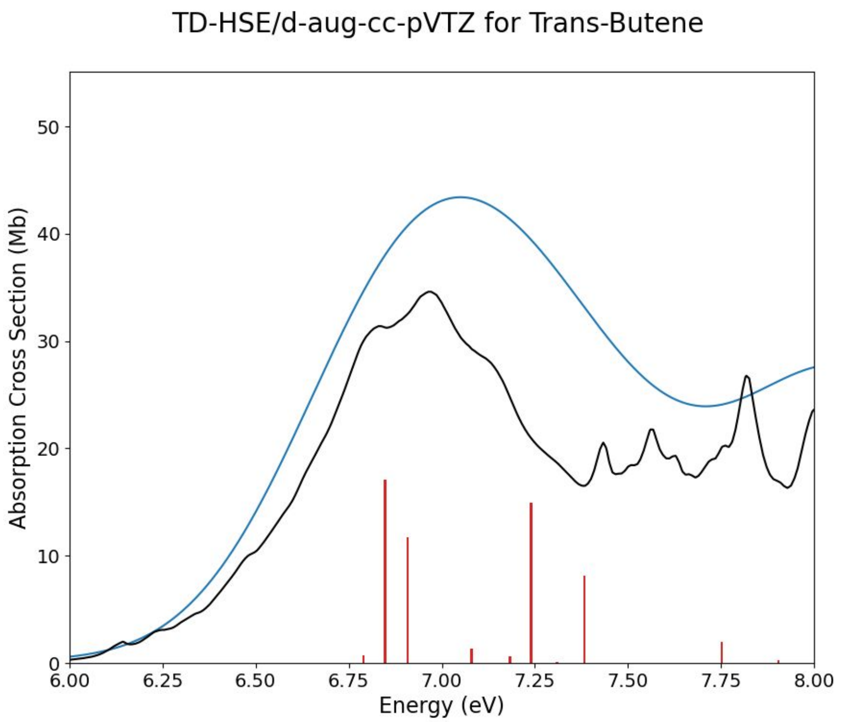
TD-HSE/d-aug-cc-pVTZ for Trans-Butene
Absorption Cross Section (Mb)
Energy (eV)
0
10
20
30
40
50
6.00
6.25
6.50
6.75
7.00
7.25
7.50
7.75
8.00

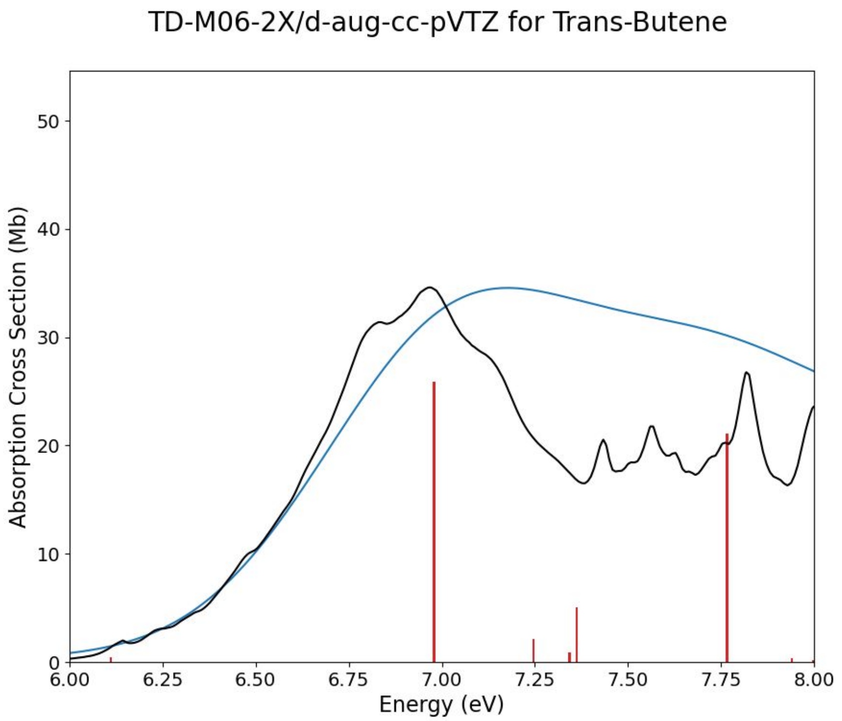
TD-M06-2X/d-aug-cc-pVTZ for Trans-Butene
Absorption Cross Section (Mb)
Energy (eV)
0
10
20
30
40
50
6.00
6.25
6.50
6.75
7.00
7.25
7.50
7.75
8.00

TD-M11/d-aug-cc-pVTZ for Trans-Butene

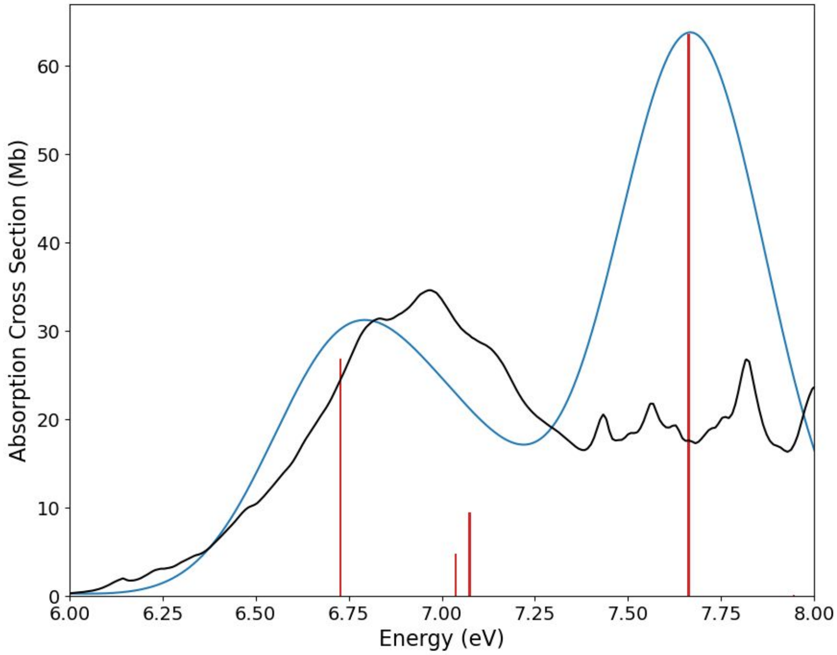

TD-PBE0/d-aug-cc-pVTZ for Trans-Butene

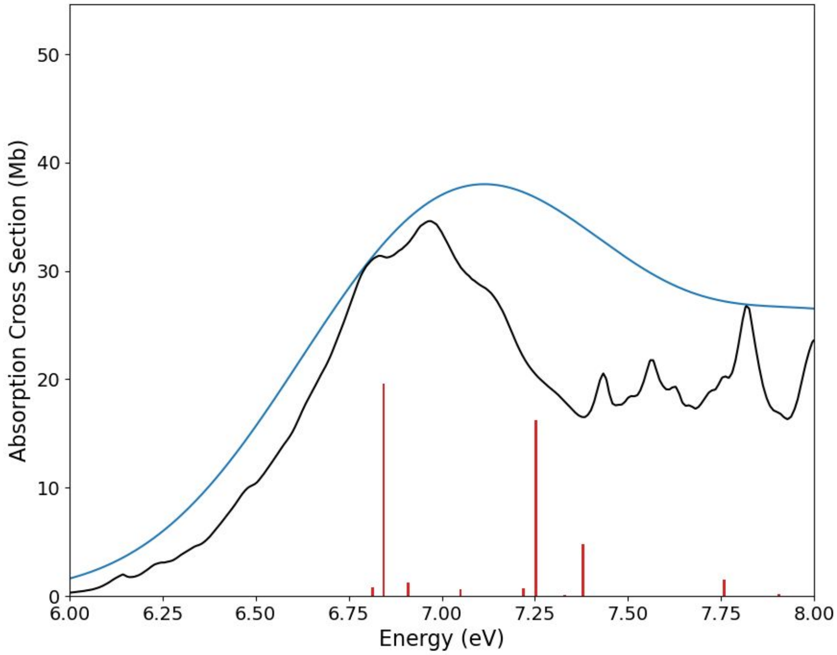

TD-wB97x-D/d-aug-cc-pVTZ for Trans-Butene

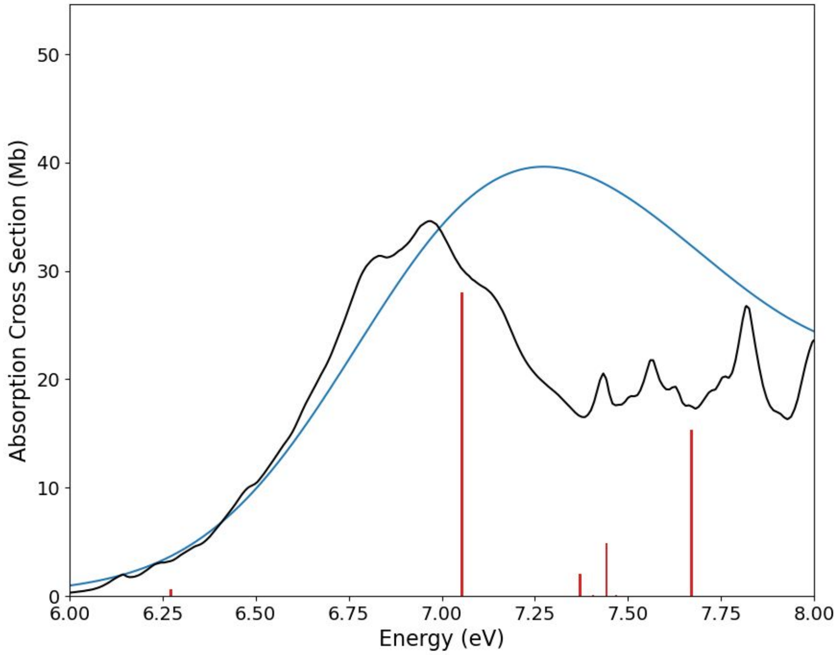

TD-X3LYP/d-aug-cc-pVTZ for Trans-Butene

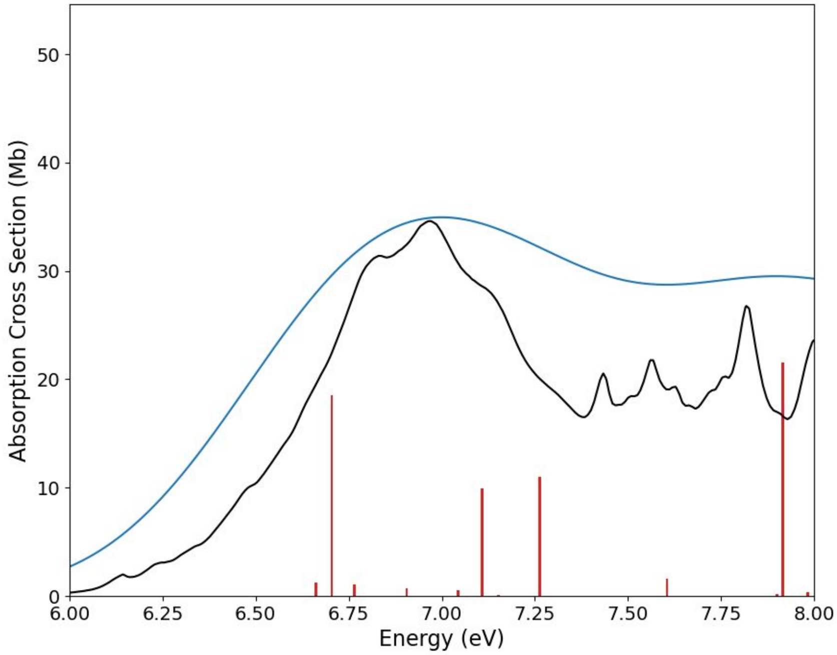

9 Acetaldehyde

Table S33. Optimized Geometry of Acetaldehyde in Å

	CCSD(T)/d-aug-cc-pVTZ			M06-2X/d-aug-cc-pVTZ		
C	0.0000000000	-0.4218188588	-0.1242540186	-0.0894927268	0.4271615132	0.0000000000
C	0.0000000000	0.1665290535	1.2609861412	-1.0196437644	-0.7480717889	0.0000000000
O	0.0000000000	0.2326917624	-1.1463203681	1.1074997035	0.3446556952	0.0000000000
H	0.0000000000	-1.5286110106	-0.1739294560	-0.5764308131	1.4213986338	0.0000000000
H	0.0000000000	1.2546549628	1.2137020571	-0.4594030628	-1.6786605812	0.0000000000
H	-0.8802475754	-0.1887570065	1.8052265897	-1.6673459616	-0.6958251854	0.8768364711
H	0.8802475754	-0.1887570065	1.8052265897	-1.6673459616	-0.6958251854	-0.8768364711

Table S34. Frequencies of Acetaldehyde in cm^{-1}

CCSD(T)/d-aug-cc-pVTZ	M06-2X/d-aug-cc-pVTZ
160.335	148.374
503.523	512.711
775.800	774.872
896.007	900.478
1130.603	1137.967
1136.253	1145.107
1388.366	1380.798
1420.453	1430.910
1473.868	1465.329
1484.648	1474.567
1777.439	1864.777
2918.344	2939.924
3031.241	3065.001
3099.422	3127.600
3151.144	3181.345

Table S35. Transition Energies of Acetaldehyde in eV

CCSD(T)/d-aug-cc-pVTZ	M06-2X/d-aug-cc-pVTZ
4.326	4.112
6.986	6.915
7.673	7.515
7.843	7.632
7.884	7.686
8.575	8.341
8.595	8.375
8.699	8.469
8.766	8.499
8.780	8.510
8.867	8.601

9.058	8.714<br>8.750<br>8.791<br>8.808

Table S36. Quantitative Metrics for the Bandwidth (γ), cosine similarity (S), relative integral change (RIC), mean signed error (MSE), and mean average error (MAE) for the band shape of acetaldehyde compared to experiment.

Method	γ	S	RIC	MSE	MAE
B3LYP	0.06	0.548	1.233	1.826	6.353
BH&HLYP	0.06	0.527	0.858	-2.429	4.468
BMK	0.05	0.390	1.005	-2.402	5.339
CAM-B3LYP	0.07	0.464	1.188	-0.856	5.812
CC	0.07	0.527	0.919	-1.194	4.597
HSE	0.06	0.664	2.088	9.915	10.974
M06-2X	0.07	0.663	0.708	-2.039	3.523
M11	0.05	0.542	2.333	8.801	12.187
PBE0	0.05	0.657	2.176	10.557	11.697
wB97x-D	0.07	0.527	1.072	-0.695	5.422
X3LYP	0.06	0.507	1.533	4.075	8.305

Figure S9. Spectra of Acetaldehyde

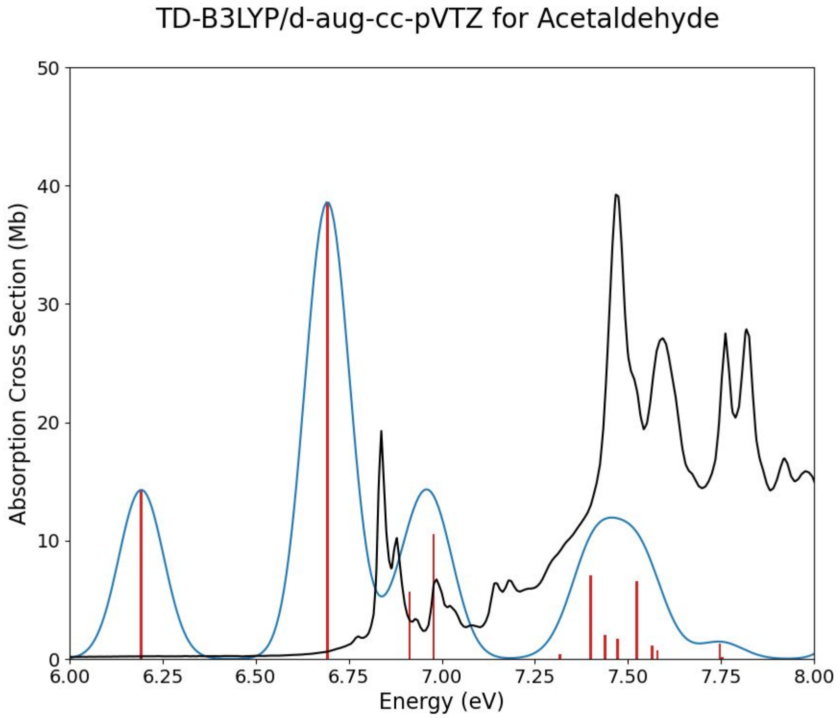

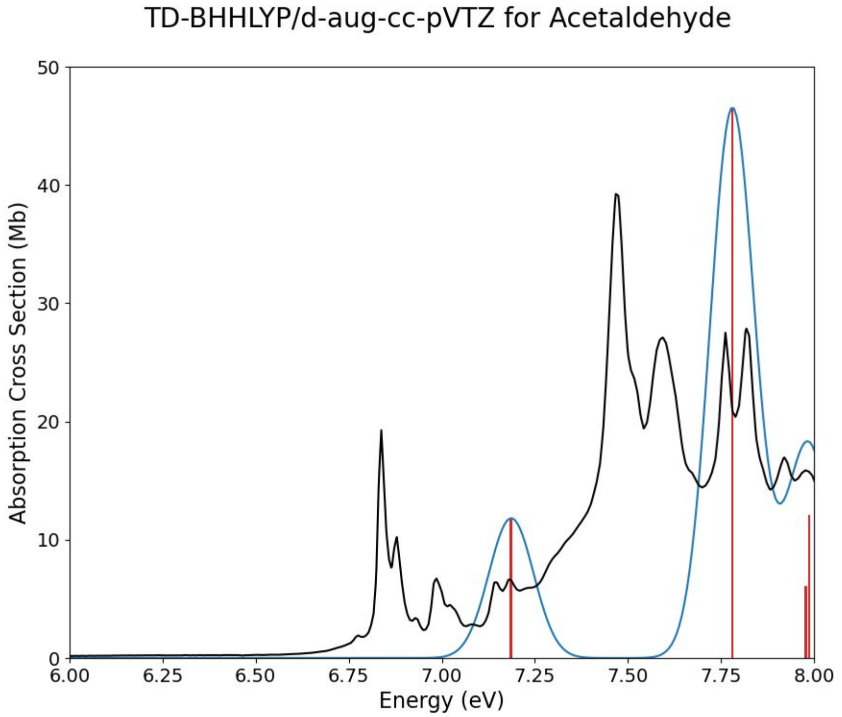
TD-BHHLYP/d-aug-cc-pVTZ for Acetaldehyde
Absorption Cross Section (Mb)
Energy (eV)
0
10
20
30
40
50
6.00
6.25
6.50
6.75
7.00
7.25
7.50
7.75
8.00

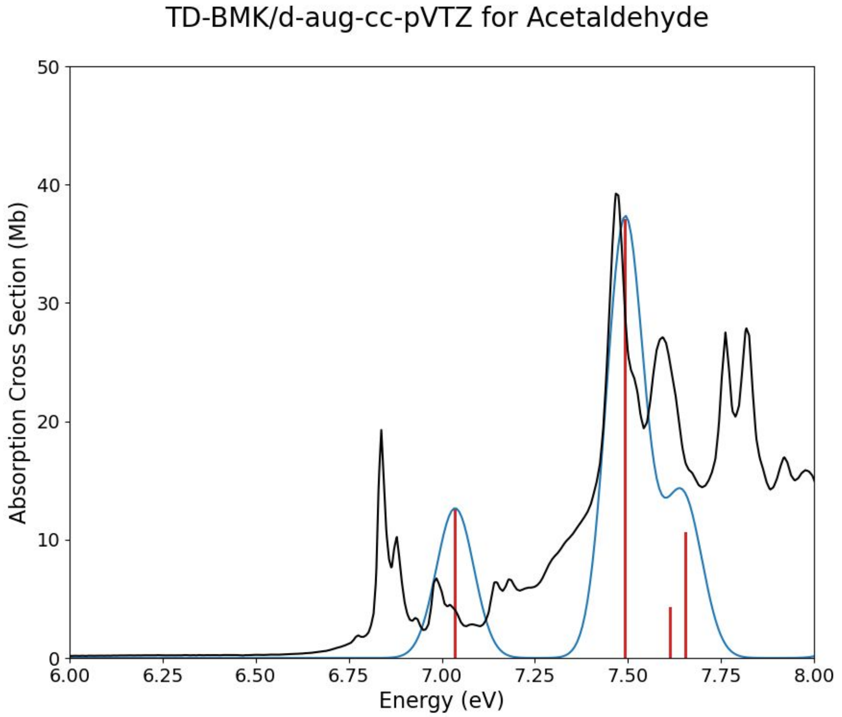
TD-BMK/d-aug-cc-pVTZ for Acetaldehyde
Absorption Cross Section (Mb)
Energy (eV)
0
10
20
30
40
50
6.00
6.25
6.50
6.75
7.00
7.25
7.50
7.75
8.00

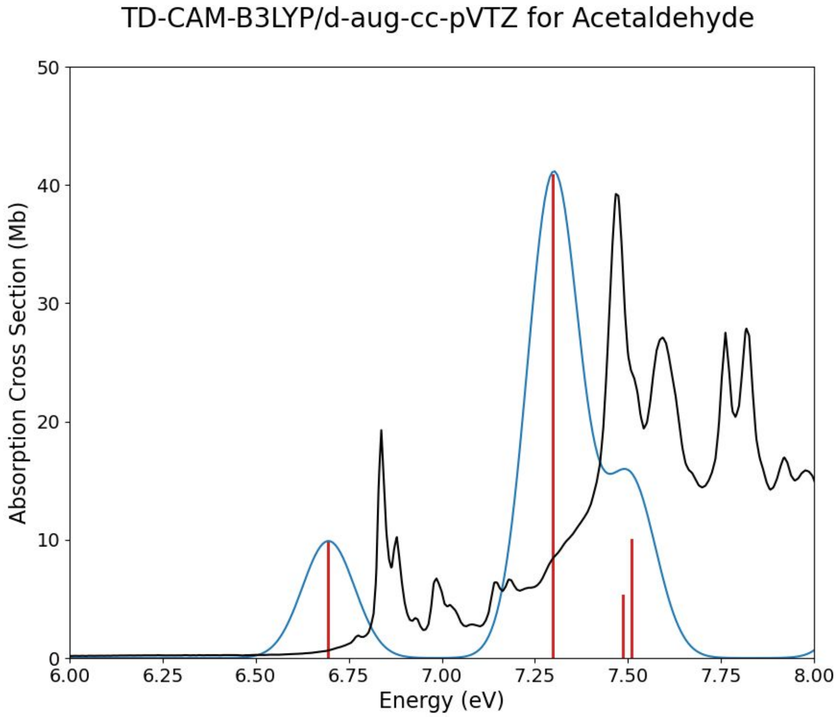
TD-CAM-B3LYP/d-aug-cc-pVTZ for Acetaldehyde
Absorption Cross Section (Mb)
Energy (eV)
0
10
20
30
40
50
6.00
6.25
6.50
6.75
7.00
7.25
7.50
7.75
8.00

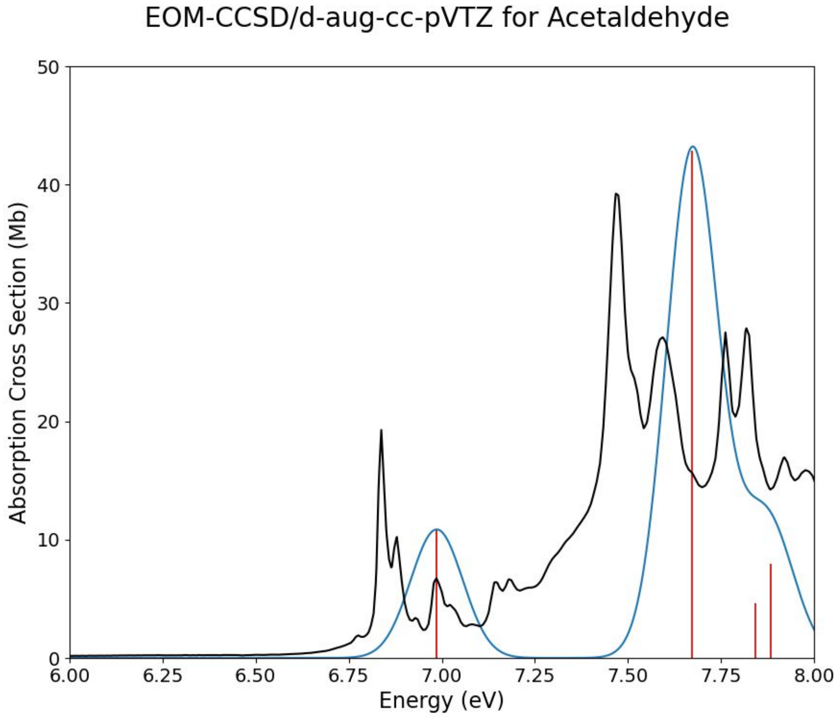
EOM-CCSD/d-aug-cc-pVTZ for Acetaldehyde
Absorption Cross Section (Mb)
Energy (eV)
0
10
20
30
40
50
6.00
6.25
6.50
6.75
7.00
7.25
7.50
7.75
8.00

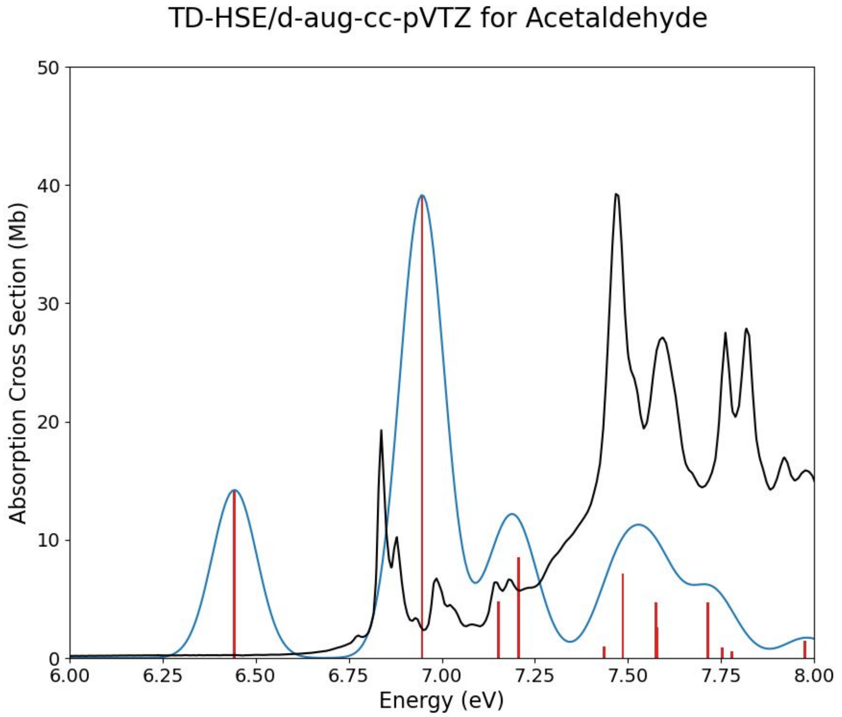
TD-HSE/d-aug-cc-pVTZ for Acetaldehyde
Absorption Cross Section (Mb)
Energy (eV)
0
10
20
30
40
50
6.00
6.25
6.50
6.75
7.00
7.25
7.50
7.75
8.00

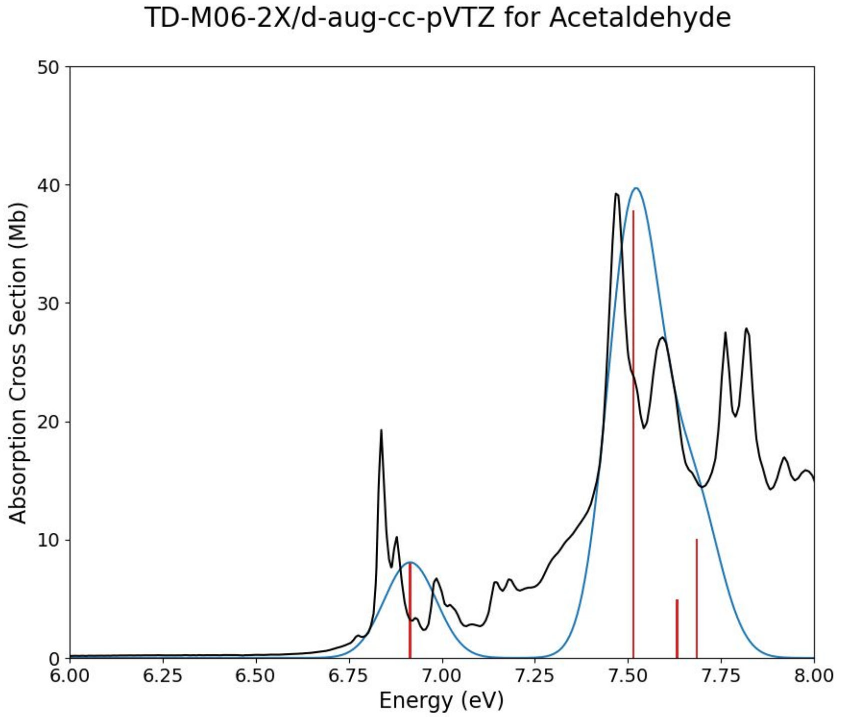
TD-M06-2X/d-aug-cc-pVTZ for Acetaldehyde
Absorption Cross Section (Mb)
Energy (eV)
0
10
20
30
40
50
6.00
6.25
6.50
6.75
7.00
7.25
7.50
7.75
8.00

TD-M11/d-aug-cc-pVTZ for Acetaldehyde

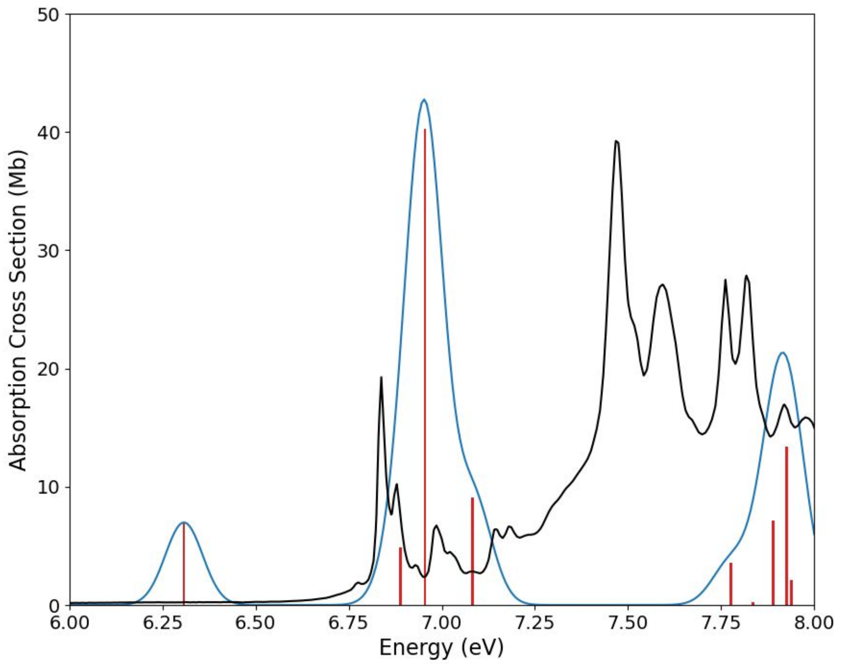

TD-PBE0/d-aug-cc-pVTZ for Acetaldehyde

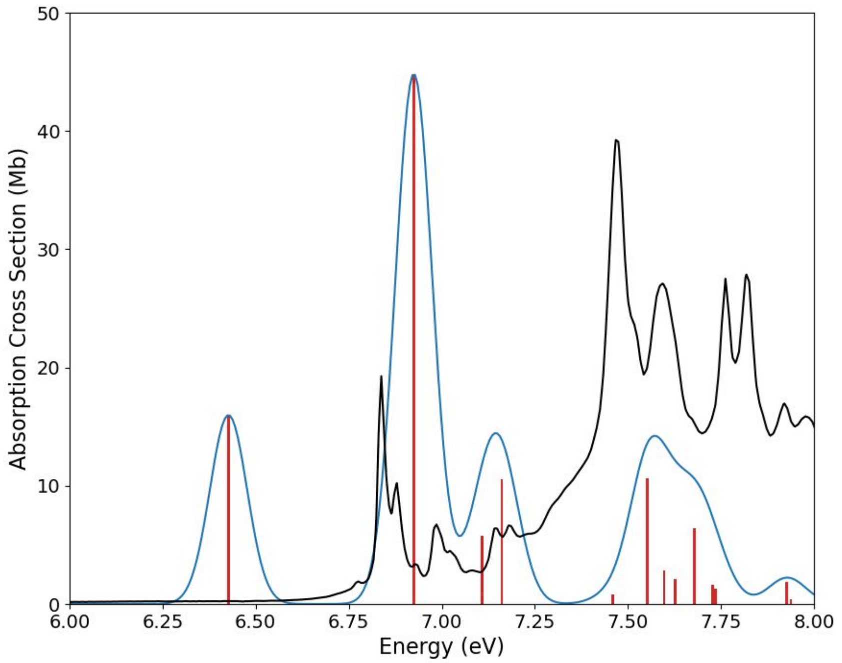

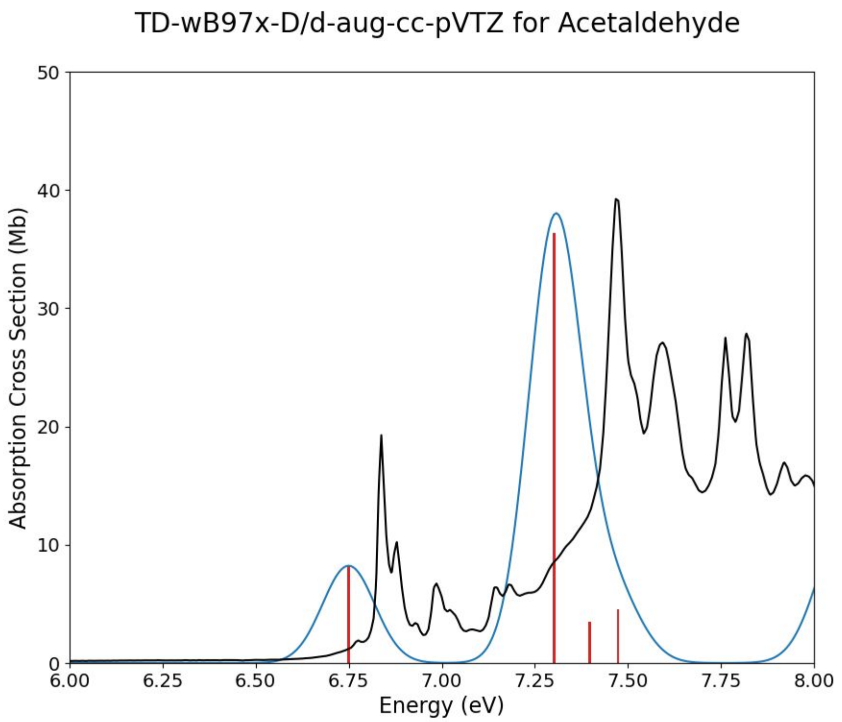
TD-wB97x-D/d-aug-cc-pVTZ for Acetaldehyde
Absorption Cross Section (Mb)
Energy (eV)
0
10
20
30
40
50
6.00
6.25
6.50
6.75
7.00
7.25
7.50
7.75
8.00

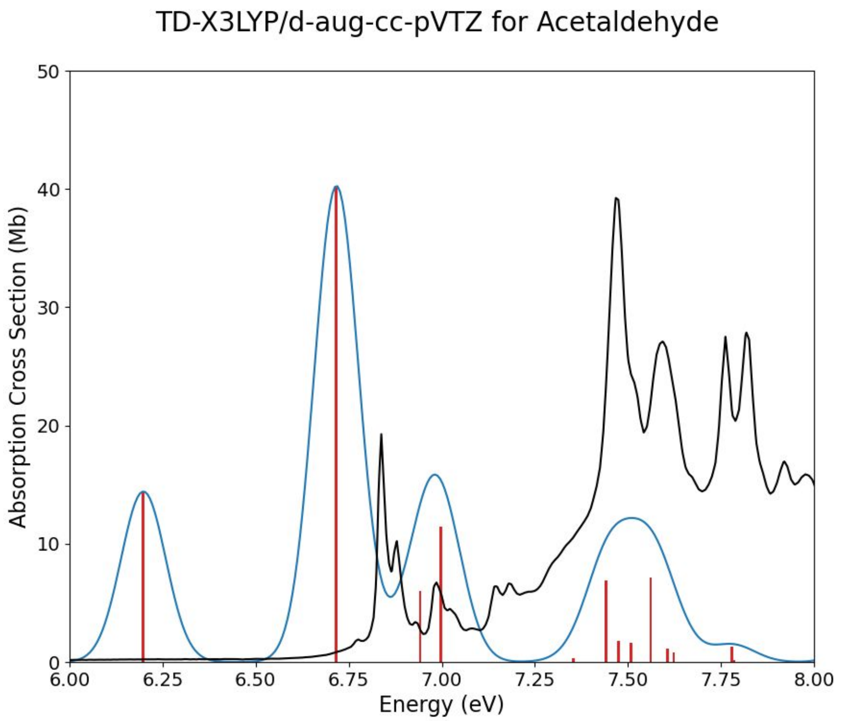
TD-X3LYP/d-aug-cc-pVTZ for Acetaldehyde
Absorption Cross Section (Mb)
Energy (eV)
0
10
20
30
40
50
6.00
6.25
6.50
6.75
7.00
7.25
7.50
7.75
8.00

10 Acetone

Table S37. Optimized Geometry of Acetone in Å

	CCSD(T)/d-aug-cc-pVTZ			M06-2X/d-aug-cc-pVTZ		
C	0.0000000000	0.0000000000	0.0998864490	0.0000000000	0.0000000000	0.1022786947
O	0.0000000000	0.0000000000	1.3176672548	0.0000000000	0.0000000000	1.3070938989
C	-1.2875636905	0.0000000000	-0.6991981050	0.0000000000	1.2821613089	-0.6946179664
C	1.2875636905	0.0000000000	-0.6991981050	0.0000000000	-1.2821613089	-0.6946179664
H	-2.1433189464	0.0000000000	-0.0262464164	0.0000000000	2.1370193906	-0.0255397932
H	2.1433189464	0.0000000000	-0.0262464164	0.0000000000	-2.1370193906	-0.0255397932
H	-1.3221777683	0.8804882625	-1.3474450636	0.8770926206	1.3151259225	-1.3424674410
H	-1.3221777683	-0.8804882625	-1.3474450636	-0.8770926206	1.3151259225	-1.3424674410
H	1.3221777683	-0.8804882625	-1.3474450636	-0.8770926206	-1.3151259225	-1.3424674410
H	1.3221777683	0.8804882625	-1.3474450636	0.8770926206	-1.3151259225	-1.3424674410

Table S38. Frequencies of Acetone in cm^{-1}

CCSD(T)/d-aug-cc-pVTZ	M06-2X/d-aug-cc-pVTZ
47.816	69.518
142.843	160.008
372.392	385.563
480.183	494.982
528.422	536.302
794.770	807.790
891.153	886.997
896.890	900.737
1083.444	1087.880
1118.336	1126.000
1246.630	1251.808
1391.767	1389.308
1398.366	1398.713
1470.756	1464.683
1476.256	1470.390
1478.693	1475.508
1499.377	1494.305
1769.789	1847.655
3030.573	3066.455
3035.665	3072.009
3097.753	3127.848
3104.001	3134.837
3151.964	3184.028
3153.356	3184.905

Table S39. Transition Energies of Acetone in eV

CCSD(T)/d-aug-cc-pVTZ	M06-2X/d-aug-cc-pVTZ
4.504	4.279
6.590	6.553
7.498	7.355
7.577	7.439
7.597	7.449
7.982	7.845
8.213	8.078
8.230	8.101
8.271	8.129
8.291	8.131
8.417	8.303
8.659	8.454
8.692	8.467
8.714	8.506
8.884	8.691
8.930	8.705
8.979	8.738
8.980	8.792
9.005	8.838
9.023	8.840
	8.863
	8.900
	9.032
	9.053
	9.055
	9.067

Table S40. Quantitative Metrics for the Bandwidth (γ), cosine similarity (S), relative integral change (RIC), mean signed error (MSE), and mean average error (MAE) for the band shape of acetone compared to experiment.

Method	γ	S	RIC	MSE	MAE
B3LYP	0.05	0.454	0.826	-4.559	4.817
BH&HLYP	0.06	0.089	0.990	-5.234	5.578
BMK	0.05	0.209	1.499	-0.770	8.290
CAM-B3LYP	0.06	0.695	1.056	0.725	5.971
CC	0.05	0.387	1.347	0.201	6.692
HSE	0.06	0.121	0.981	-5.230	5.230
M06-2X	0.05	0.493	1.076	0.030	5.982
M11	0.04	0.049	1.000	-5.482	5.482
PBE0	0.05	0.102	0.986	-5.170	5.170
wB97x-D	0.05	0.619	1.063	0.448	5.655
X3LYP	0.05	0.367	0.859	-4.603	4.902

Figure S10. Spectra of Acetone

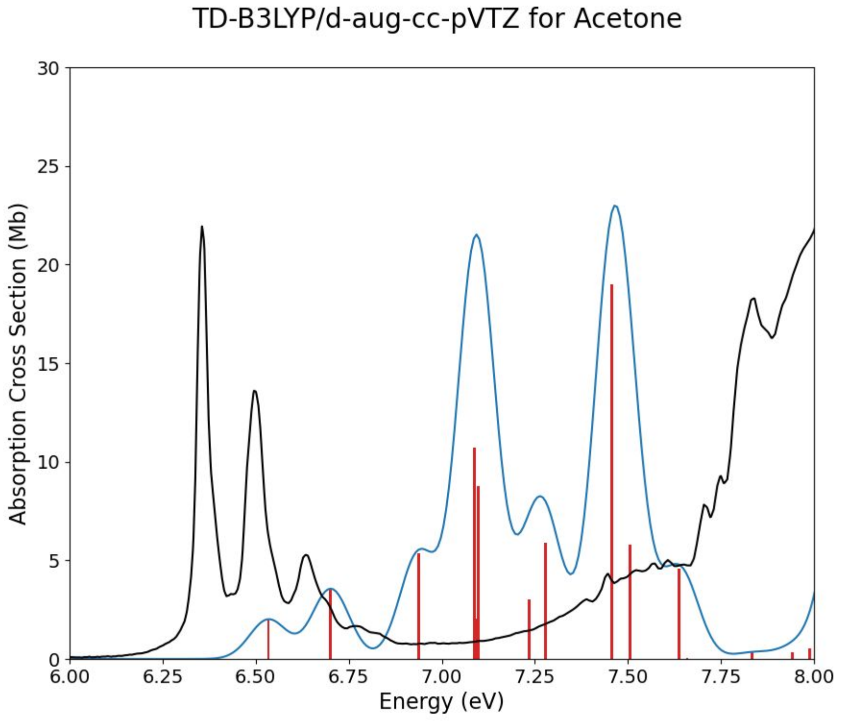

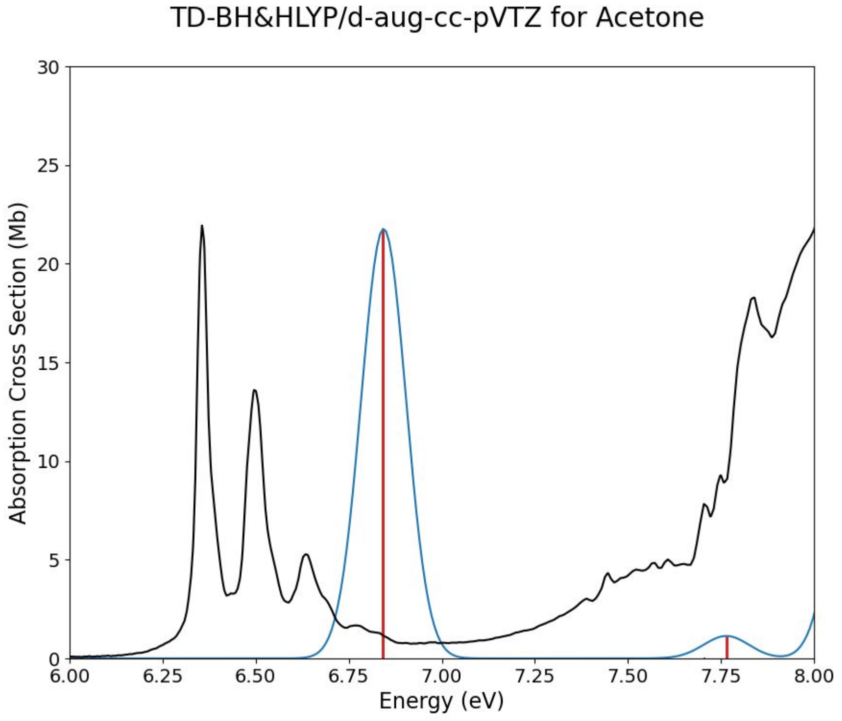

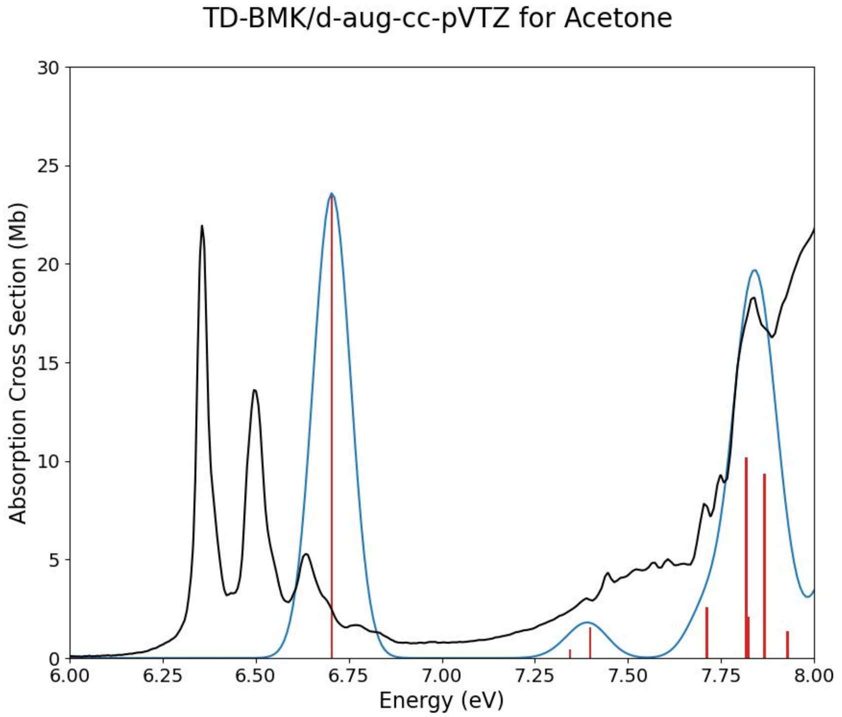
TD-BMK/d-aug-cc-pVTZ for Acetone
Absorption Cross Section (Mb)
Energy (eV)
0
5
10
15
20
25
30
6.00
6.25
6.50
6.75
7.00
7.25
7.50
7.75
8.00

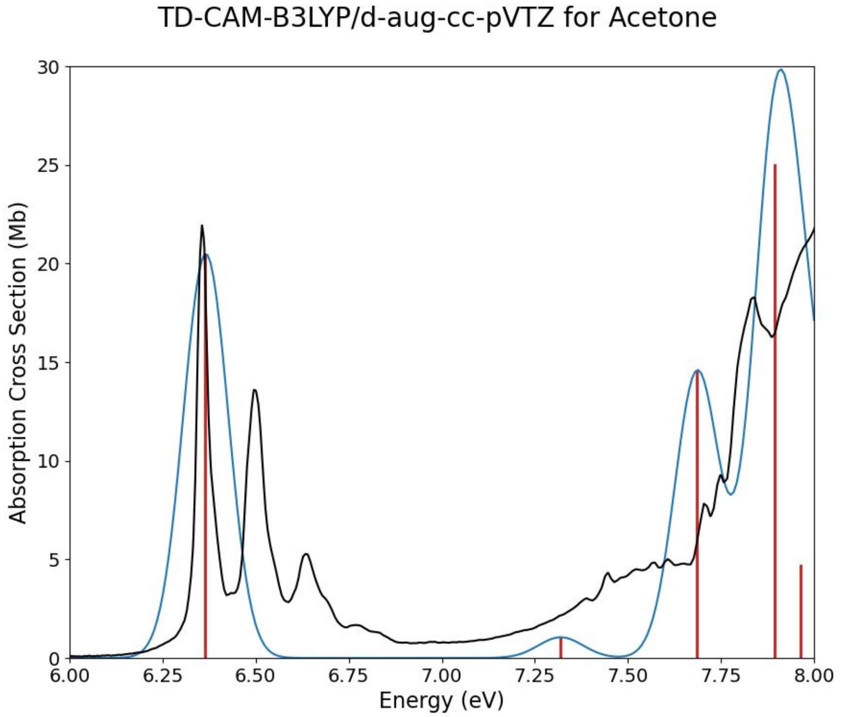
TD-CAM-B3LYP/d-aug-cc-pVTZ for Acetone
Absorption Cross Section (Mb)
Energy (eV)
0
5
10
15
20
25
30
6.00
6.25
6.50
6.75
7.00
7.25
7.50
7.75
8.00

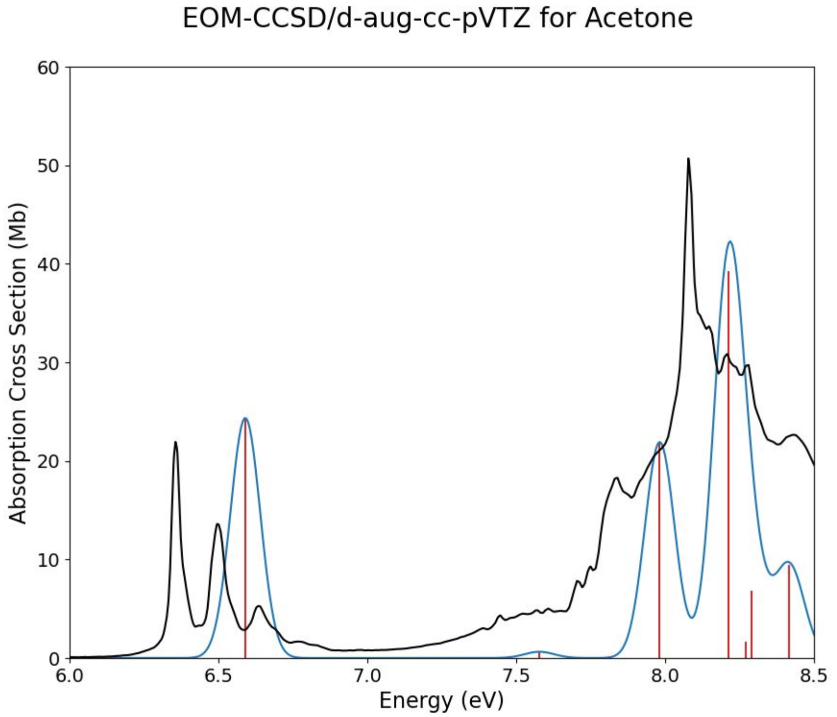
EOM-CCSD/d-aug-cc-pVTZ for Acetone
Absorption Cross Section (Mb)
Energy (eV)
0
10
20
30
40
50
60
6.0
6.5
7.0
7.5
8.0
8.5

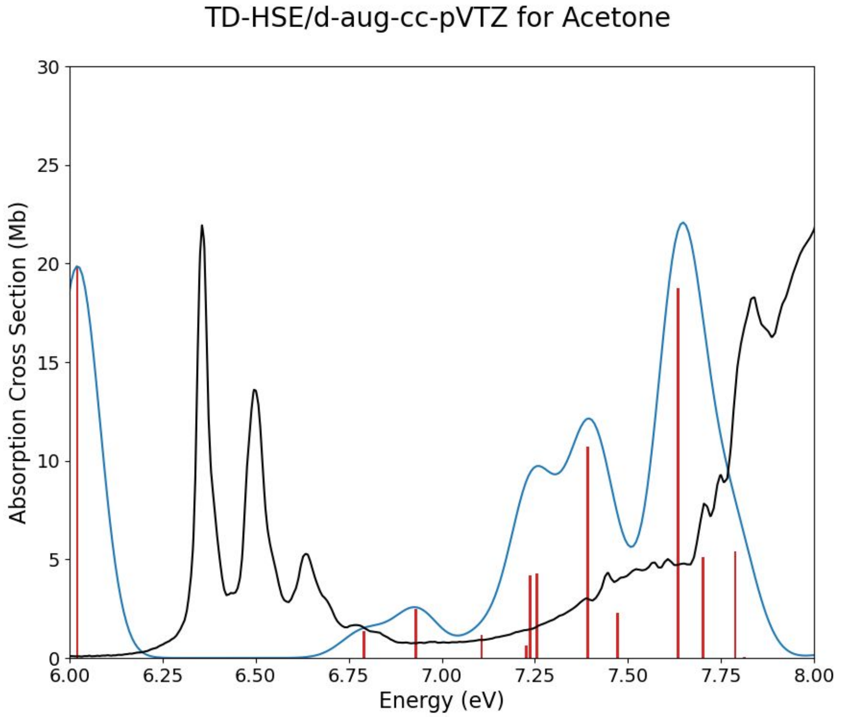
TD-HSE/d-aug-cc-pVTZ for Acetone
Absorption Cross Section (Mb)
Energy (eV)
0
5
10
15
20
25
30
6.00
6.25
6.50
6.75
7.00
7.25
7.50
7.75
8.00

TD-M06-2X/d-aug-cc-pVTZ for Acetone

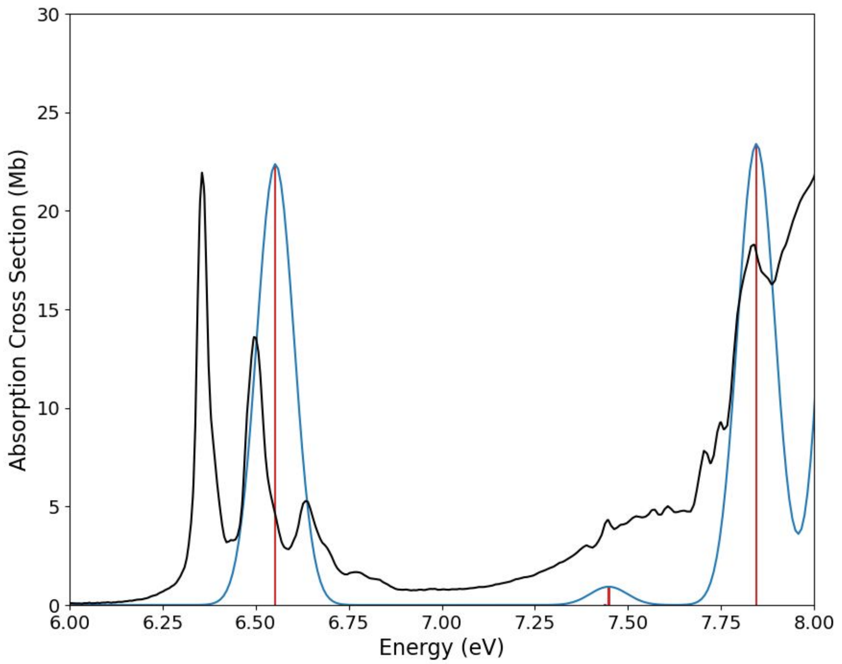

TD-M11/d-aug-cc-pVTZ for Acetone

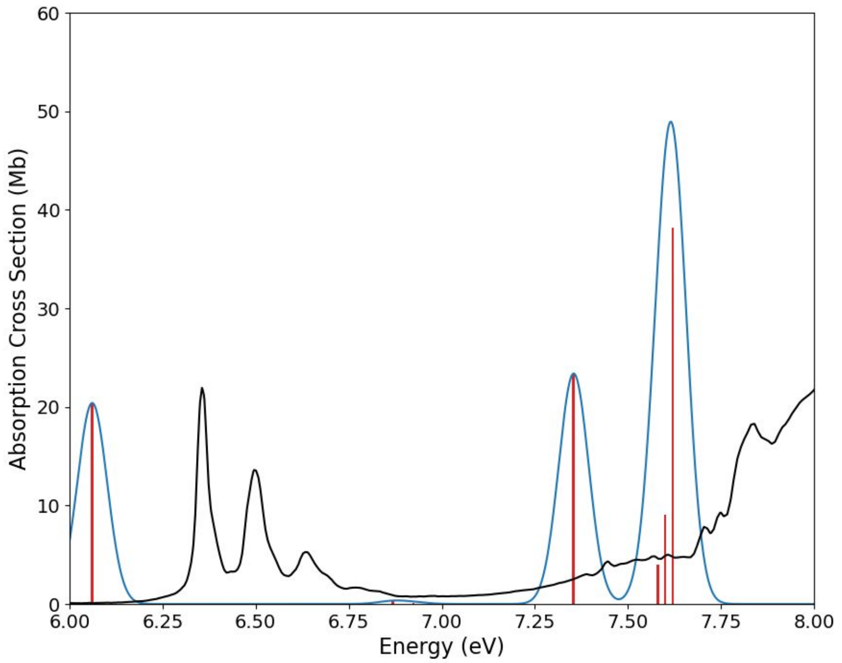

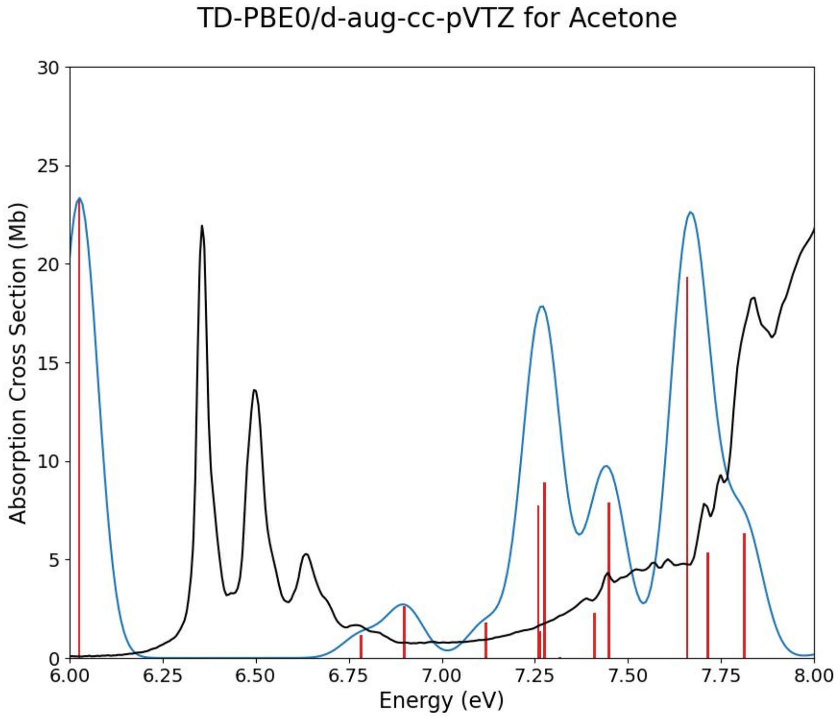
TD-PBE0/d-aug-cc-pVTZ for Acetone
Absorption Cross Section (Mb)
Energy (eV)
0
5
10
15
20
25
30
6.00
6.25
6.50
6.75
7.00
7.25
7.50
7.75
8.00

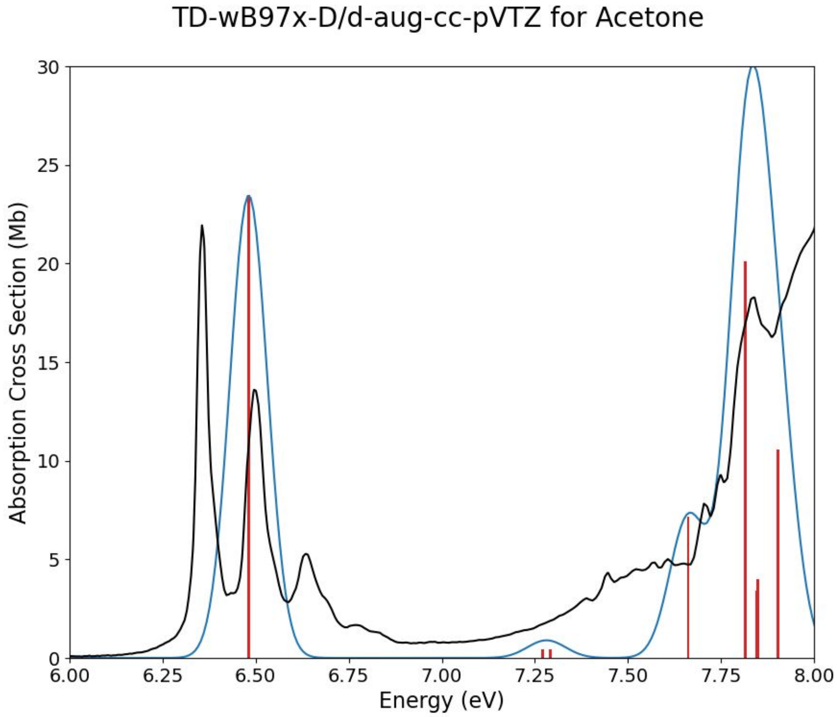
TD-wB97x-D/d-aug-cc-pVTZ for Acetone
Absorption Cross Section (Mb)
Energy (eV)
0
5
10
15
20
25
30
6.00
6.25
6.50
6.75
7.00
7.25
7.50
7.75
8.00

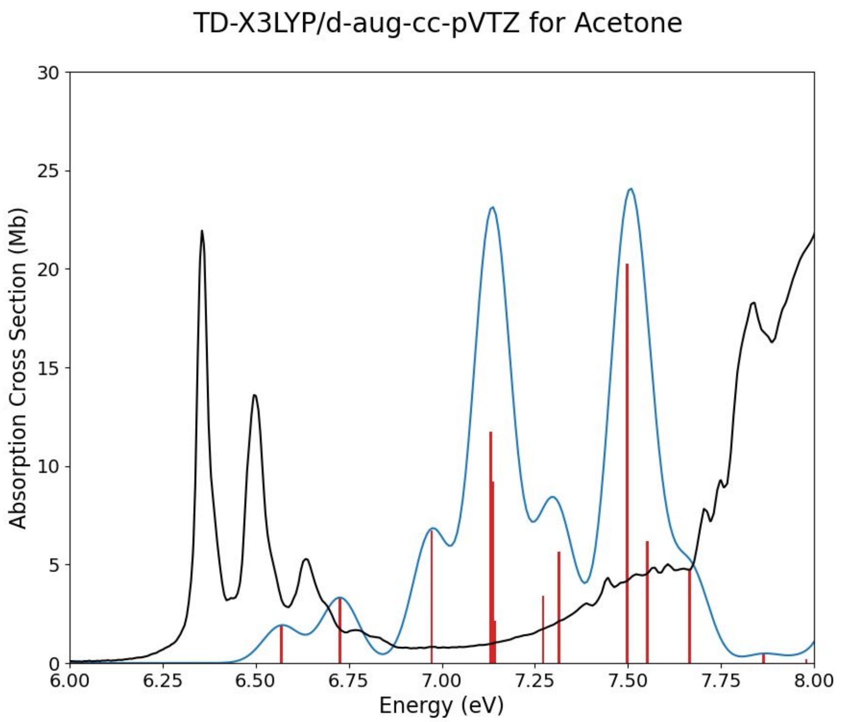
TD-X3LYP/d-aug-cc-pVTZ for Acetone
Absorption Cross Section (Mb)
Energy (eV)
0
5
10
15
20
25
30
6.00
6.25
6.50
6.75
7.00
7.25
7.50
7.75
8.00

11 Methyl Vinyl Ketone

Table S41. Optimized Geometry of Methyl Vinyl Ketone in Å

	CCSD(T)/d-aug-cc-pVTZ			M06-2X/d-aug-cc-pVTZ		
C	0.0000000000	-0.7207608152	-1.7320722441	0.3236807398	-1.8373654411	0.0000000000
C	0.0000000000	0.0912937871	-0.4563501655	0.3236807398	-0.3312147815	0.0000000000
O	0.0000000000	1.3116880774	-0.4637126062	-0.2123432972	-2.2036993111	0.8772951230
C	0.0000000000	-0.6894542048	0.8219663077	1.3418269542	-2.2131481748	0.0000000000
C	0.0000000000	-0.0747301859	2.0132596724	-0.2123432972	-2.2036993111	-0.8772951230
H	-0.8808027414	-1.3696847274	-1.7557569583	1.0214883141	0.3233344214	0.0000000000
H	0.0000000000	-0.0593231956	-2.5964745936	1.1386646315	1.6427617942	0.0000000000
H	0.8808027414	-1.3696847274	-1.7557569583	-1.8916088277	-0.3223606478	0.0000000000
H	0.0000000000	-1.7729198701	0.7511778371	-0.2490878726	2.2603715408	0.0000000000
H	0.0000000000	1.0072005359	2.0776006513	-2.1034777564	2.1305371508	0.0000000000
H	0.0000000000	-0.6492069158	2.9323300370	1.3445877237	0.3127104100	0.0000000000

Table S42. Frequencies of Methyl Vinyl Ketone in cm^{-1}

CCSD(T)/d-aug-cc-pVTZ	M06-2X/d-aug-cc-pVTZ
108.861	90.135
147.843	150.911
267.829	274.594
411.009	419.329
454.878	462.686
597.886	607.128
678.019	690.341
783.321	792.108
967.414	968.554
985.544	1033.015
1010.785	1037.113
1045.779	1053.188
1076.853	1083.352
1206.289	1210.214
1313.578	1331.339
1392.586	1389.170
1436.344	1438.760
1476.523	1468.930
1485.609	1478.619
1657.541	1712.009
1751.535	1831.672
3030.660	3065.023
3097.662	3126.273
3143.558	3169.680
3155.218	3187.269
3167.523	3193.365

3243.519	3265.260

Table S43. Transition Energies of Methyl Vinyl Ketone in eV

CCSD(T)/d-aug-cc-pVTZ	M06-2X/d-aug-cc-pVTZ
3.796	3.637
6.296	6.019
6.831	6.848
7.427	7.075
7.524	7.227
7.555	7.483
7.610	7.496
7.646	7.525
7.912	7.611
8.091	7.841
	7.944
	8.022
	8.026
	8.099

Table S44. Quantitative Metrics for the Bandwidth (γ), cosine similarity (S), relative integral change (RIC), mean signed error (MSE), and mean average error (MAE) for the band shape of methyl vinyl ketone compared to experiment.

Method	γ	S	RIC	MSE	MAE
B3LYP	0.32	0.826	0.762	11.428	16.084
BH&HLYP	0.36	0.985	0.256	5.127	5.127
BMK	0.33	0.976	0.370	7.656	7.657
CAM-B3LYP	0.33	0.955	0.527	10.246	10.303
CC	0.31	0.991	0.380	-7.840	7.840
HSE	0.32	0.895	0.678	12.603	14.254
M06-2X	0.32	0.975	0.403	8.364	8.364
M11	0.33	0.979	0.360	8.037	8.037
PBE0	0.32	0.901	0.675	12.834	14.177
wB97x-D	0.33	0.957	0.513	10.141	10.194
X3LYP	0.33	0.845	0.729	11.490	15.384

Figure S11. Spectra of Methyl Vinyl Ketone

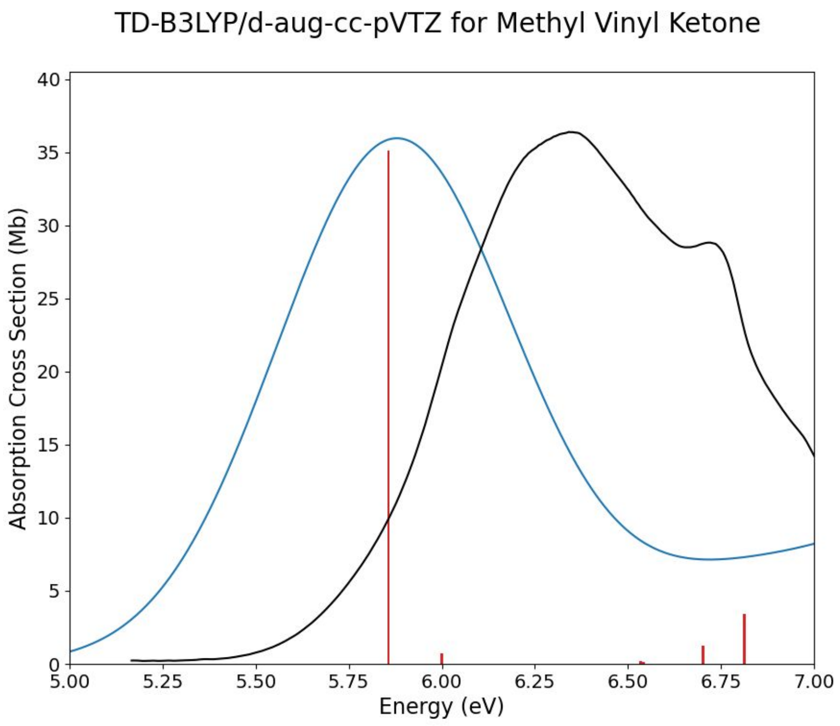

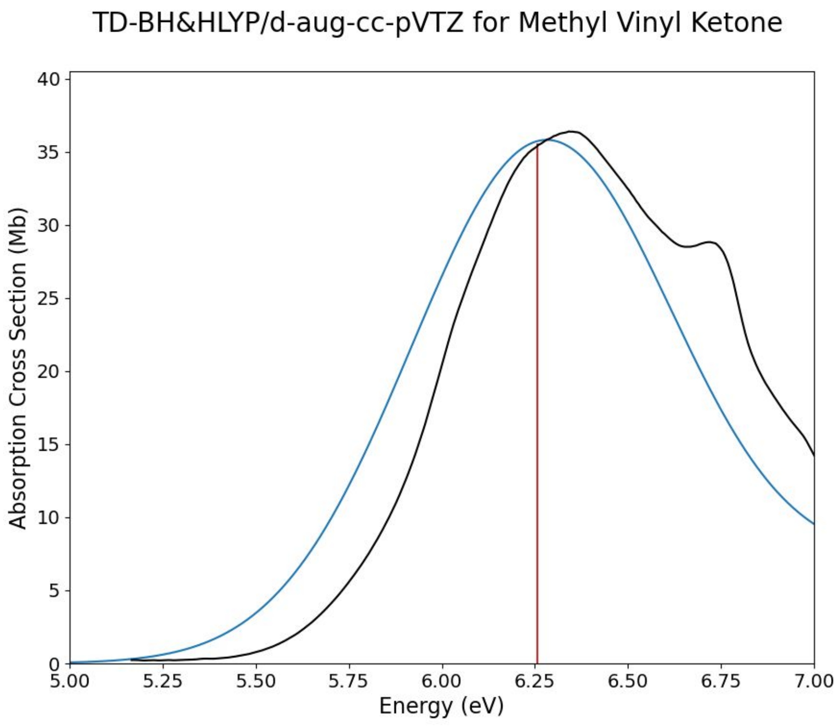

TD-BMK/d-aug-cc-pVTZ for Methyl Vinyl Ketone

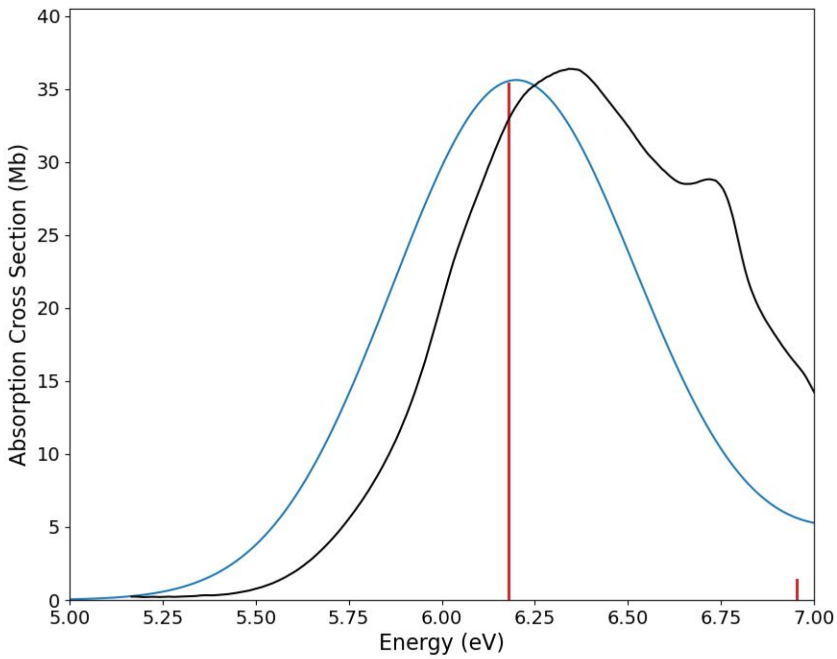

TD-CAM-B3LYP/d-aug-cc-pVTZ for Methyl Vinyl Ketone

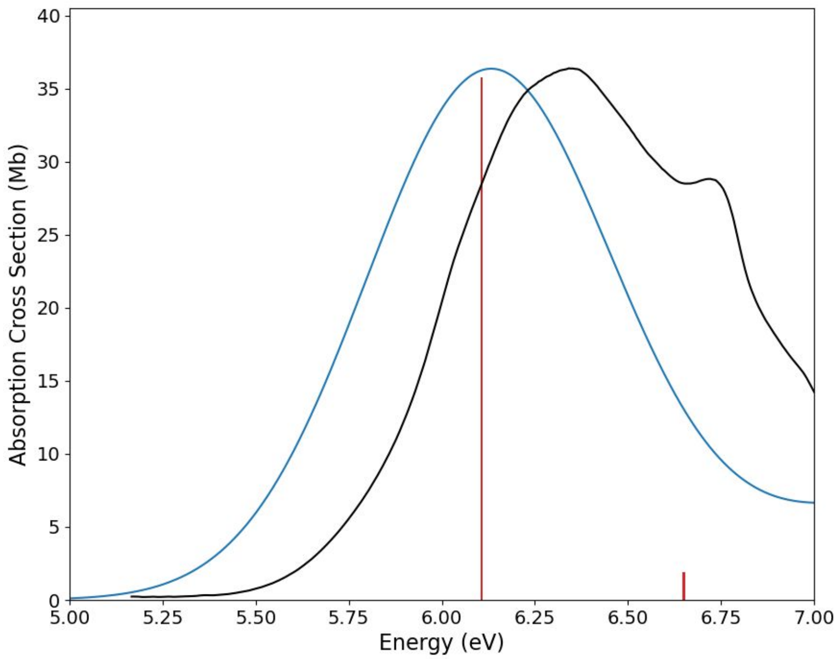

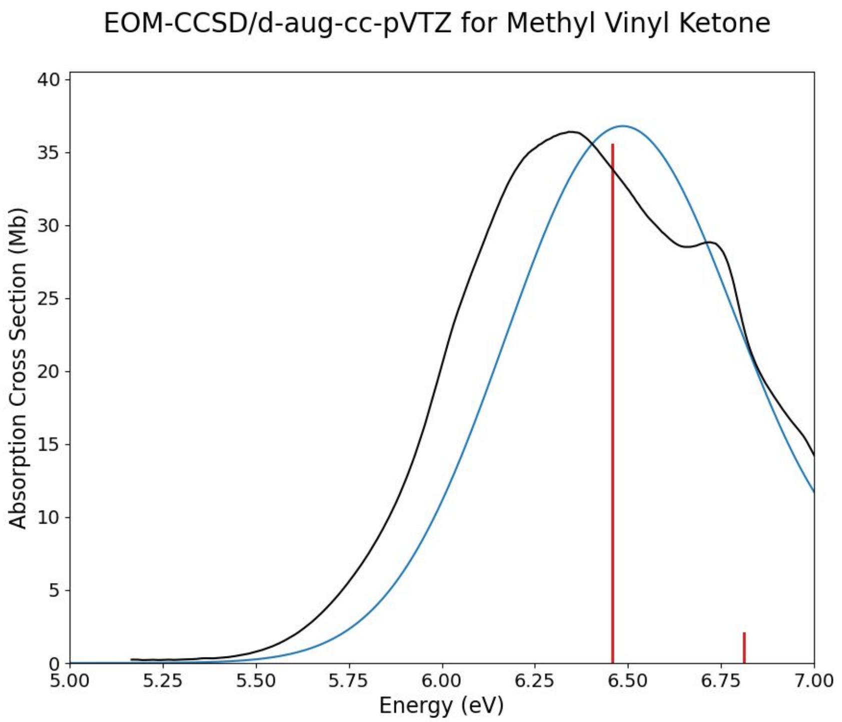
EOM-CCSD/d-aug-cc-pVTZ for Methyl Vinyl Ketone
Absorption Cross Section (Mb)
Energy (eV)
0
5
10
15
20
25
30
35
40
5.00
5.25
5.50
5.75
6.00
6.25
6.50
6.75
7.00

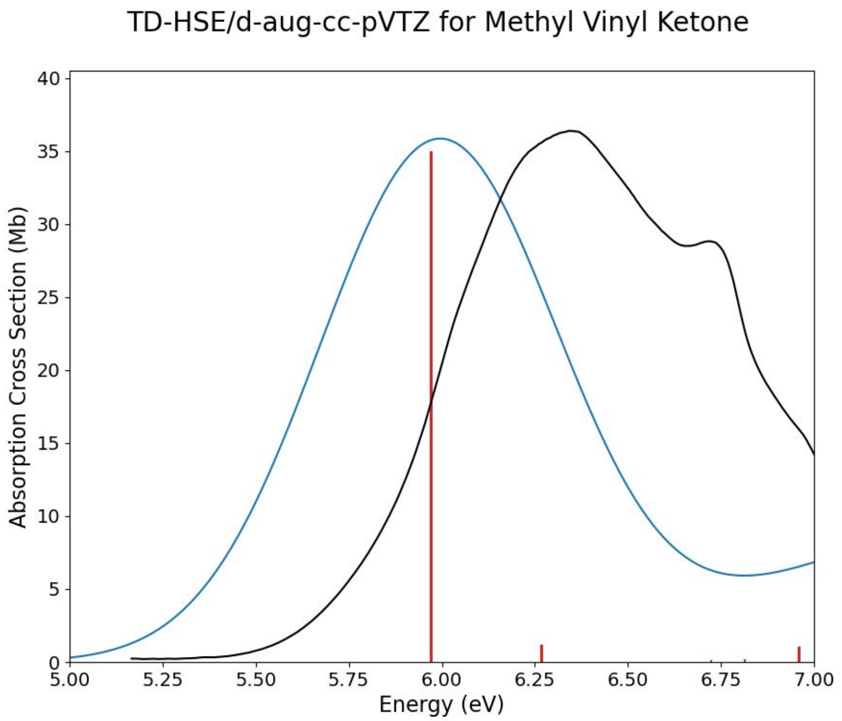
TD-HSE/d-aug-cc-pVTZ for Methyl Vinyl Ketone
Absorption Cross Section (Mb)
Energy (eV)
0
5
10
15
20
25
30
35
40
5.00
5.25
5.50
5.75
6.00
6.25
6.50
6.75
7.00

TD-M06-2X/d-aug-cc-pVTZ for Methyl Vinyl Ketone

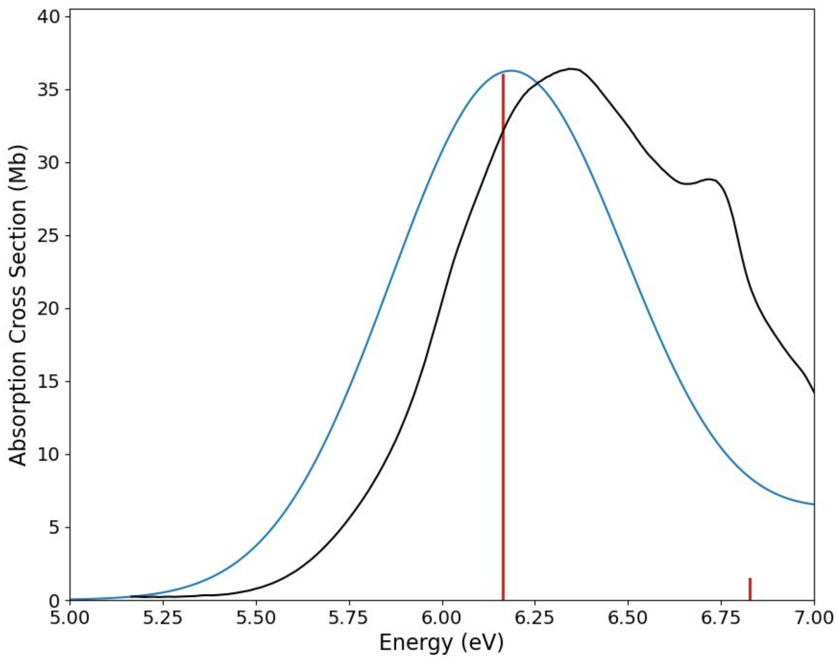

TD-M11/d-aug-cc-pVTZ for Methyl Vinyl Ketone

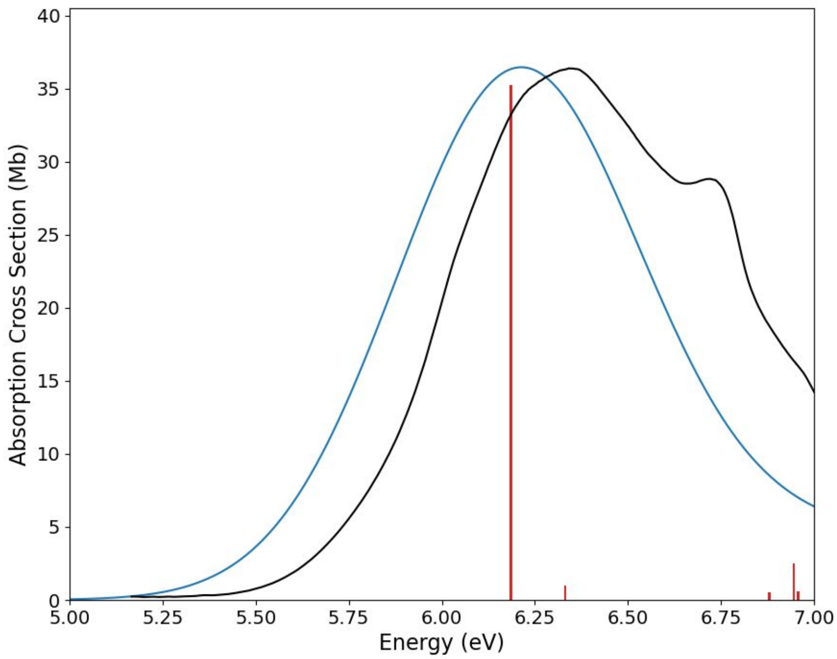

TD-PBE0/d-aug-cc-pVTZ for Methyl Vinyl Ketone

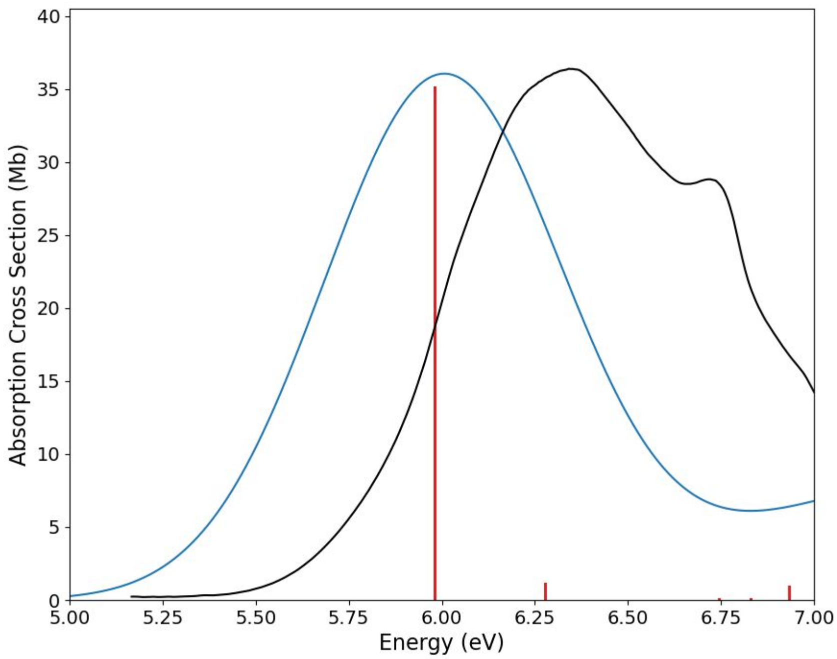

TD-wB97x-D/d-aug-cc-pVTZ for Methyl Vinyl Ketone

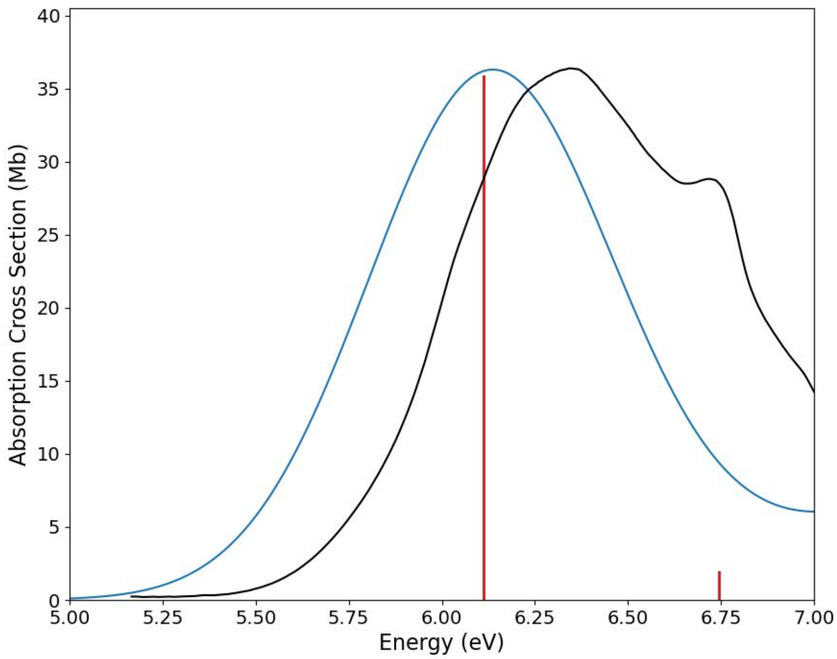

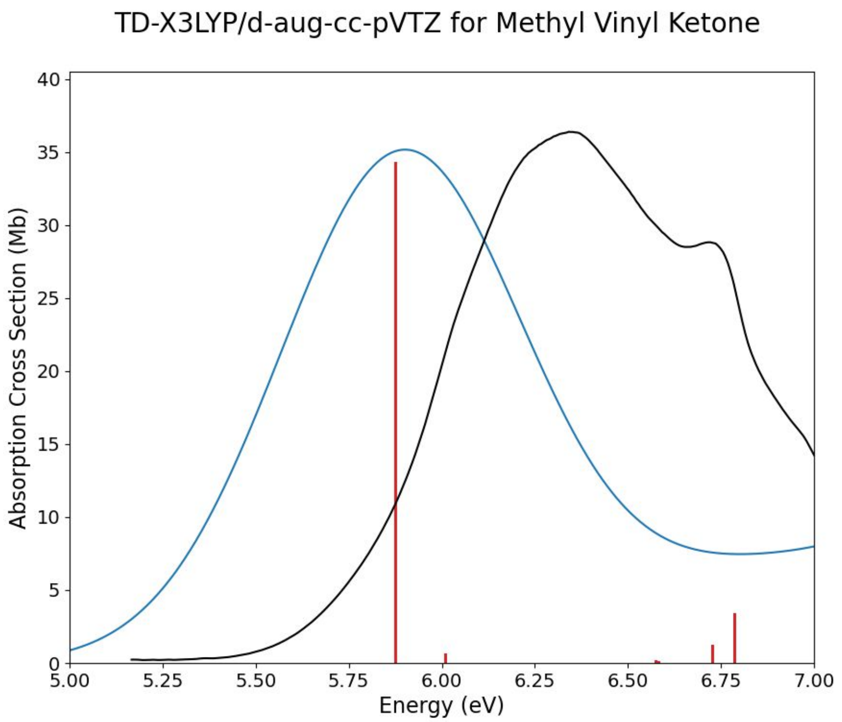

TD-X3LYP/d-aug-cc-pVTZ for Methyl Vinyl Ketone
Absorption Cross Section (Mb)
0
5
10
15
20
25
30
35
40
Energy (eV)
5.00
5.25
5.50
5.75
6.00
6.25
6.50
6.75
7.00

12 Formic Acid

Table S45. Optimized Geometry of Formic Acid in Å

	CCSD(T)/d-aug-cc-pVTZ			M06-2X/d-aug-cc-pVTZ		
C	0.0000000000	-0.4145197396	-0.0992163846	-0.0334818356	0.4227177078	0.0000000000
O	0.0000000000	0.1221850047	1.1373000306	-1.0334607538	-0.4658878967	0.0000000000
O	0.0000000000	0.2155933470	-1.1251494246	1.1263993611	0.1430612087	0.0000000000
H	0.0000000000	-1.5074498044	-0.0311390610	-0.4340927188	1.4416354275	0.0000000000
H	0.0000000000	1.0853469333	1.0205685803	-0.6422479411	-1.3513689488	0.0000000000

Table S46. Frequencies of Formic Acid in cm^{-1}

CCSD(T)/d-aug-cc-pVTZ	M06-2X/d-aug-cc-pVTZ
626.070	644.621
666.441	668.793
1050.692	1074.080
1130.820	1162.544
1311.727	1317.235
1403.106	1413.431
1802.186	1867.726
3088.516	3098.522
3741.376	3789.911

Table S47. Transition Energies of Formic Acid in eV

CCSD(T)/d-aug-cc-pVTZ	M06-2X/d-aug-cc-pVTZ
5.839	5.669
7.898	7.850
8.433	8.349
8.685	8.497
8.690	8.705
9.064	8.850
9.209	9.010
	9.327
	9.364
	9.533

Table S48. Quantitative Metrics for the Bandwidth (γ), cosine similarity (S), relative integral change (RIC), mean signed error (MSE), and mean average error (MAE) for the band shape of formic acid compared to experiment.

Method	γ	S	RIC	MSE	MAE
B3LYP	0.25	0.970	2.146	4.699	4.699
BH&HLYP	0.65	0.996	0.479	-1.040	1.040
BMK	0.65	0.997	0.270	-0.570	0.570
CAM-B3LYP	0.35	0.997	0.155	0.328	0.328
CC	0.45	0.978	0.537	-1.171	1.171
HSE	0.45	0.994	0.976	2.129	2.129
M06-2X	0.65	0.997	0.073	0.121	0.164
M11	0.23	0.999	0.427	0.918	0.918
PBE0	0.23	1.000	1.114	2.410	2.410
wB97x-D	0.4	0.995	0.086	0.005	0.187
X3LYP	0.3	0.974	2.018	4.429	4.429

Figure S12. Spectra of Formic Acid

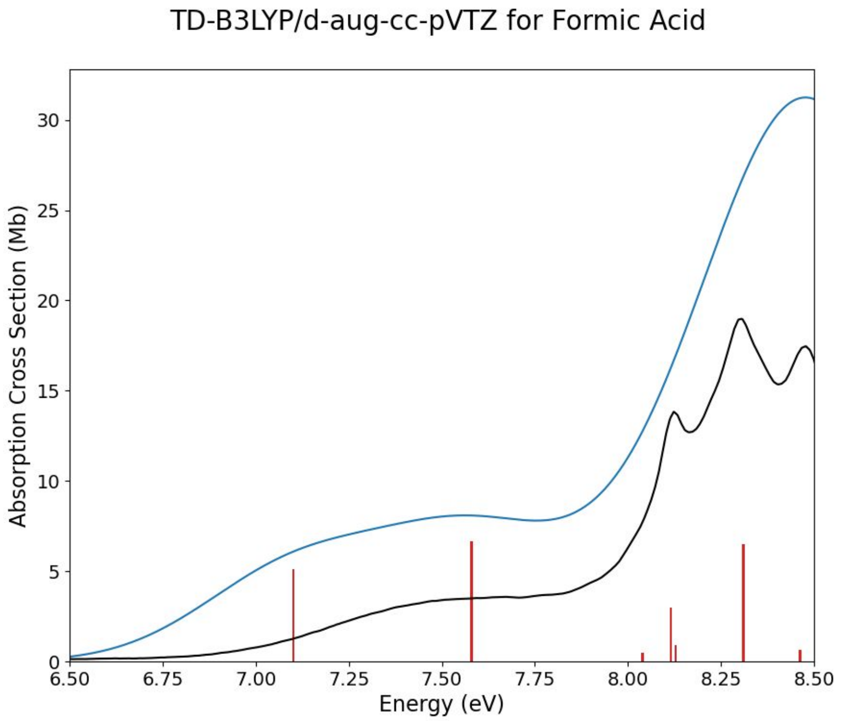

TD-BH&HYLP/d-aug-cc-pVTZ for Formic Acid

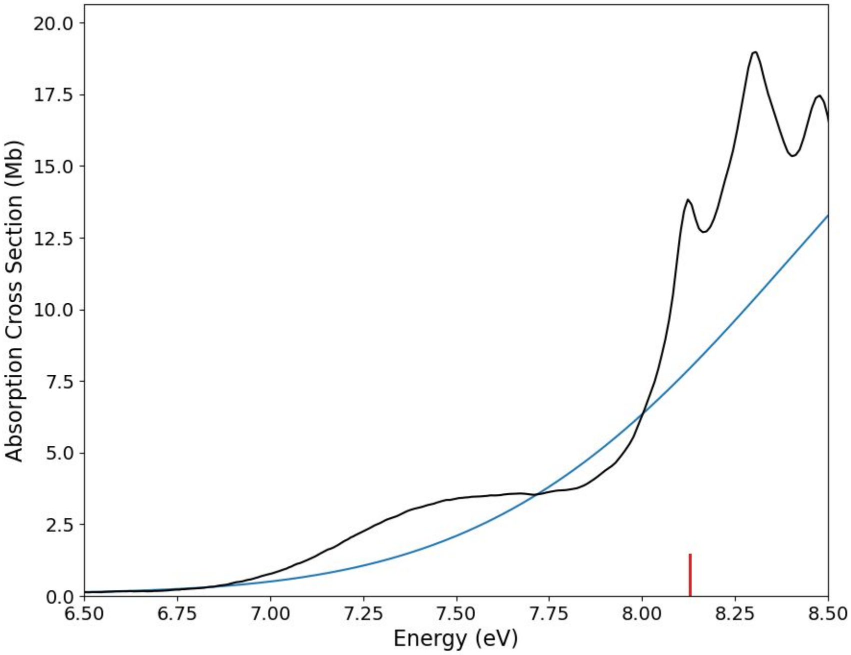

TD-BMK/d-aug-cc-pVTZ for Formic Acid

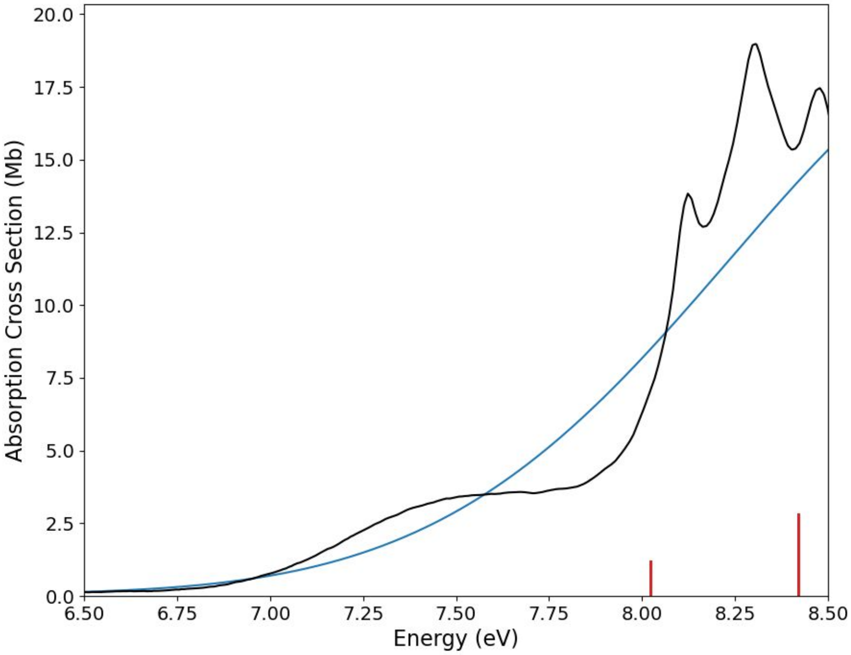

TD-CAM-B3LYP/d-aug-cc-pVTZ for Formic Acid

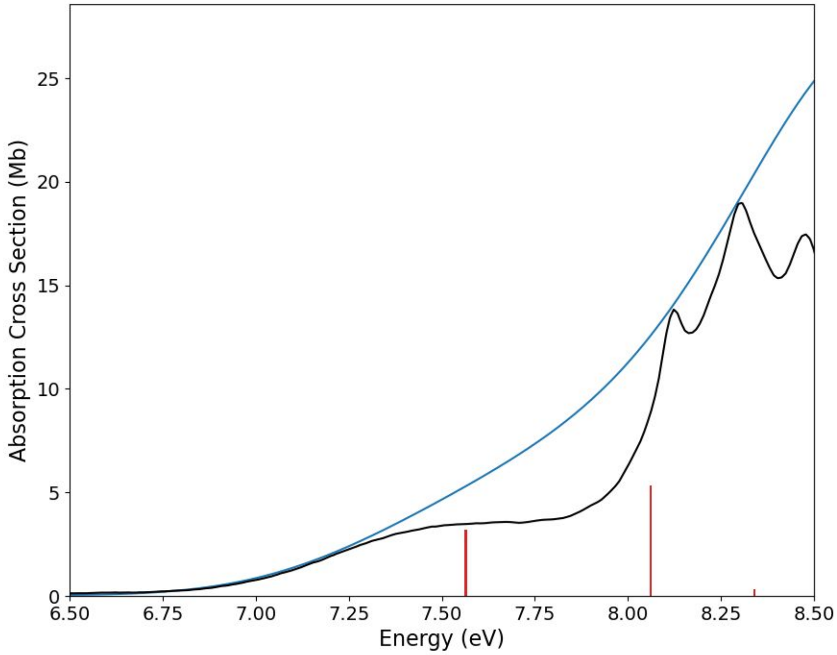

EOM-CCSD/d-aug-cc-pVTZ for Formic Acid

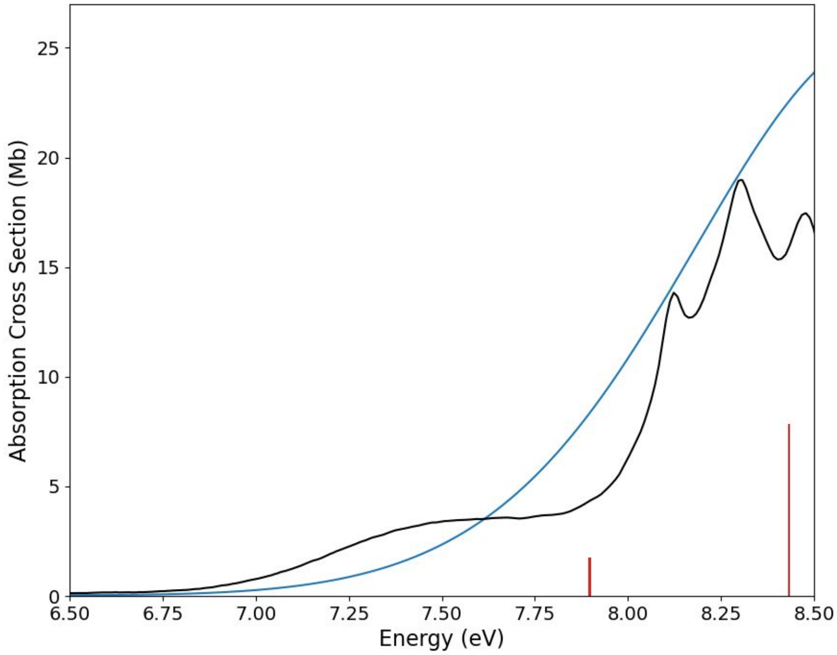

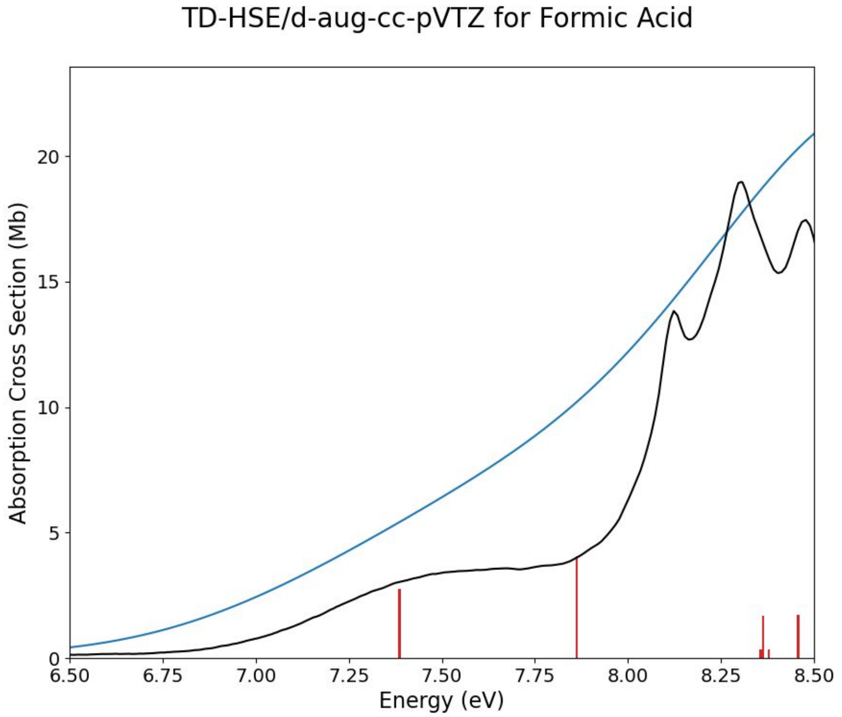
TD-HSE/d-aug-cc-pVTZ for Formic Acid
Absorption Cross Section (Mb)
Energy (eV)
0
5
10
15
20
6.50
6.75
7.00
7.25
7.50
7.75
8.00
8.25
8.50

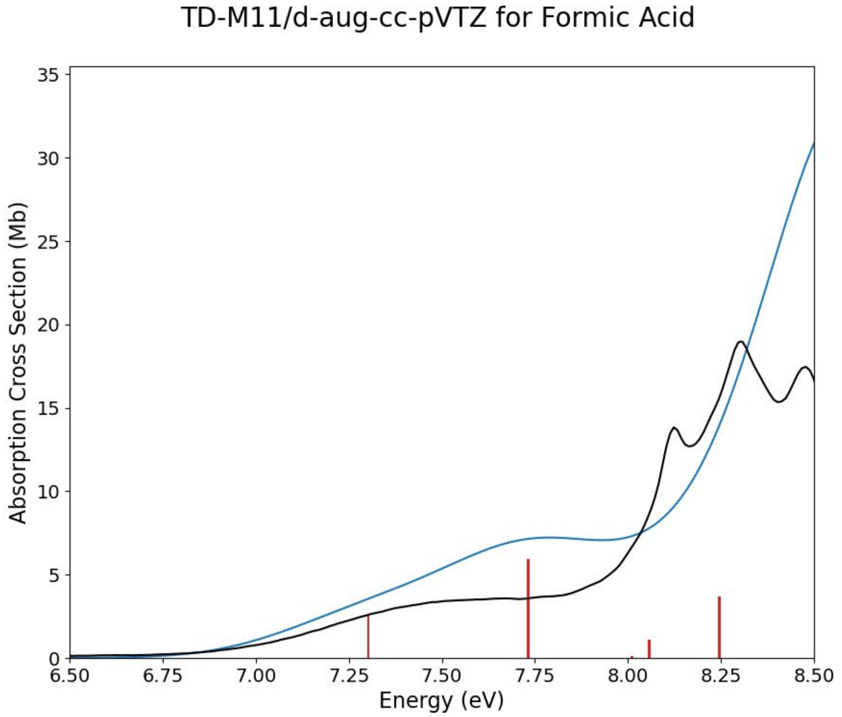
TD-M11/d-aug-cc-pVTZ for Formic Acid
Absorption Cross Section (Mb)
Energy (eV)
0
5
10
15
20
25
30
35
6.50
6.75
7.00
7.25
7.50
7.75
8.00
8.25
8.50

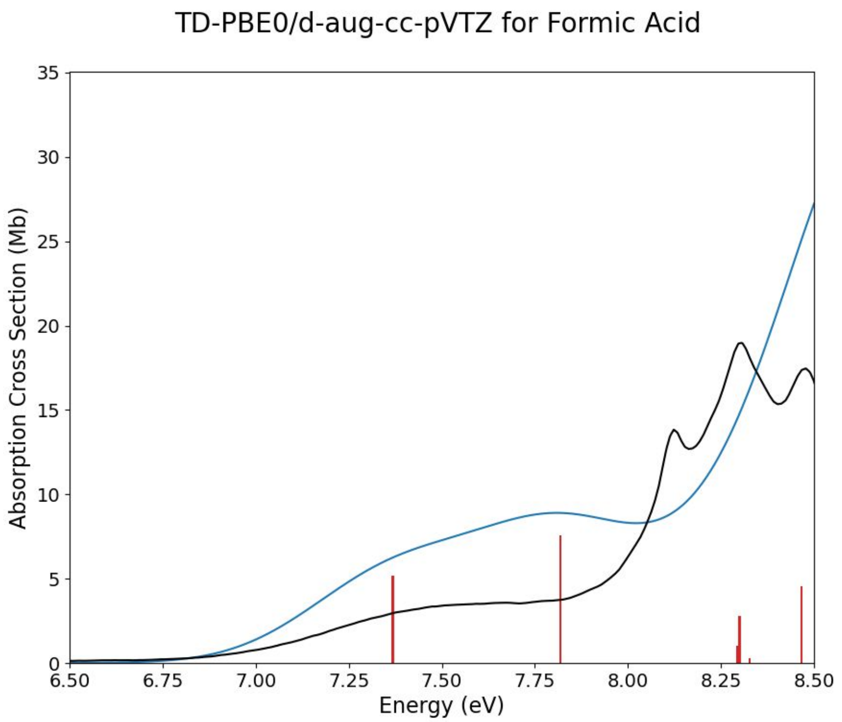

TD-PBE0/d-aug-cc-pVTZ for Formic Acid
Absorption Cross Section (Mb)
Energy (eV)
0
5
10
15
20
25
30
35
6.50
6.75
7.00
7.25
7.50
7.75
8.00
8.25
8.50

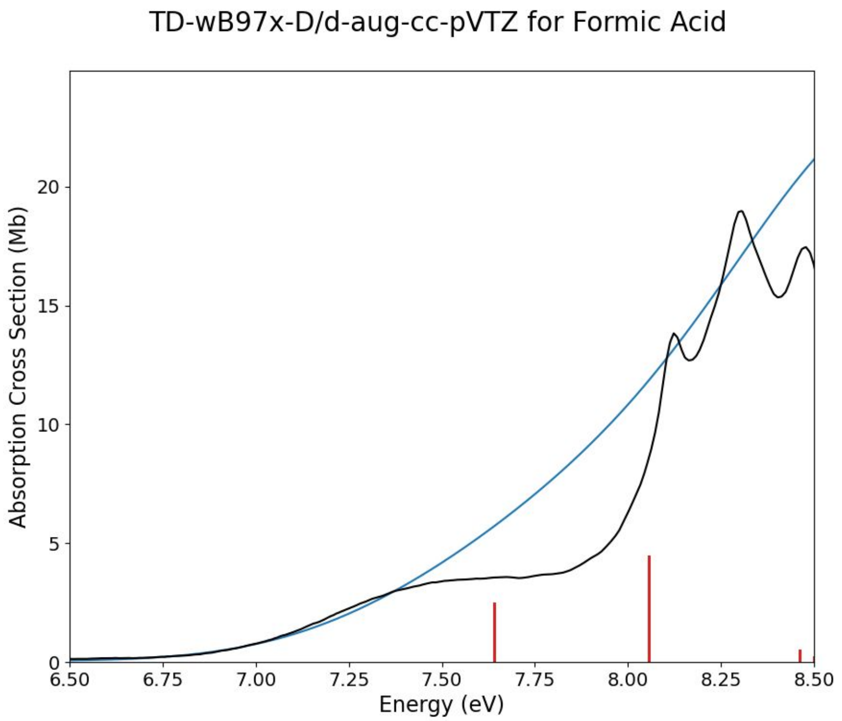

TD-wB97x-D/d-aug-cc-pVTZ for Formic Acid
Absorption Cross Section (Mb)
Energy (eV)
0
5
10
15
20
6.50
6.75
7.00
7.25
7.50
7.75
8.00
8.25
8.50

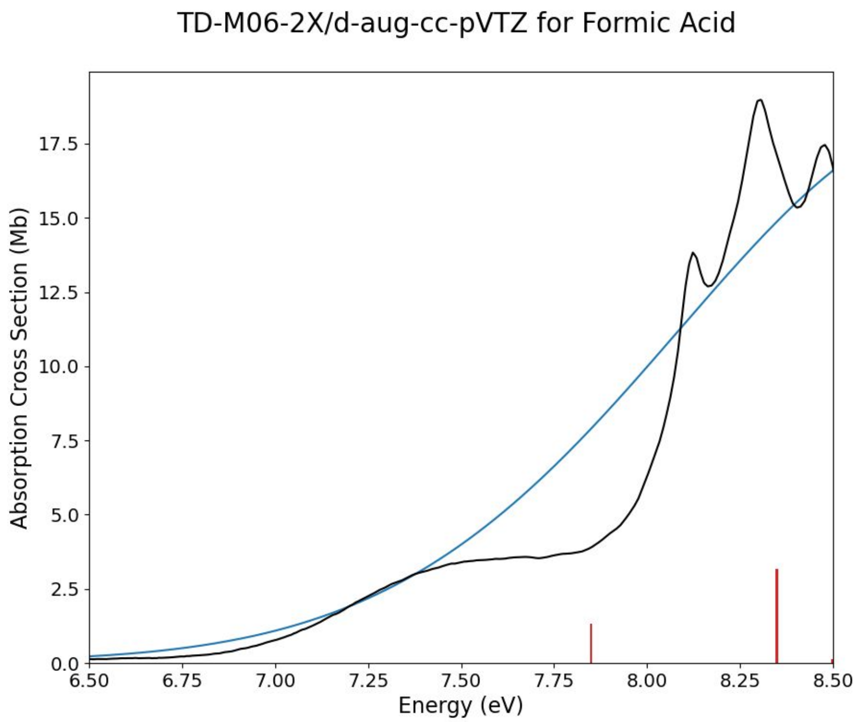
TD-M06-2X/d-aug-cc-pVTZ for Formic Acid
Absorption Cross Section (Mb)
0.0
2.5
5.0
7.5
10.0
12.5
15.0
17.5
6.50
6.75
7.00
7.25
7.50
7.75
8.00
8.25
8.50
Energy (eV)

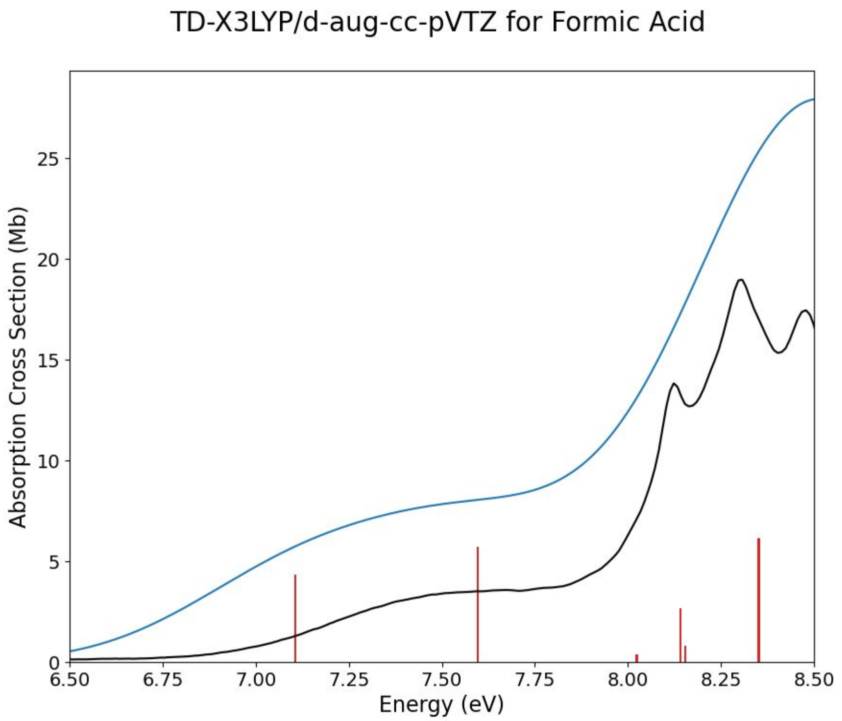
TD-X3LYP/d-aug-cc-pVTZ for Formic Acid
Absorption Cross Section (Mb)
0
5
10
15
20
25
6.50
6.75
7.00
7.25
7.50
7.75
8.00
8.25
8.50
Energy (eV)

13 Acetic Acid

Table S49. Optimized Geometry of Acetic Acid in Å

	CCSD(T)/d-aug-cc-pVTZ			M06-2X/d-aug-cc-pVTZ		
O	0.0000000000	-1.1104276079	-0.6524547130	1.1449096563	-0.8956325524	0.0000000000
C	0.0000000000	0.1015334375	0.0387162166	0.0285183629	0.1038786805	0.0000000000
O	0.0000000000	1.1408112647	-0.6559747915	0.1489366607	1.2964556946	0.0000000000
C	0.0000000000	-0.0418522194	1.4584115816	2.0963245360	-0.3763274147	0.0000000000
H	0.0000000000	-0.9264762020	-1.6039169647	1.0594408407	-1.5333156169	0.8782842128
H	0.0000000000	0.9436290181	1.9167169937	1.0594408407	-1.5333156169	-0.8782842128
H	0.8814212066	-0.6053140066	1.7693723446	-1.1785852826	-0.4981940001	0.0000000000
H	-0.8814212066	-0.6053140066	1.7693723446	-1.8457417887	0.2012439860	0.0000000000

Table S50. Frequencies of Acetic Acid in cm^{-1}

CCSD(T)/d-aug-cc-pVTZ	M06-2X/d-aug-cc-pVTZ
85.408	38.807
418.418	427.641
542.830	546.987
582.187	597.202
656.553	663.377
865.864	885.682
1006.603	1008.991
1073.774	1074.932
1215.580	1227.112
1348.178	1349.879
1421.080	1422.701
1484.622	1475.594
1490.860	1479.935
1816.992	1871.948
3058.621	3091.034
3131.488	3157.853
3175.074	3205.186
3754.941	3808.103

Table S51. Transition Energies of Acetic Acid in eV

CCSD(T)/d-aug-cc-pVTZ	M06-2X/d-aug-cc-pVTZ
5.943	5.760
7.449	7.422
8.280	8.158
8.420	8.288
8.591	8.413
8.740	8.556
8.749	8.763

	8.925

Table S52. Quantitative Metrics for the Bandwidth (γ), cosine similarity (S), relative integral change (RIC), mean signed error (MSE), and mean average error (MAE) for the band shape of acetic acid compared to experiment.

Method	γ	S	RIC	MSE	MAE
B3LYP	0.3	0.640	0.795	-0.071	2.821
BH&HLYP	0.25	0.861	0.889	-3.111	3.111
BMK	0.25	0.897	0.783	-2.743	2.743
CAM-B3LYP	0.35	0.962	0.391	1.411	1.439
CC	0.3	0.986	0.350	-1.227	1.227
HSE	0.3	0.846	0.753	2.157	2.760
M06-2X	0.3	0.990	0.317	-1.118	1.118
M11	0.27	0.839	0.570	0.550	2.060
PBE0	0.3	0.838	0.739	1.939	2.702
wB97x-D	0.33	0.989	0.189	0.687	0.688
X3LYP	0.33	0.693	0.739	-0.086	2.671

Figure S13. Spectra of Acetic Acid

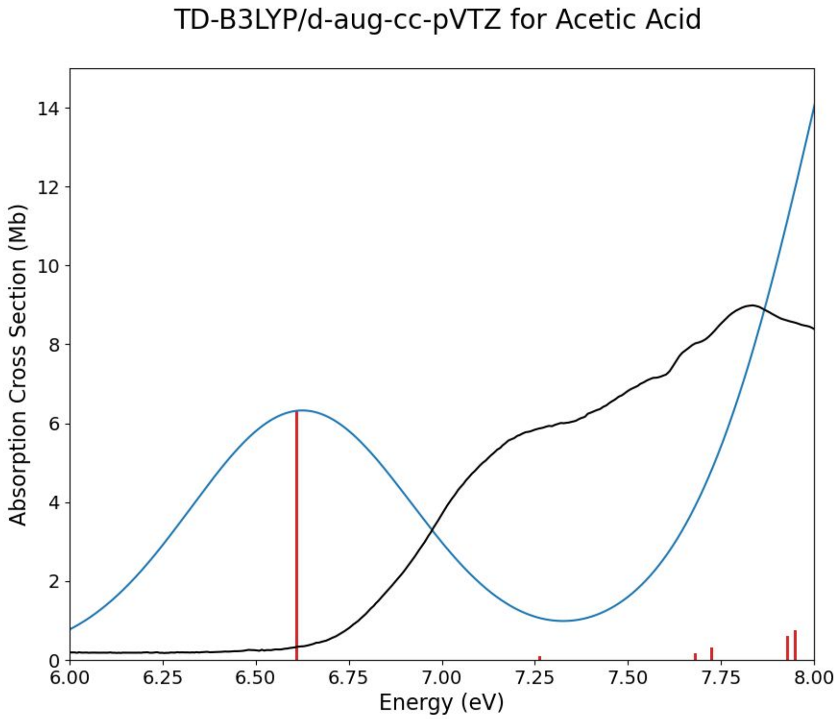

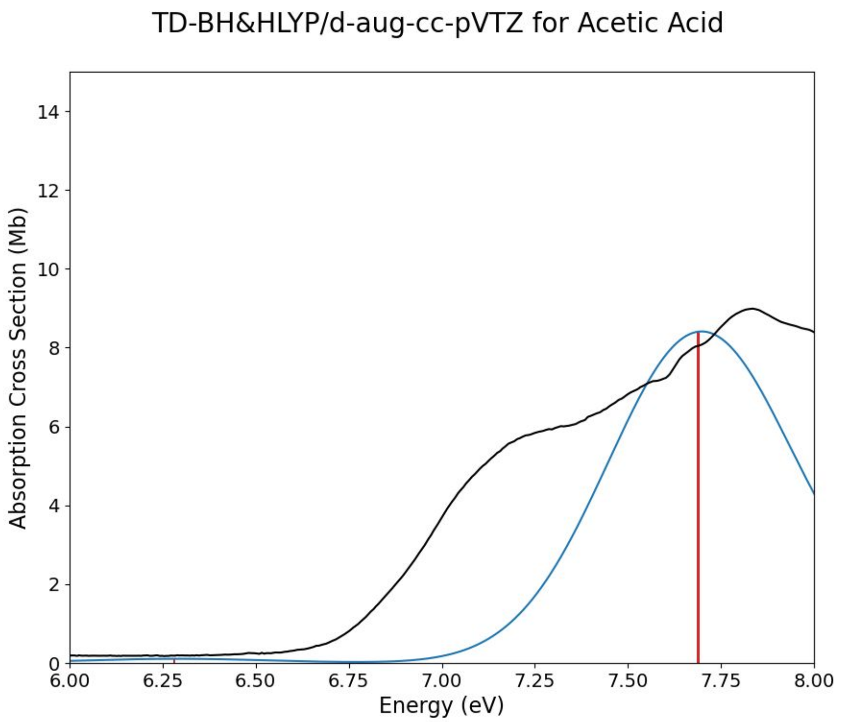
TD-BH&HLYP/d-aug-cc-pVTZ for Acetic Acid
Absorption Cross Section (Mb)
Energy (eV)
0
2
4
6
8
10
12
14
6.00
6.25
6.50
6.75
7.00
7.25
7.50
7.75
8.00

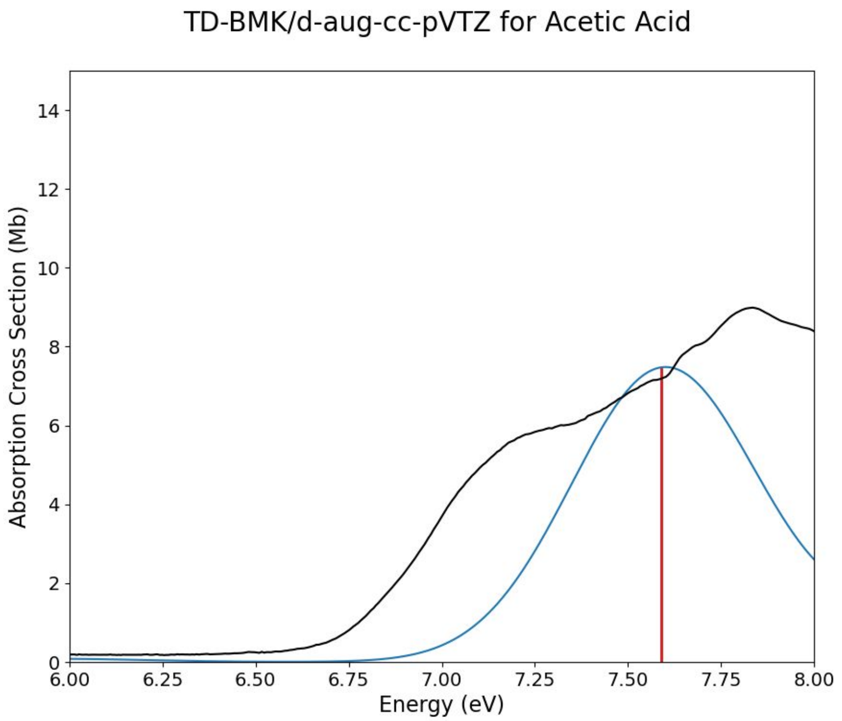
TD-BMK/d-aug-cc-pVTZ for Acetic Acid
Absorption Cross Section (Mb)
Energy (eV)
0
2
4
6
8
10
12
14
6.00
6.25
6.50
6.75
7.00
7.25
7.50
7.75
8.00

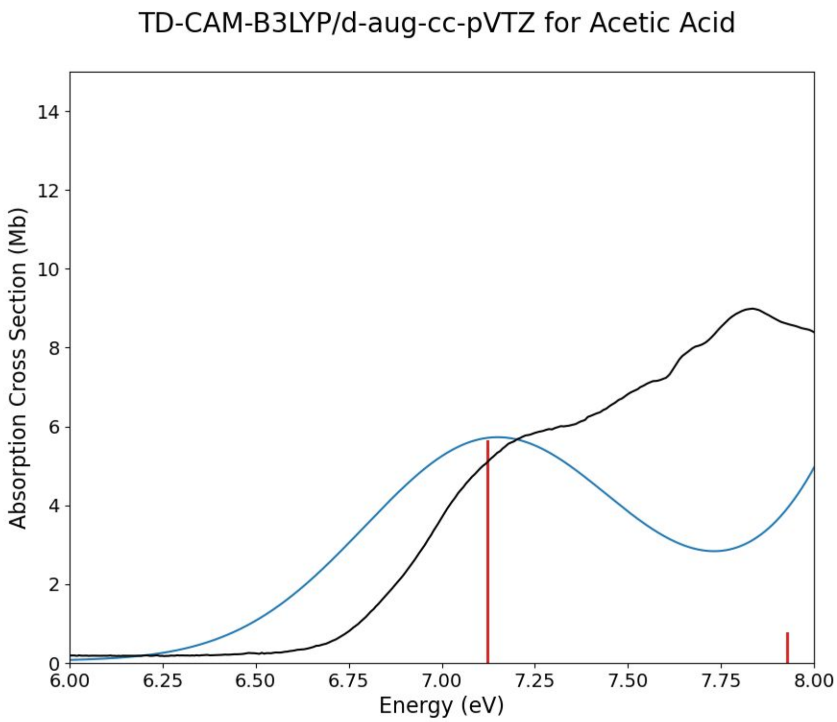
TD-CAM-B3LYP/d-aug-cc-pVTZ for Acetic Acid
Absorption Cross Section (Mb)
Energy (eV)
0
2
4
6
8
10
12
14
6.00
6.25
6.50
6.75
7.00
7.25
7.50
7.75
8.00

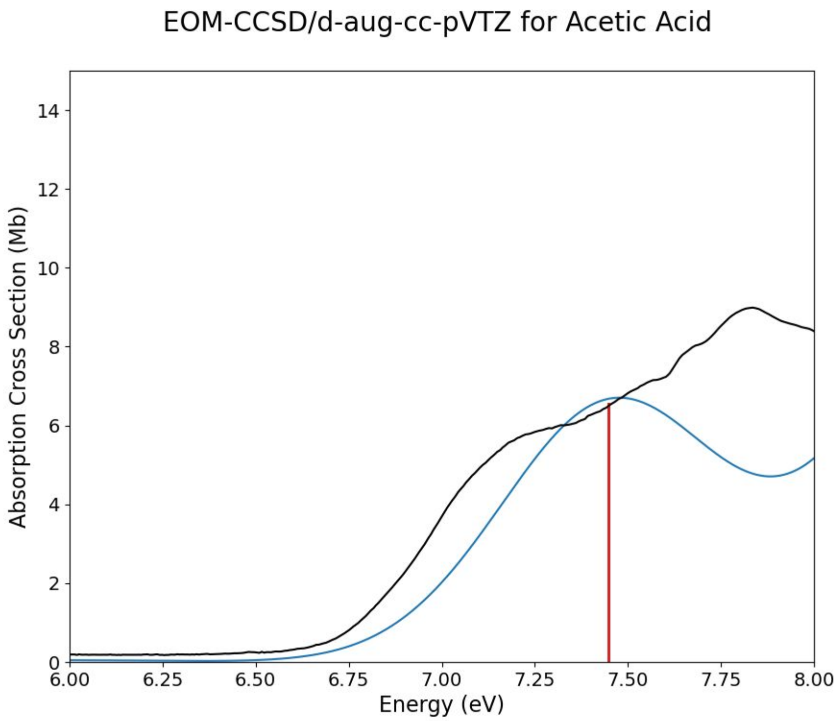
EOM-CCSD/d-aug-cc-pVTZ for Acetic Acid
Absorption Cross Section (Mb)
Energy (eV)
0
2
4
6
8
10
12
14
6.00
6.25
6.50
6.75
7.00
7.25
7.50
7.75
8.00

TD-HSE/d-aug-cc-pVTZ for Acetic Acid

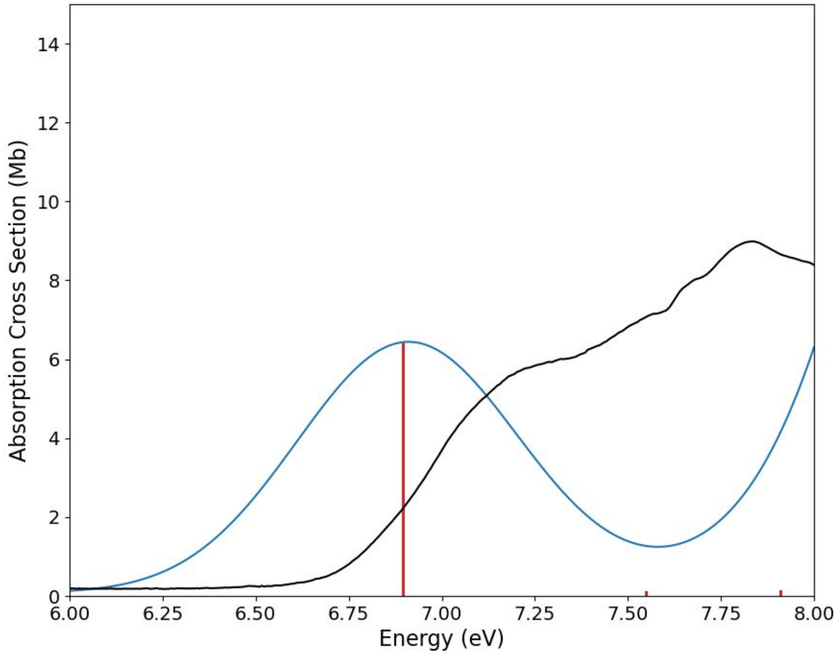

TD-M06-2X/d-aug-cc-pVTZ for Acetic Acid

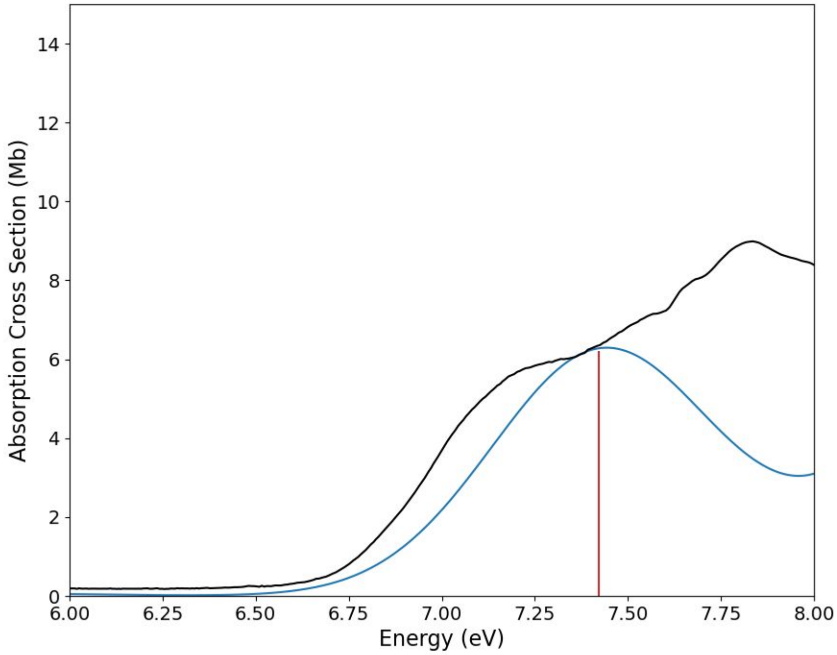

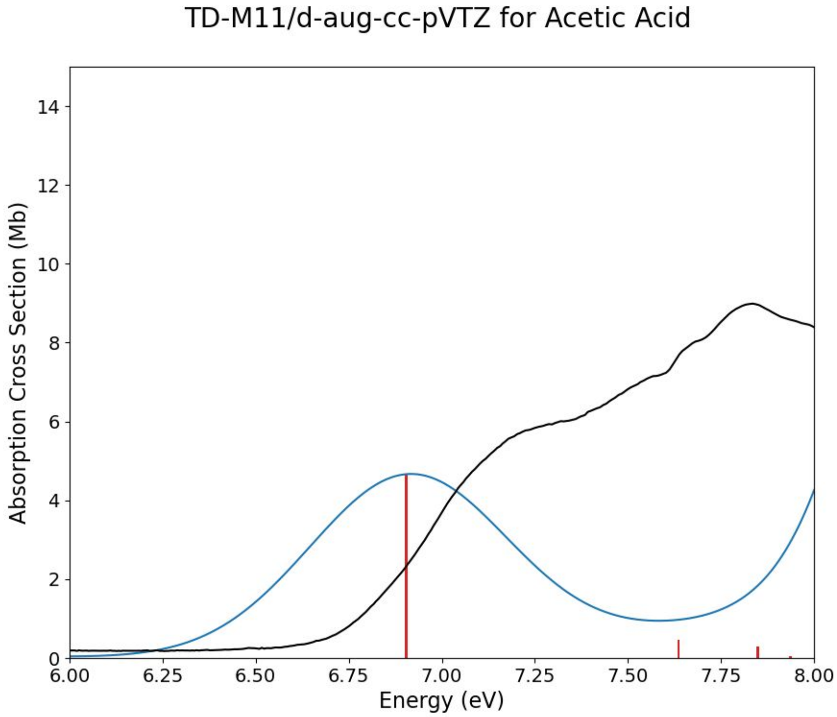
TD-M11/d-aug-cc-pVTZ for Acetic Acid
Absorption Cross Section (Mb)
Energy (eV)
0
2
4
6
8
10
12
14
6.00
6.25
6.50
6.75
7.00
7.25
7.50
7.75
8.00

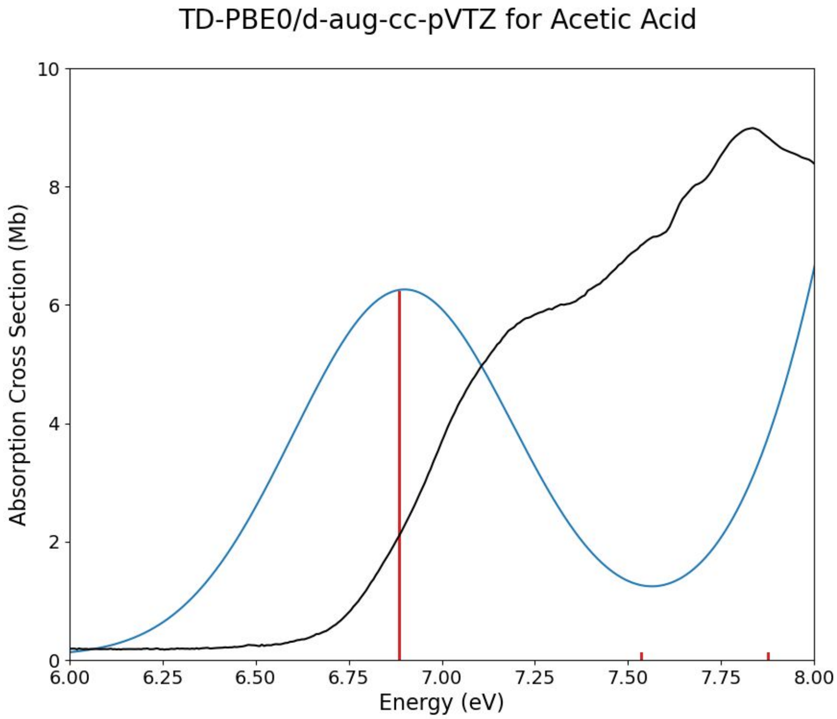
TD-PBE0/d-aug-cc-pVTZ for Acetic Acid
Absorption Cross Section (Mb)
Energy (eV)
0
2
4
6
8
10
6.00
6.25
6.50
6.75
7.00
7.25
7.50
7.75
8.00

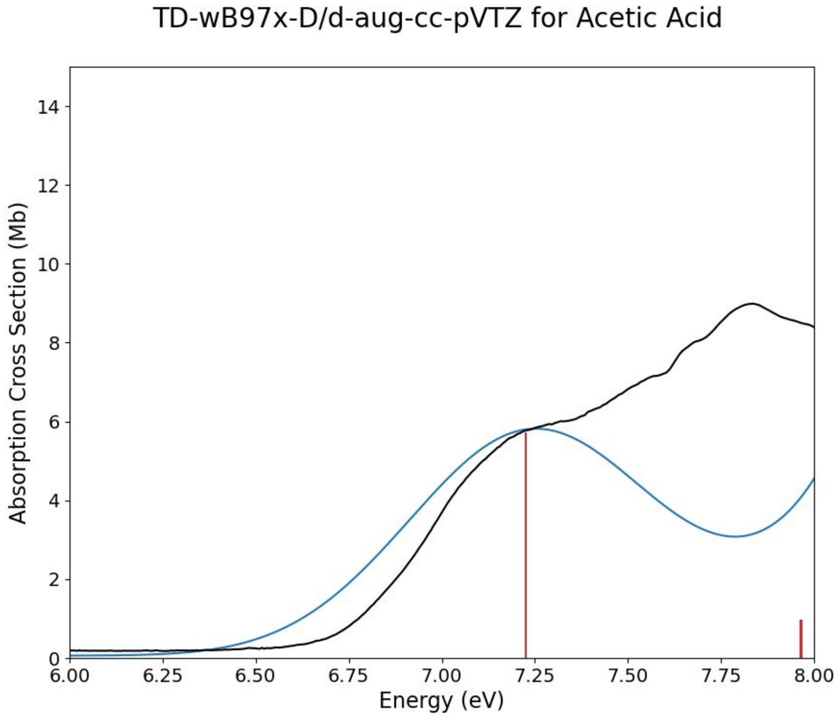
TD-wB97x-D/d-aug-cc-pVTZ for Acetic Acid
Absorption Cross Section (Mb)
Energy (eV)
0
2
4
6
8
10
12
14
6.00
6.25
6.50
6.75
7.00
7.25
7.50
7.75
8.00

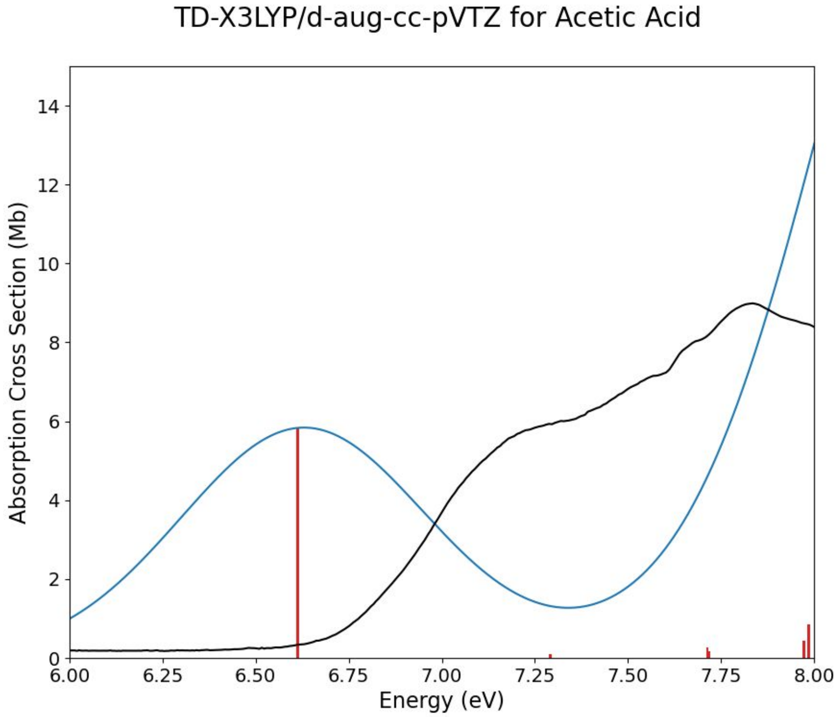
TD-X3LYP/d-aug-cc-pVTZ for Acetic Acid
Absorption Cross Section (Mb)
Energy (eV)
0
2
4
6
8
10
12
14
6.00
6.25
6.50
6.75
7.00
7.25
7.50
7.75
8.00

14 Propionic Acid

Table S53. Optimized Geometry of Propionic Acid in Å

	CCSD(T)/d-aug-cc-pVTZ			M06-2X/d-aug-cc-pVTZ		
O	0.0000000000	-0.6797213092	-1.6083345097	-0.0284775695	0.4869083253	0.0000000000
C	0.0000000000	0.0808047954	-0.4827804588	-0.5852109615	-0.9099551626	0.0000000000
O	0.0000000000	1.2896254293	-0.5168359866	0.5036499639	-1.9673131580	0.0000000000
C	0.0000000000	-0.7740377747	0.7608337427	-1.0066869282	1.4155029143	0.0000000000
C	0.0000000000	0.0578828222	2.0380546390	1.1339939599	0.7814928325	0.0000000000
H	0.0000000000	-0.0567650713	-2.3506750998	-1.2383742627	-0.9965554074	0.8701141598
H	0.8739052967	-1.4300036287	0.7042026477	-1.2383742627	-0.9965554074	-0.8701141598
H	-0.8739052967	-1.4300036287	0.7042026477	0.0662280643	-2.9636560203	0.0000000000
H	0.0000000000	-0.5942815689	2.9135025402	1.1390696972	-1.8656044102	-0.8773734232
H	-0.8807840791	0.7004496424	2.0813720332	1.1390696972	-1.8656044102	0.8773734232
H	0.8807840791	0.7004496424	2.0813720332	-0.5778635443	2.2816657896	0.0000000000

Table S54. Frequencies of Propionic Acid in cm^{-1}

CCSD(T)/d-aug-cc-pVTZ	M06-2X/d-aug-cc-pVTZ
58.186	71.050
214.328	218.347
249.565	257.273
464.913	474.510
518.209	519.847
610.820	624.936
644.022	646.808
809.848	817.374
821.536	837.365
1016.357	1020.770
1094.209	1103.448
1115.799	1116.714
1173.601	1190.978
1289.926	1286.116
1313.171	1310.524
1414.274	1417.605
1427.800	1430.199
1472.112	1466.800
1503.608	1499.217
1511.037	1507.382
1811.082	1865.751
3046.852	3080.443
3049.956	3083.935
3085.852	3112.553
3124.402	3157.965
3129.306	3159.282

3754.308	3808.455

Table S55. Transition Energies of Propionic Acid in eV

CCSD(T)/d-aug-cc-pVTZ	M06-2X/d-aug-cc-pVTZ
5.973	5.792
7.447	7.430
8.219	8.065
8.405	8.292
8.487	8.313
8.612	8.428
8.726	8.762
9.077	8.842
	9.004
	9.063
	9.067

Table S56. Quantitative Metrics for the Bandwidth (γ), cosine similarity (S), relative integral change (RIC), mean signed error (MSE), and mean average error (MAE) for the band shape of propionic acid compared to experiment.

Method	γ	S	RIC	MSE	MAE
B3LYP	0.50	0.915	1.224	2.159	2.159
BH&HLYP	0.50	0.986	0.147	-0.195	0.310
BMK	0.45	0.986	0.129	-0.098	0.311
CAM-B3LYP	0.50	0.969	1.137	2.046	2.046
CC	0.50	0.992	0.568	0.976	0.976
HSE	0.50	0.935	1.315	2.146	2.146
M06-2X	0.50	0.991	0.412	0.791	0.791
M11	0.47	0.937	0.555	1.170	1.170
PBE0	0.50	0.934	1.243	2.347	2.347
wB97x-D	0.50	0.983	0.839	1.608	1.608
X3LYP	0.50	0.907	1.229	2.224	2.224

Figure S14. Spectra of Propionic Acid

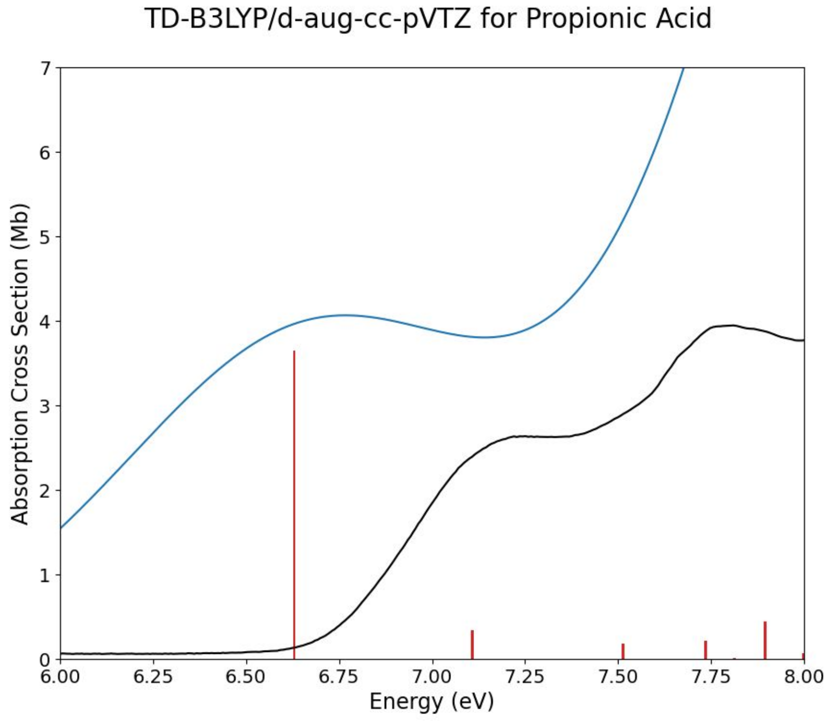

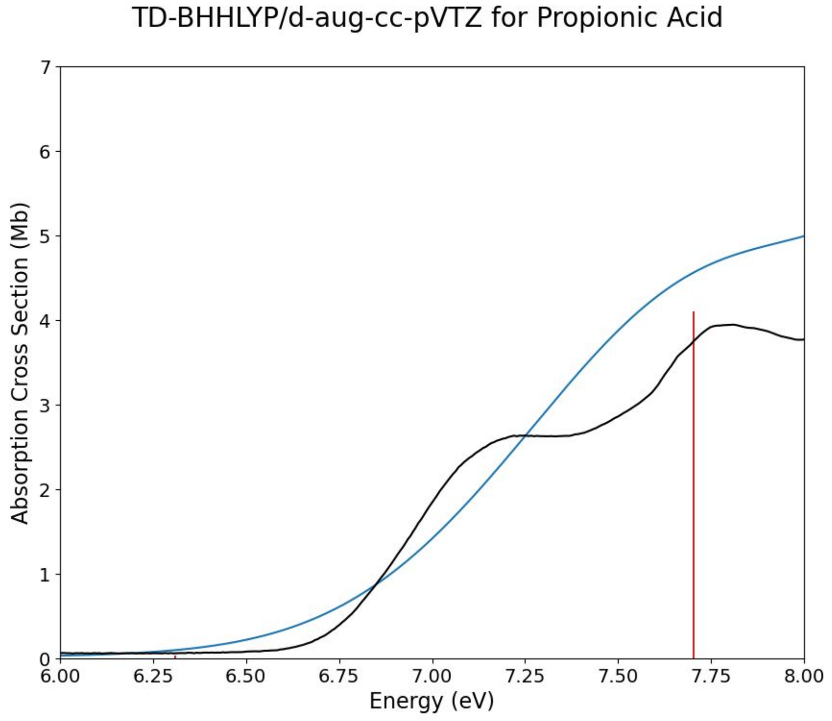

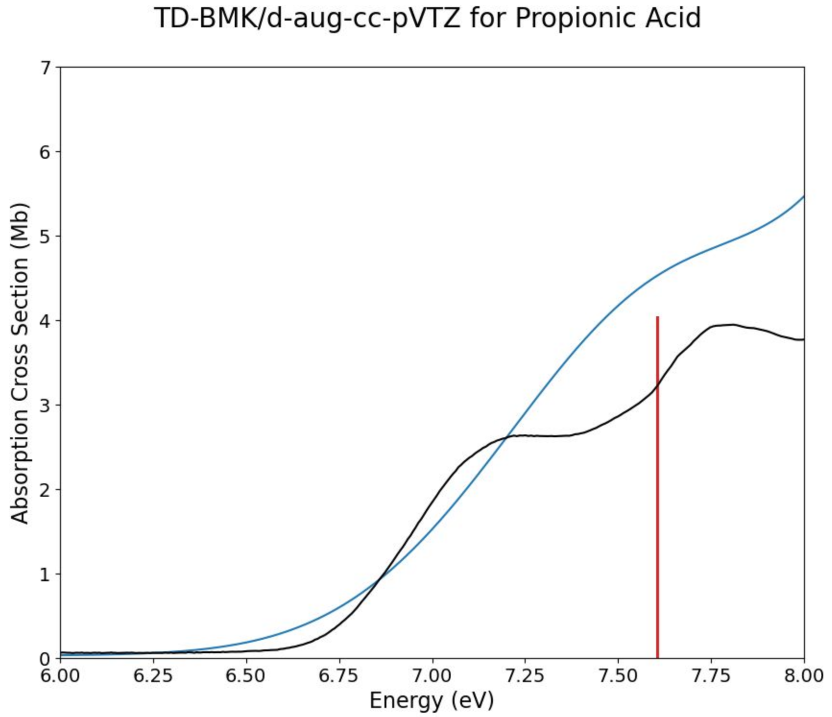
TD-BMK/d-aug-cc-pVTZ for Propionic Acid
Absorption Cross Section (Mb)
Energy (eV)
0
1
2
3
4
5
6
7
6.00
6.25
6.50
6.75
7.00
7.25
7.50
7.75
8.00

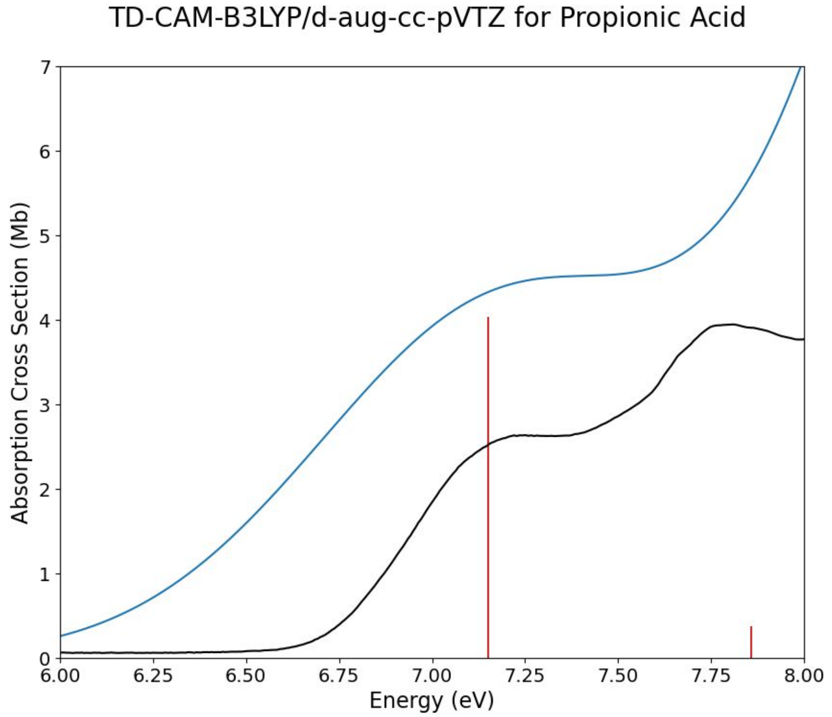
TD-CAM-B3LYP/d-aug-cc-pVTZ for Propionic Acid
Absorption Cross Section (Mb)
Energy (eV)
0
1
2
3
4
5
6
7
6.00
6.25
6.50
6.75
7.00
7.25
7.50
7.75
8.00

EOM-CCSD/d-aug-cc-pVTZ for Propionic Acid

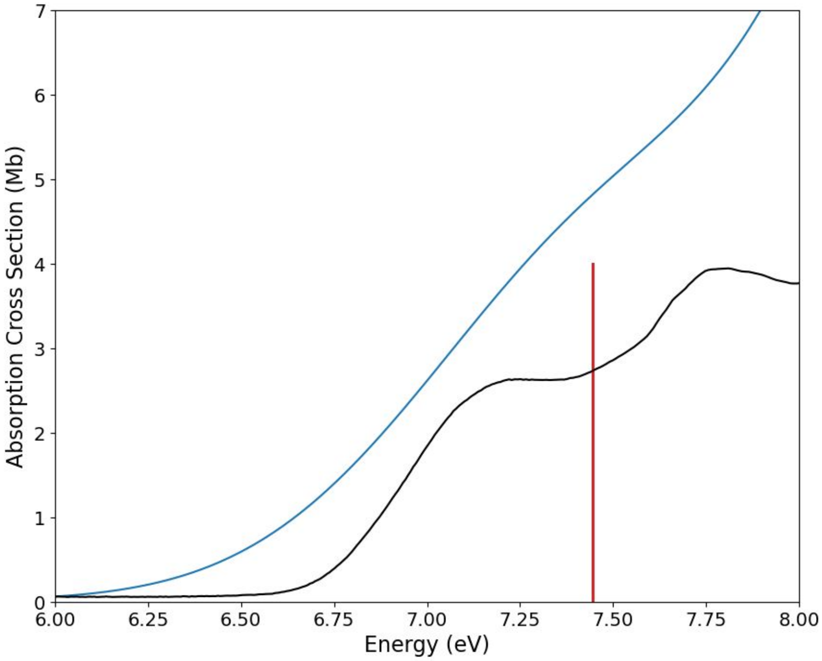

TD-HSE/d-aug-cc-pVTZ for Propionic Acid

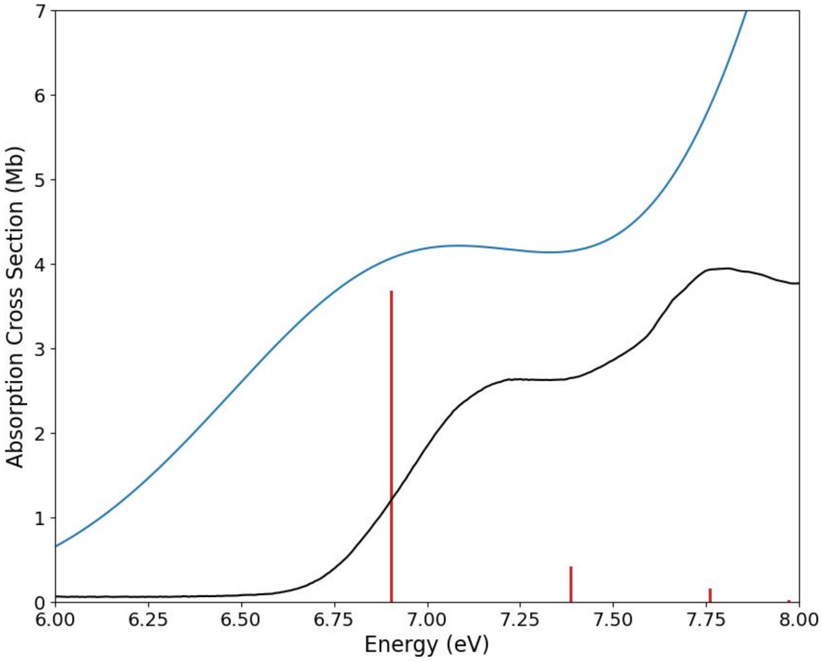

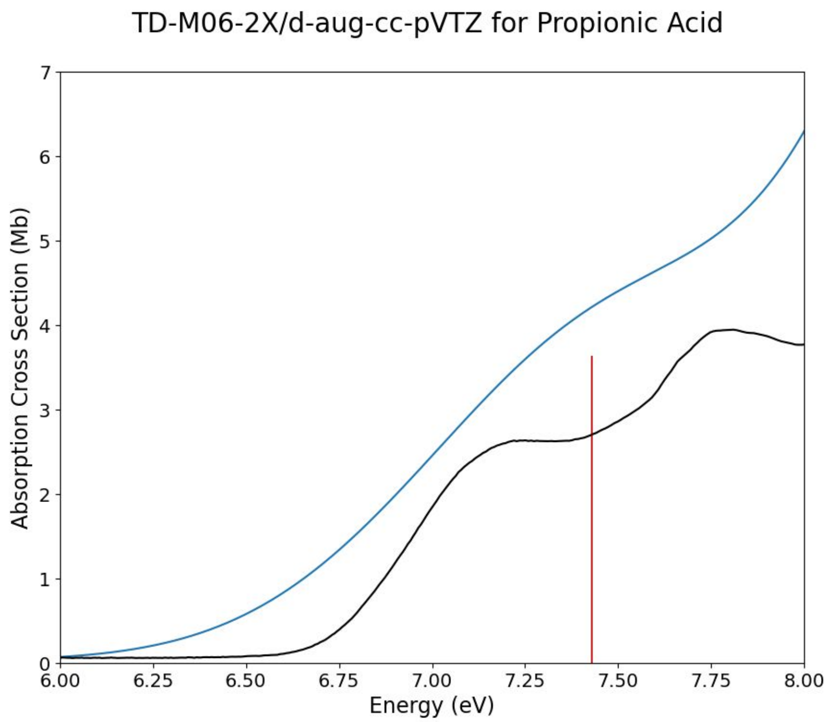

TD-M06-2X/d-aug-cc-pVTZ for Propionic Acid
Absorption Cross Section (Mb)
Energy (eV)
0
1
2
3
4
5
6
7
6.00
6.25
6.50
6.75
7.00
7.25
7.50
7.75
8.00

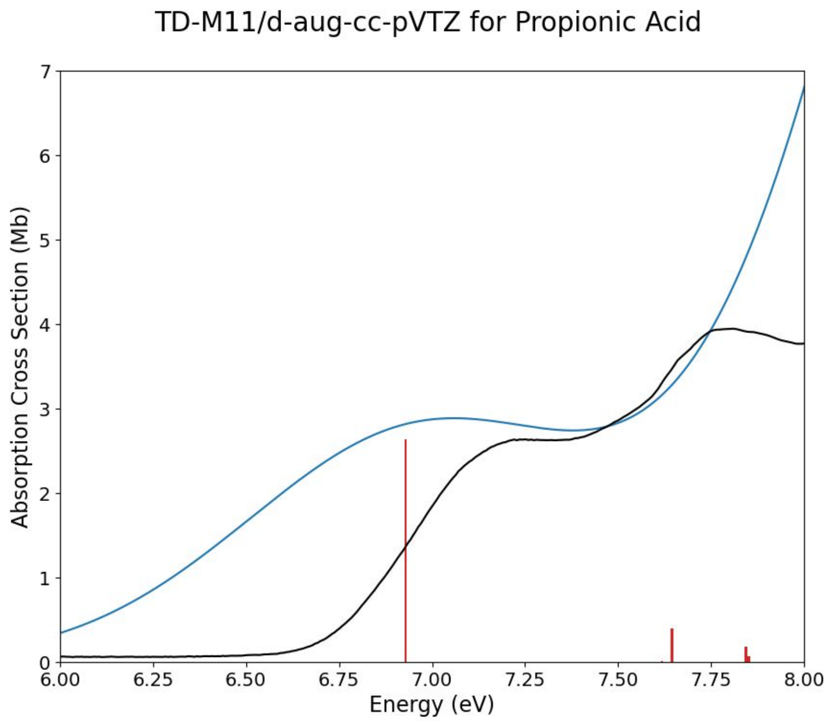

TD-M11/d-aug-cc-pVTZ for Propionic Acid
Absorption Cross Section (Mb)
Energy (eV)
0
1
2
3
4
5
6
7
6.00
6.25
6.50
6.75
7.00
7.25
7.50
7.75
8.00

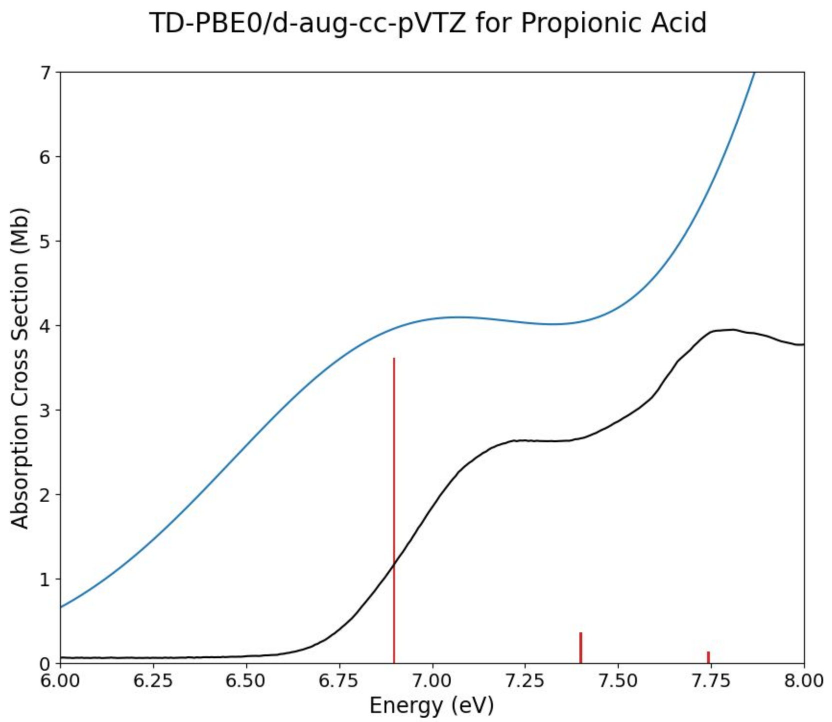
TD-PBE0/d-aug-cc-pVTZ for Propionic Acid
Absorption Cross Section (Mb)
Energy (eV)
0
1
2
3
4
5
6
7
6.00
6.25
6.50
6.75
7.00
7.25
7.50
7.75
8.00

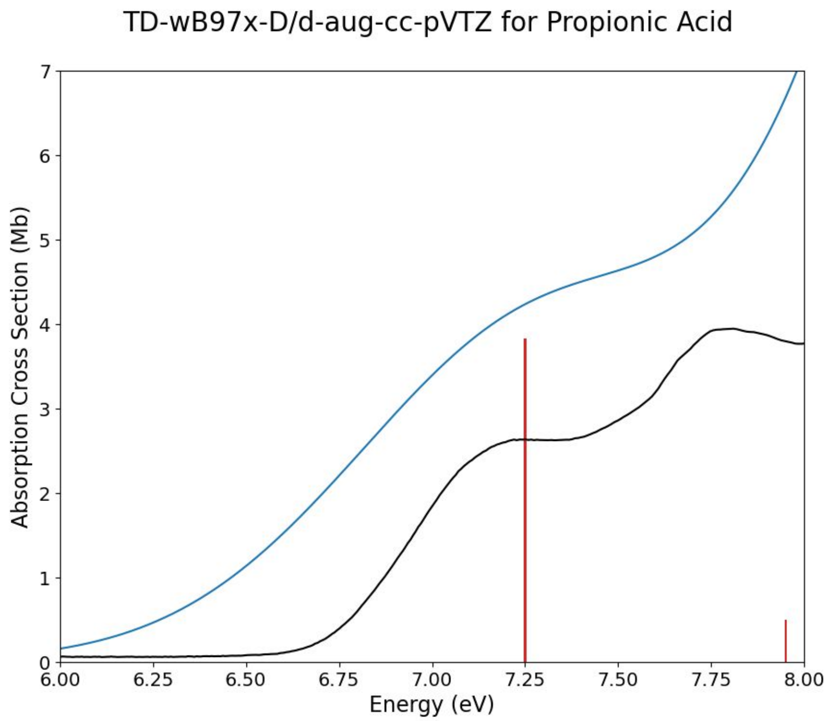
TD-wB97x-D/d-aug-cc-pVTZ for Propionic Acid
Absorption Cross Section (Mb)
Energy (eV)
0
1
2
3
4
5
6
7
6.00
6.25
6.50
6.75
7.00
7.25
7.50
7.75
8.00

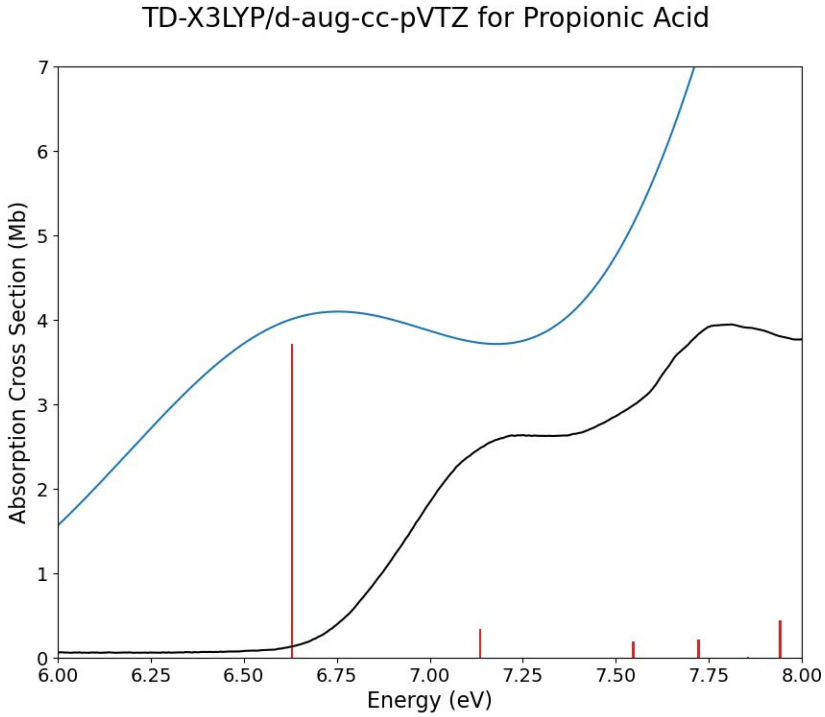
TD-X3LYP/d-aug-cc-pVTZ for Propionic Acid
Absorption Cross Section (Mb)
Energy (eV)
0
1
2
3
4
5
6
7
6.00
6.25
6.50
6.75
7.00
7.25
7.50
7.75
8.00

15 Methanol

Table S57. Optimized Geometry of Methanol in Å

	CCSD(T)/d-aug-cc-pVTZ			M06-2X/d-aug-cc-pVTZ		
C	0.0000000000	0.0130589257	-0.7311078112	-0.0263049533	0.7250922662	0.0000000000
O	0.0000000000	-0.0641588529	0.6927288213	-0.0263049533	-0.6892987223	0.0000000000
H	0.0000000000	0.8327736012	1.0382011369	0.8794326280	1.0027824367	0.0000000000
H	0.0000000000	-1.0122307615	-1.0994494606	-1.0663983795	1.0416791434	0.0000000000
H	0.8922267464	0.5211294544	-1.1112640373	0.4588265107	1.1336124168	-0.8896359326
H	-0.8922267464	0.5211294544	-1.1112640373	0.4588265107	1.1336124168	0.8896359326

Table S58. Frequencies of Methanol in cm^{-1}

CCSD(T)/d-aug-cc-pVTZ	M06-2X/d-aug-cc-pVTZ
289.460	300.308
1054.035	1069.814
1082.878	1110.755
1175.707	1178.949
1380.242	1369.007
1482.362	1486.336
1511.332	1508.475
1521.679	1519.471
3010.733	3041.406
3069.070	3094.181
3128.355	3152.235
3843.109	3899.496

Table S59. Transition Energies of Methanol in eV

CCSD(T)/d-aug-cc-pVTZ	M06-2X/d-aug-cc-pVTZ
6.883	6.730
8.074	7.754
8.504	8.165
8.524	8.192
8.857	8.405
9.128	8.781
9.311	8.921
9.428	9.077
9.469	9.084
9.568	9.151
9.604	9.187
9.678	9.189
9.792	9.234
9.845	9.282
	9.304

	9.623<br>9.646<br>9.672<br>9.882

Table S60. Quantitative Metrics for the Bandwidth (γ), cosine similarity (S), relative integral change (RIC), mean signed error (MSE), and mean average error (MAE) for the band shape of methanol compared to experiment.

Method	γ	S	RIC	MSE	MAE
B3LYP	0.07	0.260	1.983	1.766	5.097
BH&HLYP	0.1	0.797	0.993	-2.478	2.478
BMK	0.08	0.935	0.678	-1.735	1.735
CAM-B3LYP	0.09	0.460	1.997	3.208	5.248
CC	0.1	0.868	0.998	-2.629	2.629
HSE	0.058	0.596	1.630	3.156	3.997
M06-2X	0.09	0.917	0.634	0.118	1.589
M11	0.06	0.280	2.439	3.629	6.227
PBE0	0.08	0.532	1.663	3.029	4.255
wB97x-D	0.09	0.713	1.591	3.110	3.922
X3LYP	0.07	0.273	2.016	2.282	5.364

Figure S15. Spectra of Methanol

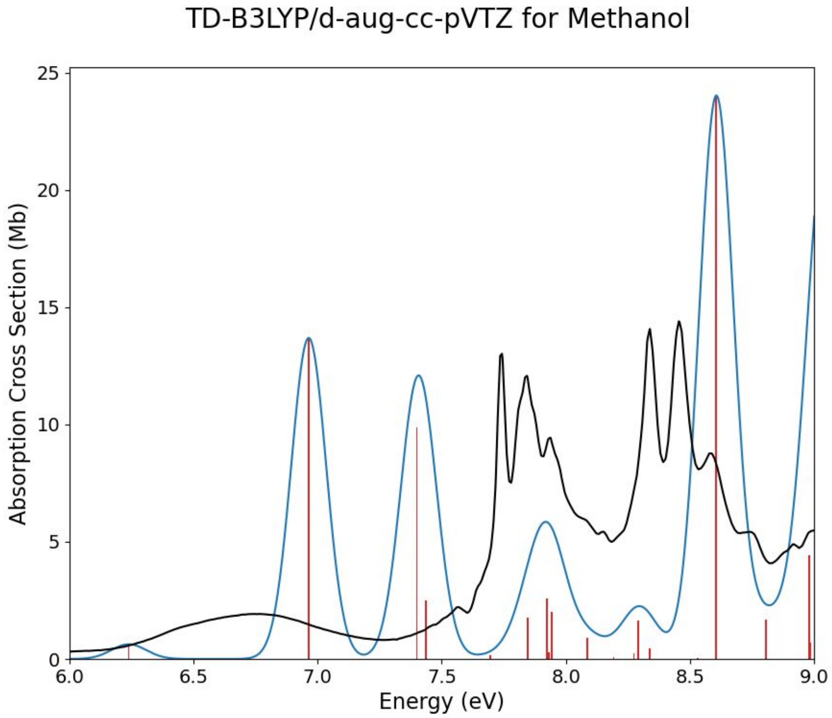

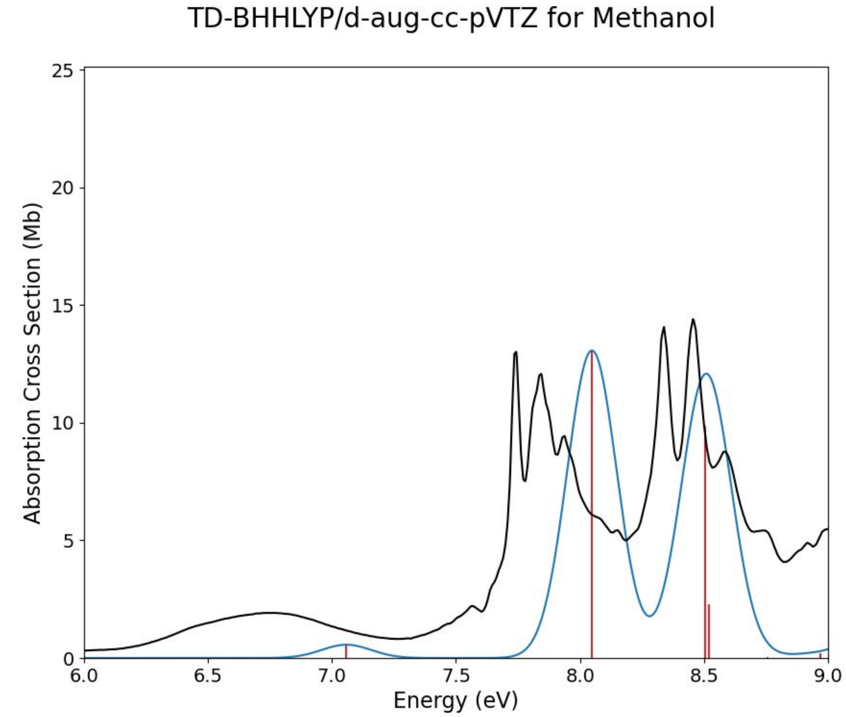
TD-BHHLYP/d-aug-cc-pVTZ for Methanol
Absorption Cross Section (Mb)
Energy (eV)
0
5
10
15
20
25
6.0
6.5
7.0
7.5
8.0
8.5
9.0

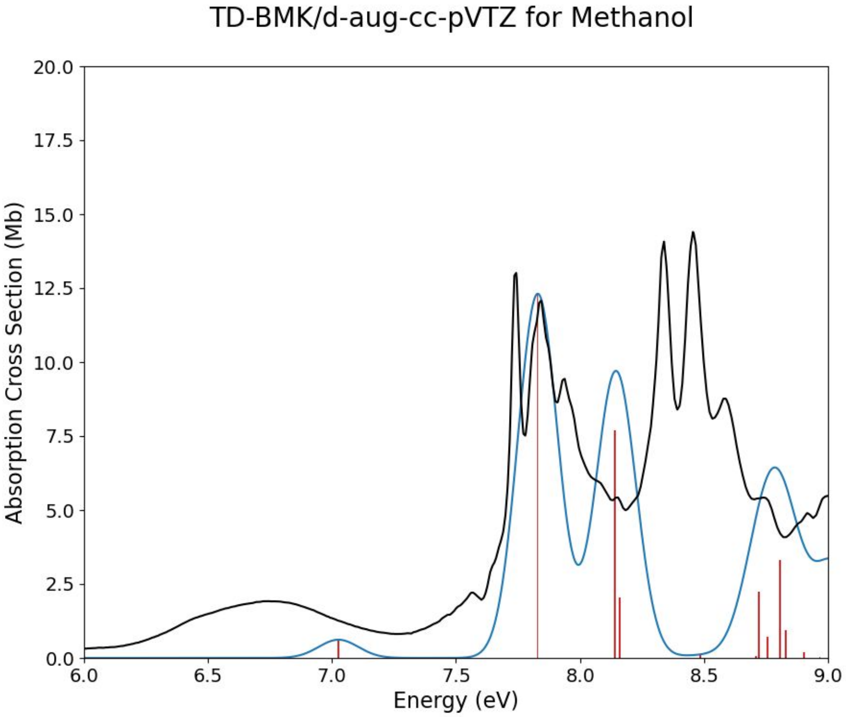
TD-BMK/d-aug-cc-pVTZ for Methanol
Absorption Cross Section (Mb)
Energy (eV)
0.0
2.5
5.0
7.5
10.0
12.5
15.0
17.5
20.0
6.0
6.5
7.0
7.5
8.0
8.5
9.0

TD-CAM-B3LYP/d-aug-cc-pVTZ for Methanol

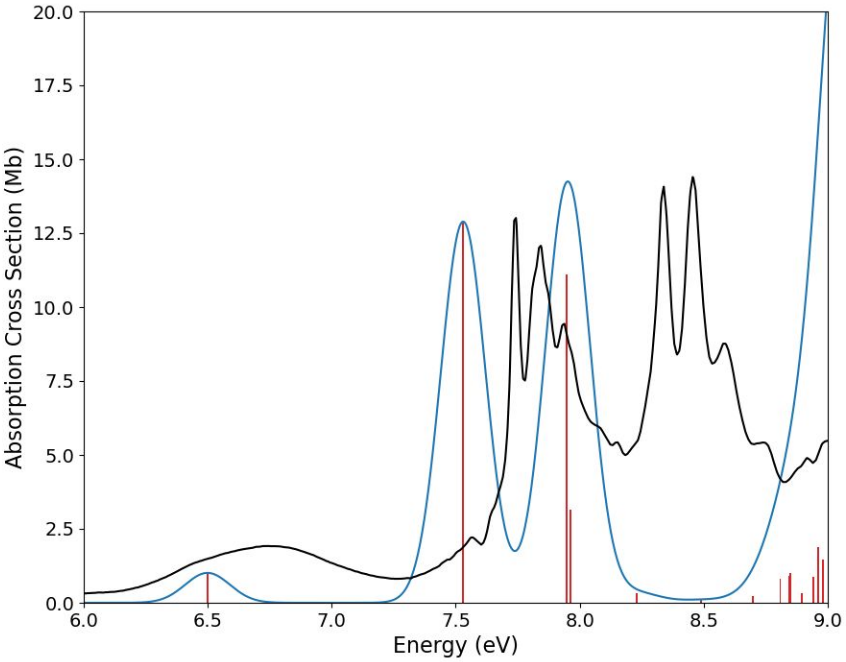

EOM-CCSD/d-aug-cc-pVTZ for Methanol

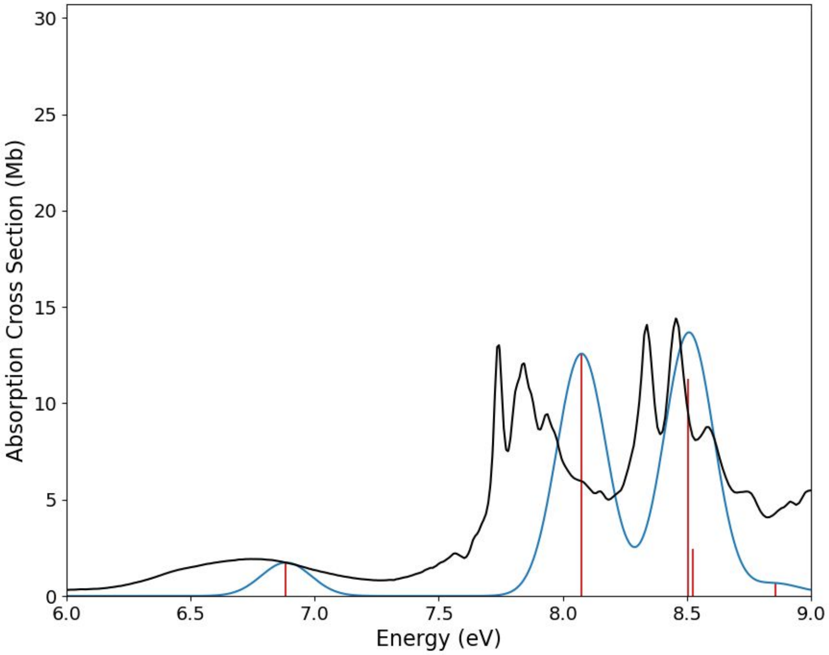

TD-HSE/d-aug-cc-pVTZ for Methanol

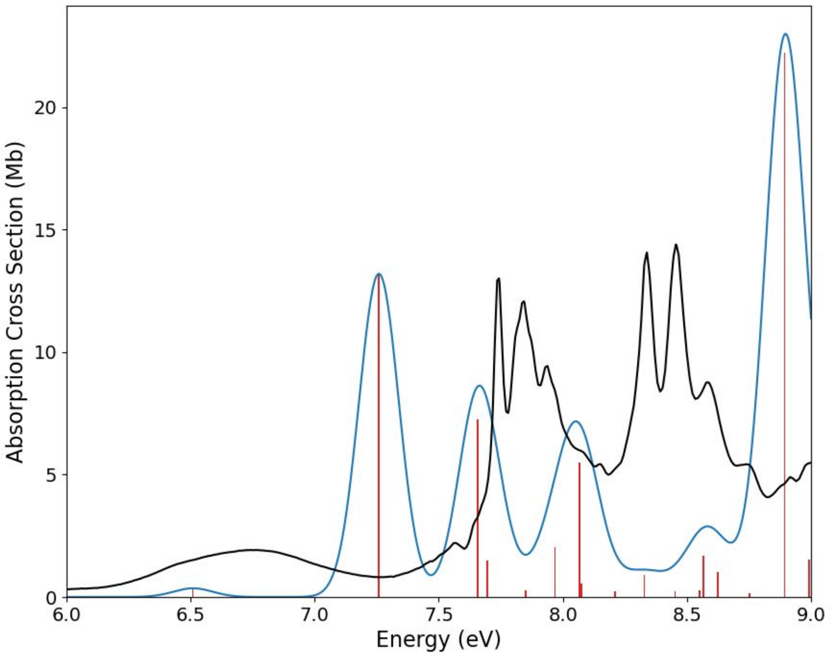

TD-M06-2X/d-aug-cc-pVTZ for Methanol

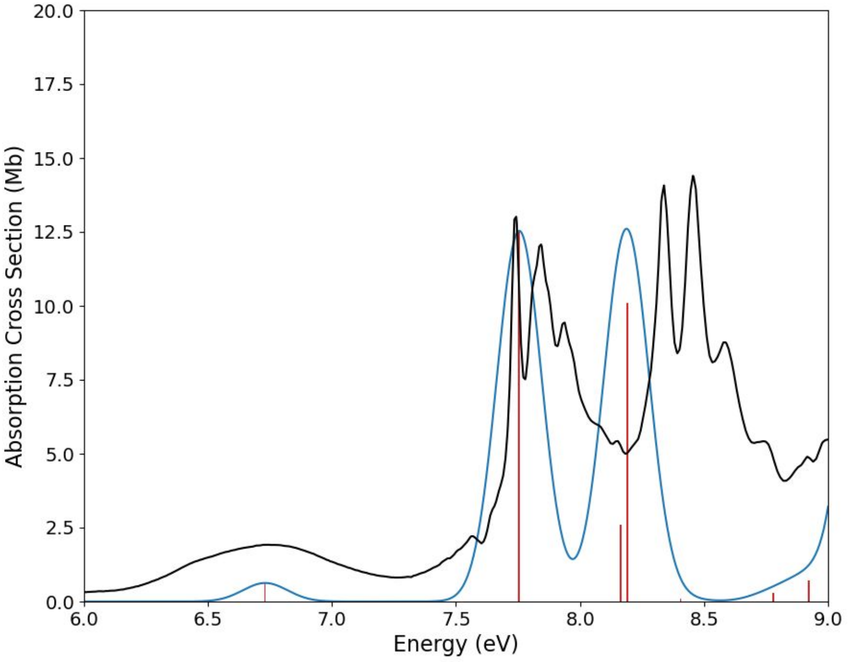

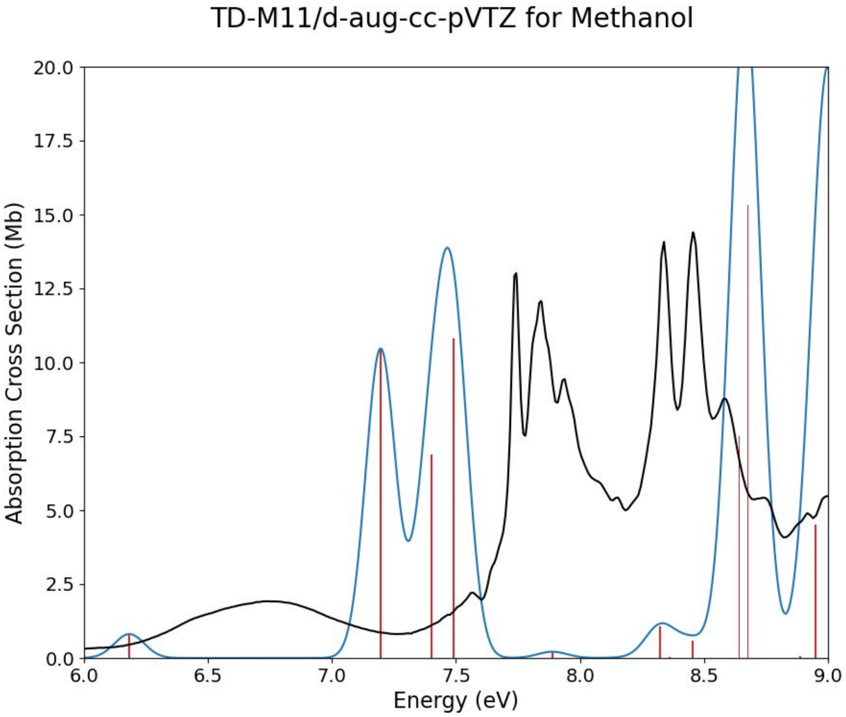

TD-M11/d-aug-cc-pVTZ for Methanol
Absorption Cross Section (Mb)
Energy (eV)

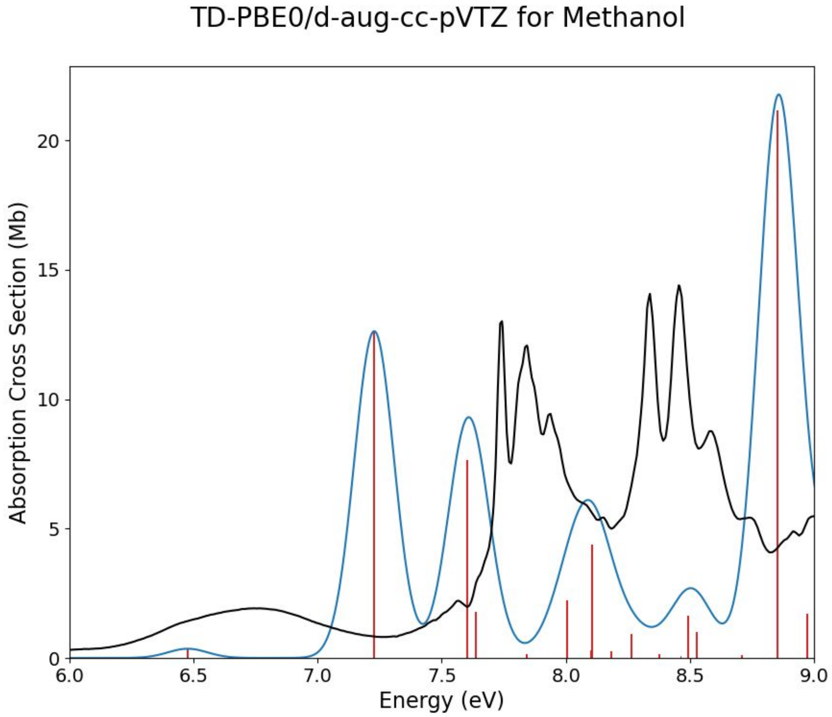

TD-PBE0/d-aug-cc-pVTZ for Methanol
Absorption Cross Section (Mb)
Energy (eV)

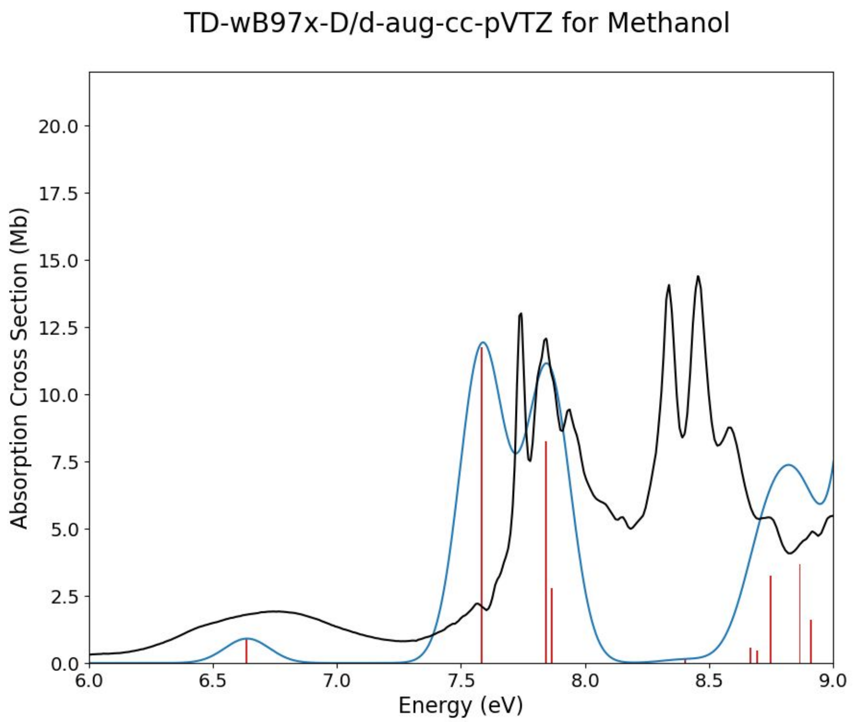
TD-wB97x-D/d-aug-cc-pVTZ for Methanol
Absorption Cross Section (Mb)
0.0
2.5
5.0
7.5
10.0
12.5
15.0
17.5
20.0
6.0
6.5
7.0
7.5
8.0
8.5
9.0
Energy (eV)

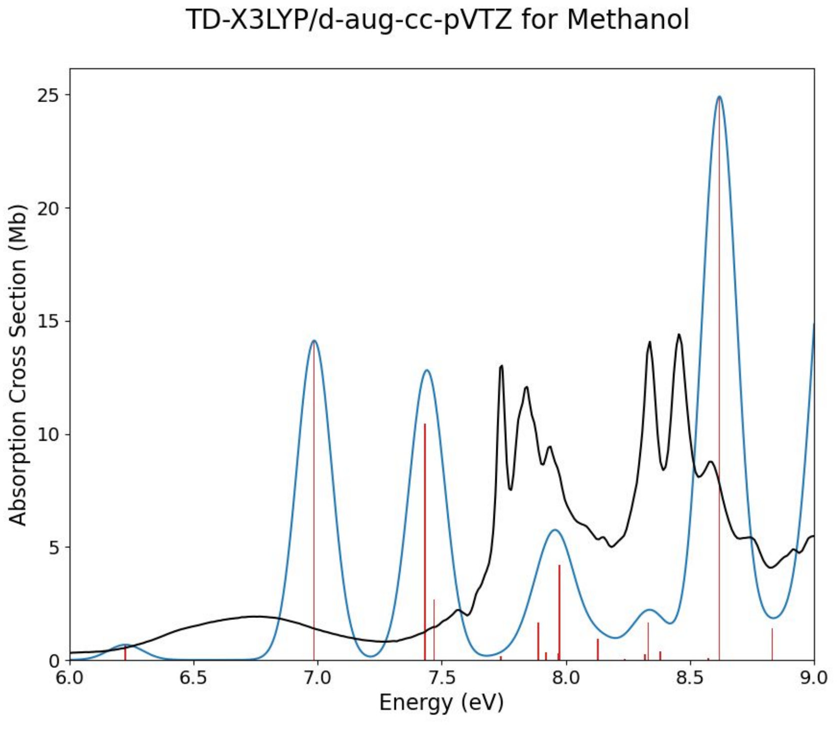
TD-X3LYP/d-aug-cc-pVTZ for Methanol
Absorption Cross Section (Mb)
0
5
10
15
20
25
6.0
6.5
7.0
7.5
8.0
8.5
9.0
Energy (eV)

16 Ethanol

Table S61. Optimized Geometry of Ethanol in Å

	CCSD(T)/d-aug-cc-pVTZ			M06-2X/d-aug-cc-pVTZ		
O	0.0000000000	0.3250967747	-1.1144136123	-1.1401657224	-0.1817148177	0.0000000000
C	0.0000000000	-0.5797999702	-0.0060580607	1.2173555220	-0.4042094467	0.0000000000
C	0.0000000000	0.2520418705	1.2615647525	0.0633614437	0.5715191797	0.0000000000
H	0.0000000000	-0.1922714204	-1.9252703411	-1.8894353413	0.4166842780	0.0000000000
H	0.8880432793	-1.2227416672	-0.0386012368	2.1681749029	0.1273725955	0.0000000000
H	-0.8880432793	-1.2227416672	-0.0386012368	1.1713934460	-1.0398192350	0.8830215960
H	0.0000000000	-0.3955805995	2.1417328875	1.1713934460	-1.0398192350	-0.8830215960
H	-0.8860161808	0.8893233900	1.2945673729	0.1122267280	1.2136996461	0.8847507810
H	0.8860161808	0.8893233900	1.2945673729	0.1122267280	1.2136996461	-0.8847507810

Table S62. Frequencies of Ethanol in cm^{-1}

CCSD(T)/d-aug-cc-pVTZ	M06-2X/d-aug-cc-pVTZ
233.837	237.761
280.997	286.871
413.630	420.935
818.009	821.095
904.432	918.377
1048.246	1051.084
1108.947	1133.812
1183.334	1184.270
1276.922	1269.592
1303.614	1307.526
1405.837	1401.809
1457.289	1456.536
1492.964	1487.155
1510.157	1504.795
1535.136	1534.885
3000.936	3030.071
3034.617	3060.205
3038.103	3073.427
3116.803	3149.298
3122.109	3151.120
3835.467	3894.994

Table S63. Transition Energies of Ethanol in eV

CCSD(T)/d-aug-cc-pVTZ	M06-2X/d-aug-cc-pVTZ
6.877	6.719
8.033	7.680
8.263	7.958

8.338	7.990
8.795	8.383
9.033	8.618
9.090	8.725
9.229	8.793
9.262	8.842
9.272	8.888
9.317	8.928
9.499	9.062
9.567	9.092
	9.097
	9.138
	9.320
	9.336
	9.397
	9.449
	9.452
	9.505
	9.560

Table S64. Quantitative Metrics for the Bandwidth (γ), cosine similarity (S), relative integral change (RIC), mean signed error (MSE), and mean average error (MAE) for the band shape of ethanol compared to experiment.

Method	γ	S	RIC	MSE	MAE
B3LYP	0.37	0.886	0.488	-2.141	2.923
BH&HLYP	0.15	0.915	0.764	-4.612	4.612
BMK	0.1	0.947	0.348	-0.842	2.137
CAM-B3LYP	0.15	0.852	0.983	4.560	6.010
CC	0.13	0.868	0.746	-4.502	4.502
HSE	0.15	0.791	0.557	-0.090	3.406
M06-2X	0.13	0.978	0.365	1.325	2.169
M11	0.1	0.315	0.934	-5.722	5.806
PBE0	0.13	0.851	0.546	-1.682	3.292
wB97x-D	0.13	0.717	1.041	2.948	6.366
X3LYP	0.13	0.943	0.541	-3.259	3.259

Figure S16. Spectra of Ethanol

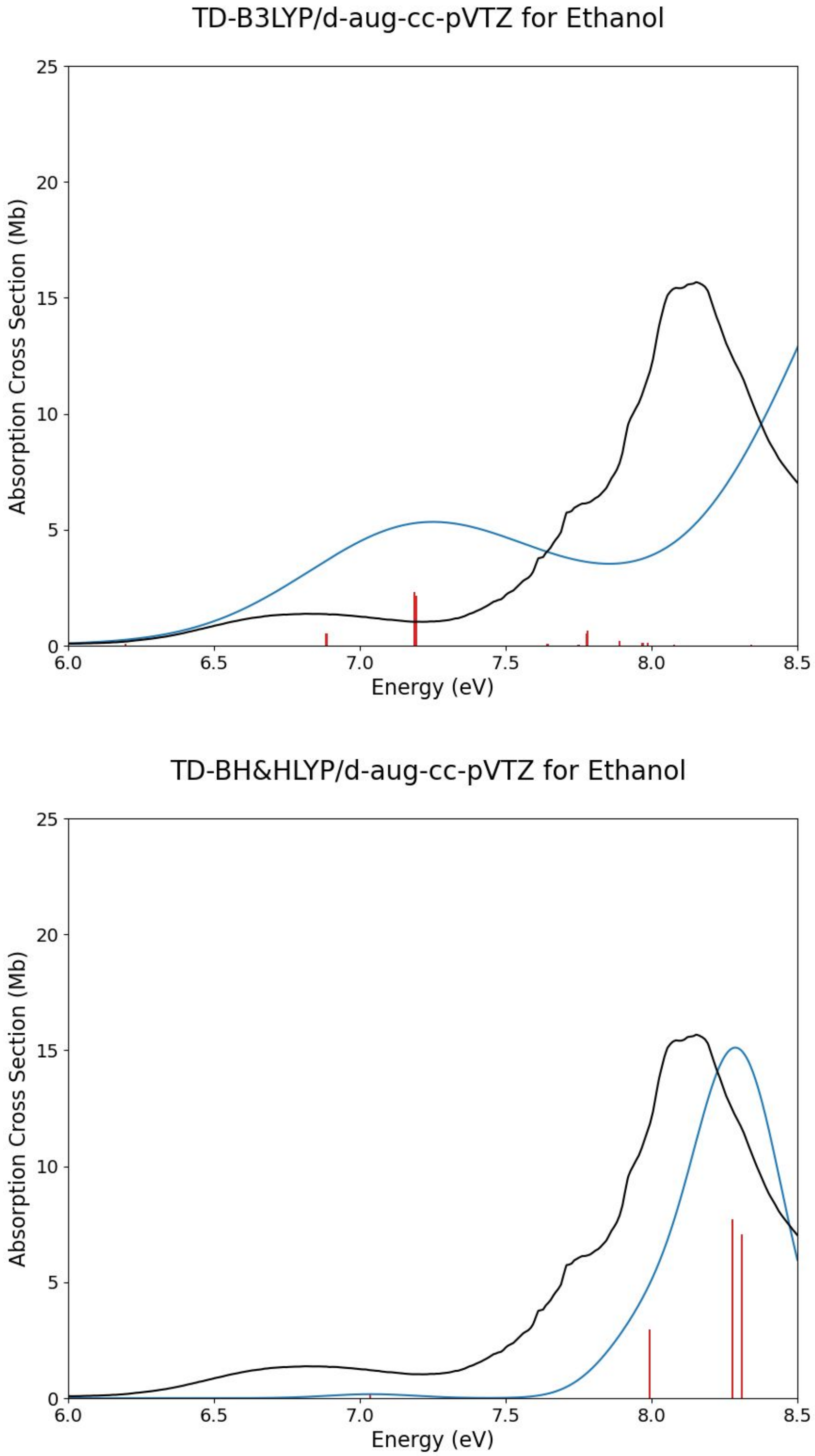

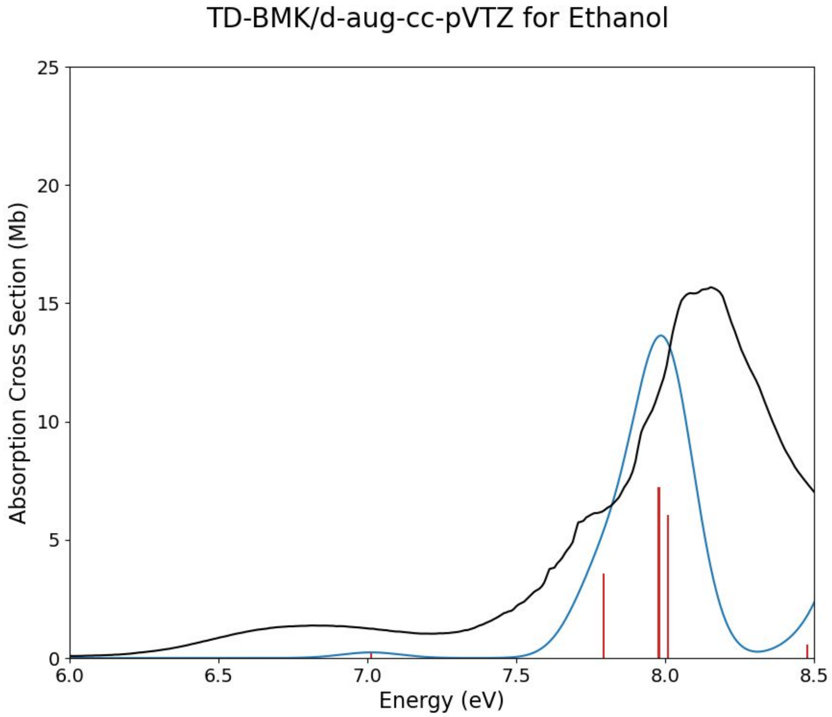
TD-BMK/d-aug-cc-pVTZ for Ethanol
Absorption Cross Section (Mb)
Energy (eV)
0
5
10
15
20
25
6.0
6.5
7.0
7.5
8.0
8.5

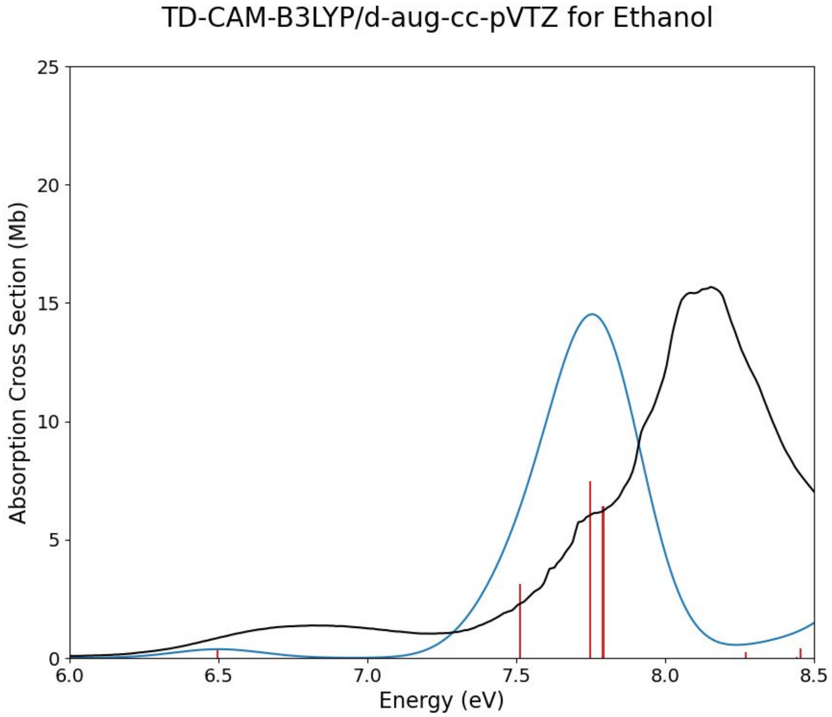
TD-CAM-B3LYP/d-aug-cc-pVTZ for Ethanol
Absorption Cross Section (Mb)
Energy (eV)
0
5
10
15
20
25
6.0
6.5
7.0
7.5
8.0
8.5

EOM-CCSD/d-aug-cc-pVTZ for Ethanol

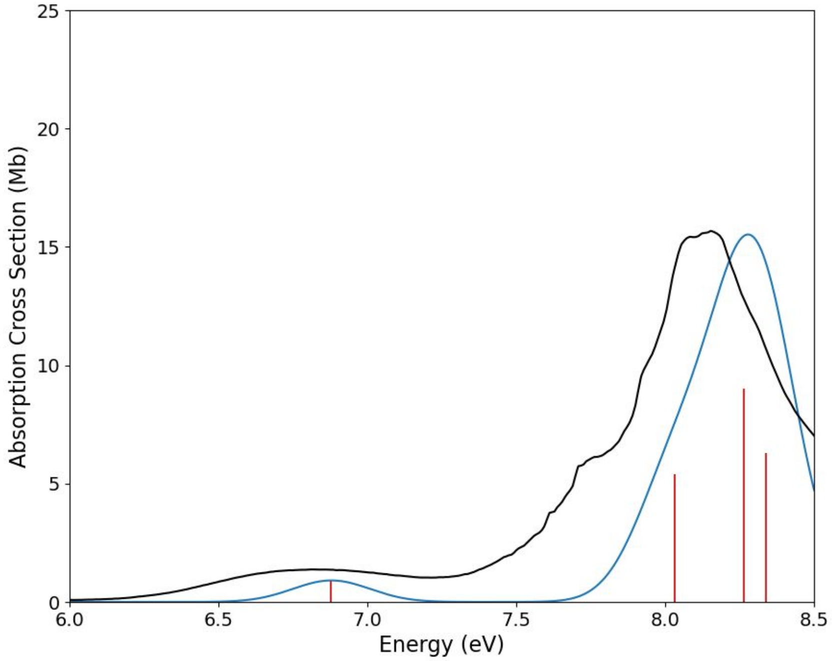

TD-HSE/d-aug-cc-pVTZ for Ethanol

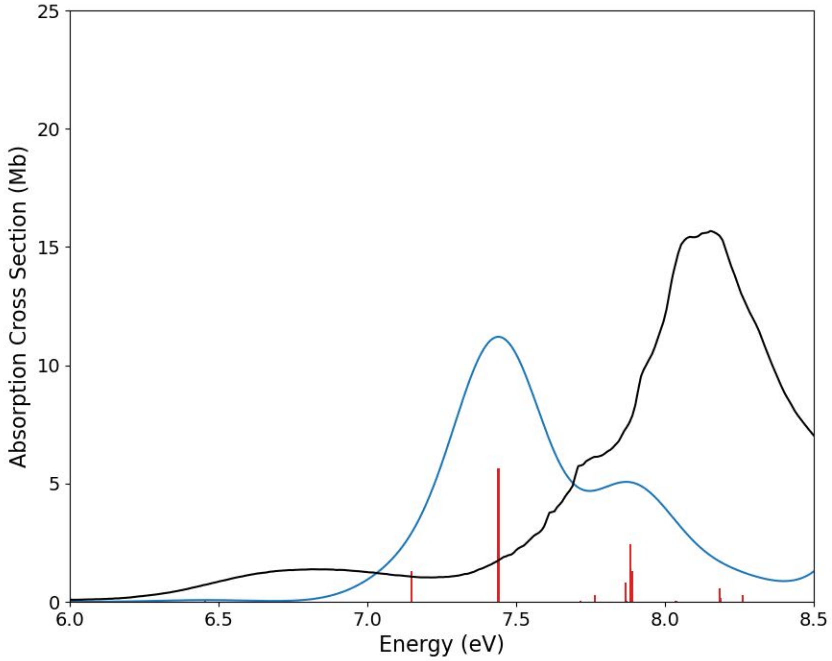

TD-M06-2X/d-aug-cc-pVTZ for Ethanol

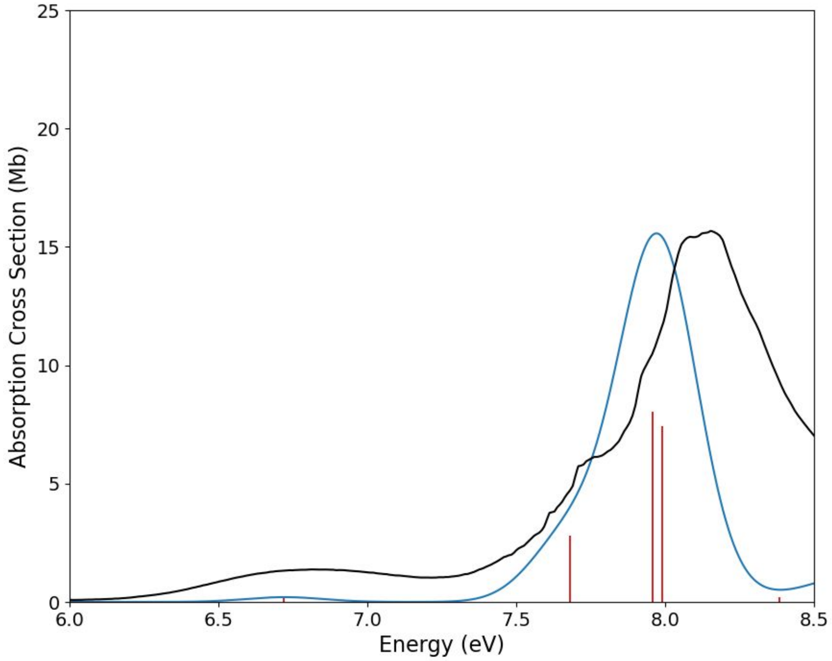

TD-M11/d-aug-cc-pVTZ for Ethanol

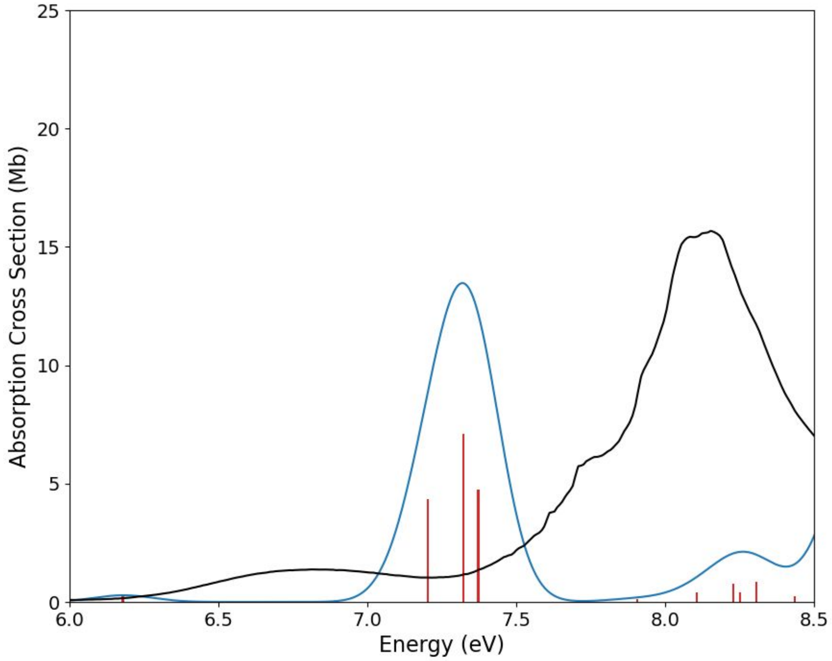

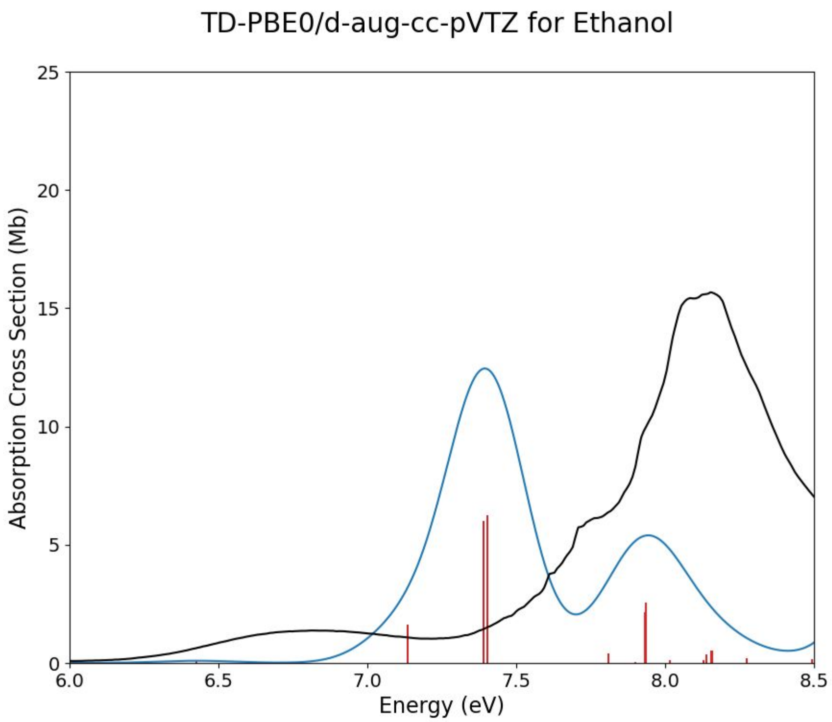
TD-PBE0/d-aug-cc-pVTZ for Ethanol
Absorption Cross Section (Mb)
Energy (eV)
0
5
10
15
20
25
6.0
6.5
7.0
7.5
8.0
8.5

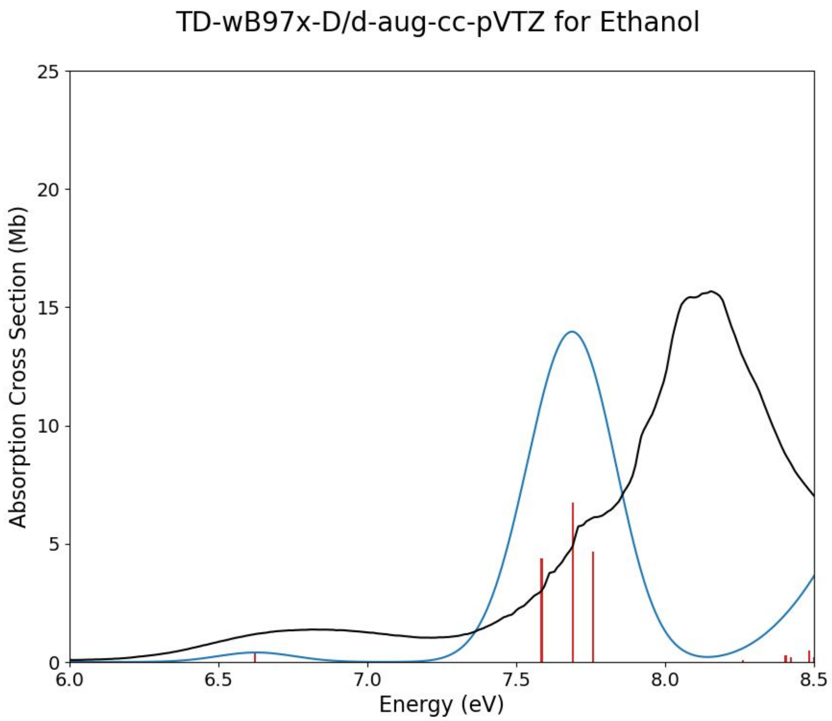
TD-wB97x-D/d-aug-cc-pVTZ for Ethanol
Absorption Cross Section (Mb)
Energy (eV)
0
5
10
15
20
25
6.0
6.5
7.0
7.5
8.0
8.5

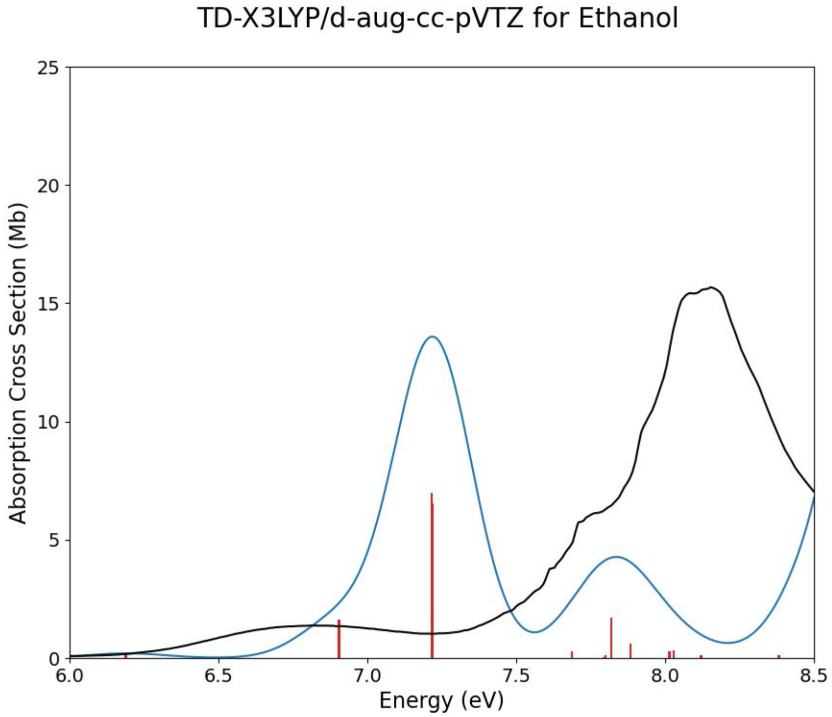
TD-X3LYP/d-aug-cc-pVTZ for Ethanol
Absorption Cross Section (Mb)
Energy (eV)
0
5
10
15
20
25
6.0
6.5
7.0
7.5
8.0
8.5

17 1-Propanol

Table S65. Optimized Geometry of 1-Propanol in Å

	CCSD(T)/d-aug-cc-pVTZ			M06-2X/d-aug-cc-pVTZ		
O	0.0000000000	0.1857832400	-1.7835767293	1.4117456476	-1.0920119234	0.0000000000
C	0.0000000000	-0.5106251232	-0.5351634358	-1.5100562787	1.2427580542	0.0000000000
C	0.0000000000	0.5204762107	0.5788631277	-0.0611661865	0.7743422271	0.0000000000
C	0.0000000000	-0.1412980986	1.9574413547	0.0390550439	-0.7355811270	0.0000000000
H	0.0000000000	-0.4653087561	-2.4915181468	-1.5732266691	2.3295020151	0.0000000000
H	0.8887231237	-1.1503612402	-0.4503029322	-2.0401640698	0.8783979252	0.8810052124
H	-0.8887231237	-1.1503612402	-0.4503029322	-2.0401640698	0.8783979252	-0.8810052124
H	-0.8800666136	1.1597937796	0.4611596820	0.4650012841	1.1555080714	0.8764916482
H	0.8800666136	1.1597937796	0.4611596820	0.4650012841	1.1555080714	-0.8764916482
H	0.0000000000	0.6070757394	2.7526696824	-0.4654491814	-1.1388527821	0.8853152364
H	0.8841602256	-0.7716321415	2.0910676369	-0.4654491814	-1.1388527821	-0.8853152364
H	-0.8841602256	-0.7716321415	2.0910676369	1.4922776977	-2.0474157922	0.0000000000

Table S66. Frequencies of 1-Propanol in cm^{-1}

CCSD(T)/d-aug-cc-pVTZ	M06-2X/d-aug-cc-pVTZ
123.595	124.952
229.031	226.317
256.092	262.022
269.061	275.145
456.418	463.811
762.174	760.550
893.852	898.310
900.902	900.564
1045.443	1060.260
1077.648	1086.033
1098.550	1119.071
1187.058	1188.554
1263.712	1258.216
1269.942	1271.029
1324.529	1323.798
1351.401	1347.297
1416.828	1413.369
1457.489	1458.089
1501.083	1496.670
1509.479	1504.749
1515.383	1511.444
1530.661	1531.300
2987.084	3015.630
3021.317	3046.286
3025.080	3061.737

3040.941	3073.692
3077.166	3107.469
3100.599	3131.301
3107.871	3141.313
3835.731	3895.999

Table S67. Transition Energies of 1-Propanol in eV

CCSD(T)/d-aug-cc-pVTZ	M06-2X/d-aug-cc-pVTZ
6.868	6.728
8.078	7.692
8.224	7.920
8.306	7.964
8.670	8.231
8.956	8.538
9.111	8.605
9.127	8.667
9.168	8.707
9.194	8.719
9.229	8.754
9.277	8.871
9.287	8.889
9.449	8.957
9.511	9.036
9.536	9.039
9.559	9.084
	9.084
	9.131
	9.282
	9.296
	9.306
	9.337
	9.355
	9.371
	9.387
	9.420
	9.471
	9.476
	9.508
	9.584

Table S68. Quantitative Metrics for the Bandwidth (γ), cosine similarity (S), relative integral change (RIC), mean signed error (MSE), and mean average error (MAE) for the band shape of 1-propanol compared to experiment.

Method	γ	S	RIC	MSE	MAE
B3LYP	0.3	0.845	1.041	2.323	2.323
BH&HLYP	0.35	0.991	0.455	-0.990	0.990
BMK	0.27	0.993	0.147	-0.285	0.315
CAM-B3LYP	0.35	0.965	1.394	3.050	3.050
CC	0.4	0.997	0.283	-0.579	0.589
HSE	0.33	0.890	1.577	3.462	3.462
M06-2X	0.33	0.998	0.498	1.015	1.015
M11	0.28	0.746	1.180	2.088	2.574
PBE0	0.3	0.858	1.475	3.211	3.211
wB97x-D	0.33	0.964	1.231	2.695	2.695
X3LYP	0.3	0.836	1.147	2.551	2.551

Figure S17. Spectra of 1-Propanol

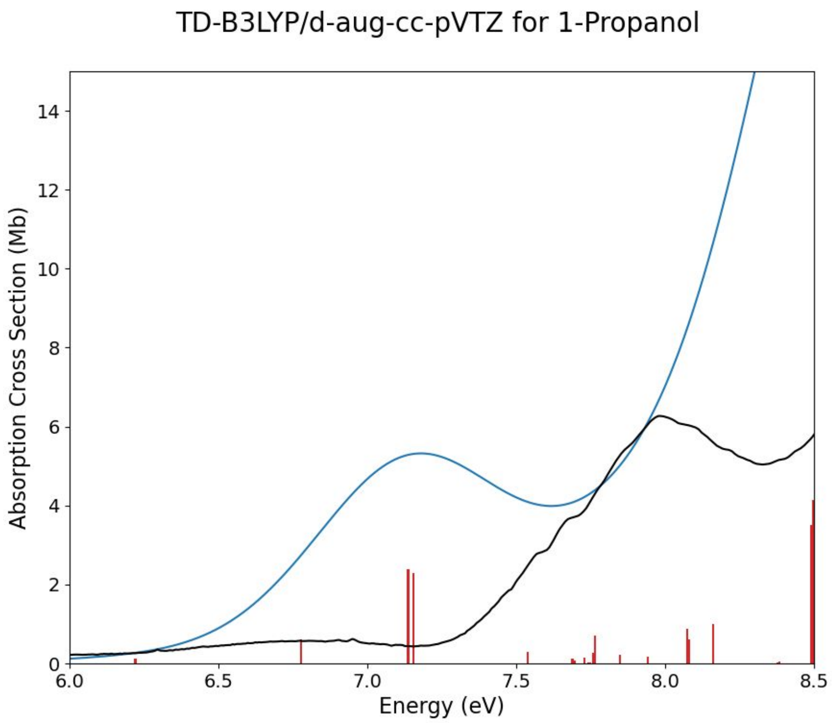

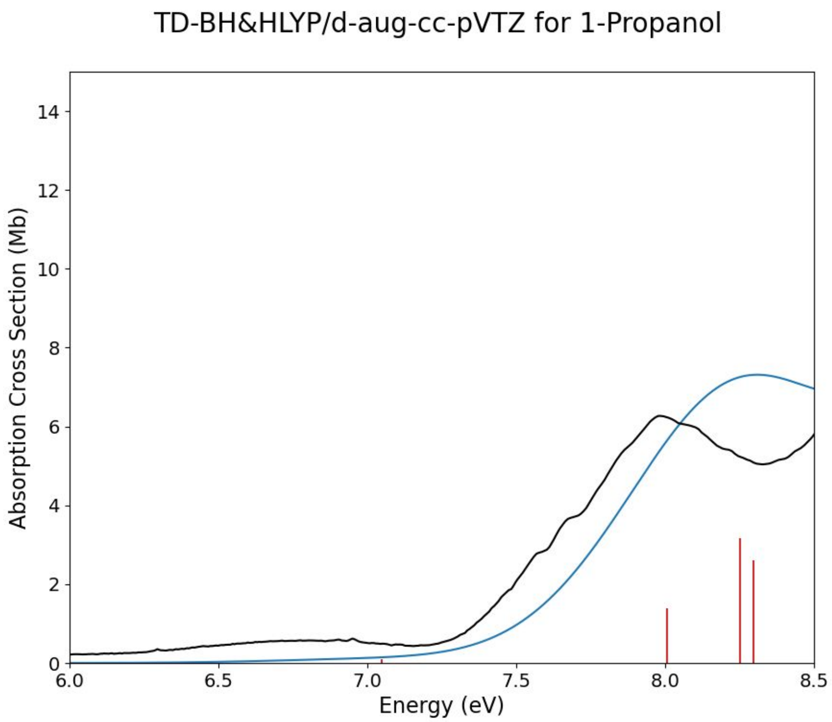

TD-BH&HLYP/d-aug-cc-pVTZ for 1-Propanol
Absorption Cross Section (Mb)
Energy (eV)
0
2
4
6
8
10
12
14
6.0
6.5
7.0
7.5
8.0
8.5

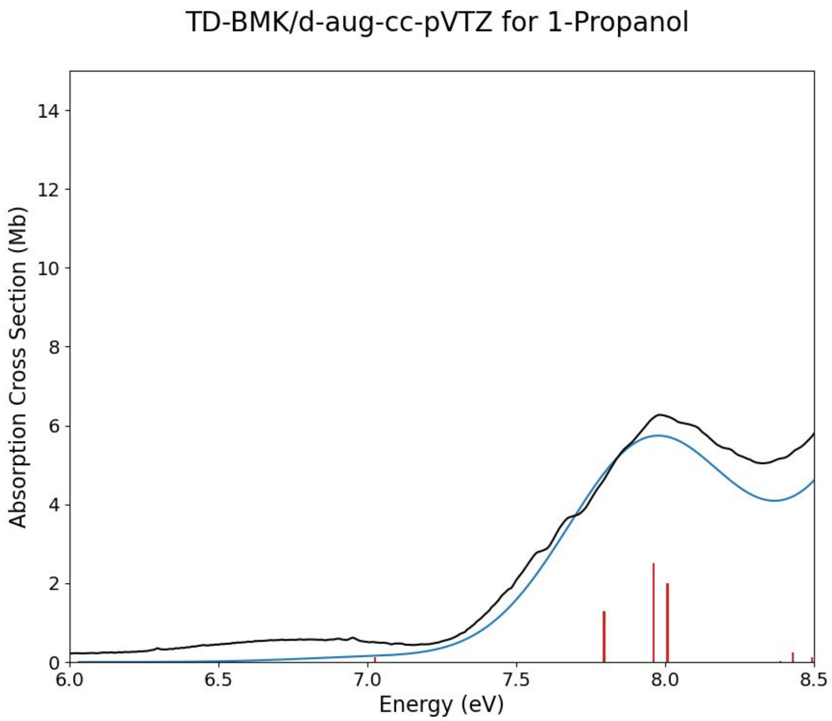

TD-BMK/d-aug-cc-pVTZ for 1-Propanol
Absorption Cross Section (Mb)
Energy (eV)
0
2
4
6
8
10
12
14
6.0
6.5
7.0
7.5
8.0
8.5

TD-CAM-B3LYP/d-aug-cc-pVTZ for 1-Propanol

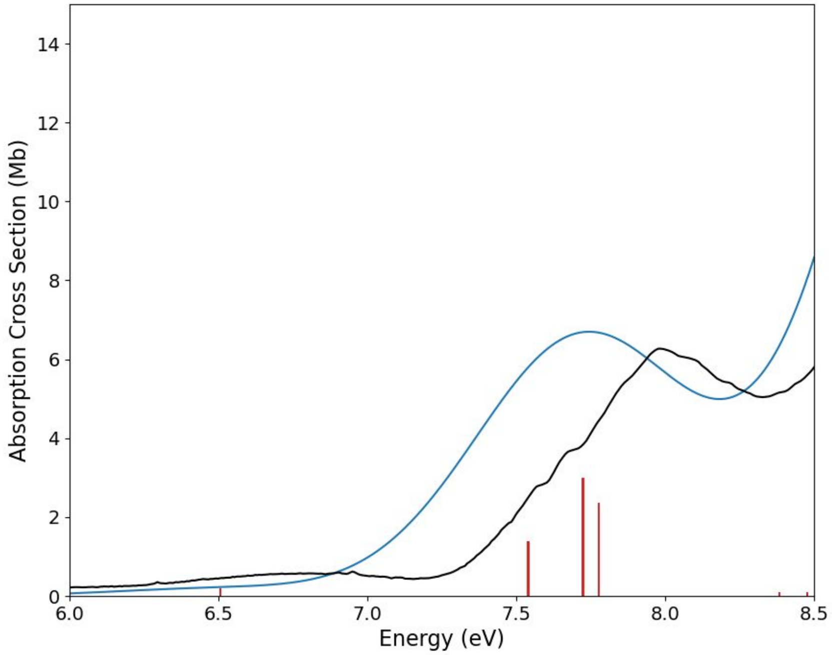

EOM-CCSD/d-aug-cc-pVTZ for Water

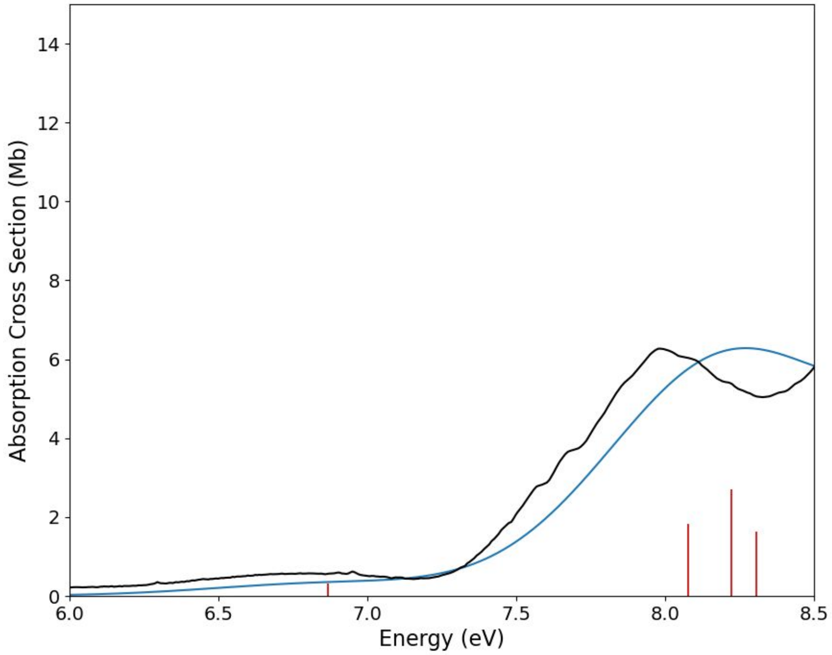

TD-HSE/d-aug-cc-pVTZ for 1-Propanol

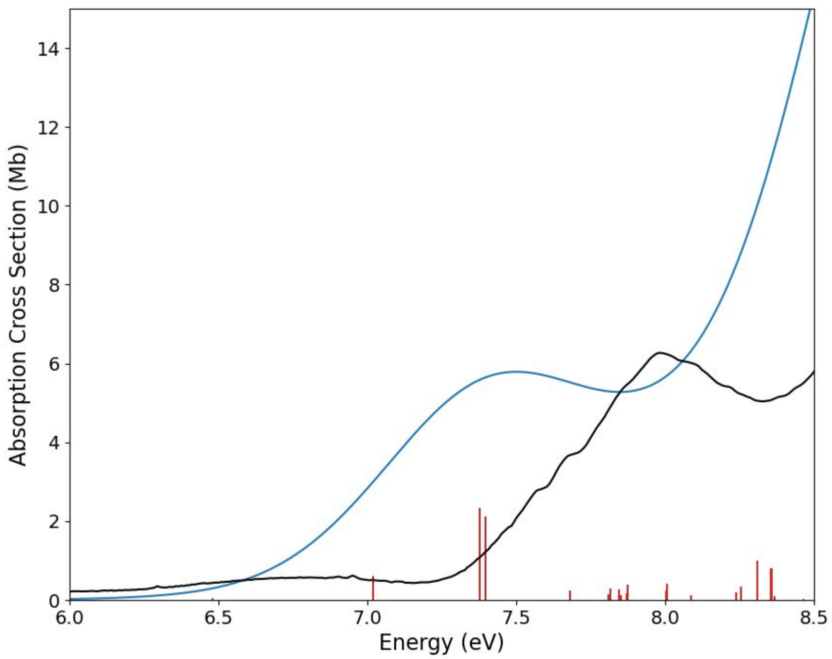

TD-M06-2X/d-aug-cc-pVTZ for 1-Propanol

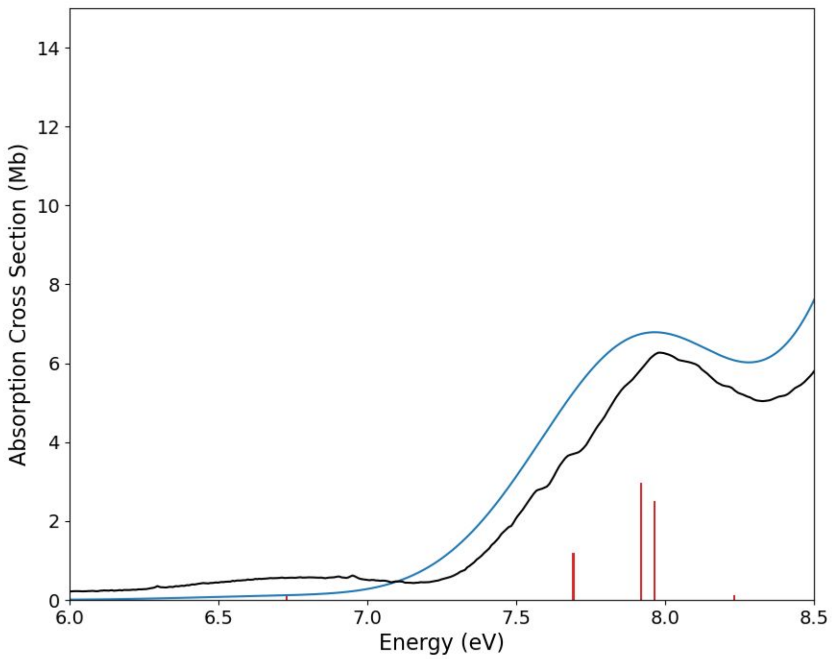

TD-M11/d-aug-cc-pVTZ for 1-Propanol

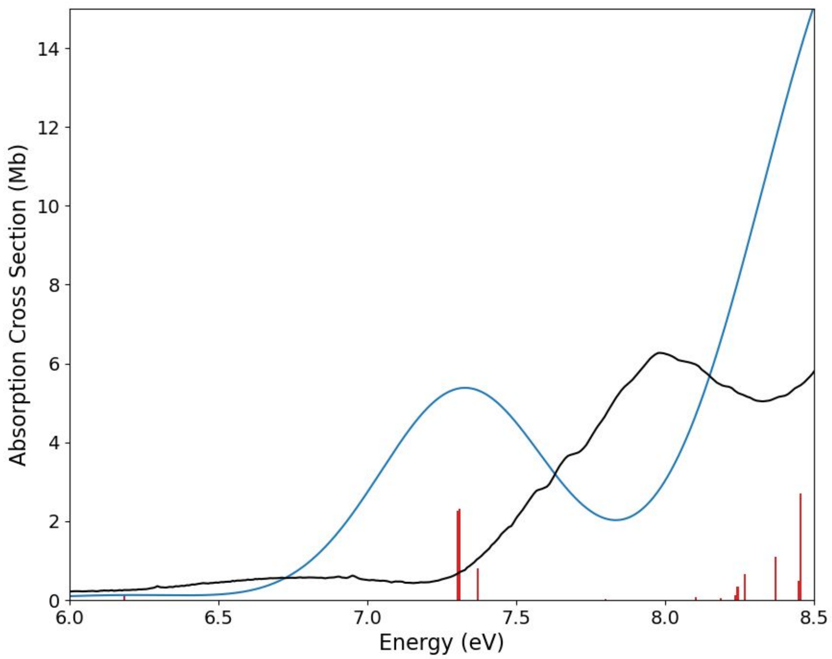

TD-PBE0/d-aug-cc-pVTZ for 1-Propanol

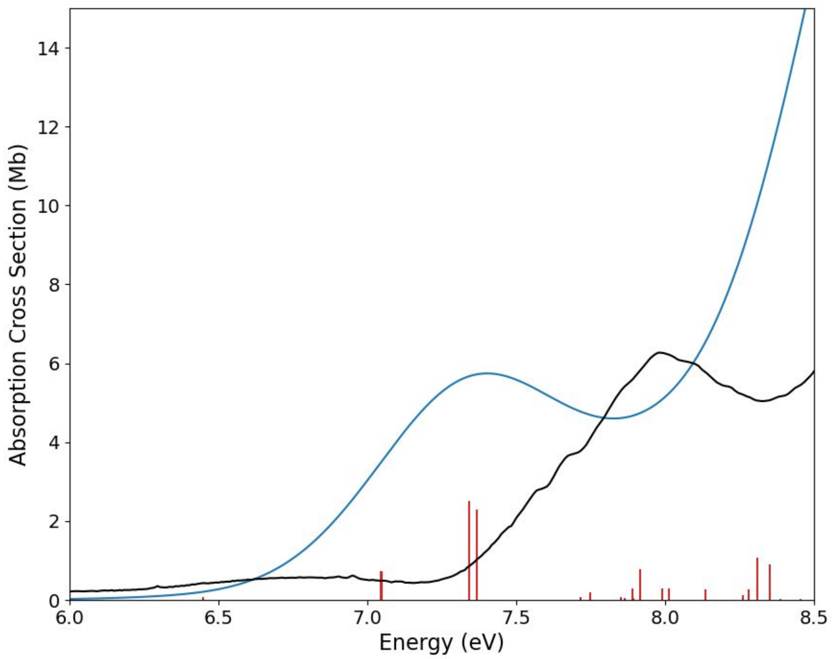

TD-wB97x-D/d-aug-cc-pVTZ for 1-Propanol

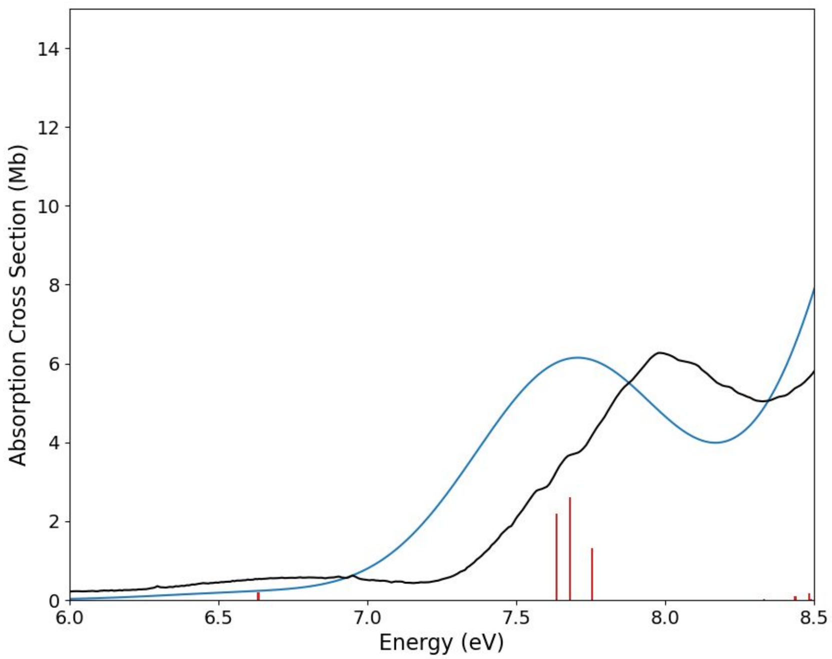

TD-X3LYP/d-aug-cc-pVTZ for 1-Propanol

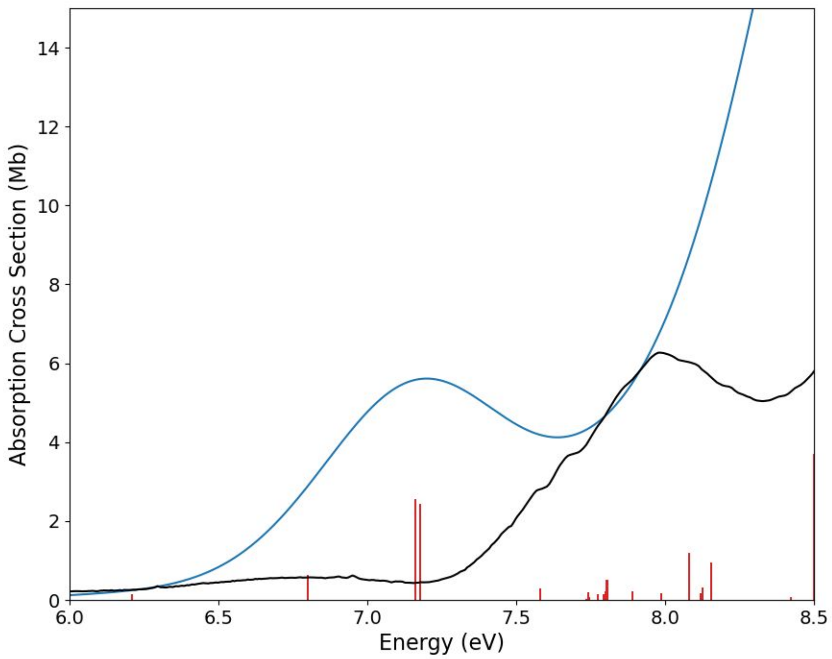

18 2-Propanol

Table S69. Optimized Geometry of 2-Propanol in Å

	CCSD(T)/d-aug-cc-pVTZ			M06-2X/d-aug-cc-pVTZ		
C	-0.3715533211	0.0189195851	0.0072836693	-0.0048675459	-0.0134772633	0.3711677742
C	0.1138281061	0.6406881259	1.3116179284	-1.2184204154	-0.7929893366	-0.1066565126
C	0.1018222177	0.7959686516	-1.2089949989	1.2933405659	-0.6417541607	-0.0904330890
O	0.1320999710	-1.3149383722	-0.1467774843	-0.0158122193	1.3128538859	-0.1456987705
H	-1.4701102418	-0.0083042267	0.0087856228	-0.0118821312	0.0264479943	1.4666023202
H	-0.2379263552	0.0661012916	2.1742825770	-2.1439172528	-0.3144749334	0.2185555671
H	1.2068074472	0.6615546749	1.3372237171	-1.2199530825	-0.8428068506	-1.1959794338
H	-0.2612902370	1.6634709609	1.4059387121	-1.2077574747	-1.8079845451	0.2908479614
H	-0.2612956398	0.3267384473	-2.1252884814	2.1418434937	-0.0499921917	0.2481781708
H	-0.2730786620	1.8219401745	-1.1684807340	1.3904055309	-1.6535924743	0.3014922128
H	1.1942563835	0.8205443580	-1.2371600333	1.3141789306	-0.6832693741	-1.1798592404
H	-0.1364338459	-1.8247550668	0.6248620468	-0.8460709803	1.7334449839	0.0897886816

Table S70. Frequencies of 2-Propanol in cm^{-1}

CCSD(T)/d-aug-cc-pVTZ	M06-2X/d-aug-cc-pVTZ
215.422	215.670
262.896	268.163
284.498	291.435
359.077	365.721
409.689	422.290
475.559	486.948
828.859	842.671
927.733	932.451
953.082	954.384
977.576	991.140
1097.689	1097.361
1160.971	1174.108
1195.331	1201.997
1283.583	1279.498
1377.218	1380.287
1392.587	1391.721
1414.780	1413.569
1435.097	1433.261
1489.731	1485.059
1494.652	1490.560
1503.563	1500.546
1515.917	1512.460
2990.119	3018.647
3020.240	3055.958
3033.501	3068.922

3094.499	3125.860
3106.288	3138.008
3113.522	3144.829
3120.744	3152.780
3817.934	3877.866

Table S71. Transition Energies of 2-Propanol in eV

CCSD(T)/d-aug-cc-pVTZ	M06-2X/d-aug-cc-pVTZ
6.916	6.806
8.003	7.746
8.185	7.898
8.309	8.011
8.594	8.199
8.782	8.454
8.926	8.619
8.951	8.645
	8.671
	8.742
	8.807
	8.839
	8.990
	9.003

Table S72. Quantitative Metrics for the Bandwidth (γ), cosine similarity (S), relative integral change (RIC), mean signed error (MSE), and mean average error (MAE) for the band shape of 2-propanol compared to experiment.

Method	γ	S	RIC	MSE	MAE
B3LYP	0.20	0.891	1.212	4.617	4.617
BH&HLYP	0.24	0.937	0.683	-3.623	3.623
BMK	0.16	0.975	0.447	-1.576	1.576
CAM-B3LYP	0.18	0.949	1.009	4.564	4.564
CC	0.20	0.967	0.736	-2.767	2.767
HSE	0.20	0.792	1.097	5.843	5.959
M06-2X	0.20	0.998	0.197	0.520	1.025
M11	0.12	0.579	1.248	3.175	6.091
PBE0	0.20	0.762	1.123	5.394	5.564
wB97x-D	0.17	0.830	0.696	2.631	3.850
X3LYP	0.20	0.874	1.258	3.991	3.991

Figure S18. Spectra of 2-Propanol

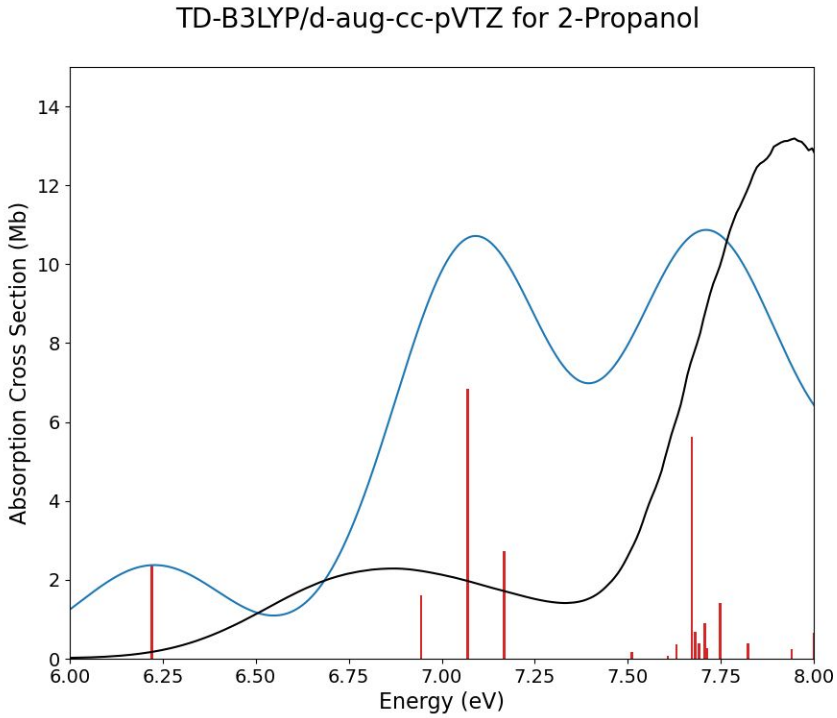

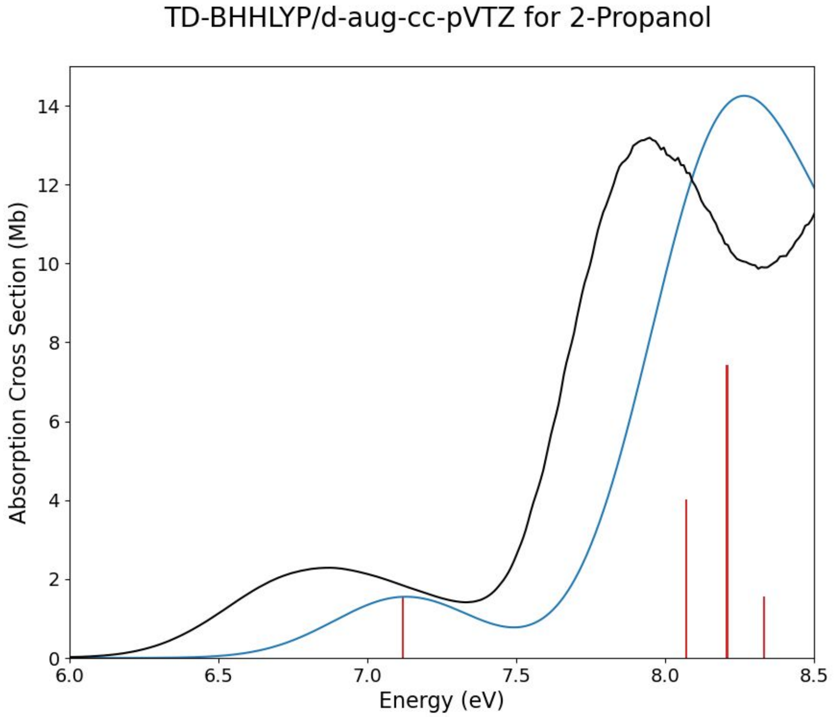

TD-BMK/d-aug-cc-pVTZ for 2-Propanol

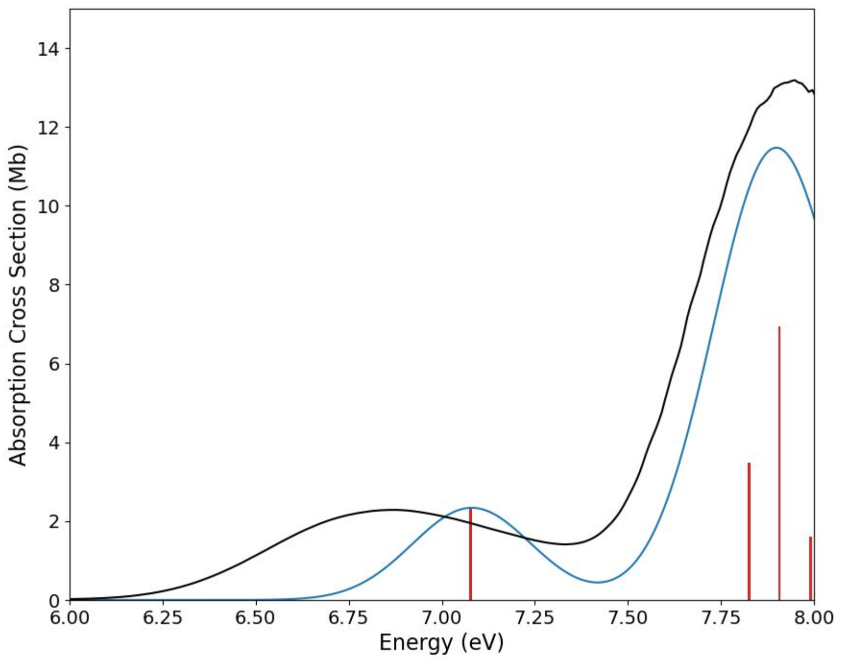

TD-CAM-B3LYP/d-aug-cc-pVTZ for 2-Propanol

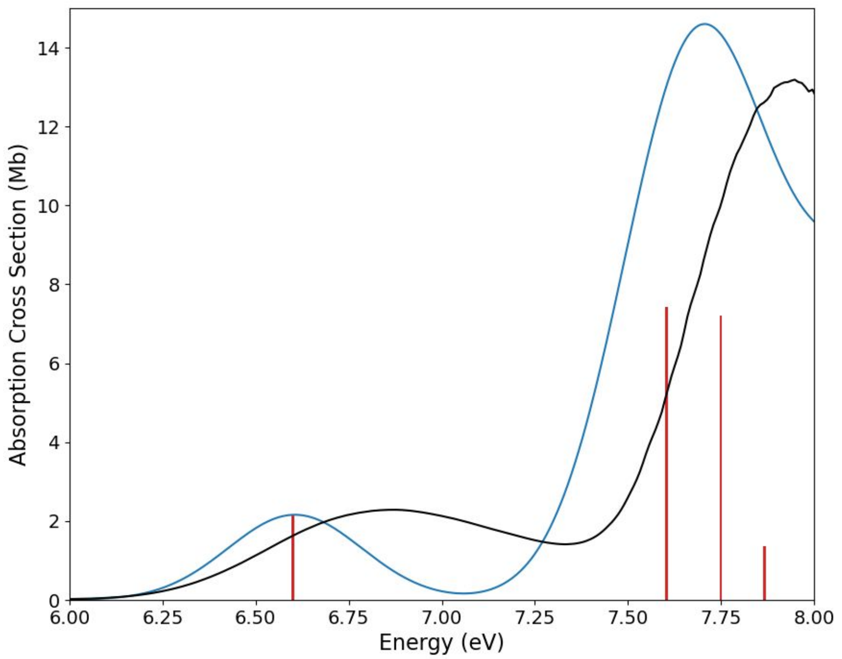

EOM-CCSD/d-aug-cc-pVTZ for 2-Propanol

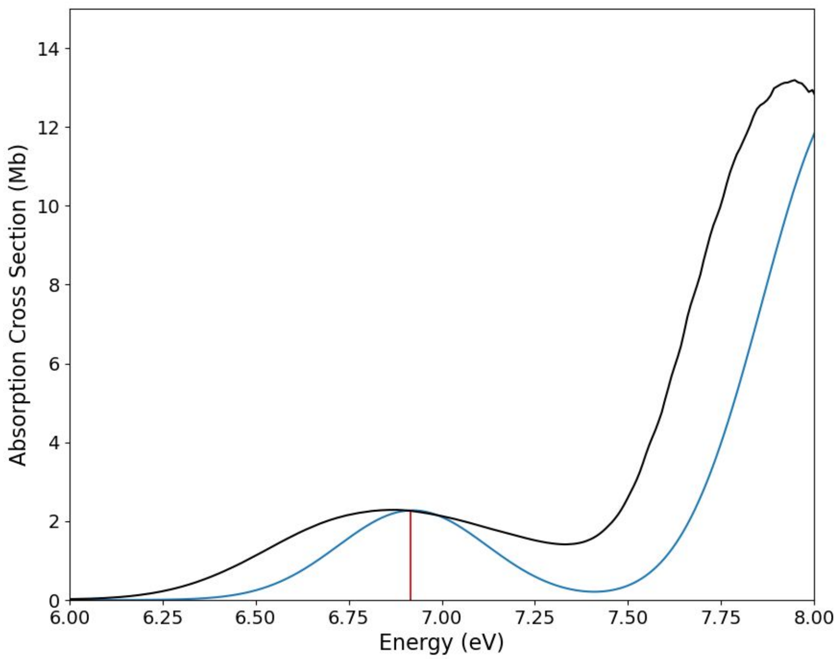

TD-HSE/d-aug-cc-pVTZ for 2-Propanol

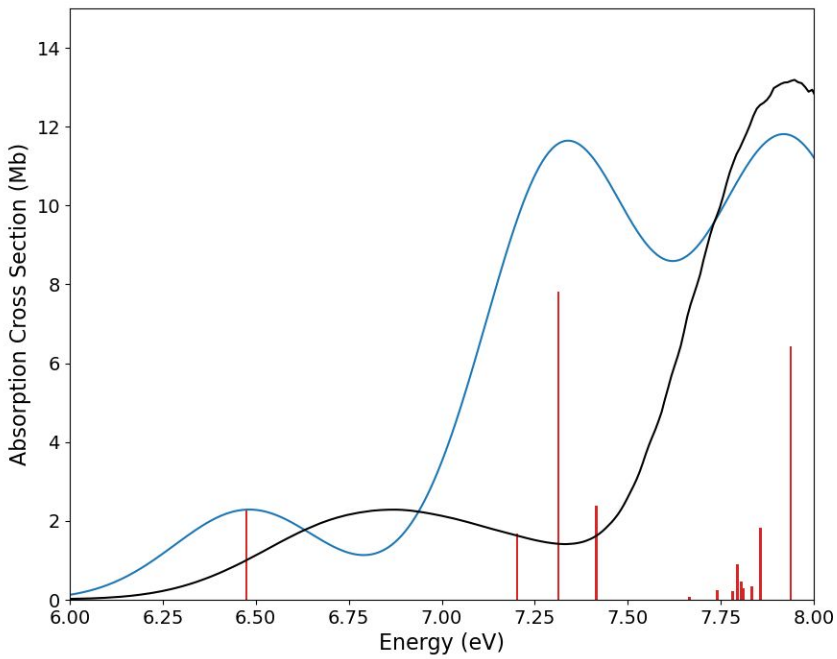

TD-M06-2X/d-aug-cc-pVTZ for 2-Propanol

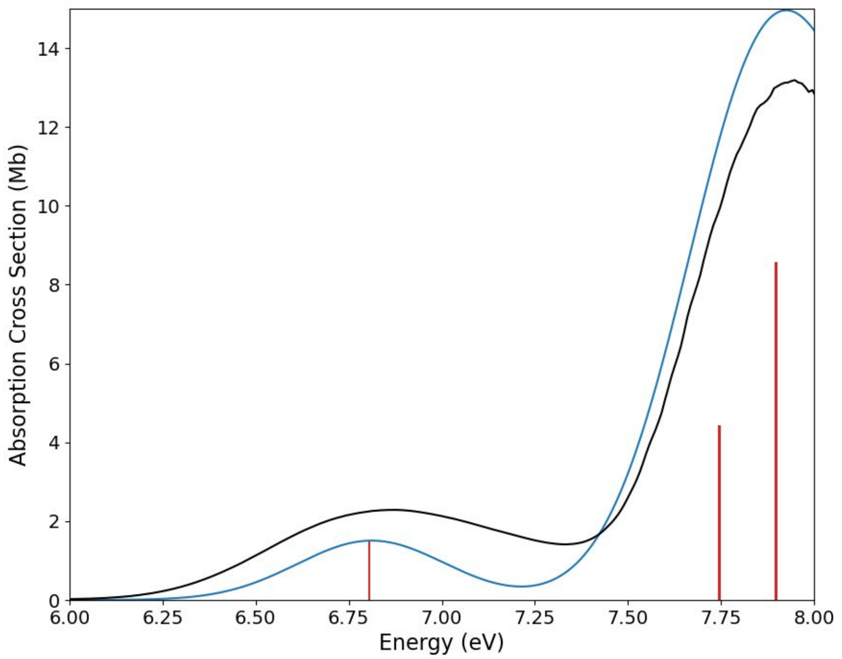

TD-M11/d-aug-cc-pVTZ for 2-Propanol

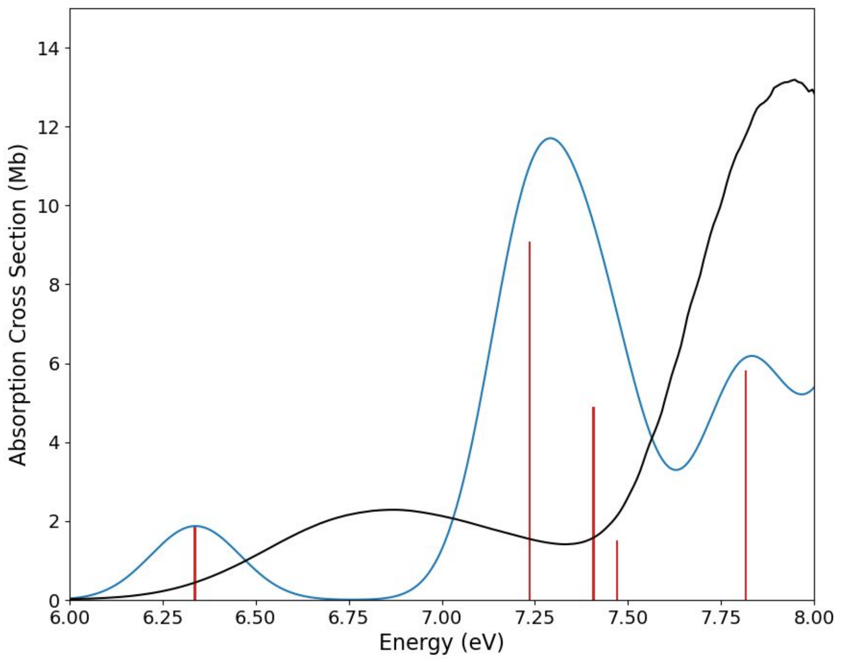

TD-PBE0/d-aug-cc-pVTZ for 2-Propanol

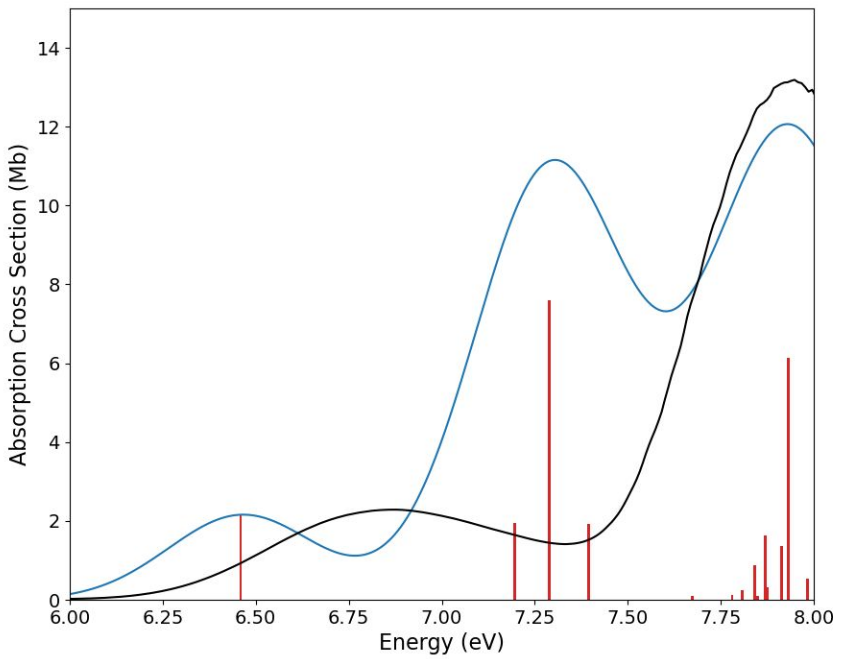

TD-wB97x-D/d-aug-cc-pVTZ for 2-Propanol

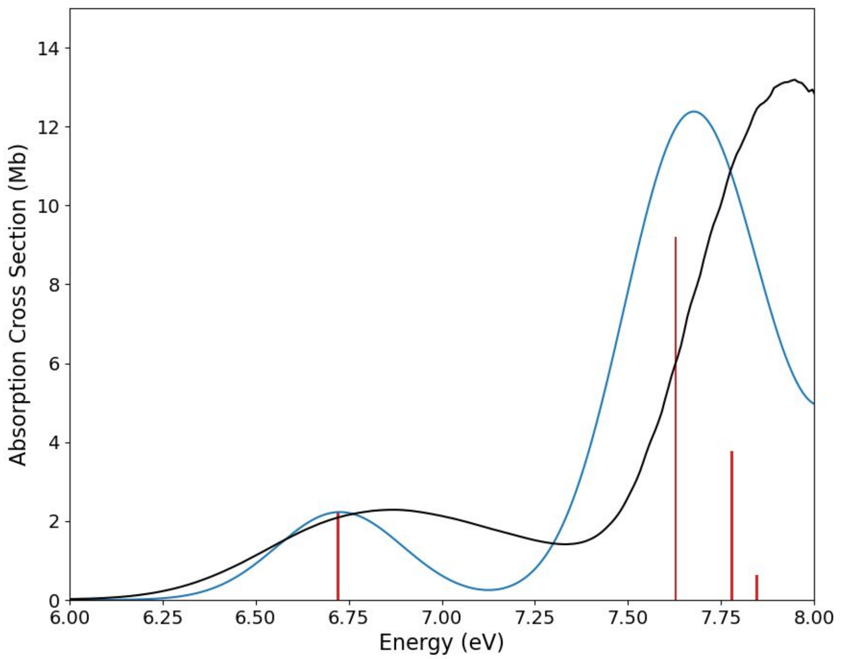

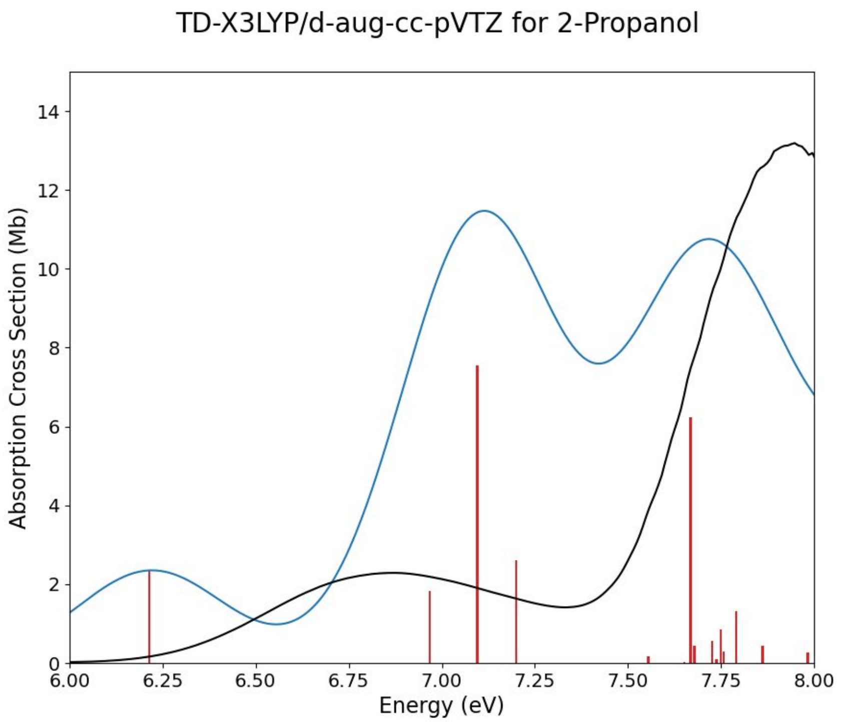
TD-X3LYP/d-aug-cc-pVTZ for 2-Propanol
Absorption Cross Section (Mb)
Energy (eV)
0
2
4
6
8
10
12
14
6.00
6.25
6.50
6.75
7.00
7.25
7.50
7.75
8.00

19 Dimethyl Ether

Table S73. Optimized Geometry of Dimethyl Ether in Å

	CCSD(T)/d-aug-cc-pVTZ			M06-2X/d-aug-cc-pVTZ		
O	0.0000000000	0.0000000000	-0.5503314741	0.0000000000	0.0000000000	0.5393661760
C	0.0000000000	1.1650926519	0.2520070903	-0.0000000000	1.1629592900	-0.2464983877
C	0.0000000000	-1.1650926519	0.2520070903	0.0000000000	-1.1629592900	-0.2464983877
H	0.0000000000	2.0239230001	-0.4186659672	-0.0000000000	2.0174094908	0.4256274133
H	0.0000000000	-2.0239230001	-0.4186659672	0.0000000000	-2.0174094908	0.4256274133
H	-0.8919866935	1.2045836311	0.8917295210	0.8892074446	1.2083080914	-0.8853398253
H	0.8919866935	1.2045836311	0.8917295210	-0.8892074446	1.2083080914	-0.8853398253
H	0.8919866935	-1.2045836311	0.8917295210	-0.8892074446	-1.2083080914	-0.8853398253
H	-0.8919866935	-1.2045836311	0.8917295210	0.8892074446	-1.2083080914	-0.8853398253

Table S74. Frequencies of Dimethyl Ether in cm^{-1}

CCSD(T)/d-aug-cc-pVTZ	M06-2X/d-aug-cc-pVTZ
204.363	207.415
253.142	269.086
417.227	429.441
951.686	984.289
1121.598	1141.470
1164.947	1175.783
1195.573	1205.879
1203.338	1238.310
1272.847	1280.901
1459.671	1466.053
1490.513	1494.945
1493.890	1498.877
1504.327	1504.846
1507.299	1505.956
1521.425	1522.212
2976.676	3009.431
2984.325	3017.460
3029.122	3058.186
3035.029	3063.347
3125.838	3153.399
3127.520	3154.516

Table S75. Transition Energies of Dimethyl Ether in eV

CCSD(T)/d-aug-cc-pVTZ	M06-2X/d-aug-cc-pVTZ
6.877	6.816
7.520	7.401
7.686	7.491
7.750	7.620
8.068	7.876
8.508	8.282
8.508	8.350
8.577	8.367
8.650	8.453
8.708	8.510
8.816	8.536
8.906	8.659
8.951	8.672
8.952	8.682
9.080	8.832
	8.880
	8.931
	9.061
	9.063

Table S76. Quantitative Metrics for the Bandwidth (γ), cosine similarity (S), relative integral change (RIC), mean signed error (MSE), and mean average error (MAE) for the band shape of dimethyl ether compared to experiment.

Method	γ	S	RIC	MSE	MAE
B3LYP	0.15	0.992	0.127	0.223	0.570
BH&HLYP	0.17	0.752	0.831	1.076	3.649
BMK	0.15	0.917	0.451	0.790	2.004
CAM-B3LYP	0.17	0.752	0.650	-0.843	2.848
CC	0.12	0.909	0.385	-0.661	1.682
HSE	0.2	0.824	0.523	-0.402	2.329
M06-2X	0.15	0.980	0.194	-0.113	0.837
M11	0.17	0.957	0.295	0.278	1.289
PBE0	0.2	0.863	0.456	-0.255	2.013
wB97x-D	0.15	0.830	0.548	-0.710	2.392
X3LYP	0.13	0.977	0.253	0.571	1.107

Figure S19. Spectra of Dimethyl Ether

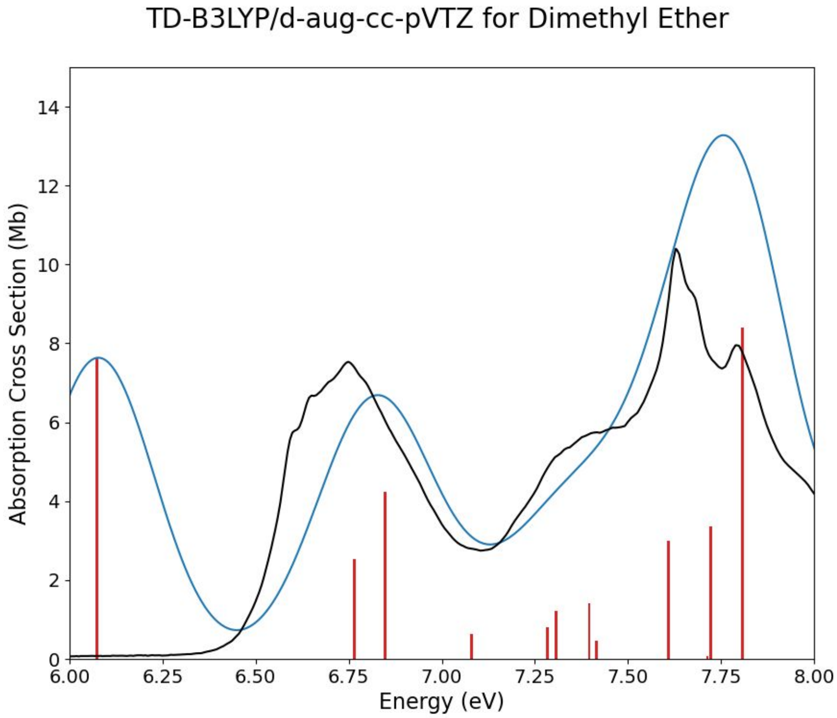

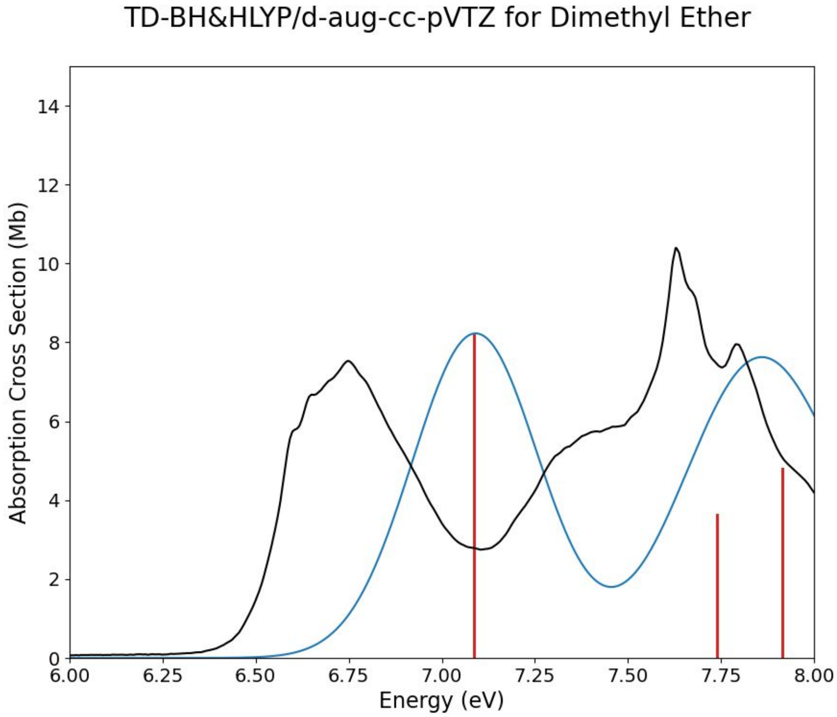

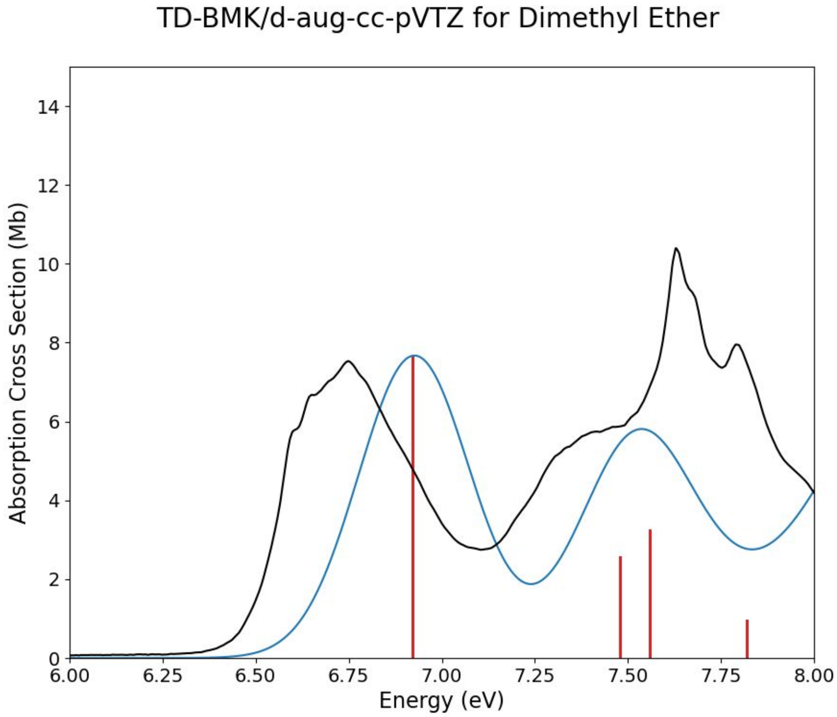
TD-BMK/d-aug-cc-pVTZ for Dimethyl Ether
Absorption Cross Section (Mb)
0
2
4
6
8
10
12
14
6.00
6.25
6.50
6.75
7.00
7.25
7.50
7.75
8.00
Energy (eV)

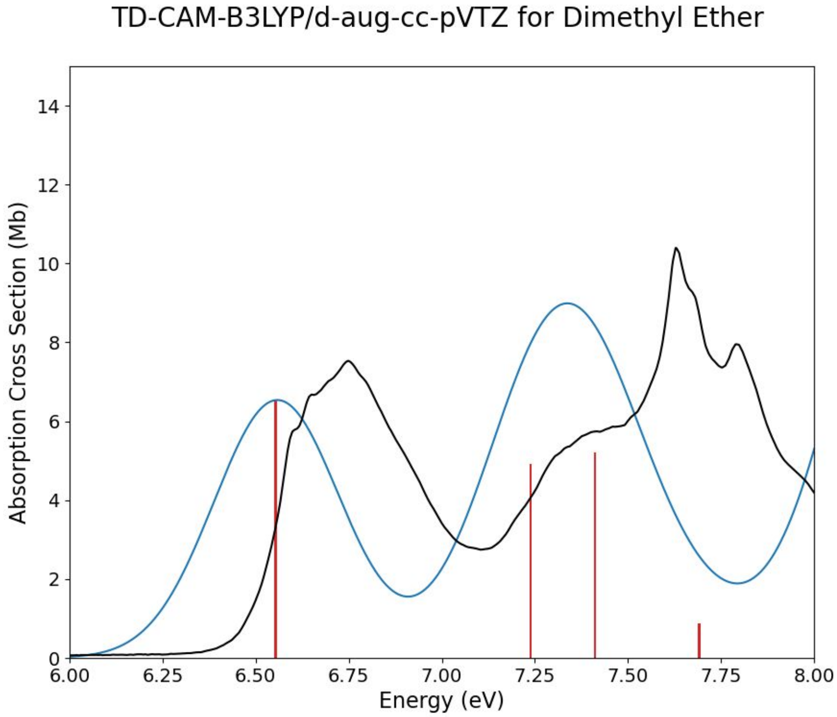
TD-CAM-B3LYP/d-aug-cc-pVTZ for Dimethyl Ether
Absorption Cross Section (Mb)
0
2
4
6
8
10
12
14
6.00
6.25
6.50
6.75
7.00
7.25
7.50
7.75
8.00
Energy (eV)

EOM-CCSD/d-aug-cc-pVTZ for Dimethyl Ether

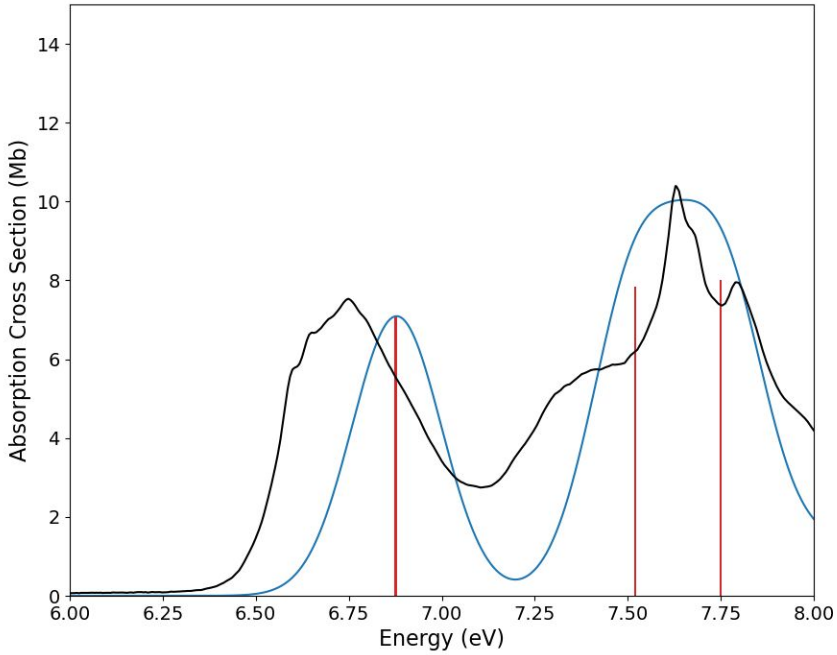

TD-HSE/d-aug-cc-pVTZ for Dimethyl Ether

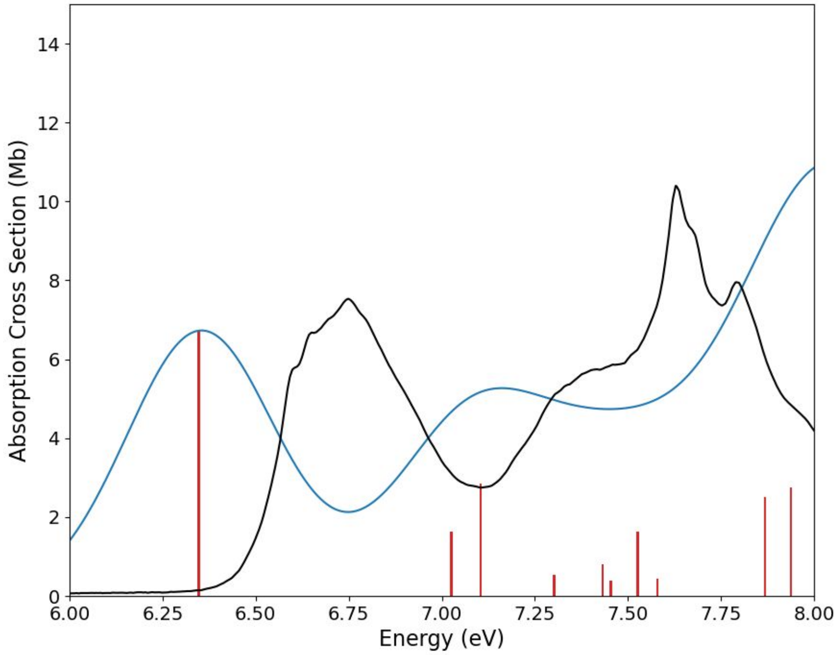

TD-M06-2X/d-aug-cc-pVTZ for Dimethyl Ether

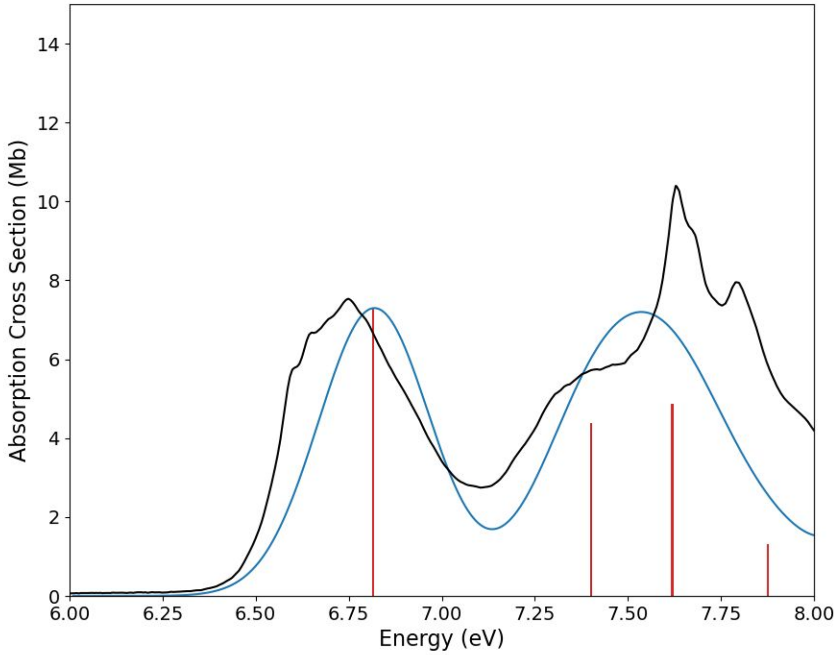

TD-M11/d-aug-cc-pVTZ for Dimethyl Ether

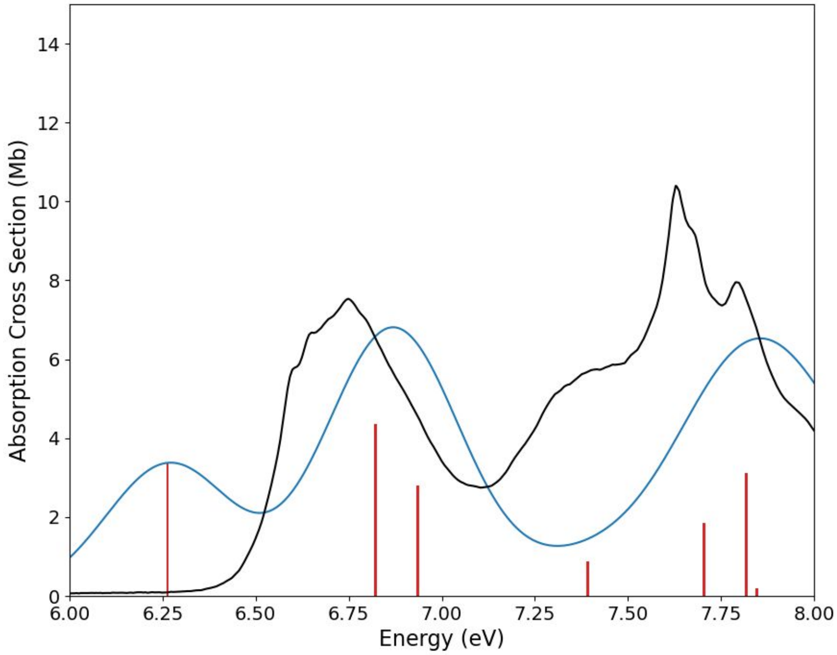

TD-PBE0/d-aug-cc-pVTZ for Dimethyl Ether

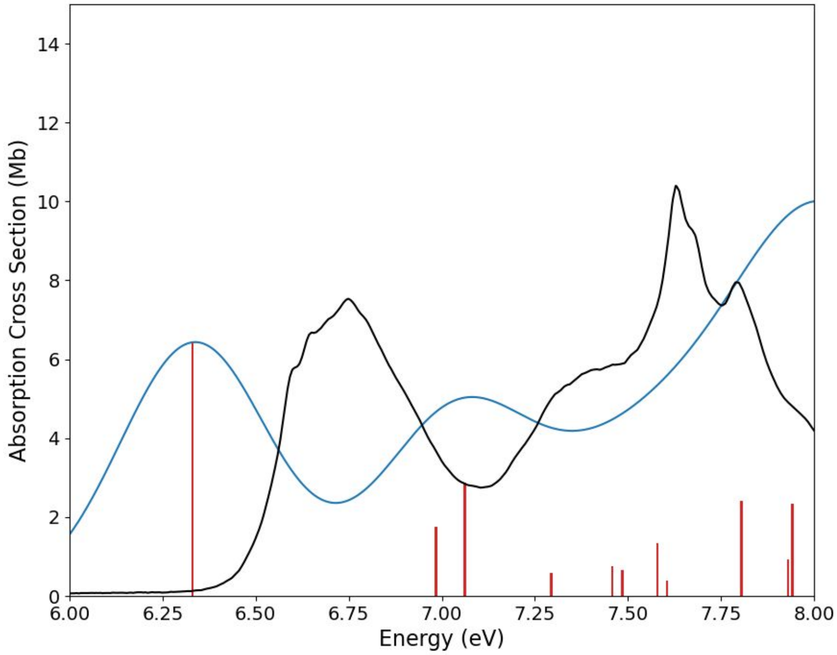

TD-wB97x-D/d-aug-cc-pVTZ for Dimethyl Ether

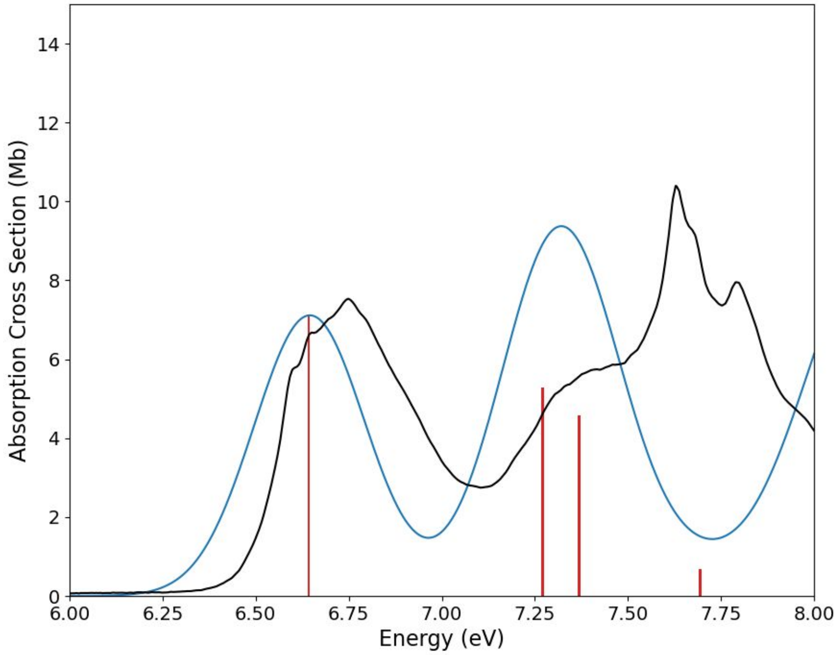

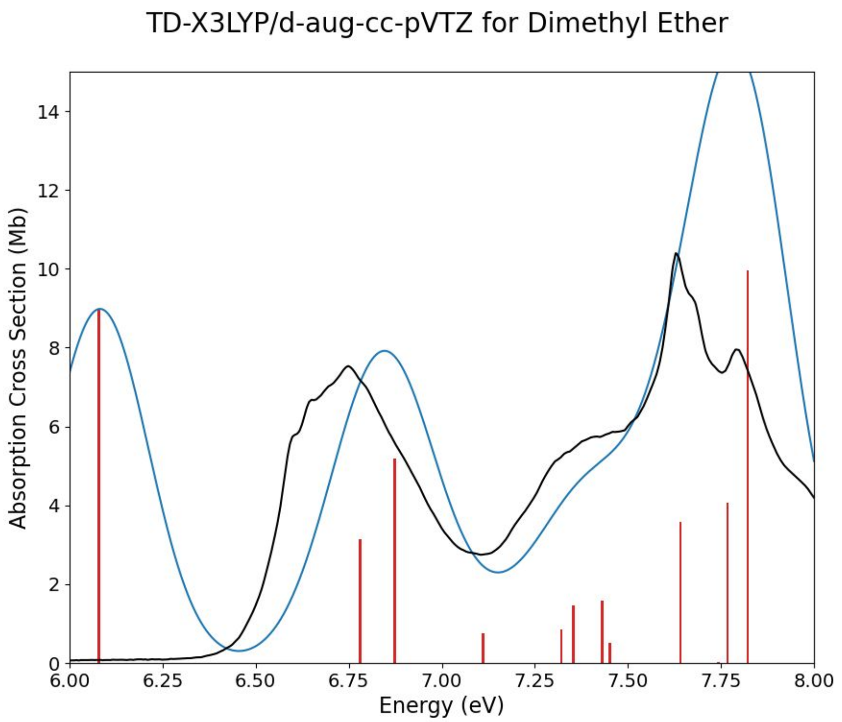
TD-X3LYP/d-aug-cc-pVTZ for Dimethyl Ether
Absorption Cross Section (Mb)
Energy (eV)
0
2
4
6
8
10
12
14
6.00
6.25
6.50
6.75
7.00
7.25
7.50
7.75
8.00

20 QUEST Database Comparisons

Table S77. Difference between EOM-CCSD/d-aug-cc-pVTZ and the chosen TD-DFT functionals for the three lowest singlet transitions of water.

Method	$^1A_1 \rightarrow {}^1B_1$	$^1A_1 \rightarrow {}^1A_2$	$^1A_1 \rightarrow {}^1A_1$
B3LYP	0.682	1.058	1.110
BH&HLYP	-0.136	0.059	0.082
BMK	-0.156	0.234	0.312
CAM-B3LYP	0.466	0.669	0.732
HSE	0.376	0.725	0.786
M06-2X	0.136	0.431	0.367
M11	0.637	1.216	1.009
PBE0	0.411	0.804	0.889
wB97x-D	0.340	0.719	0.840
X3LYP	0.690	1.032	1.078

Table S78. Difference between EOM-CCSD/d-aug-cc-pVTZ and the chosen TD-DFT functionals for the lowest singlet transitions of ethene.

Method	$^1A_g \rightarrow {}^1B_{3u}$	$^1A_g \rightarrow {}^1B_{1u}$	$^1A_g \rightarrow {}^1B_{1g}$
B3LYP	0.755	0.574	0.882
BH&HLYP	0.375	0.395	0.459
BMK	0.219	0.326	0.382
CAM-B3LYP	0.410	0.415	0.476
HSE	0.470	0.415	0.588
M06-2X	0.414	0.398	0.551
M11	0.736	0.547	0.942
PBE0	0.513	0.429	0.644
wB97x-D	0.338	0.378	0.429
X3LYP	0.777	0.575	0.889

Table S79. Difference between EOM-CCSD/d-aug-cc-pVTZ and the chosen TD-DFT functionals for the lowest singlet transitions of acetaldehyde.

Method	$^1A' \rightarrow {}^1A''$
B3LYP	0.020
BH&HLYP	-0.274
BMK	0.026
CAM-B3LYP	-0.033
HSE	-0.021
M06-2X	0.215
M11	0.333
PBE0	-0.021
wB97x-D	-0.038
X3LYP	0.008

Table S80. Difference between EOM-CCSD/d-aug-cc-pVTZ and the chosen TD-DFT functionals for the five lowest singlet transitions of acetone.

Method	$^1A_1 \rightarrow {}^1A_2$	$^1A_1 \rightarrow {}^1B_2$	$^1A_1 \rightarrow {}^1A_2$	$^1A_1 \rightarrow {}^1A_1$	$^1A_1 \rightarrow {}^1B_2$
B3LYP	0.076	0.832	0.864	1.063	0.875
BH&HLYP	-0.273	-0.253	-0.220	-0.109	-0.189
BMK	0.052	-0.115	0.154	0.253	0.178
CAM-B3LYP	-0.020	0.224	0.229	0.306	0.258
HSE	0.027	0.569	0.618	0.806	0.646
M06-2X	0.225	0.037	0.144	0.158	0.128
M11	0.317	0.529	0.835	0.673	0.708
PBE0	0.024	0.564	0.652	0.813	0.678
wB97x-D	-0.021	0.109	0.303	0.305	0.306
X3LYP	0.059	0.829	0.835	1.027	0.850

Table S81. Difference between EOM-CCSD/d-aug-cc-pVTZ and the QUEST database for water.

Method	$^1A_1 \rightarrow {}^1B_1$	$^1A_1 \rightarrow {}^1A_2$	$^1A_1 \rightarrow {}^1A_1$
TBE/aug-cc-pVTZ	-0.04	-0.07	-0.12
TBE(Full)/CBS	-0.12	-0.13	-0.10
ADC(2)/aug-cc-pVTZ	0.40	0.50	0.35
ADC(2.5)/aug-cc-pVTZ	0.07	0.10	0.00
ADC(3)/aug-cc-pVTZ	-0.26	-0.29	-0.35
CC2/aug-cc-pVTZ	0.35	0.45	0.29
CC3/6-31+G(d)	-0.74	-1.40	-0.97
CC3/aug-cc-pVDZ	0.07	0.05	-0.05
CC3/aug-cc-pVQZ	-0.07	-0.09	-0.13
CC3/aug-cc-pVTZ	-0.02	-0.04	-0.10
CC3/d-aug-cc-pVQZ	-0.07	-0.08	-0.07
CC3/d-aug-cc-pVTZ	-0.02	-0.03	-0.02
CC3/t-aug-cc-pVQZ	-0.06	-0.08	-0.06
CC3(Full)/aug-cc-pV5Z	-0.09	-0.10	-0.13
CC3(Full)/aug-cc-pVQZ	-0.08	-0.09	-0.13
CC3(Full)/d-aug-cc-pV5Z	-0.09	-0.10	-0.08
CC3(Full)/d-aug-cc-pVQZ	-0.08	-0.08	-0.07
CC3(Full)/t-aug-cc-pVQZ	-0.08	-0.08	-0.07
CCSD/aug-cc-pVTZ	-0.02	-0.02	-0.09
CCSDR(3)/aug-cc-pVTZ	-0.02	-0.04	-0.09
CCSDT/6-31+G(d)	-0.74	-1.39	-0.96
CCSDT/aug-cc-pVDZ	0.08	0.06	-0.03
CCSDT/aug-cc-pVQZ	-0.06	-0.07	-0.11
CCSDT/aug-cc-pVTZ	-0.01	-0.03	-0.08
CCSDT-3/aug-cc-pVTZ	-0.03	-0.04	-0.10
CCSDTQ/6-31+G(d)	-0.75	-1.40	-0.97
CCSDTQ/aug-cc-pVDZ	0.05	0.03	-0.07
CCSDTQP/aug-cc-pVDZ	0.05	0.02	-0.07
CIS(D)/aug-cc-pVTZ	0.41	0.42	0.35
CIS(D∞)/aug-cc-pVTZ	0.40	0.50	0.35
FCI/aug-cc-pVDZ	0.05	0.02	-0.07
FCI/aug-cc-pVQZ	-0.10	-0.12	-0.15
STEOM-CCSD/aug-cc-pVTZ	0.02	-0.03	-0.05
experimental/CBS	0.17	0.14	0.20
CCSD(T)(a)*/aug-cc-pVTZ	-0.02	-0.04	-0.09
EOM-MP2/aug-cc-pVTZ	0.00	-0.01	-0.04
SCS-CC2/aug-cc-pVTZ	0.20	0.26	0.13
SOS-ADC(2) [QC]/aug-cc-pVTZ	0.35	0.39	0.28
SOS-ADC(2) [TM]/aug-cc-pVTZ	0.18	0.22	0.11
SOS-CC2/aug-cc-pVTZ	0.13	0.17	0.05

Table S82. Difference between EOM-CCSD/d-aug-cc-pVTZ and the QUEST database for ethene.

Method	$^1A_g \rightarrow {}^1B_{3u}$	$^1A_g \rightarrow {}^1B_{1u}$	$^1A_g \rightarrow {}^1B_{1g}$
TBE/aug-cc-pVTZ	0.01	0.05	-0.03
TBE(Full)/CBS	-0.04	0.09	-0.04
ADC(2)/aug-cc-pVTZ	0.06	0.07	0.06
ADC(2.5)/aug-cc-pVTZ	0.14	0.18	0.13
ADC(3)/aug-cc-pVTZ	0.23	0.29	0.21
CC2/aug-cc-pVTZ	0.11	0.06	0.10
CC3/6-31+G(d)	-0.32	-0.16	-0.24
CC3/aug-cc-pVDZ	0.11	0.04	0.08
CC3/aug-cc-pVQZ	0.02	0.08	0.01
CC3/aug-cc-pVTZ	0.05	0.07	0.02
CC3/d-aug-cc-pVQZ	0.03	0.08	0.02
CC3(Full)/aug-cc-pV5Z	0.01	0.07	0.00
CC3(Full)/aug-cc-pVQZ	0.01	0.07	0.00
CC3(Full)/d-aug-cc-pV5Z	0.01	0.08	0.00
CC3(Full)/d-aug-cc-pVQZ	0.02	0.05	0.01
CCSD/aug-cc-pVTZ	-0.02	-0.04	-0.03
CCSDR(3)/aug-cc-pVTZ	0.05	0.09	0.03
CCSDT/6-31+G(d)	-0.33	-0.17	-0.25
CCSDT/aug-cc-pVDZ	0.11	0.04	0.07
CCSDT/aug-cc-pVTZ	0.03	0.06	0.01
CCSDT-3/aug-cc-pVTZ	0.04	0.06	0.02
CCSDTQ/6-31+G(d)	-0.32	-0.16	-0.24
CCSDTQ/aug-cc-pVDZ	0.10	0.05	0.06
CIS(D)/aug-cc-pVTZ	0.05	0.03	0.04
CIS(D∞)/aug-cc-pVTZ	0.06	0.06	0.06
FCI/aug-cc-pVDZ	0.09	0.05	0.05
STEOM-CCSD/aug-cc-pVTZ	-0.02		-0.05
experimental/CBS	0.29	0.38	0.25
CCSD(T)(a)*/aug-cc-pVTZ	0.05	0.08	0.03
EOM-MP2/aug-cc-pVTZ	0.01	0.10	0.01
SCS-CC2/aug-cc-pVTZ	0.01	0.00	0.00
SOS-ADC(2) [QC]/aug-cc-pVTZ	0.01	0.10	-0.01
SOS-ADC(2) [TM]/aug-cc-pVTZ	-0.08	0.00	-0.09
SOS-CC2/aug-cc-pVTZ	-0.04	-0.02	-0.06

Table S83. Difference between EOM-CCSD/d-aug-cc-pVTZ and the QUEST database for acetaldehyde.

Method	$^1A' \rightarrow ^1A''$
TBE/aug-cc-pVTZ	0.02
TBE(Full)/CBS	0.02
ADC(2)/aug-cc-pVTZ	0.09
ADC(2.5)/aug-cc-pVTZ	0.06
ADC(3)/aug-cc-pVTZ	0.04
CC2/aug-cc-pVTZ	-0.08
CC3/6-31+G(d)	-0.06
CC3/aug-cc-pVDZ	-0.01
CC3/aug-cc-pVQZ	0.01
CC3/aug-cc-pVTZ	0.02
CC3(Full)/aug-cc-pVQZ	0.02
CCSD/aug-cc-pVTZ	-0.03
CCSDR(3)/aug-cc-pVTZ	0.02
CCSDT/6-31+G(d)	-0.04
CCSDT/aug-cc-pVDZ	0.01
CCSDT/aug-cc-pVTZ	0.04
CCSDT-3/aug-cc-pVTZ	0.01
CIS(D)/aug-cc-pVTZ	-0.03
CIS(D∞)/aug-cc-pVTZ	0.09
FCI/aug-cc-pVDZ	-0.01
STEOM-CCSD/aug-cc-pVTZ	0.08
experimental/CBS	0.06
CCSD(T)(a)*/aug-cc-pVTZ	0.02
EOM-MP2/aug-cc-pVTZ	0.02
SCS-CC2/aug-cc-pVTZ	-0.17
SOS-ADC(2) [QC]/aug-cc-pVTZ	0.15
SOS-ADC(2) [TM]/aug-cc-pVTZ	-0.06
SOS-CC2/aug-cc-pVTZ	-0.21

Table S84. Difference between EOM-CCSD/d-aug-cc-pVTZ and the QUEST database for acetone.

Method	$^1A_1 \rightarrow {}^1A_2$	$^1A_1 \rightarrow {}^1B_2$	$^1A_1 \rightarrow {}^1A_2$	$^1A_1 \rightarrow {}^1B_2$	$^1A_1 \rightarrow {}^1B_2$
TBE/aug-cc-pVTZ	0.03	0.13	0.03	0.09	-0.04
TBE(Full)/CBS	0.02	0.08	0.06	0.05	-0.05
ADC(2)/aug-cc-pVTZ	0.13	0.72	0.69	0.75	0.59
ADC(2.5)/aug-cc-pVTZ	0.06	0.20	0.14	0.21	0.08
ADC(3)/aug-cc-pVTZ	0.00	-0.32	-0.40	-0.32	-0.43
CC2/aug-cc-pVTZ	-0.05	0.68	0.66	0.71	0.56
CC3/6-31+G(d)	-0.05	-0.06	-0.33	-0.21	-0.29
CC3/aug-cc-pVDZ	0.00	0.28	0.13	0.21	0.02
CC3/aug-cc-pVQZ	0.01	0.11	0.02	0.08	-0.02
CC3/aug-cc-pVTZ	0.02	0.16	0.05	0.12	-0.01
CC3(Full)/aug-cc-pVQZ	0.02	0.10	0.01	0.07	-0.03
CCSD/aug-cc-pVTZ	-0.04	0.00	-0.07	-0.03	-0.12
CCSDR(3)/aug-cc-pVTZ	0.02	0.13	0.03	0.08	-0.03
CCSDT/6-31+G(d)	-0.02	-0.05	-0.33	-0.21	-0.29
CCSDT/aug-cc-pVDZ	0.02	0.29	0.14	0.22	0.03
CCSDT/aug-cc-pVTZ	0.04	0.17	0.07	0.12	0.00
CCSDT-3/aug-cc-pVTZ	0.01	0.09	-0.01	0.05	-0.07
CCSDTQ/6-31+G(d)	-0.03	-0.09	-0.37	-0.24	-0.33
CIS(D)/aug-cc-pVTZ	-0.01	0.68	0.67	0.56	0.65
STEOM-CCSD/aug-cc-pVTZ	0.10	-0.03		-0.08	
experimental/CBS	0.02	0.23	0.14	0.19	0.13
CCSD(T)(a)*/aug-cc-pVTZ	0.02	0.13	0.03	0.08	-0.03
EOM-MP2/aug-cc-pVTZ	-0.02	-0.01	-0.10	-0.05	-0.16
SCS-CC2/aug-cc-pVTZ	-0.14	0.22	0.21	0.27	0.15
SOS-ADC(2) [QC]/aug-cc-pVTZ	0.19	0.25	0.23	0.28	0.17
SOS-ADC(2) [TM]/aug-cc-pVTZ	-0.02	-0.05	0.03	0.08	-0.03
SOS-CC2/aug-cc-pVTZ	-0.18	0.00	-0.01	0.04	-0.06
FCI/6-31+G(d)	-0.10				
